# Supplementary material for: Spatial isoform sequencing at single-cell resolution reveals cell-type-specific spatial isoform variability in multiple brain cell types
Source: Nat Methods. 2026 Sep 4;23(9):1869–81. doi: 10.1038/s41592-026-03211-w (PMC13545012; doi:10.1038/s41592-026-03211-w)
Supplement: Supplementary file 1 [file 41592_2026_3211_MOESM1_ESM.pdf]

nature methods

Supplementary information https://doi.org/10.1038/s41592-026-03211-w

Spatial isoform sequencing at single-cell resolution reveals cell-type-specific spatial isoform variability in multiple brain cell types

In the format provided by the authors and unedited

Supplementary Material

Content

Supplementary Tables 2

Supplementary Figures 16

Table S1. Stereo-seq short-read statistics

	Sample 1	Sample 2
Number of segmented cells	91,690	108,845
Mean number of barcodes per cell	753	642
Median number of barcodes per cell	702	586
Mean number of UMIs per cell	1,353	925
Median number of UMIs per cell	1,144	792
Mean number of genes per cell	678	499
Median number of genes per cell	614	452

Table S2. Stereo-seq ONT barcode calling statistics

	Sample 1 (AE)	Sample 1 (3.3K)	Sample 2 (AE)
Total reads	207,282,773	87,520,126	58,799,050
cDNAs extracted	218,287,258	88,514,020	62,775,129
PolyT detected	182,228,343	56,831,671	54,111,424
Primer detected	159,898,493	39,694,971	48,318,281
Linker detected	160,091,513	39,749,743	48,380,796
Barcode detected	130,360,866	31,182,006	40,354,173
PolyT detected	83.48%	64.21%	86.20%
Primer detected	73.25%	44.85%	76.97%
Linker detected	73.34%	44.91%	77.07%
Barcode detected	59.72%	35.23%	64.28%

Table S3. Stereo-seq ONT barcode calling statistics: the effect of de-concatenation barcode calling algorithm

Dataset	S1 (AE)	S2 (AE)
Total reads	207,282,775	58,799,051
cDNAs extracted	218,287,260	62,775,130
Barcoded cDNAs	129,178,416	40,354,173
Barcoded cDNAs without deconcatenation	118,173,931	36,378,094
Reads with >= 2 cDNAs	10,458,285	3,903,874
Barcoded cDNAs in these reads	21,462,770	7,879,953
Additional barcoded cDNAs recovered	11,004,485	3,976,079
**Additional barcoded cDNAs recovered (%)**	**9.31%**	**10.93%**

Table S4. Stereo-seq PacBio barcode calling statistics

	Sample 1 (AE)	Sample 2 (AE)
Total reads	19,878,737	17,627,912
cDNAs extracted	20,137,382	17,969,619
PolyT detected	17,365,347	15,487,476
Primer detected	16,951,711	15,368,317
Linker detected	16,952,851	15,369,386
Barcode detected	15,195,233	13,981,555
PolyT detected	86.23%	86.19%
Primer detected	84.18%	85.52%
Linker detected	84.19%	85.53%
Barcode detected	75.46%	77.81%

Table S5. Stereo-seq ONT read-to-isoform assignment and PCR deduplication statistics

	Sample 1 (AE)	Sample 1 (3.3K)	Sample 2 (AE)
Total reads	207,282,773	87,520,126	58,799,050
cDNAs extracted	218,287,258	88,514,020	62,775,129
Barcode detected	130,360,866	31,182,006	40,354,173
**Of them**			
Uniquely assigned	100,571,599	27,204,522	30,483,380
Uniquely assigned and spliced	79,030,635	25,712,877	24,159,223
Unique gene-barcode pairs	35,128,134	7,500,561	14,910,661
Reads after deduplication	36,728,127	7,923,634	15,295,364
Spliced reads after deduplication	25,916,277	7,016,938	11,134,293
Barcode detected	59.72%	35.23%	64.28%
**Of them**			
Uniquely assigned	77.15%	87.24%	75.54%
Uniquely assigned and spliced	60.62%	82.46%	59.87%
Reads after deduplication	26.95%	24.05%	36.95%
Spliced reads after deduplication	28.17%	25.41%	37.90%

Table S6. Stereo-seq PacBio read-to-isoform assignment and PCR deduplication statistics

	Sample 1 (AE)	Sample 2 (AE)
Total reads	19,878,737	17,627,912
cDNAs extracted	20,137,382	17,969,619
Barcode detected	15,195,233	13,981,555
**Of them**		
Uniquely assigned	12,427,208	11,407,337
Uniquely assigned and spliced	9,852,363	9,344,667
Unique gene-barcode pairs	8,479,274	7,453,247
Reads after deduplication	8,534,498	7,489,921
Spliced reads after deduplication	6,500,576	5,845,919
Barcode detected	75.46%	77.81%
**Of them**		
Uniquely assigned	81.78%	81.59%
Uniquely assigned and spliced	64.84%	66.84%
Reads after deduplication	55.80%	53.31%
Spliced reads after deduplication	56.17%	53.57%

Table S7. Stereo-seq ONT statistics after PCR deduplication

	Sample 1 (AE)	Sample 1 (3.3K)	Sample 2 (AE)
Spliced reads after deduplication	25,916,277	7,016,938	11,134,293
Overlapping with segmented cell in correct hemisphere	10,741,771	3,054,136	3,104,550
Isoform assigned	8,006,816	2,062,768	2,406,135
Unique genes detected	22,434	12,198	18,003
Mean number of reads per gene	479	250	172
Unique isoforms detected	49,860	22,060	36,273
Mean number of reads per isoform	161	94	66
Mean number of spliced reads per cell	177	50	54
Median number of spliced reads per cell	140	39	44
Mean percentage of spliced reads assigned to a gene per cell	100%	100%	100%
Mean number of genes detected per cell	152	43	50
Median number of genes detected per cell	125	35	42
Mean percentage of spliced reads assigned to an isoform per cell	75.5%	67.2%	77.4%
Mean number of isoforms detected per cell	116	30	39
Median number of isoforms detected per cell	95	24	33

Table S8. Stereo-seq Pacbio statistics after PCR deduplication

	Sample 1 (AE)	Sample 2 (AE)
Spliced reads after deduplication	6,500,576	5,845,919
Overlapping with segmented cell in correct hemisphere	2,855,135	1,668,435
Isoform assigned	2,283,296	1,339,239
Unique genes detected	17,312	15,980
Mean number of reads per gene	165	104
Unique isoforms detected	34,879	30,142
Mean number of reads per isoform	65	44
Mean number of spliced reads per cell	47	29
Median number of spliced reads per cell	37	23
Mean percentage of spliced reads assigned to a gene per cell	100%	100%
Mean number of genes detected per cell	45	28
Median number of genes detected per cell	36	23
Mean percentage of spliced reads assigned to an isoform per cell	79.8%	80.2%
Mean number of isoforms detected per cell	36	22
Median number of isoforms detected per cell	28	19

Table S9. A comparison of read statistics between samples generated using the Stereo-seq, Visium HD, and Visium platforms. Informative reads are defined as UMI-deduplicated, spliced reads with an assigned isoform and overlapping a segmented cell or spot in the correct hemisphere. However, for the Lebrigand et al paper, a subset, containing 44M and 99M reads, respectively, was uploaded to SRA. The uploaded data likely represent reads already enriched for informative molecules, as the number of reads after UMI deduplication is consistent with those reported in their Supplementary Materials.

	Stereo-seq Sample 1 (AE - ONT)	Visium HD V1 (ONT)	Visium HD V1 (PacBio)	Visium CBS1	Visium CBS2
Sequenced reads	207,282,773	98,713,075	126,023,947	173,996,000	286,458,673
Analysed reads	207,282,773	98,713,075	126,023,947	43,899,878	98,892,086
Barcode detected	130,360,866	49,089,229	84,196,089	36,625,448	80,219,056
Uniquely assigned	100,571,599	38,561,039	66,968,834	31,032,886	67,505,940
Uniquely assigned and spliced	79,030,635	25,987,614	53,617,739	19,972,628	48,768,593
Reads after deduplication	36,728,127	22,313,578	18,616,621	17,581,867	18,489,755
Spliced reads after deduplication	25,916,277	14,740,254	13,620,397	10,929,678	12,189,522
Informative reads	8,006,816	8,354,635	6,982,558	8,204,667	8,749,685

Table S10. Differential relative isoform expression results for all cells for all tested genes. See Supplementary File.

Table S11. Differential relative isoform expression results for excitatory neurons for all tested genes. See Supplementary File.

Table S12. Differential relative isoform expression results for inhibitory neurons for all tested genes. See Supplementary File.

Table S13. Differential relative isoform expression results for oligodendrocytes for all tested genes. See Supplementary File.

Table S14. Differential relative isoform expression results for astrocytes for all tested genes. See Supplementary File.

Table S15. Mapping between the original and new labels for the Lebrigand et al. dataset.

**Original label**	**New label**
CA1/CA2, CA2, DG, Hippocampus area	Hippocampus
Iscortex-1, Isocortex-2, Olfactory area, Retrosplenial area,	Cortex
Fiber tracts	White Matter
Thalamus	Thalamus
Midbrain, Hypothalamus	Midbrain

Table S16. Spl-IsoFind results for all cells for all tested isoforms. See Supplementary File.

Table S17. Spl-IsoFind results for excitatory neurons for all tested isoforms. See Supplementary File.

Table S18. Spl-IsoFind results for inhibitory neurons for all tested isoforms. See Supplementary File.

Table S19. Spl-IsoFind results for oligodendrocytes for all tested isoforms. See Supplementary File.

Table S20. Spl-IsoFind results for astrocytes for all tested isoforms. See Supplementary File.

Table S21. Visium HD cell segmentation statistics

	V1
Number of segmented cells	77,821
Mean number of barcodes per cell	46
Median number of barcodes per cell	42

Table S22. Read length statistics for Stereo-seq and Visium HD

Sample	Total reads	Mean length, bp	N50, bp	N90, bp
**Stereo-seq ONT**				
Sample 1 (AE)	207,282,773	1,282	1,409	851
Sample 1 (3.3K)	87,520,126	1,416	1,486	1,032
Sample 2 (AE)	58,799,050	1,462	1,659	971
**Stereo-seq PacBio**				
Sample 1 (AE)	19,878,737	1,099	1,270	737
Sample 2 (AE)	17,627,912	1,223	1,511	814
**Visium HD V1 ONT**				
	98,713,075	1,089	1,193	702
**Visium HD V1 PacBio**				
	126,023,947	1,049	1,096	762
**Visium HD V2 ONT**				
PBC44593	161,221,491	622	767	341
PBC44760	132,795,836	626	769	342
PBC49262	140,386,479	624	768	340
PBC52788	165,541,042	624	771	338
**Visium HD V3 PacBio**				
	108,063,589	694	743	443
	107,529,335	694	743	443
	102,934,038	693	743	443
	101,261,931	694	744	444

Table S23. Visium HD barcode calling statistics using Spl-IsoQuant-2

	V1 (ONT)	V1 (PB)	V2 (ONT)	V3 (PB)
Total reads	98,713,075	126,023,947	599,944,848	545,812,840
Barcode detected	49,089,229	84,196,089	268,552,874	388,488,422
PolyT detected	90,224,485	124,208,263	584,711,646	542,561,819
R1 detected	77,716,763	120,032,663	520,667,619	521,687,965
Barcode detected	49.73%	66.81%	44.76%	71.18%
PolyT detected	91.40%	98.56%	97.46%	99.40%
R1 detected	78.73%	95.25%	86.79%	95.58%

Table S24. Visium HD read-to-isoform assignment and PCR deduplication statistics.

	V1 (ONT, 2μm)	V1 (PB, 2μm)	V2 (ONT, 8μm)	V3 (PB, 8μm)
Total reads	98,713,075	126,023,947	599,944,848	419,788,893
Barcode detected	49,089,229	84,196,089	268,552,874	304,292,333
**Of them**				
Uniquely assigned	38,561,039	66,968,834	180,099,678	286,182,637
Uniquely assigned and spliced	25,987,614	53,617,739	49,918,226	86,331,762
Unique gene-barcode pairs	21,728,795	18,504,392	44,088,597	72,985,533
Reads after deduplication	22,313,578	18,616,621	47,600,985	80,401,514
Spliced reads after deduplication	14,740,254	13,620,397	10,844,620	19,965,368
Barcode detected	49.73%	66.81%	44.76%	72.49%
**Of them**				
Uniquely assigned	78.55%	79.54%	67.06%	94.05%
Uniquely assigned and spliced	52.94%	63.68%	18.59%	28.37%
Reads after deduplication	45.46%	22.11%	17.72%	26.42%
Spliced reads after deduplication	30.03%	16.18%	4.04%	6.56%

Table S25. Visium HD statistics after PCR deduplication

	V1 (ONT)	V1 (PB)	V2 (ONT)	V3 (PB)
Spliced reads after deduplication	14,740,254	13,620,397	10,844,620	19,965,368
Isoform assigned	8,354,635	6,982,558	7,186,605	12,604,203
Unique genes detected	21,211	20,263	27,283	29,319
Mean number of reads per gene	533	521	392	666
Unique isoforms detected	42,357	38,178	46,382	50754
Mean number of reads per isoform	197	183	155	248
Mean number of spliced reads per cell/8um bin	145	136	21	50
Median number of spliced reads per cell/8um bin	111	103	17	35
Mean percentage of spliced reads assigned to a gene per cell/8um bin	100%	100%	100%	100%
Mean number of genes detected per cell/8um bin	127	121	20	46
Median number of genes detected per cell/8um bin	102	96	17	33
Mean percentage of spliced reads assigned to an isoform per cell/8um bin	73.9%	66.0%	67.3%	64.6%
Mean number of isoforms detected per cell/8um bin	96	80	13	30
Median number of isoforms detected per cell/8um bin	76	64	11	21

Table S26. Estimated costs for the individual steps during each experiment. Per type of experiment, we also indicated which steps were included. The total cost per experiment is shown in Table S27. Shared steps (e.g., the Stereo-seq slide) are included for multiple experiments, even when the same slide was used in practice to get a fair estimate of the cost per read (e.g., as in Sample 1 (AE) and Sample 1 (3.3K)). The reported costs are an estimate and may vary across institutes depending on available infrastructure and vendor discounts.

	Est. cost	S1/S2 (AE) ONT	S1/S2 (AE) PB	S1 (3.3K) ONT	V1 ONT	V1 PB	V2 ONT	V3 PB
Stereo-seq slide (including SR seq.)	$5000	1x	1x	1x				
Visium HD slide (excluding SR seq.)	$4500				1x	1x	1x	1x
Long-mol. selection	$35	1x	1x	1x	1x	1x		
Exome enrichment	$155	1x	1x	1x	1x	1x		
3378 gene enrichment	$350			1x				
ONT library prep	$120	1x		1x	1x		1x	
PromethION flow Cell	$1040	1-2x		1x	1x		4x	
PacBio library prep	$475		1x			1x		1x
PacBio SMRT Cell	$1700		1x			1x		4x

Table S27. Estimated total cost per experiment and cost per sequencing output. Costs are reported per 1M total reads and per 100K informative reads. Informative reads are spliced reads with an assigned isoform overlapping a segmented cell in the correct hemisphere and thus suitable for downstream analysis. Estimates are calculated for the complete workflow and for the long-read sequencing part alone.

	Including array prep			Long-read sequencing only			
	Est. total cost	Cost per 1M seq. reads	Cost per 100K informative reads	Est. total cost	Cost per 1M seq. reads	Cost per 100K informative reads	
S1 (AE) - ONT	$7,390	$36	$92	$2,390	$12	$30	
S1 (AE) - PB	$7,365	$368	$323	$2,365	$118	$104	S1 (AE) - PB was sequenced using PacBio Mas-Seq with 16-fold concatenation resulting in fewer reads compared to V1 - PB, which was sequencing with the Kinnex kit with 12-fold concatenation
S1 (3.3K) - ONT	$6,700	$76	$325	$1,700	$19	$82	
S2 (AE) - ONT	$6,350	$108	$264	$1,350	$23	$56	For S2 (AE) - ONT, many reads were mapped to cells in the other hemisphere that we discarded. This largely accounts for the difference compared to S1 (AE) - ONT
S2 (AE) - PB	$7,365	$409	$550	$2,365	$131	$177	S2 (AE) - PB was sequenced using PacBio Mas-Seq with 16-fold concatenation resulting in fewer reads compared to V1 - PB, which was sequencing with the Kinnex kit with 12-fold concatenation
V1 - ONT	$5,850	$59	$70	$1,350	$14	$16	
V1 - PB	$6,865	$54	$98	$2,365	$19	$34	
V2 - ONT	$8,780	$15	$122	$4,280	$7	$60	In comparison to V1-ONT, this did not use exome enrichment and long-molecule selection. As a result, it needed more flow cells, raising the cost
V3 - PB	$11,775	$28	$93	$7,275	$17	$58	In comparison to V1-PB, this did not use exome enrichment and long-molecule selection. As a result, it needed more flow cells, raising the cost

Table S28. Spl-IsoFind results for all cells for all tested isoforms in the Visium HD data. See Supplementary File.

Table S29. Spl-IsoFind results for excitatory neurons for all tested isoforms in the Visium HD data. See Supplementary File.

Table S30. Spl-IsoFind results for inhibitory neurons for all tested isoforms in the Visium HD data. See Supplementary File.

Table S31. Spl-IsoFind results for oligodendrocytes for all tested isoforms in the Visium HD data. See Supplementary File.

Table S32. Spl-IsoFind results for astrocytes for all tested isoforms in the Visium HD data. See Supplementary File.

Table S33. Statistics for Visium HD 3' simulated ONT and PacBio data

	Data	ONT		PacBio	
	Error rate	3.8%		0.1%	
	Method	SpaceRanger	Spl-IsoQuant-2	SpaceRanger	Spl-IsoQuant-2
2um	Precision	94.18%	97.58%	99.61%	99.76%
	Recall	78.88%	76.08%	99.999%	99.26%
8um	Precision	97.50%	99.29%	99.88%	99.93%
	Recall	79.45%	76.39%	99.999%	99.26%

Table S34. List of 3,378 targeted genes.

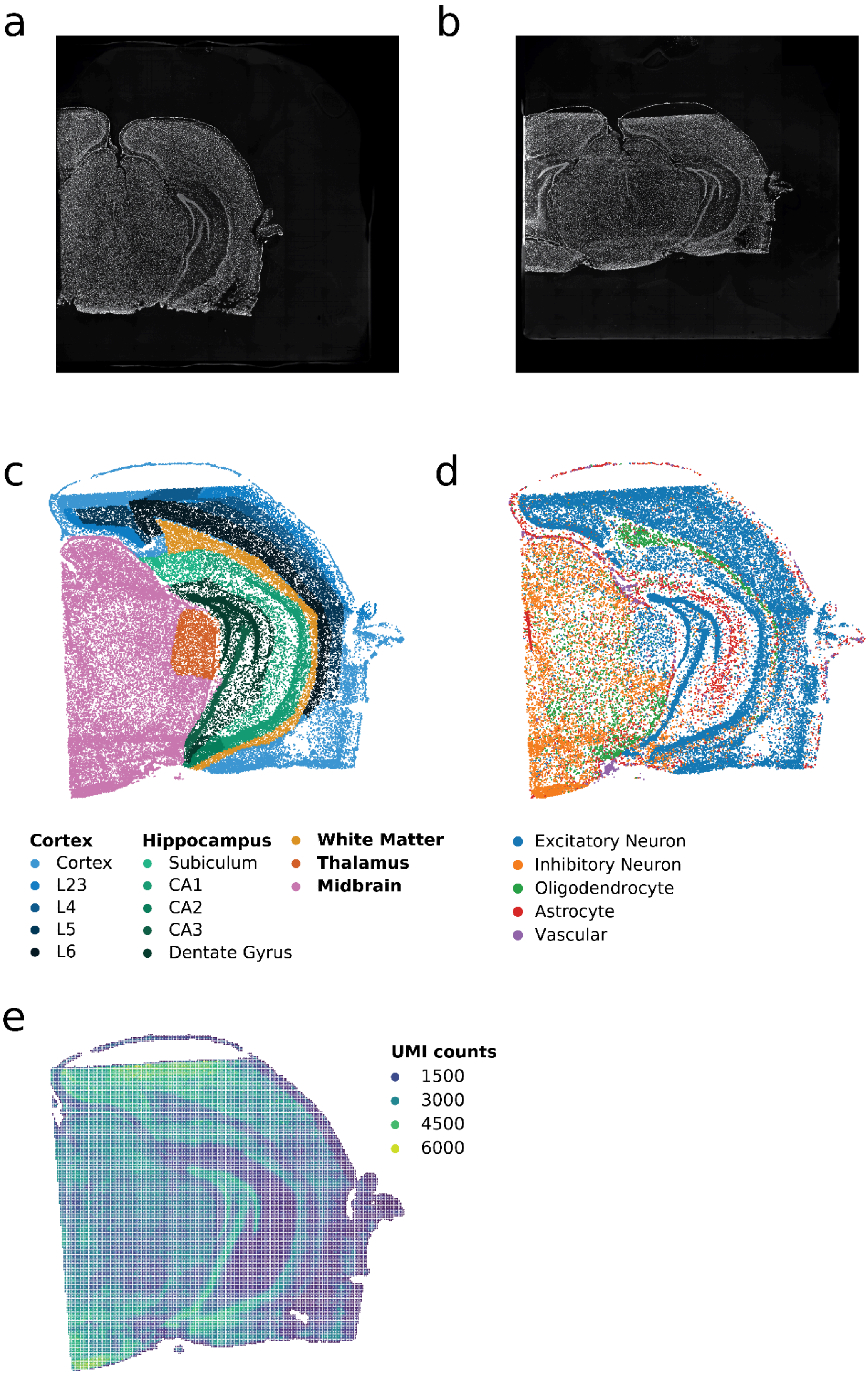

Figure S1. a-b) ssDNA staining of **a)** Sample 1 and **b)** Sample 2. **c)** (Sub)region annotation of Sample 2. **d)** Cell-type annotations of Sample 2. Only cells annotated as singlets and cell types with >100 cells are plotted. **e)** UMI counts per bin (50x50spots) for Sample 2.

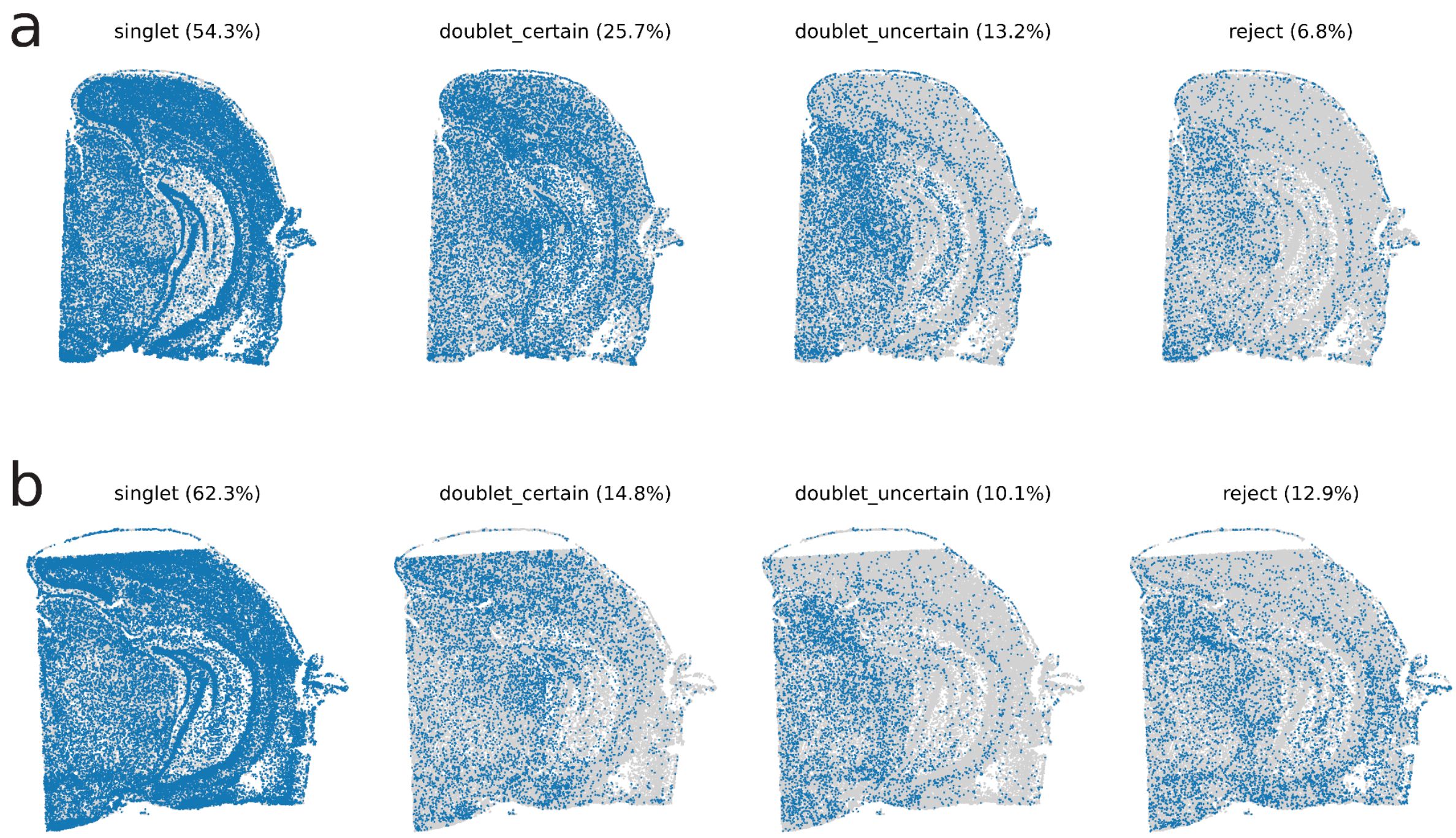

Figure S2 RCTD cell-assignment distribution in **a)** Sample 1 and **b)** Sample 2.

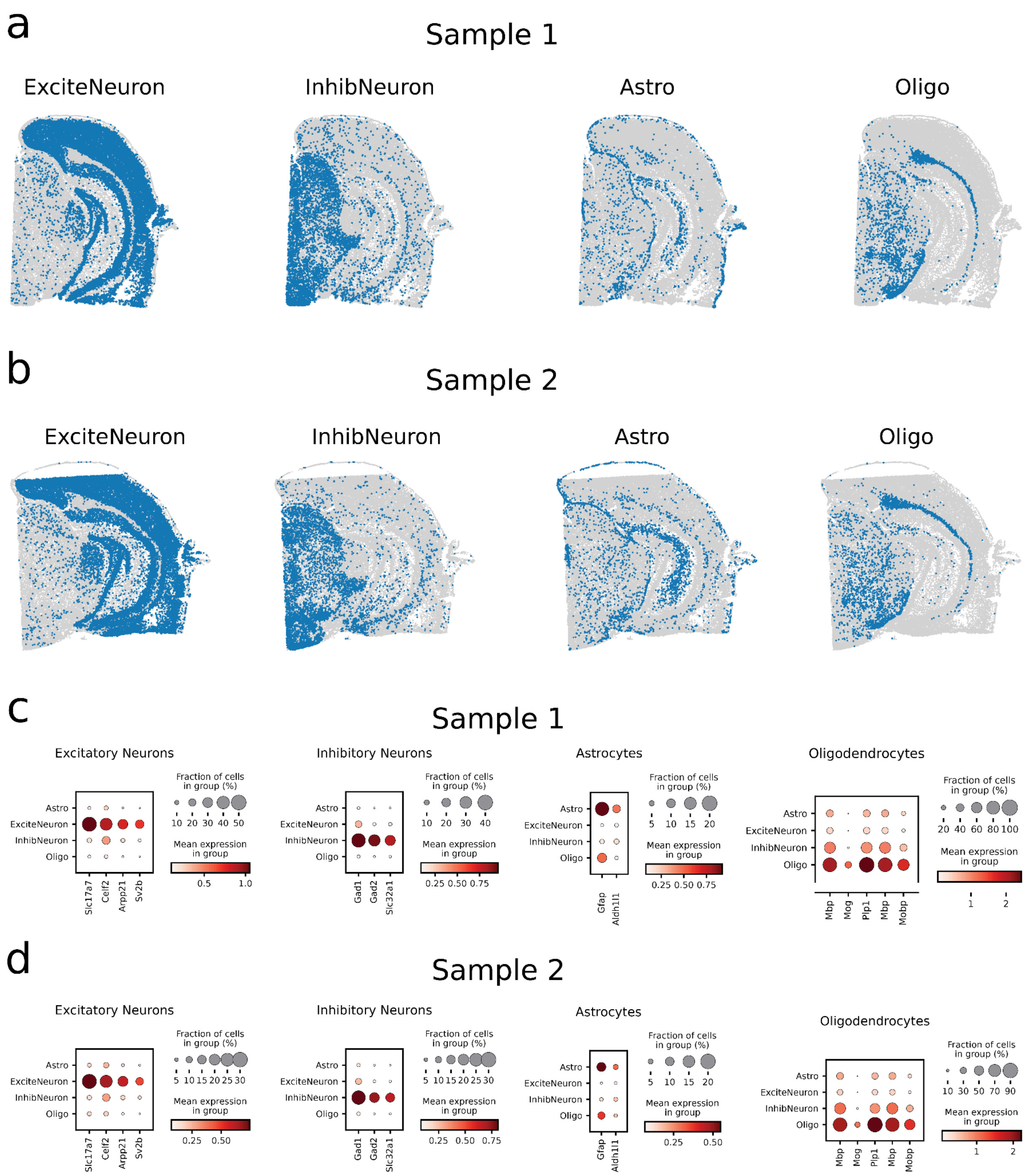

Figure S3. a-b) Cell-type distribution and **c-d)** marker gene expression of the four major cell types in **a,c)** Sample 1 and **b,d)** Sample 2. ExciteNeuron = Excitatory Neurons, InhibNeurons = Inhibitory Neurons, Astro = Astrocytes, Oligo = Oligodendrocytes

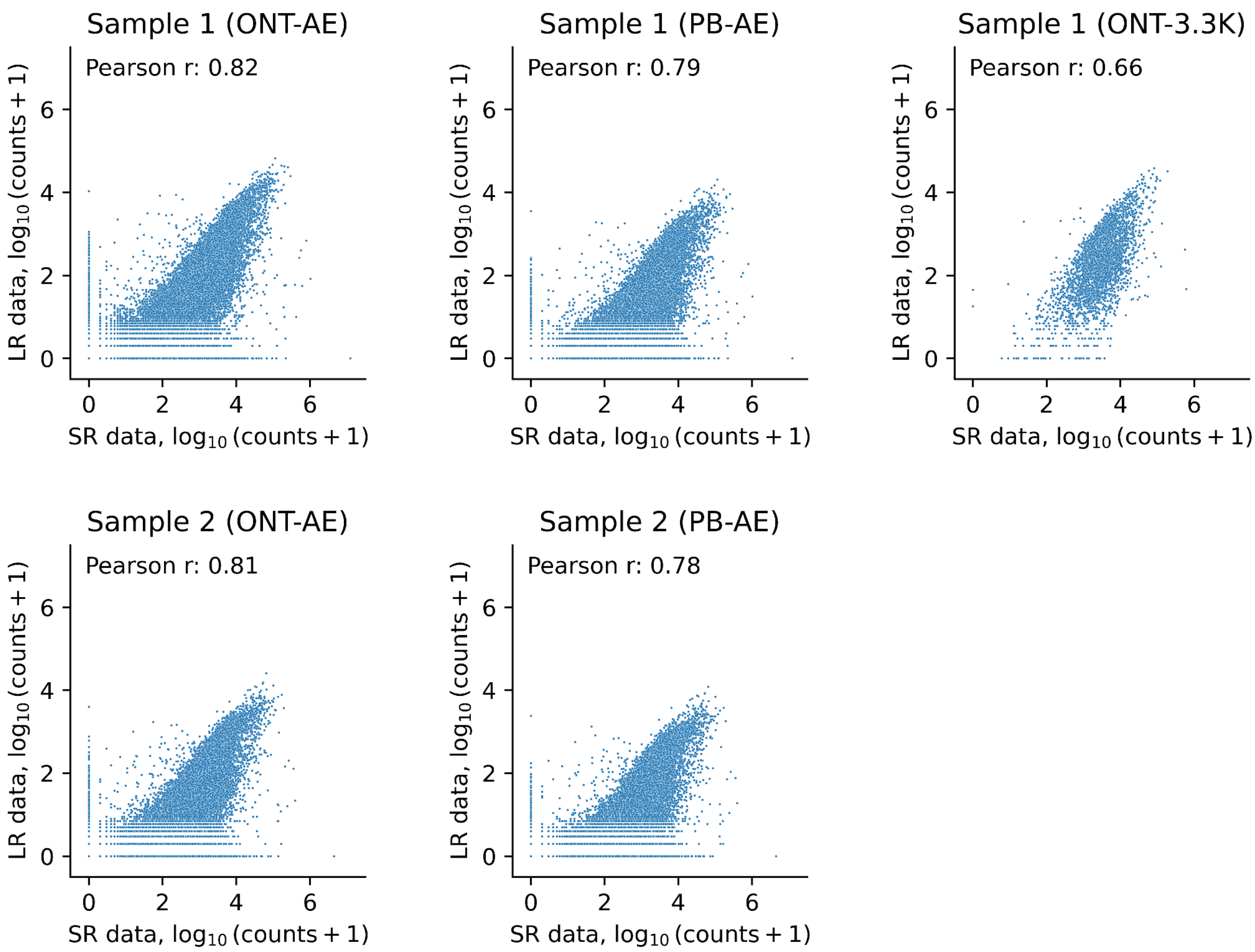

Figure S4. Correlation between gene expression as measured using the long-read and short-read sequencing. Counts are log10 transformed. Pearson correlation is indicated in the top left corner. Only the targeted genes are shown for Sample1 (3.3K). LR = long-read, SR = short-read, AE = allexome, PB = pacbio, 3.3K = 3.3K targeted dataset.

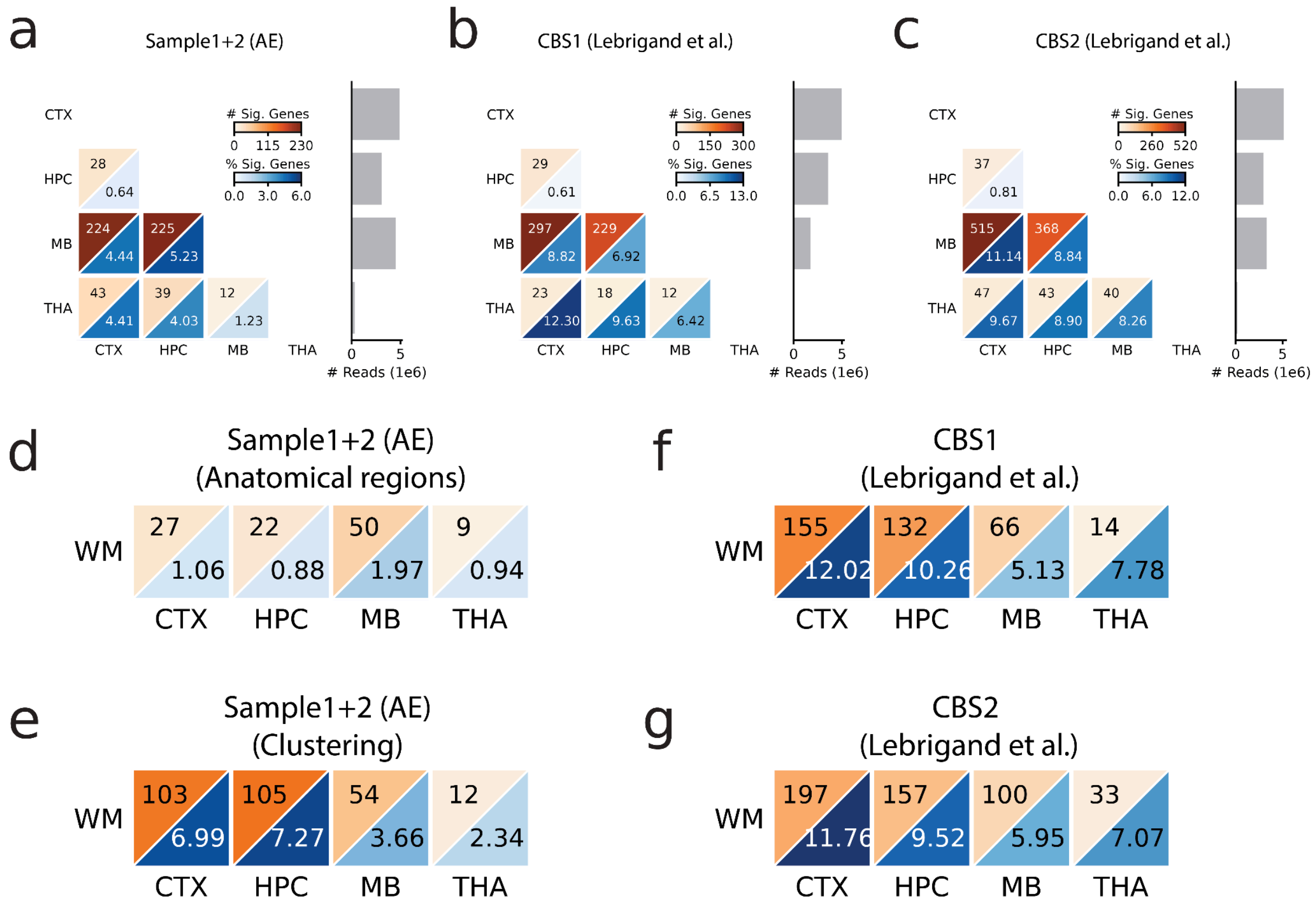

Figure S5. Number and percentage of genes changing isoform expression across predefined brain regions using all cells in **a)** combined Sample 1+2 (AE), **b)** Coronal Brain Section 1 (CBS1) from Lebrigand et al., and **c)** Coronal Brain Section 2 (CBS2) from Lebrigand et al. The barplot on the right shows the number of long reads used during the analysis per brain region. **d-g)** Number and percentage of genes changing isoform expression across predefined brain regions when comparing white matter to other brain regions in **d-e)** combined Sample 1+2 (AE) using regions defined based on **d)** anatomical locations and **e)** clustering the cells, **f)** Coronal Brain Section 1 (CBS1) from Lebrigand et al., and **g)** Coronal Brain Section 2 (CBS2) from Lebrigand et al..

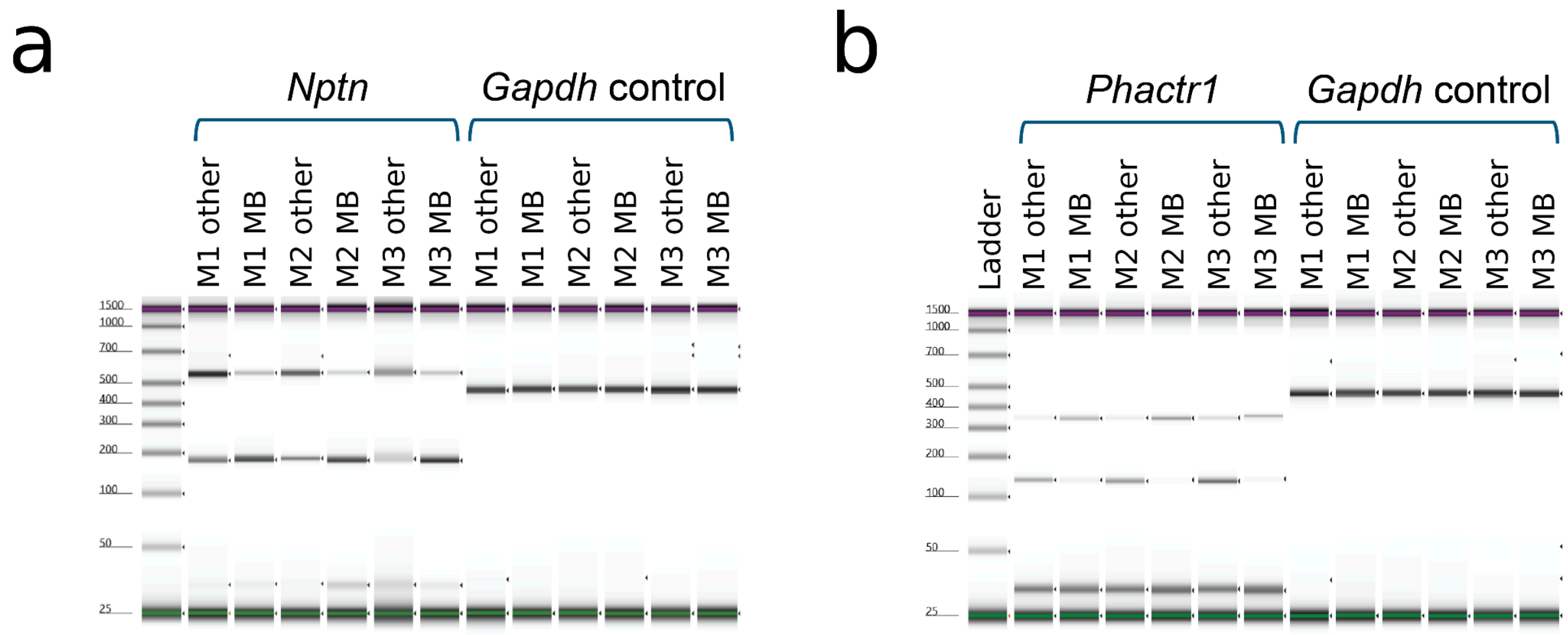

Figure S6. a-b) Tapestation gel of PCR amplified products using exon-of-interest-spanning **a)** *Nptn* and **b)** *Phatcr1* specific primers or *Gapdh* specific primers. The ladder on the left indicates molecular weight in base pairs. Mx = Mouse sample, MB = isolated bulk midbrain, other = bulk brain with midbrain removed.

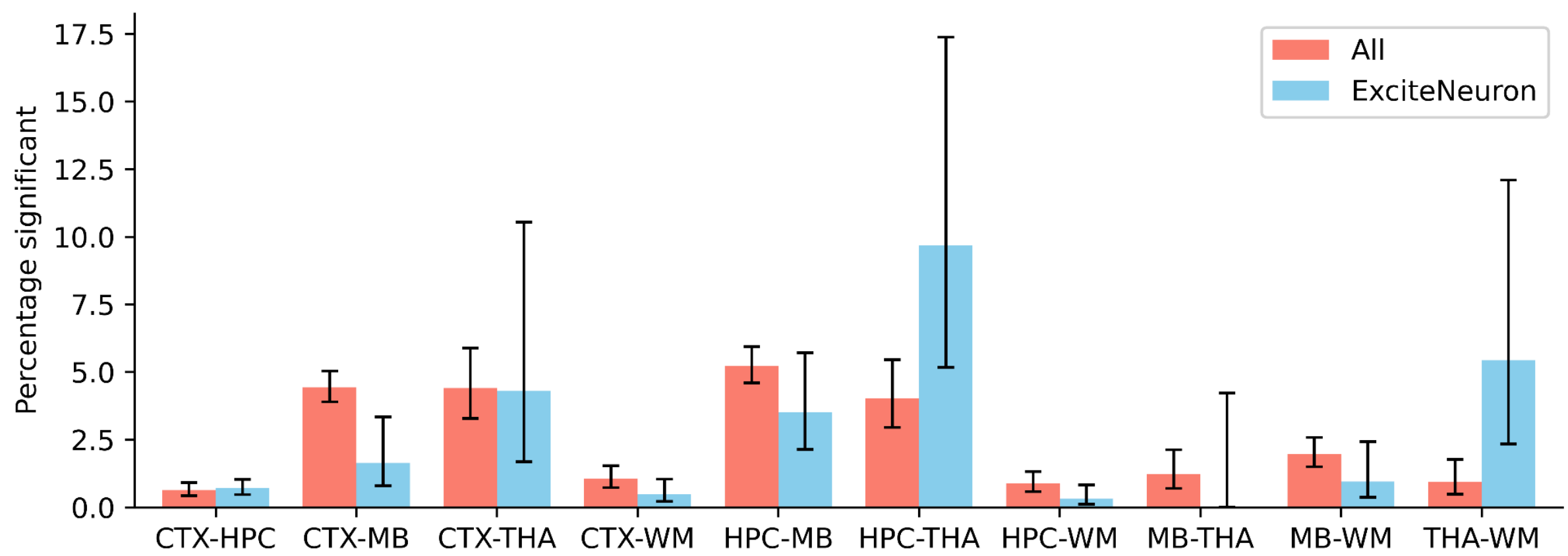

Figure S7. Percentage of significant genes for each comparison using all cells and only excitatory neurons. Each bar represents a single proportion; error bars indicate 95% confidence intervals. CTX = cortex, HPC = hippocampus, MB = midbrain, THA = thalamus, WM = white matter,

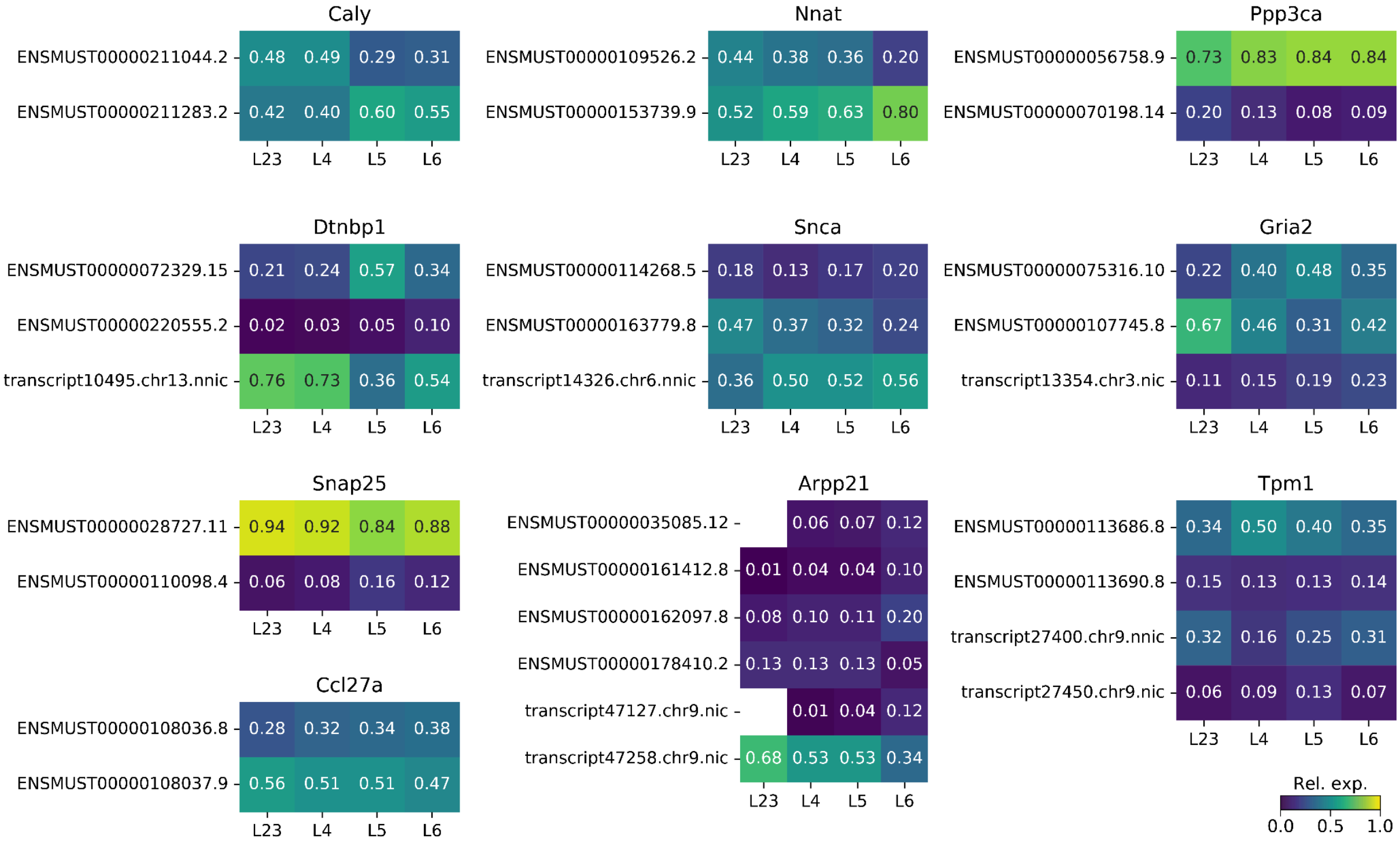

Figure S8. For all genes showing layer-specific isoform abundance in excitatory neurons, a heatmap is showing the relative expression of each isoform in the different layers. Only isoforms with relative expression ≥ 0.1 in at least one layer are plotted.

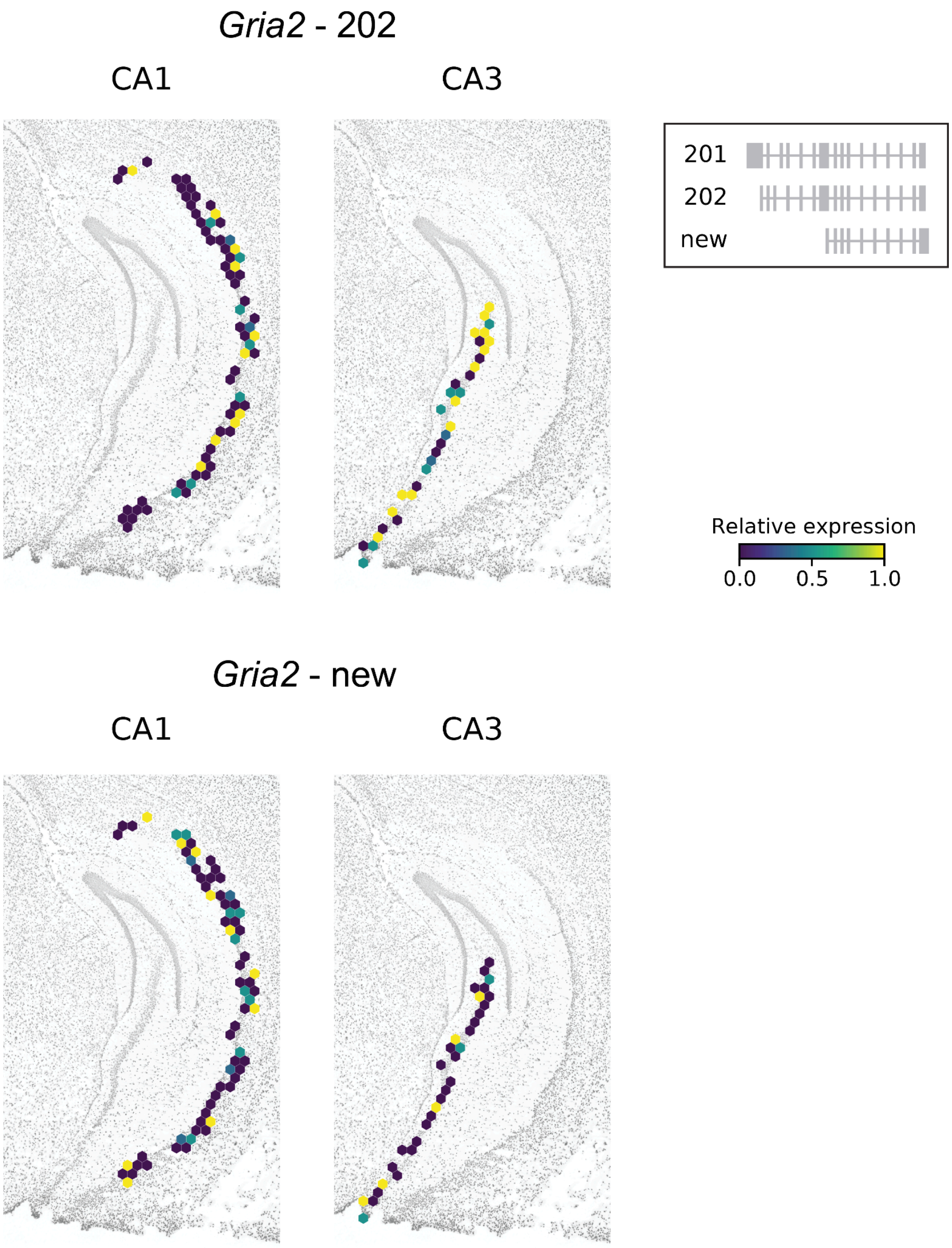

Figure S9. Relative expression of *Gria2* isoform 202, and a new transcript in excitatory neurons in different regions of the hippocampus of Sample 1. Every hexagon shows the mean relative expression of the underlying cells (Methods). *Gria2* shows differential isoform changes between CA1 and CA3.

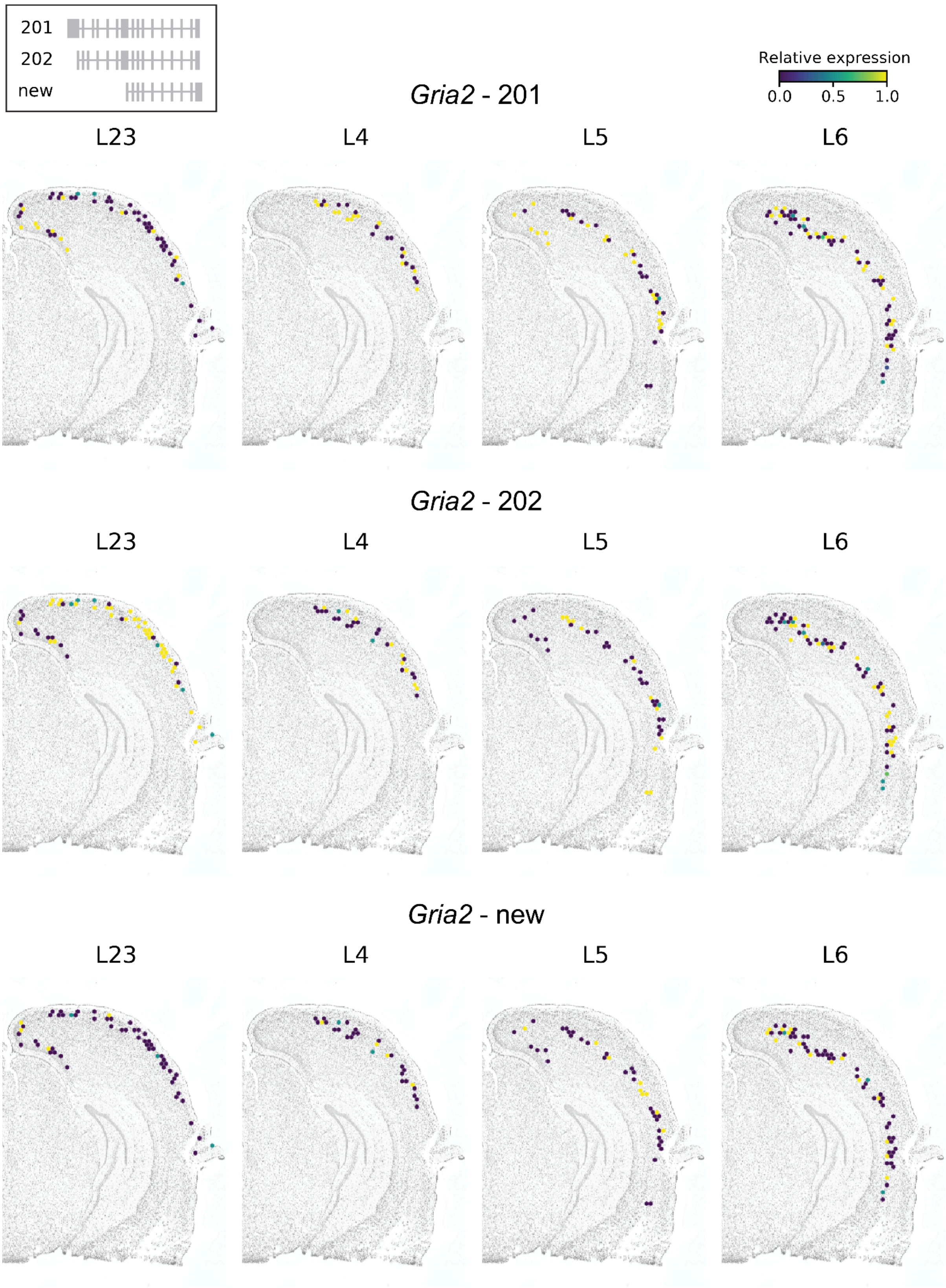

Figure S10. Relative expression of *Gria2* isoform 201, 202, and a new transcript in excitatory neurons in different cortical layers of Sample 1. Every hexagon shows the mean relative expression of the underlying cells (Methods). *Gria2* shows differential isoform changes between L23 and L5.

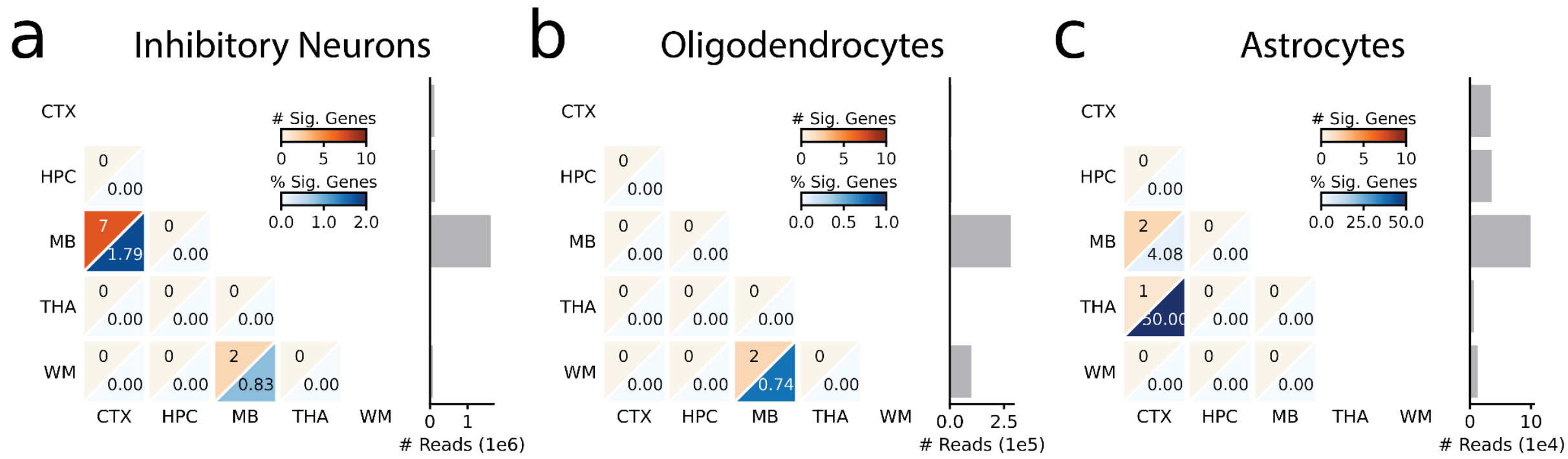

Figure S11. Number and percentage of genes changing isoform expression in **a)** inhibitory neurons, **b)** oligodendrocytes, and **c)** astrocytes across brain regions. Barplot on the right shows the number of long reads used during the analysis per brain region. CTX = cortex, HPC = hippocampus, MB = midbrain, THA = thalamus, WM = white matter.

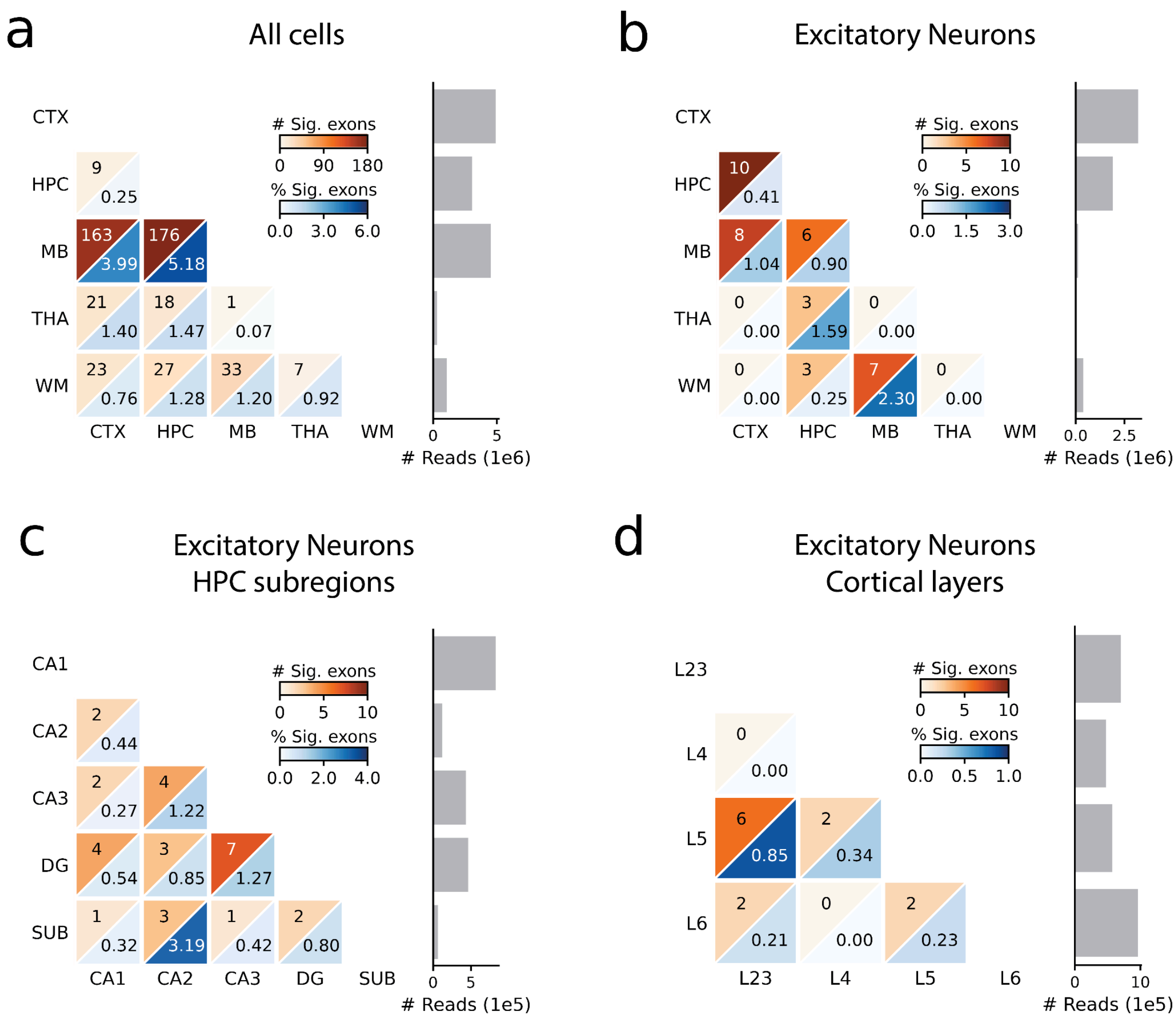

Figure S12. a) Number and percentage of exons changing inclusion across predefined brain regions using all cells in combined Sample 1+2 (AE). Barplot on the right shows the number of long reads used during the analysis per brain region. **b-d)** Number and percentage of exons changing inclusion in excitatory neurons across (sub)regions. CTX = cortex, HPC = hippocampus, MB = midbrain, THA = thalamus, WM = white matter, DG = Dentate Gyrus, SUB = Subiculum

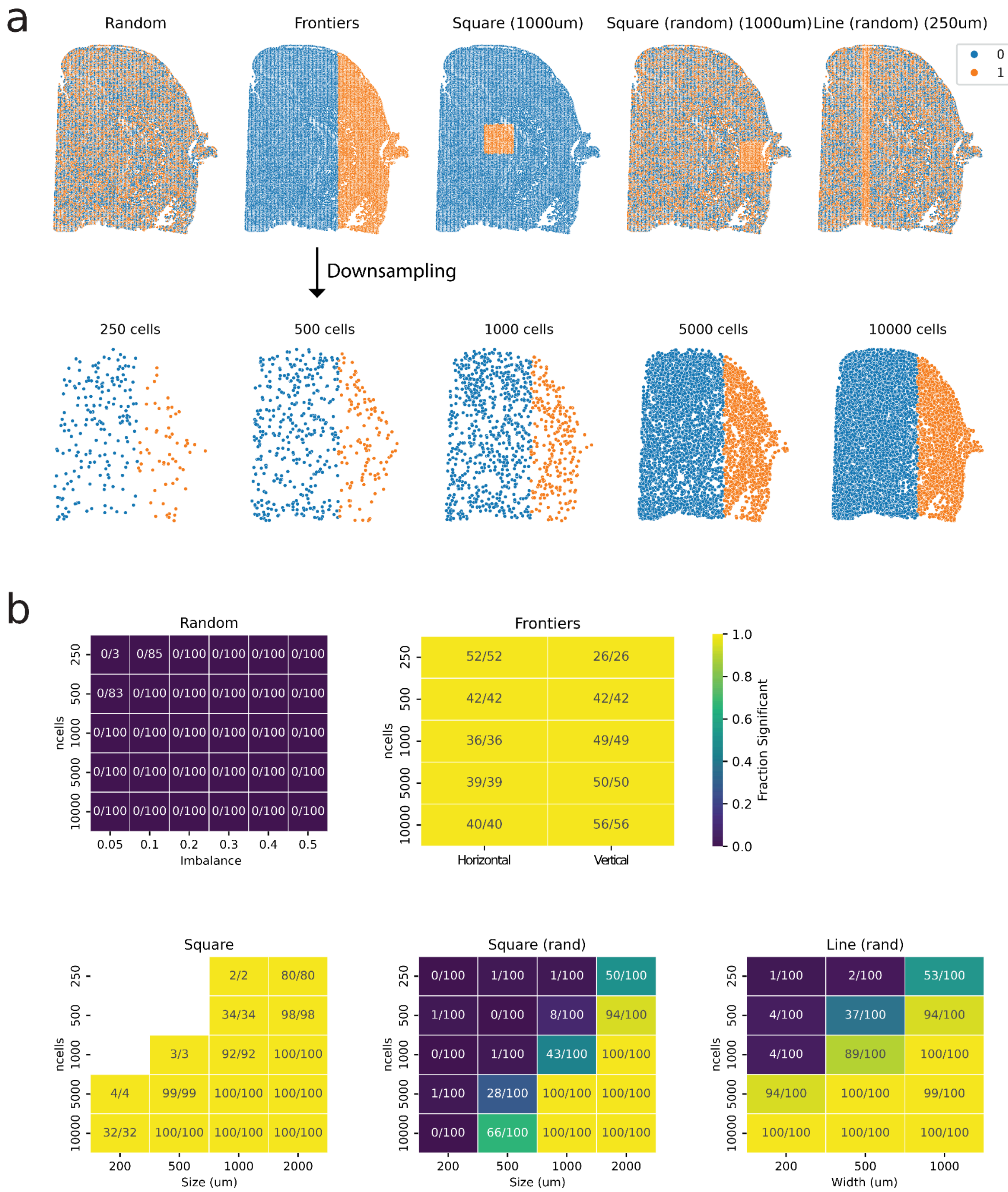

Figure S13. a) Example simulation for every category, including a downsampling example for the frontiers simulation. **b)** Heatmaps indicating the number of significant simulations per category.

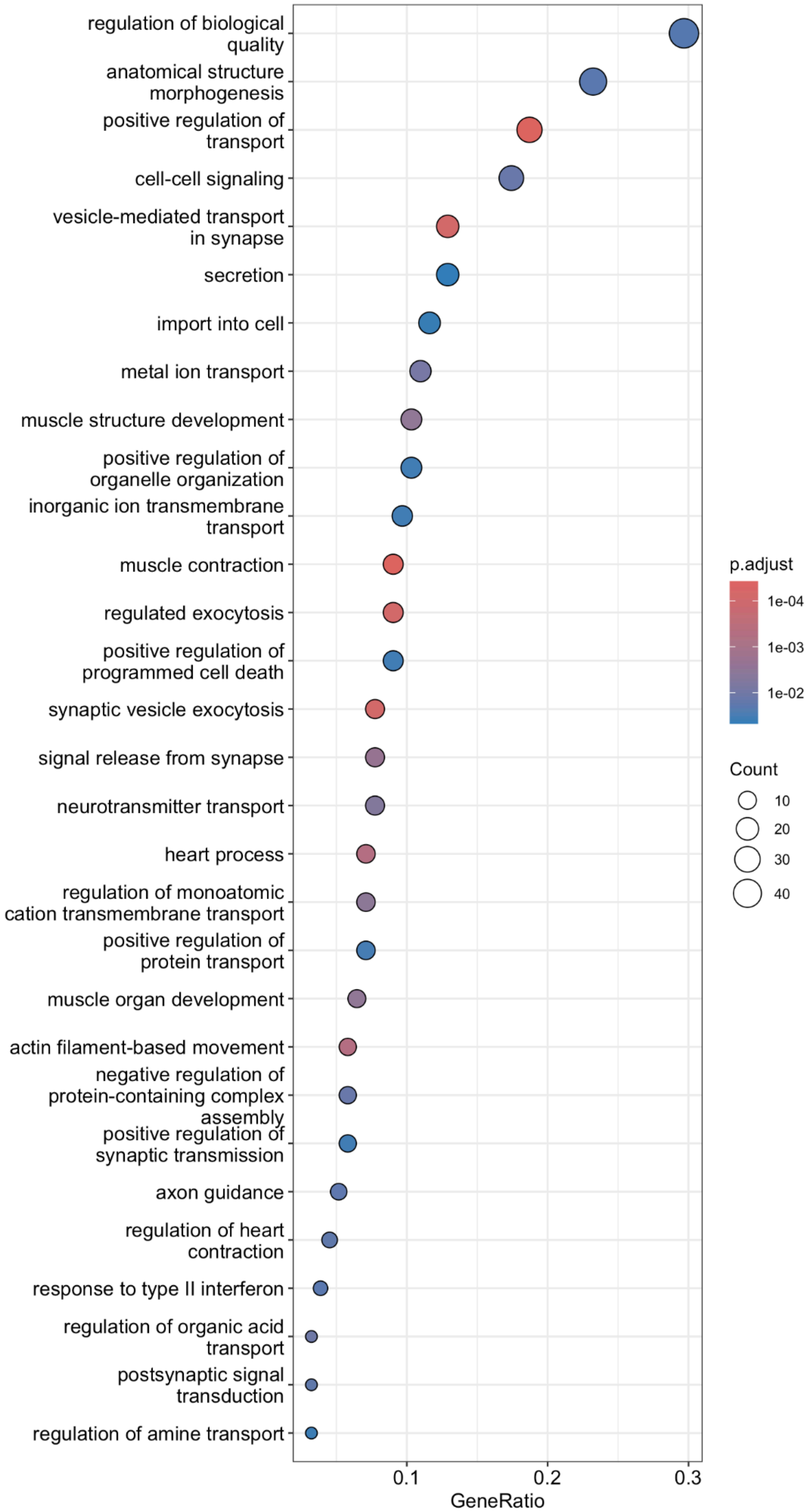

Figure S14. Significantly enriched biological processes (BP) in SVIs in the combined Sample 1+2 (AE) identified using GO enrichment analysis.

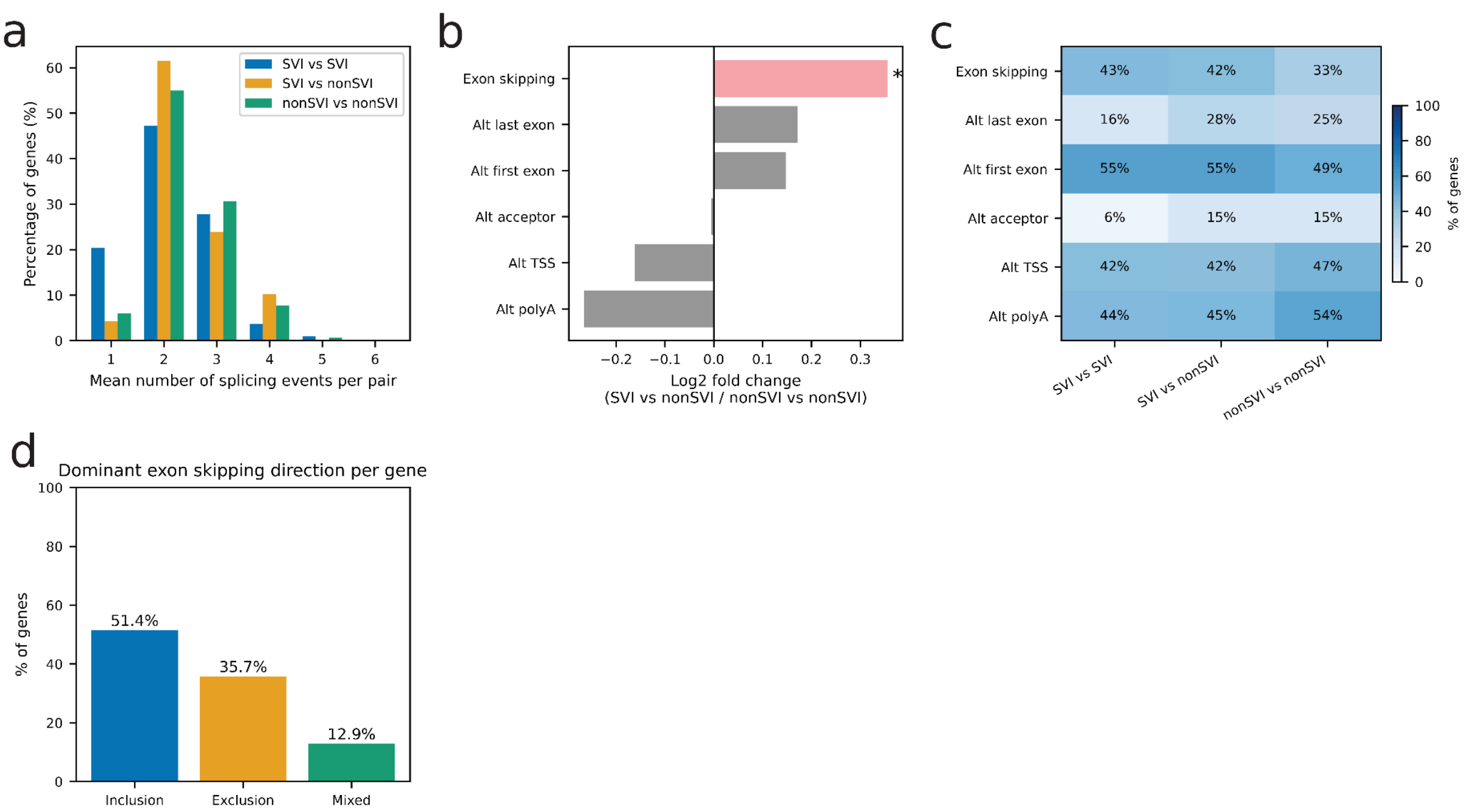

Figure S15. a) Distribution of the mean number of co-occurring splicing events per isoform pair, grouped by comparison type (SVI vs SVI, SVI vs nonSVI, nonSVI vs nonSVI). Values are averaged per gene to avoid bias from genes with large isoform repertoires. **b)** Log2 fold change of splicing event frequencies in SVI vs nonSVI pairs relative to nonSVI vs nonSVI pairs. The highlighted bar (exon skipping) indicates a statistically significant enrichment (Mann-Whitney U test, FDR-adjusted p = 0.038). Event categories with a mean frequency below 10% across all comparison types were excluded from statistical testing. **c)** Heatmap showing the percentage of genes in which each splicing event type was detected, stratified by comparison type. Values represent gene-level means averaged across all pairwise comparisons within each comparison type. **d)** Exon skipping direction per gene (for genes with at least one SVI vs nonSVI pair showing an exon skipping event). For each gene, the direction was defined as the direction (inclusion or exclusion in the SVI) observed in the majority of pairs. Genes with equal numbers of inclusion and exclusion pairs were classified as mixed.

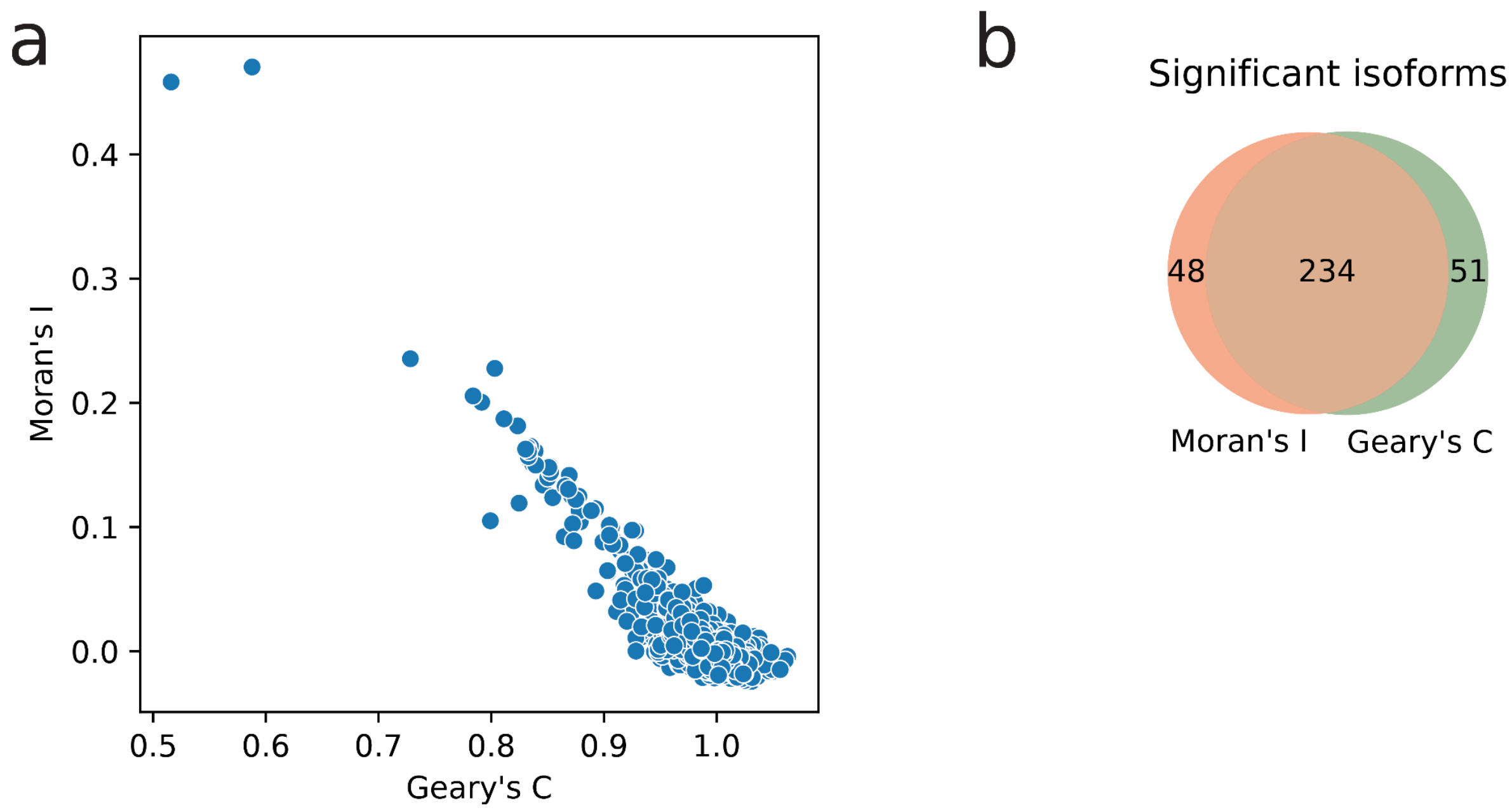

Figure S16. a) Geary's C vs. Moran's I score for the tested isoforms in the combined Sample 1+2 (AE) data. **b)** Venn diagram showing the overlap of significant isoforms when using Moran's I or Geary's C.

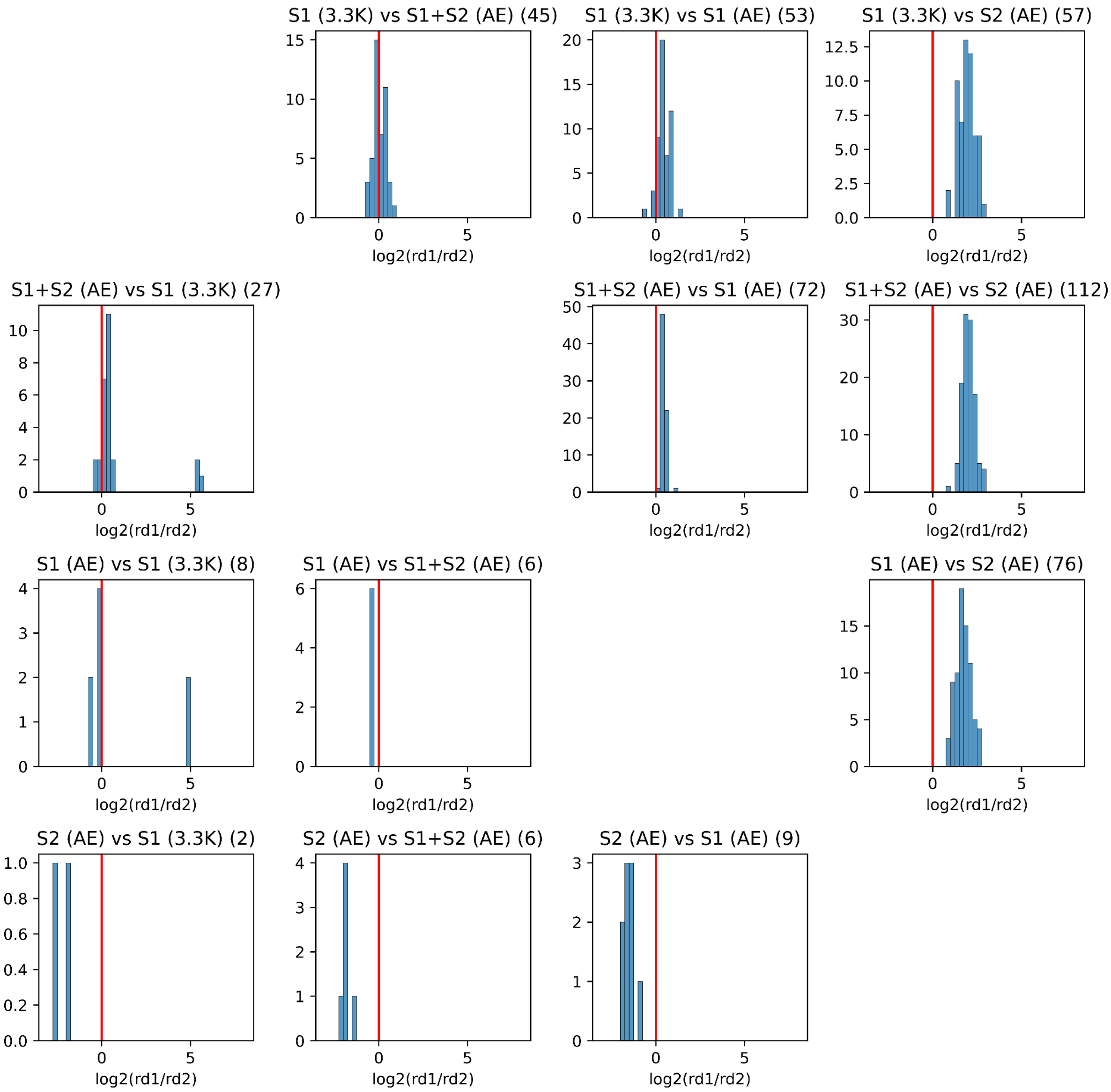

Figure S17. For every pairwise combination of datasets (e.g. S1 (3.3K) vs S1+S2 (AE)), we count the number of isoforms significant in the first dataset and not significant in the other. The comparison and number of isoforms are mentioned in the title. For these isoforms, we compare the read depth in the first dataset (rd1) compared to the second dataset (rd2). A value larger than zero indicates that the discordance is potentially explained by the difference in read depth between the samples.

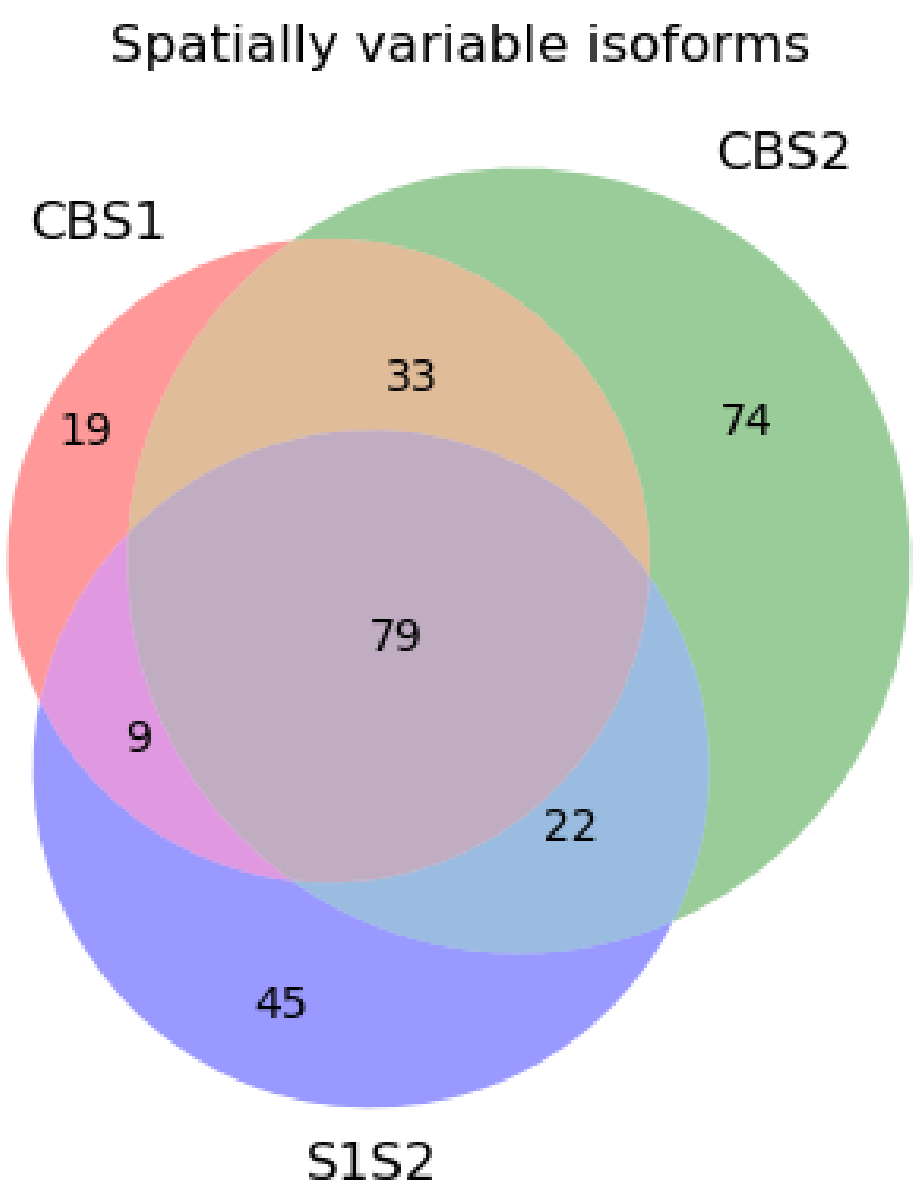

Figure S18. Overlap between the detected SVIs in our combined Sample 1+2 (AE) data, CBS1 and CBS2 from Lebrigand et al.

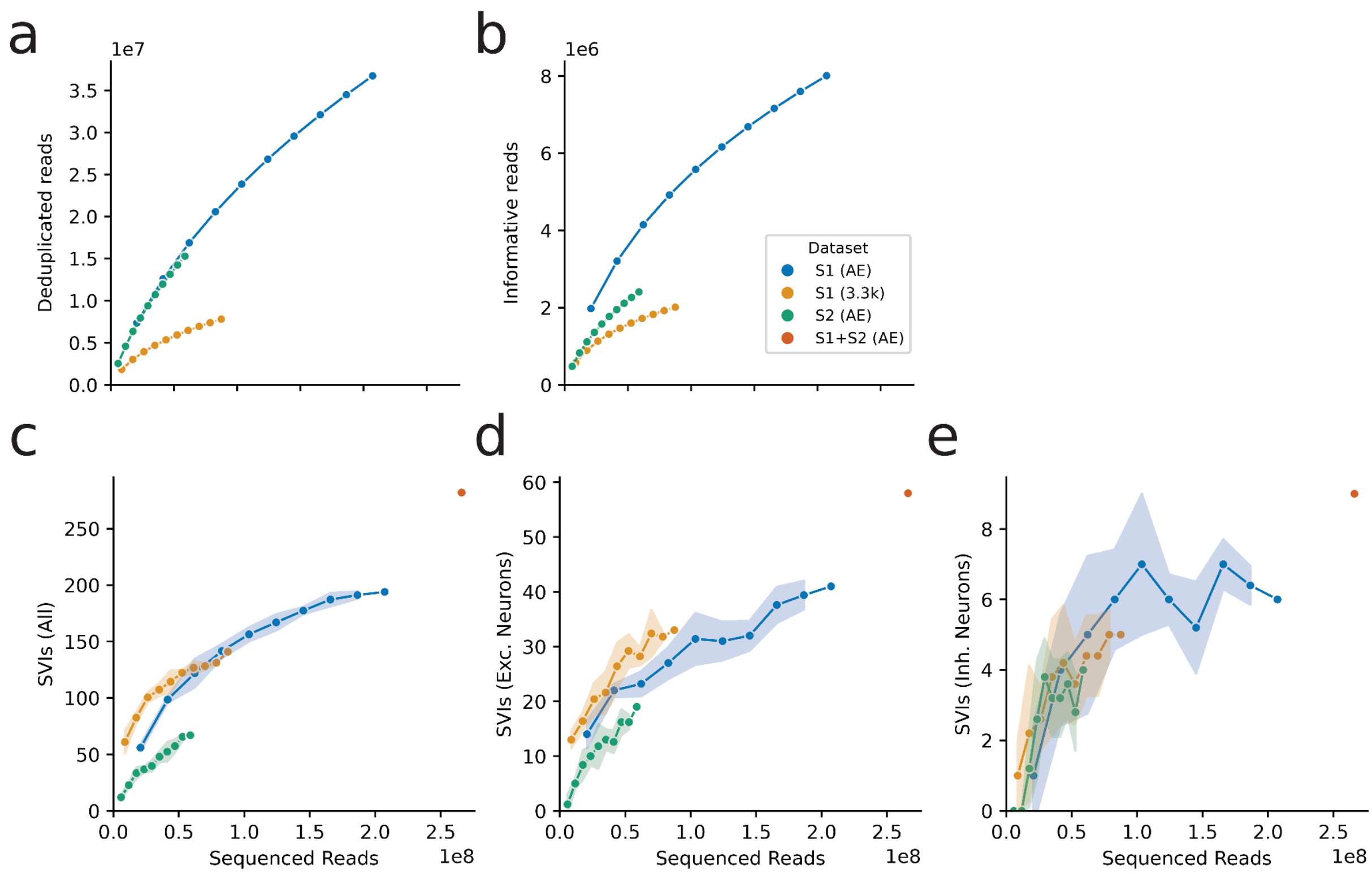

Figure S19. Saturation analysis for the Spl-ISO-Seq2 data. The plots show the number of downsampled sequenced reads vs. **a)** deduplicated reads, **b)** informative reads (spliced reads with an assigned isoform that overlap a segmented cell in the correct hemisphere), **c-e)** spatially variable isoforms (SVIs) detected in **c)** all cells, **d)** excitatory neurons, and **e)** inhibitory neurons. Lines show the mean across 10 downsampling experiments; shaded regions indicate ±1 standard deviation.

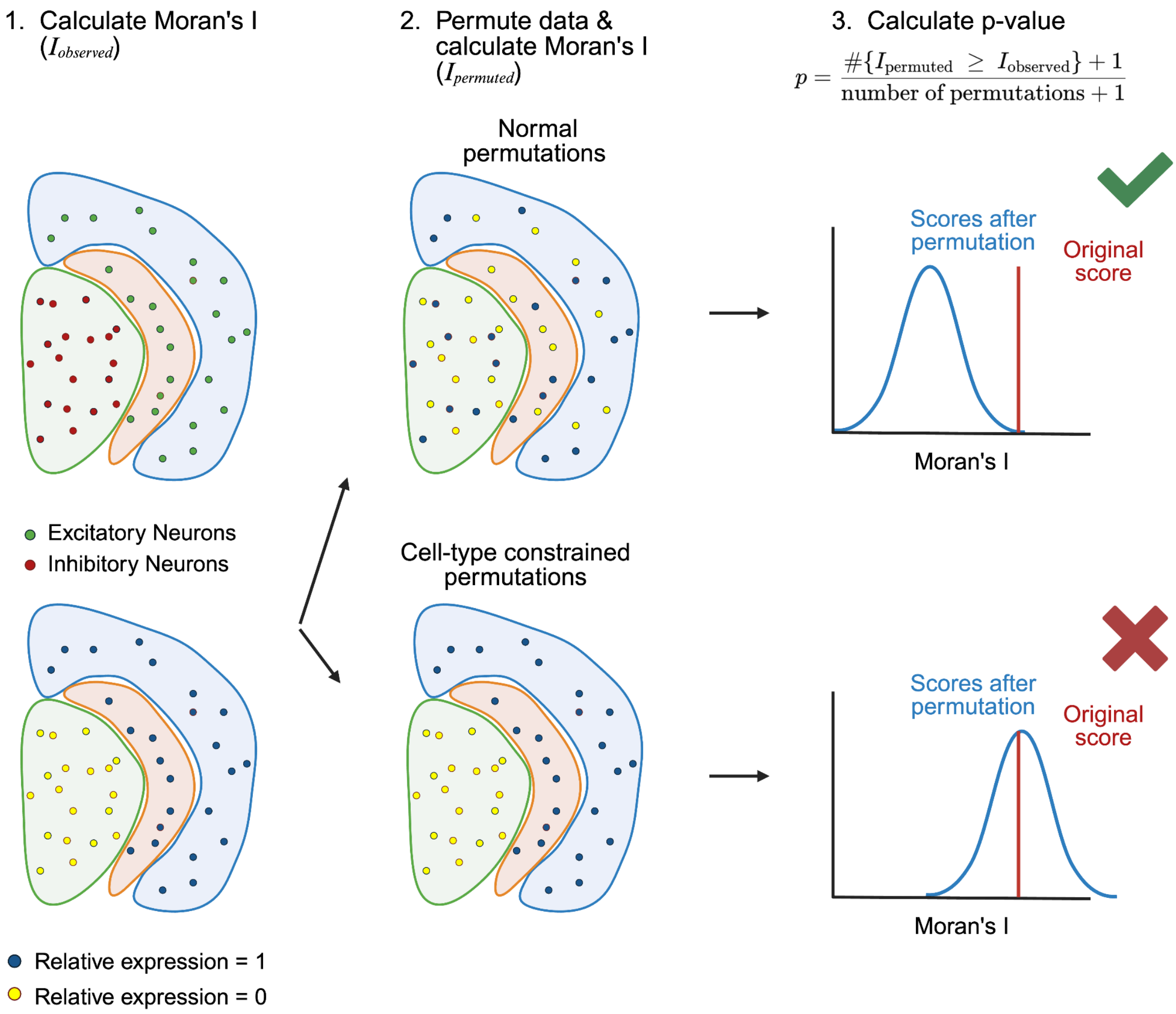

Figure S20. Schematic example to explain the difference between normal and cell-type constrained permutations. In the example, the spatial isoform pattern (high relative expression in the blue and orange region vs. low relative expression in the green region) is caused by a different cell-type composition (the blue and orange region only contains excitatory neurons, while the green region contains inhibitory neurons). During our normal permutation tests, the relative expression values of all cells are shuffled, causing the isoform to be significant. However, during the cell-type constrained permutations, only values within a cell type are shuffled and as such this example isoform is not significant anymore. Figure created in BioRender. Jarroux, J. (2026) https://BioRender.com/gg7aevk

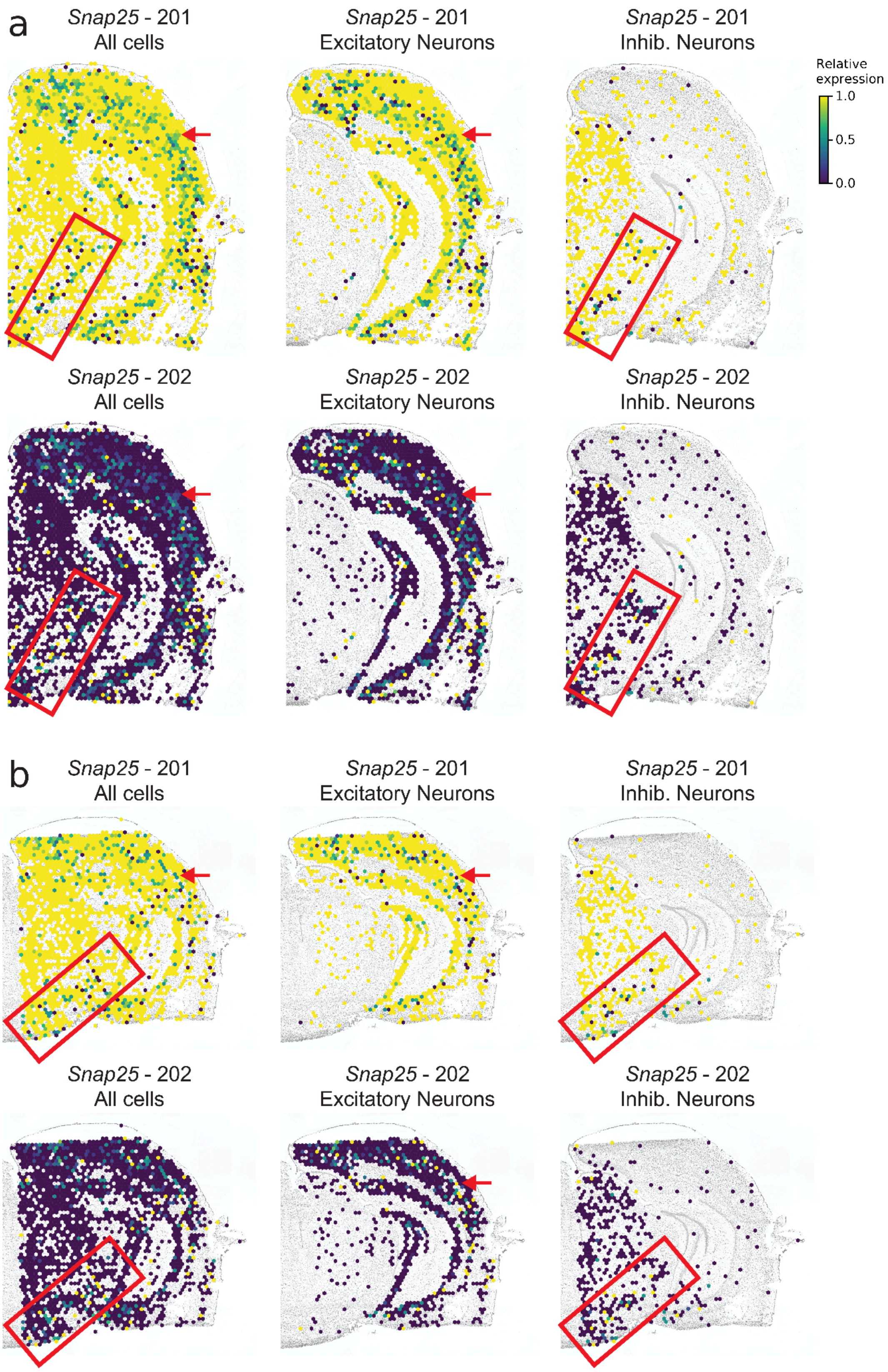

Figure S21. Spatial distribution of *Snap25* isoforms in all cells, excitatory and inhibitory neurons in **a)** Sample 1 (AE) and **b)** Sample 2 (AE). Every hexagon shows the mean relative expression of the underlying cells (Methods). Since 201 and 202 are the only isoforms for *Snap25* in our data, the relative expression values add up to 1. The red boxes and arrows indicate decreased expression of isoform 201 and thus increased expression of 202.

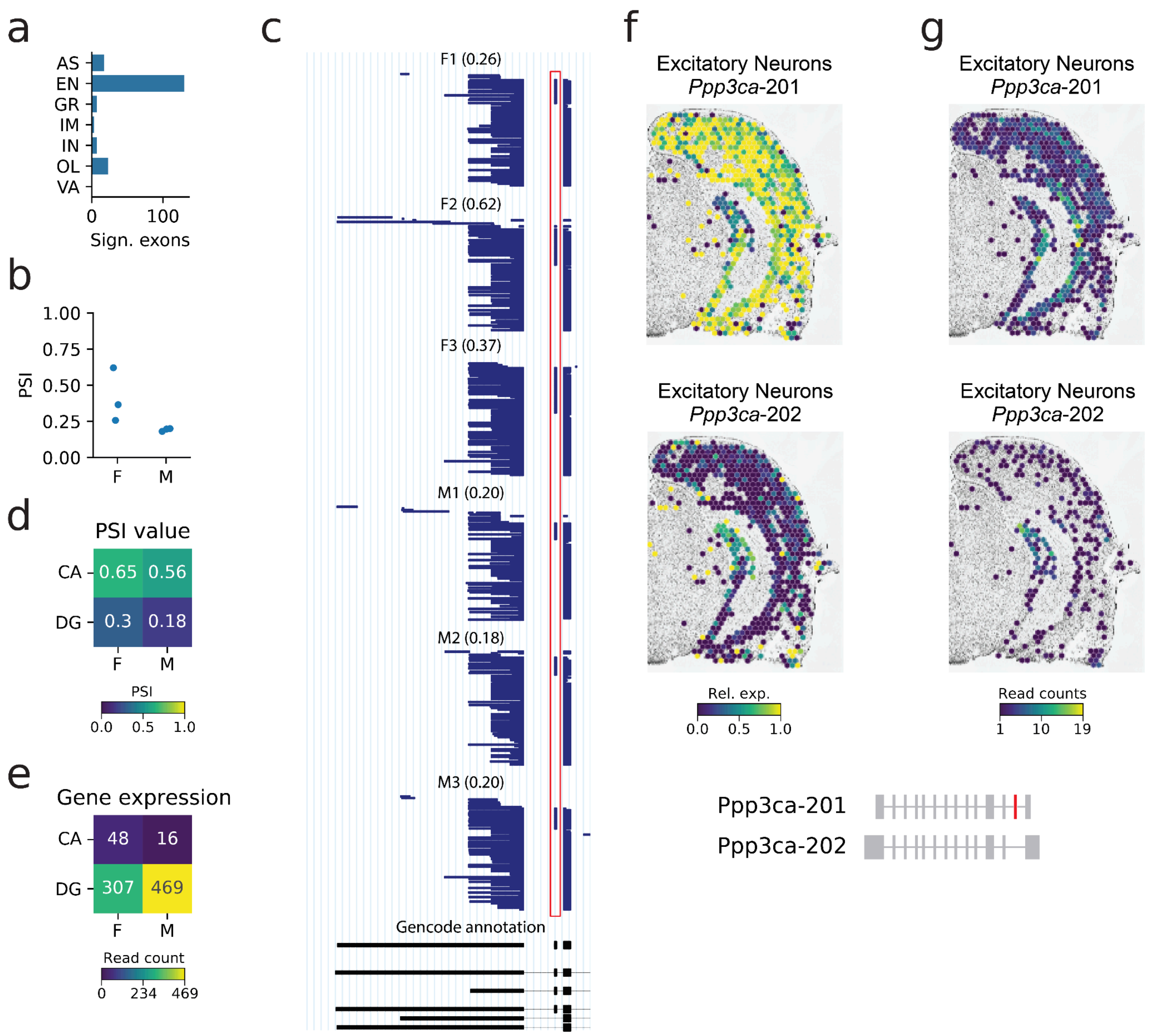

Figure S22. a) Number of significant sex-specific exons per cell type. **b)** PSI value for exon chr4_101029166_101029195_- in *PPP3CA* for each individual in excitatory neurons. **c)** Single-cell long-reads for *PPP3CA* measured in excitatory neurons per individual. Each line in the top six tracks represents a single cDNA molecule. The exon, chr4_101029166_101029195_-, in the red box is sex-specific. The bottom black track shows the Gencode annotation. The PSI value for each individual is indicated between brackets. **d)** PSI value and **e)** gene expression (number of spanning long reads) for exon chr4_101029166_101029195_- in *PPP3CA* in excitatory neurons in the Dentate Gyrus (DG) and Cornu Ammonis (CA) for females (F) and males (M). **f)** Relative and **g)** absolute expression of *Ppp3ca*-201 and *Ppp3ca*-202 in excitatory neurons in Sample 1 (AE). In **f)** every hexagon shows the mean relative expression of the underlying cells, while in **g)** every hexagon shows the sum of the absolute read count of the underlying cells. The ssDNA staining is shown in the background. The schematic diagram at the bottom shows the gene structure of *Ppp3ca*-201 and *Ppp3ca*-202, the two most common isoforms in the data. The exon highlighted in red is the main difference between the two isoforms. This is the same exon that shows sex-specific inclusion differences, noting that PPP3CA is encoded on the positive strand in human and on the negative strand in mouse. AS = Astrocytes, EN = Excitatory Neurons, GR = Granule cells, IM = Immune cells, IN = Inhibitory Neurons, OL = Oligodendrocytes, VA = Vascular cells.

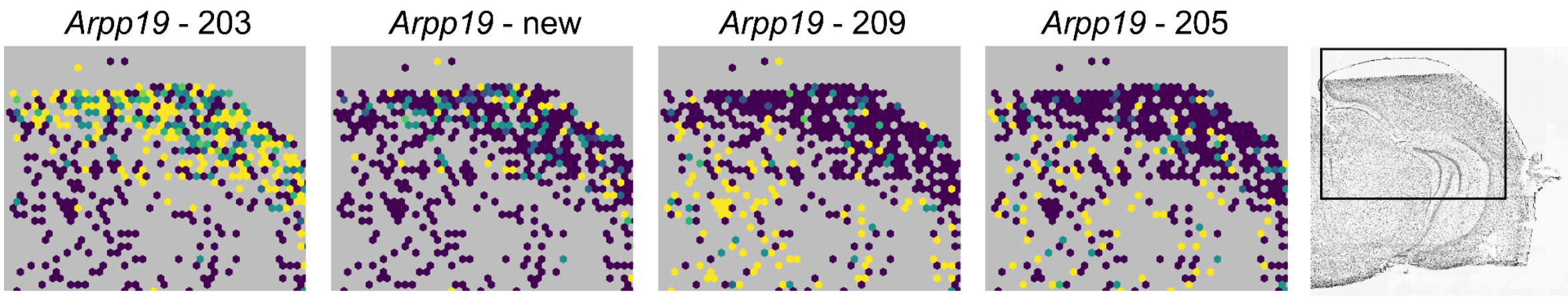

Figure S23. Spatial distribution of the four significant isoforms of *Arpp19* in Sample 2 (AE). Every hexagon shows the mean relative expression of the underlying cells (Methods).

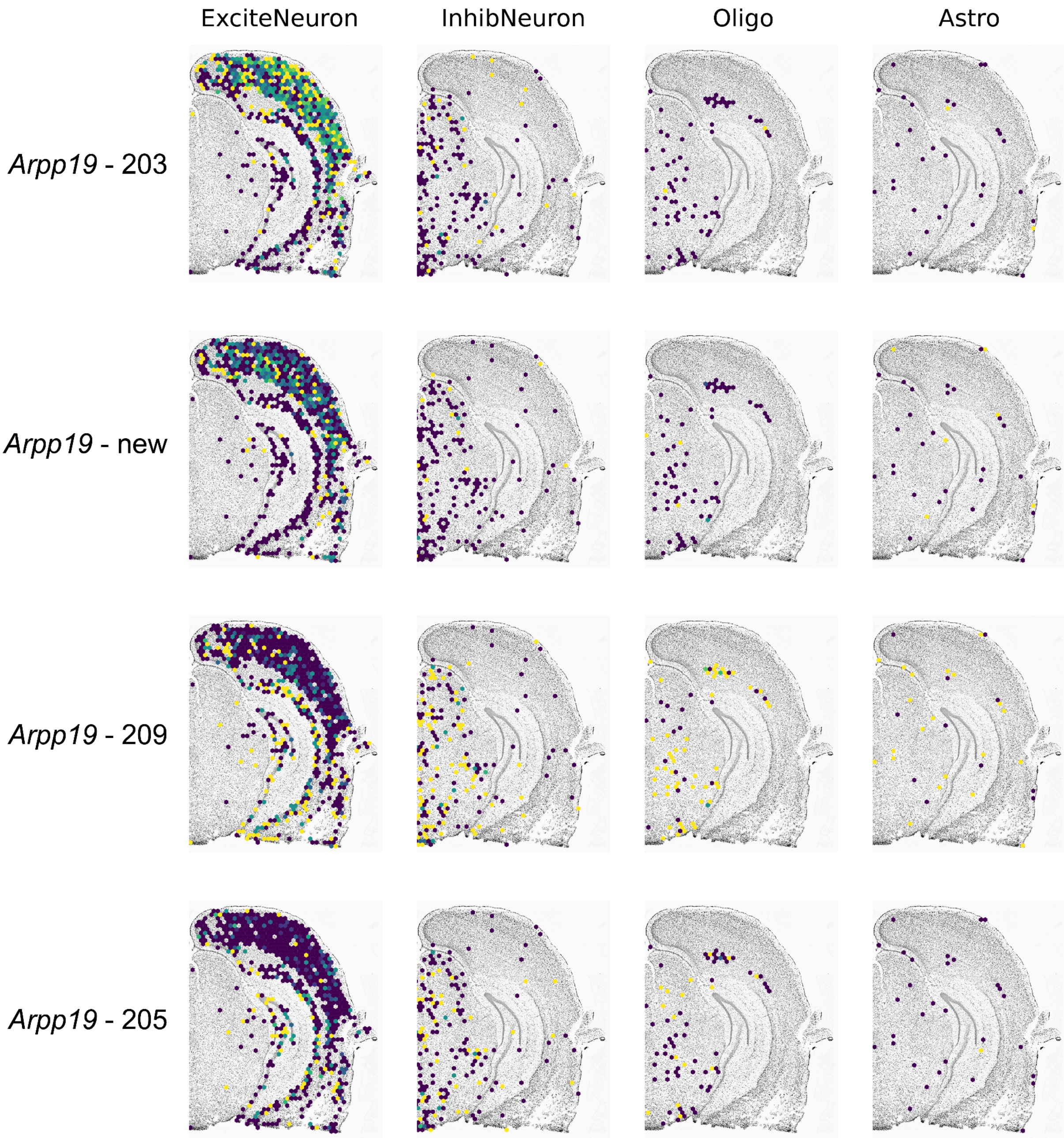

Figure S24. Spatial distribution of *Arpp19* isoforms per cell type in Sample 1 (AE). Every hexagon shows the mean relative expression of the underlying cells (Methods).

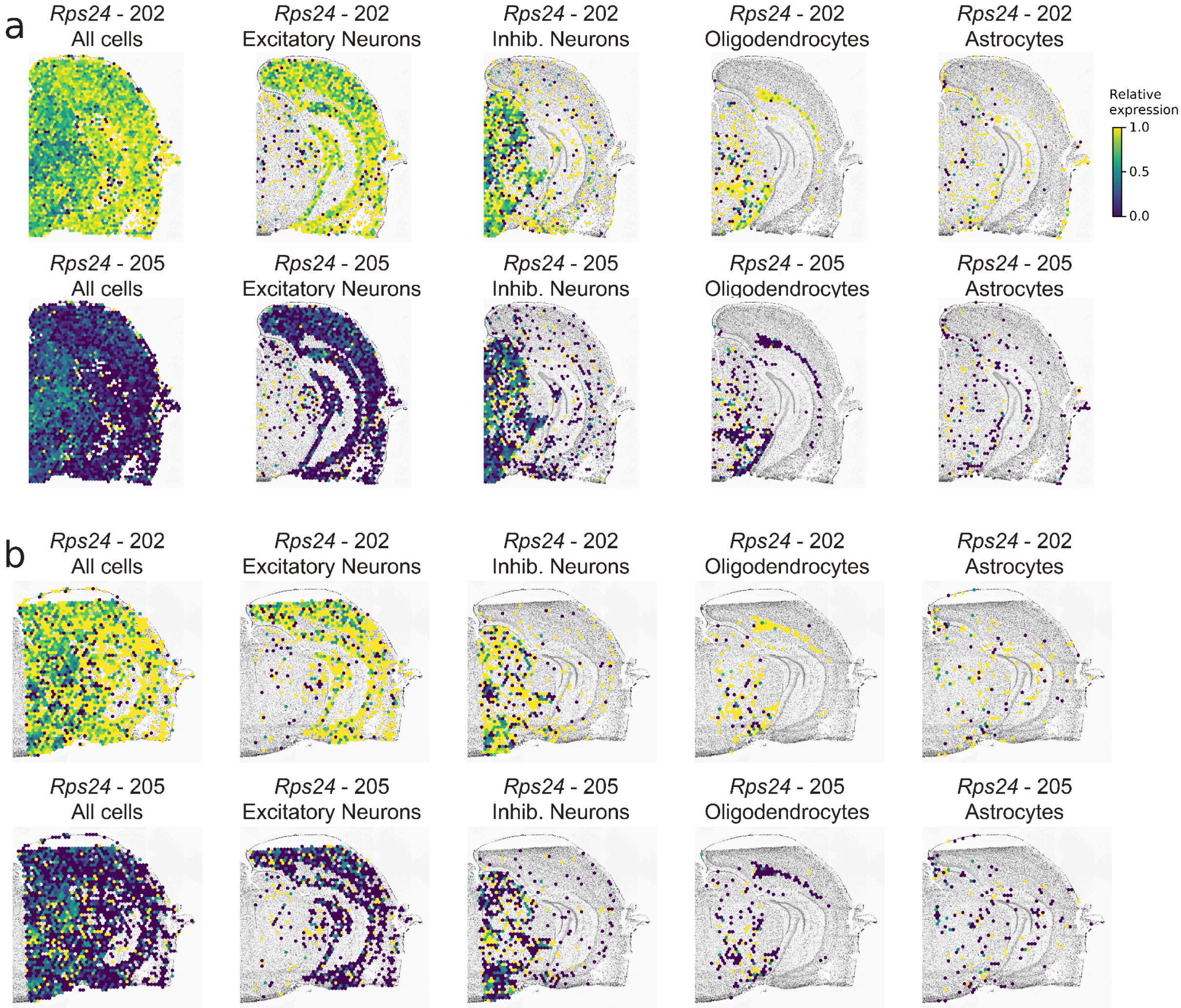

Figure S25. Spatial distribution of two isoforms of *Rps24* in all cells and per cell type in **a)** Sample 1 (AE) and **b)** Sample 2 (AE). Every hexagon shows the mean relative expression of the underlying cells (Methods).

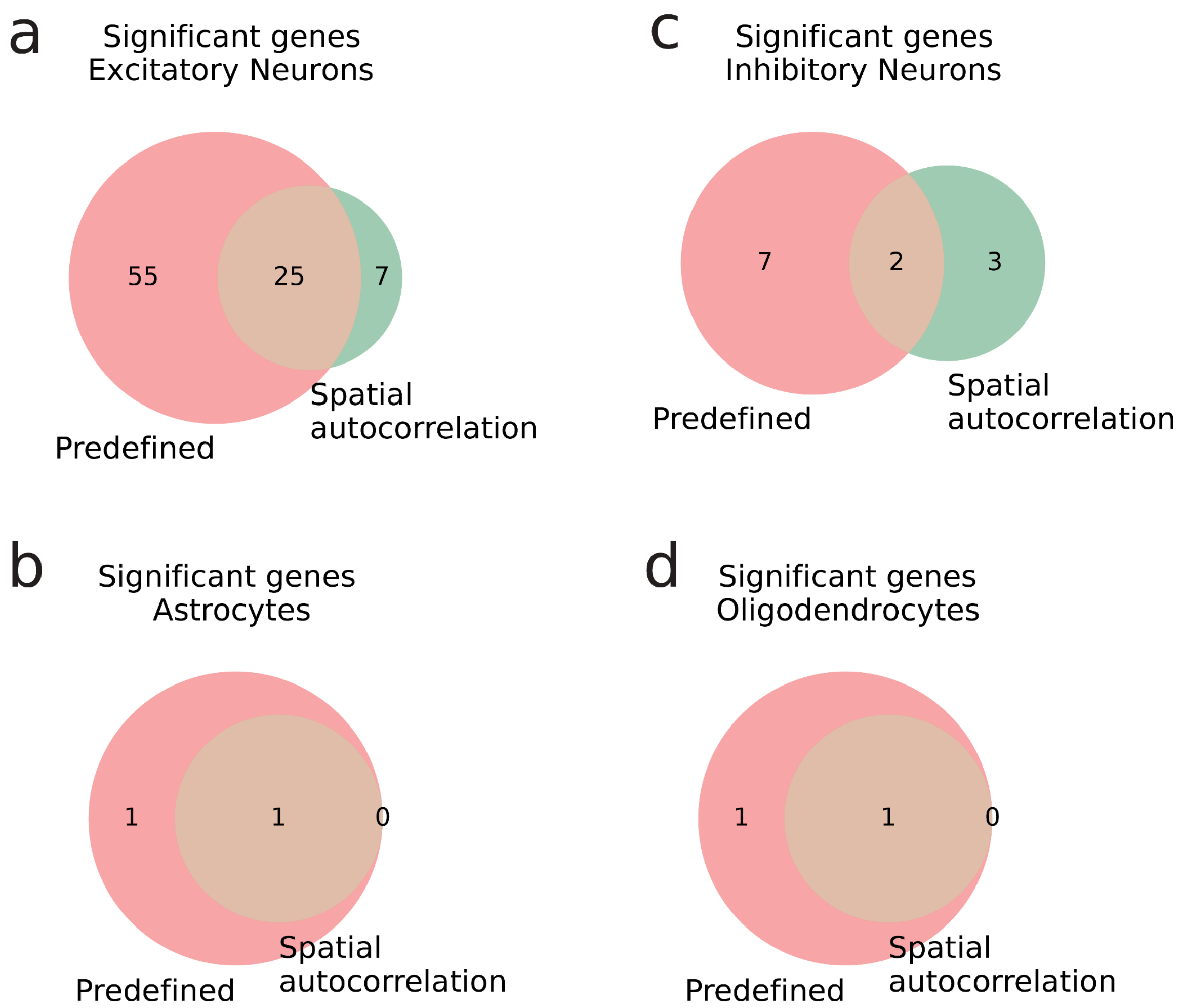

Figure S26. Overlap between the significant genes found using both methods for **a)** excitatory neurons, **b)** inhibitory neurons, **c)** astrocytes, and **d)** oligodendrocytes in the Sample 1 (AE) + Sample 2 (AE) data.

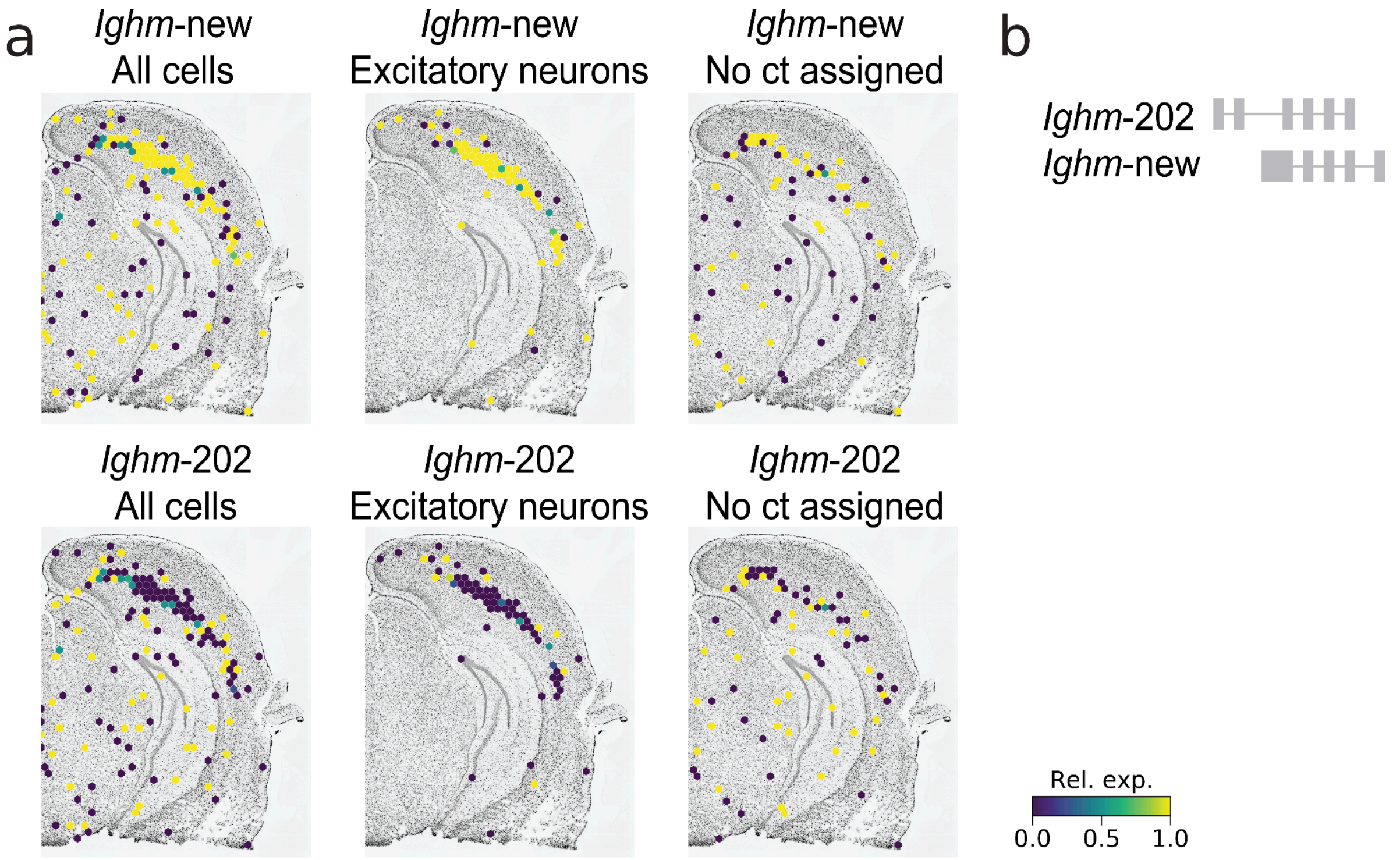

Figure S27. a) Spatial distribution of two *Ighm* isoforms in all cells, excitatory neurons, and cells that couldn't be confidently assigned a cell-type label. Every hexagon shows the mean relative expression of the underlying cells (Methods). **b)** Schematic gene diagram of the two isoforms.

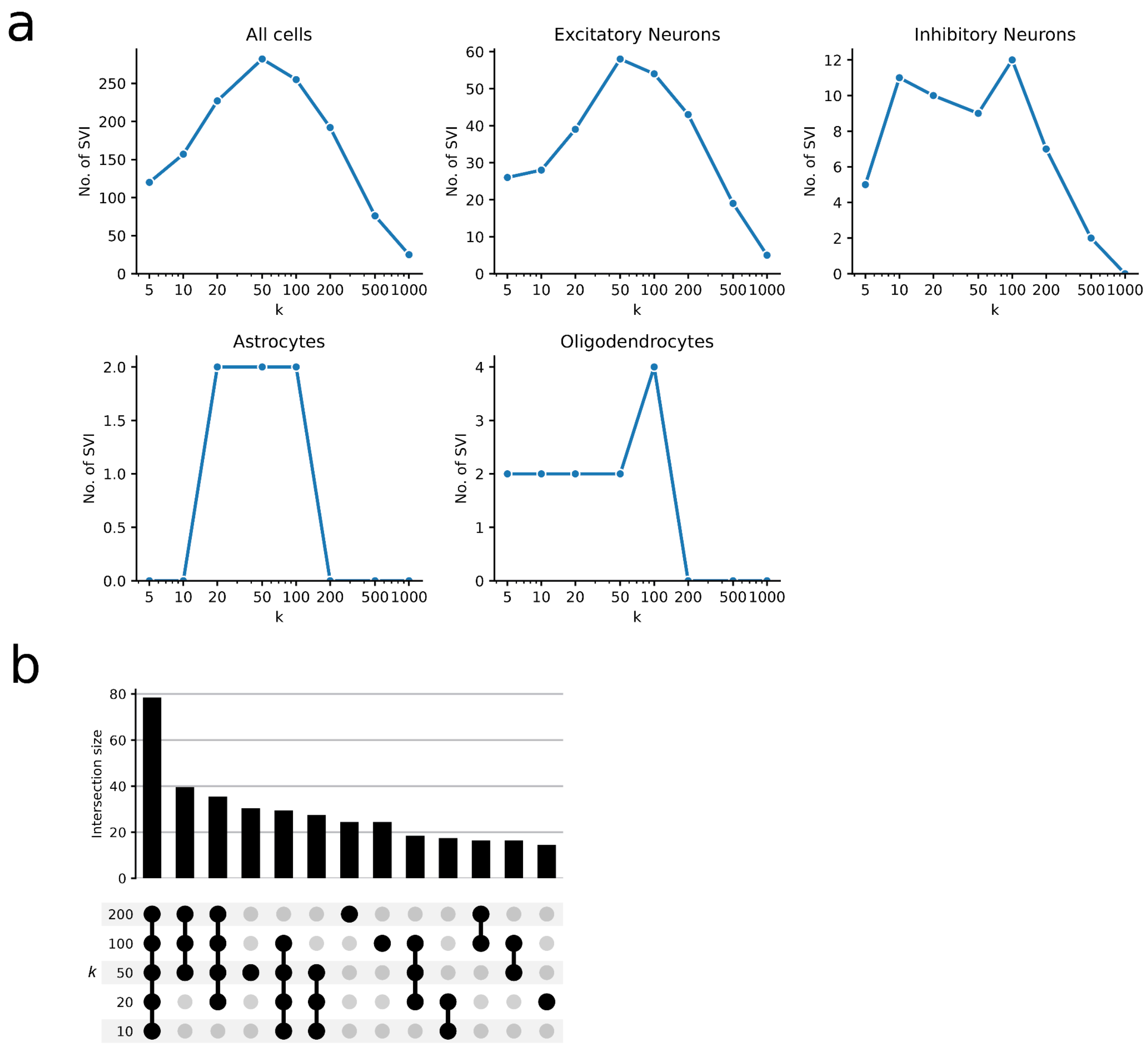

Figure S28. a) Number of significant SVIs detected by Spl-IsoFind using different numbers of nearest neighbors (*k*) when using all cells and for each cell type. **b)** The overlap in SVIs when using a different number of neighbors when using all cells.

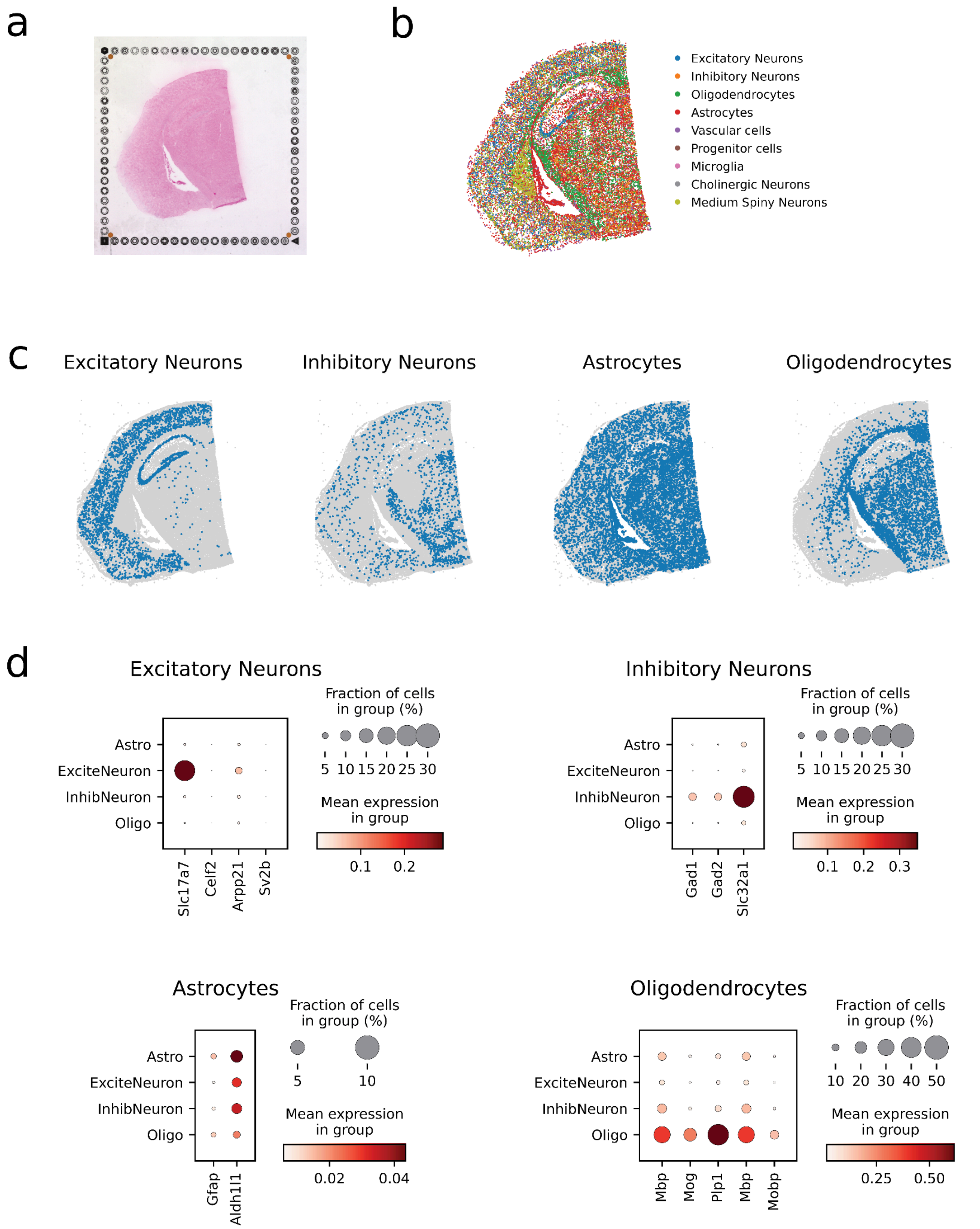

Figure S29. a) CytAssist image of the V1 brain slide. **b)** Cell-type annotations of the sample. Only cells annotated as singlets and cell types with >100 cells are plotted. **c)** Cell-type distribution and **d)** marker gene expression of the four major cell types in the Visium HD sample.

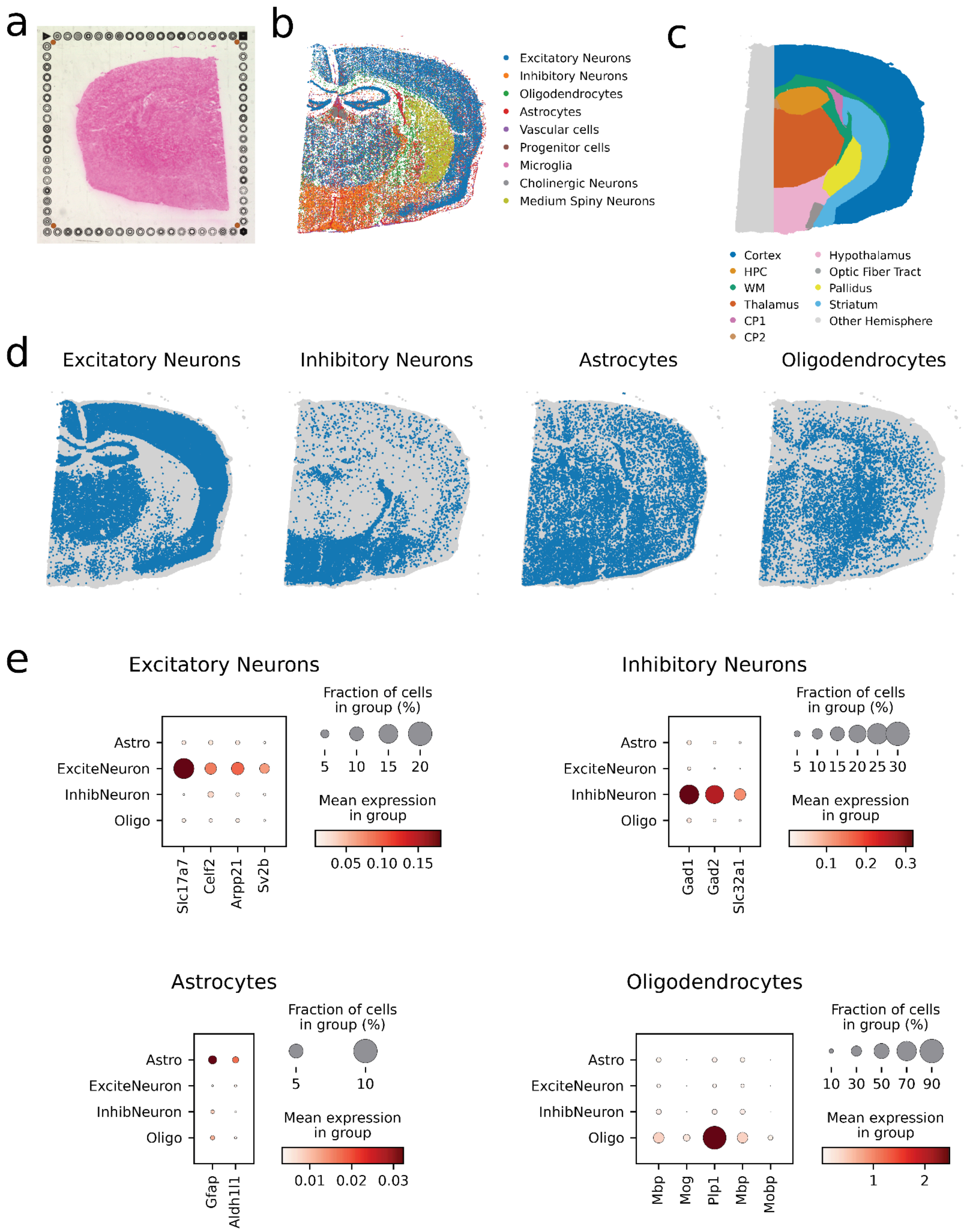

Figure S30. a) CytAssist image of the V2 brain slide. **b)** Cell-type annotations of the sample. Only cells annotated as singlets and cell types with >100 cells are plotted. **c)** Brain region annotation. **d)** Cell-type distribution and **e)** marker gene expression of the four major cell types in the Visium HD sample.

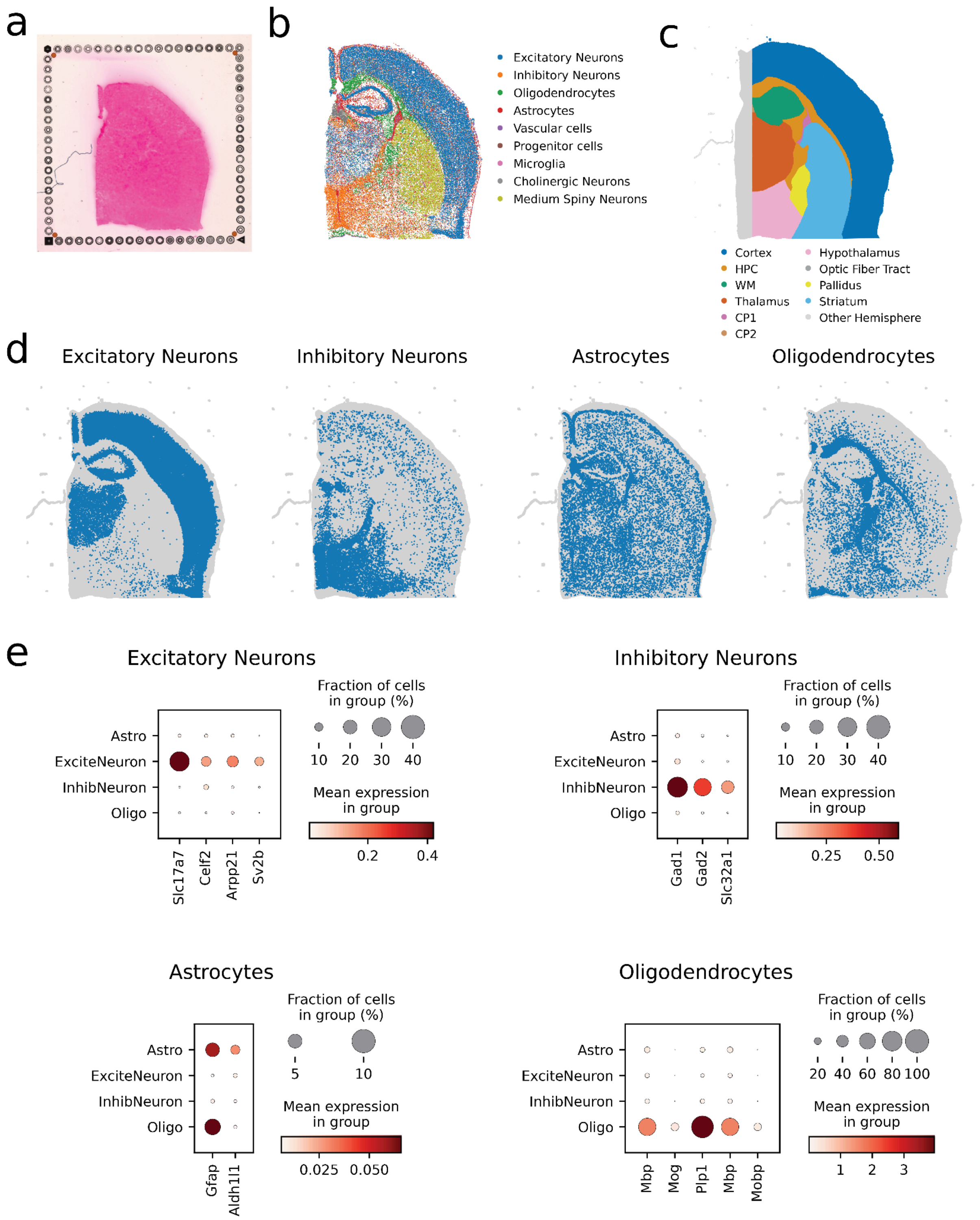

Figure S31. a) CytAssist image of the V3 brain slide. **b)** Cell-type annotations of the sample. Only cells annotated as singlets and cell types with >100 cells are plotted. **c)** Brain region annotation. **d)** Cell-type distribution and **e)** marker gene expression of the four major cell types in the Visium HD sample.

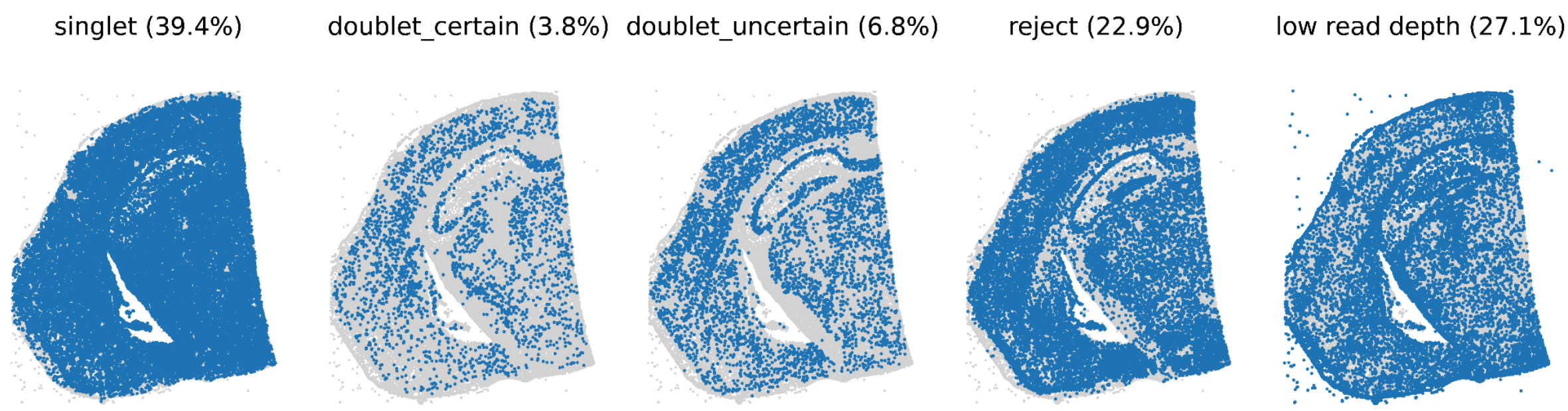

Figure S32. RCTD cell-assignment distribution in the V1 sample.

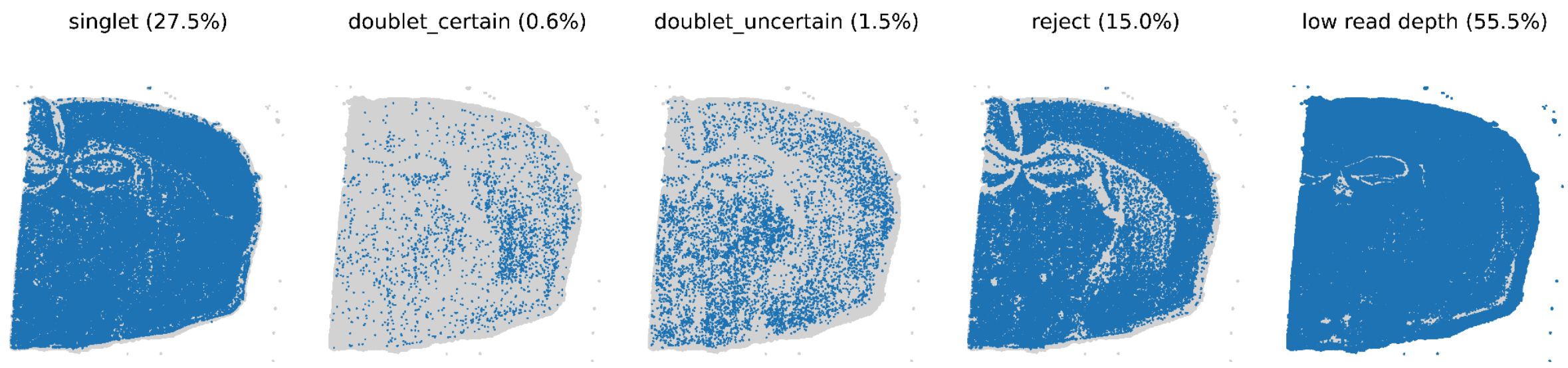

Figure S33. RCTD cell-assignment distribution in the V2 sample.

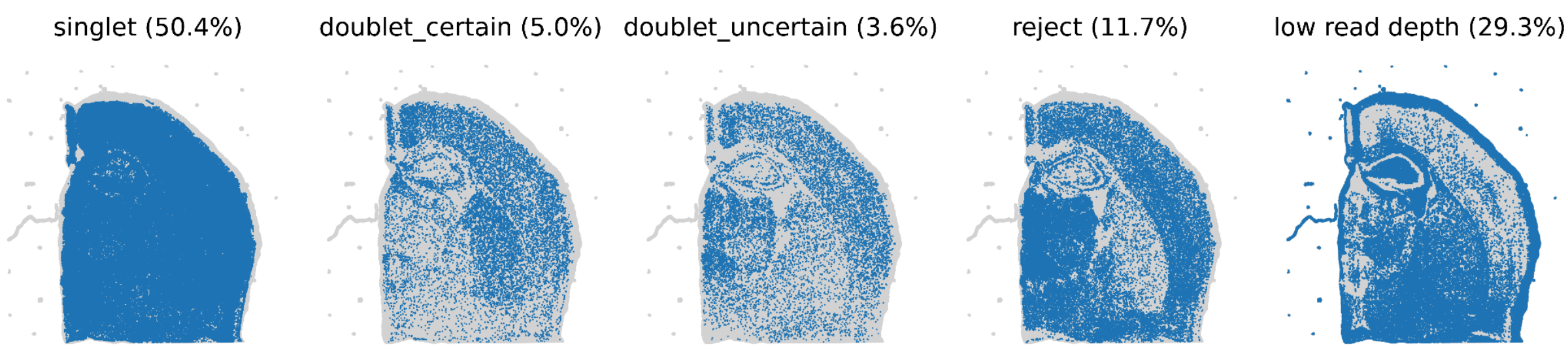

Figure S34. RCTD cell-assignment distribution in the V3 sample.

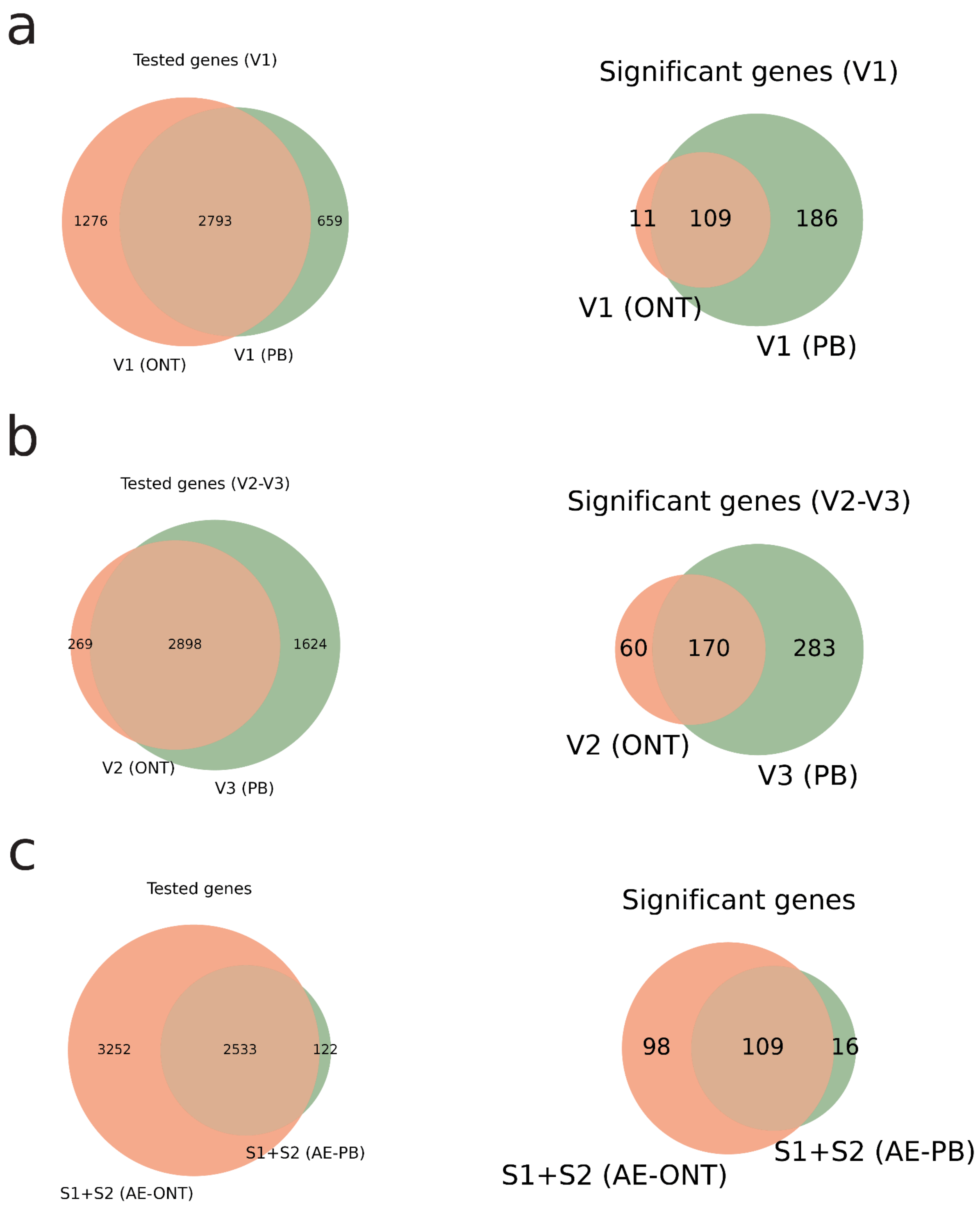

Figure S35. Overlap tested and significant genes ONT and PacBio

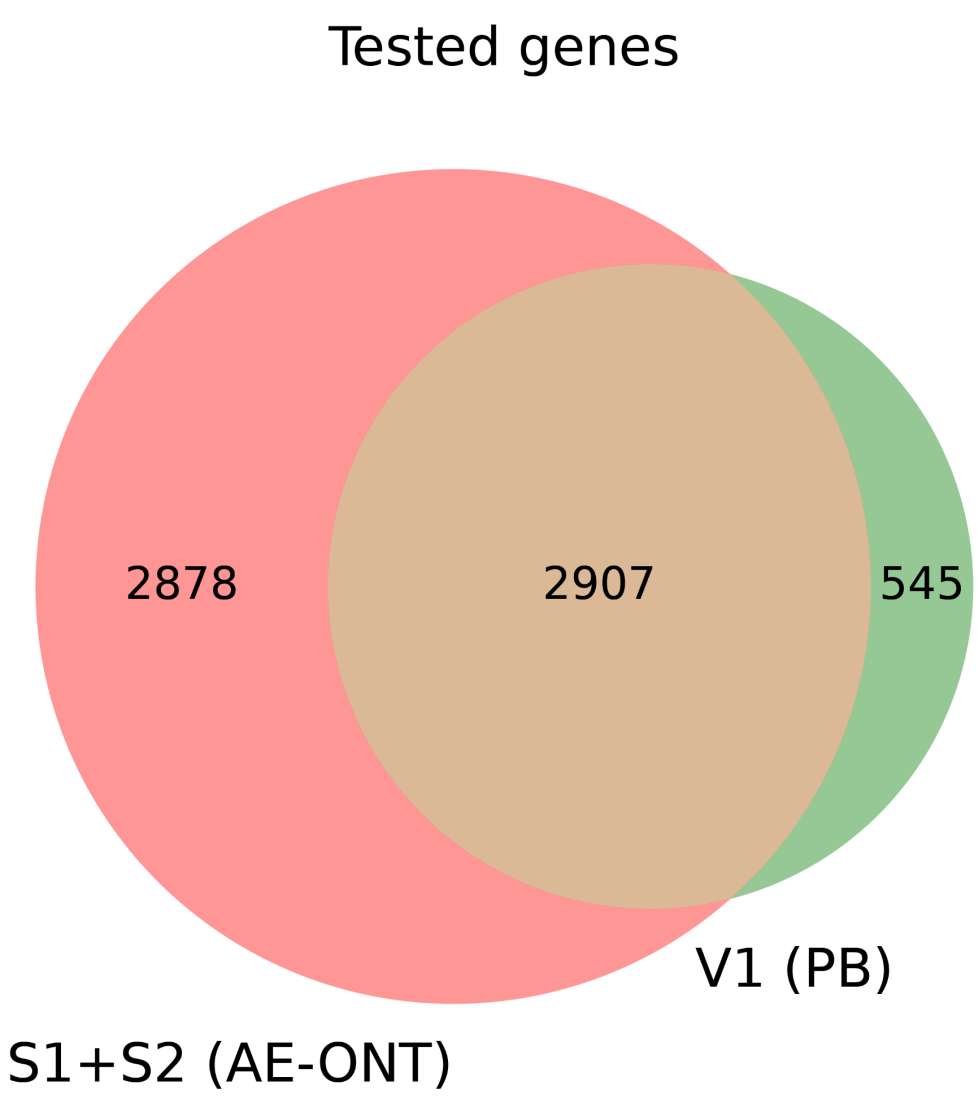

Figure S36. Overlap of significant SVIs between Sample1+2 (AE) ONT and Visium HD V1 PB data.

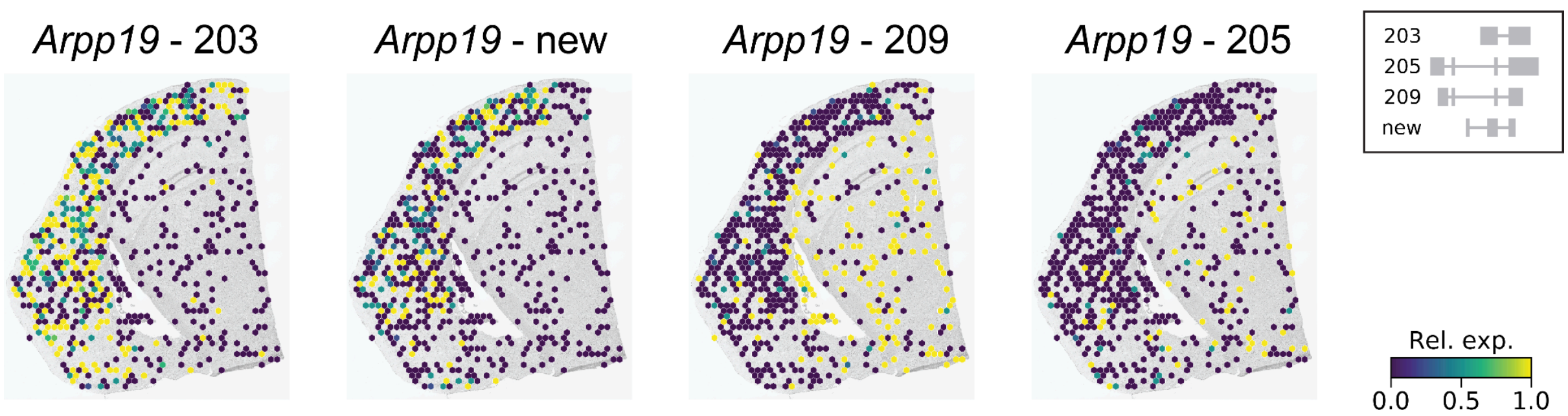

Figure S37. Spatial distribution of the four significant isoforms of *Arpp19* in the Visium HD data. Every hexagon shows the mean relative expression of the underlying cells (Methods).

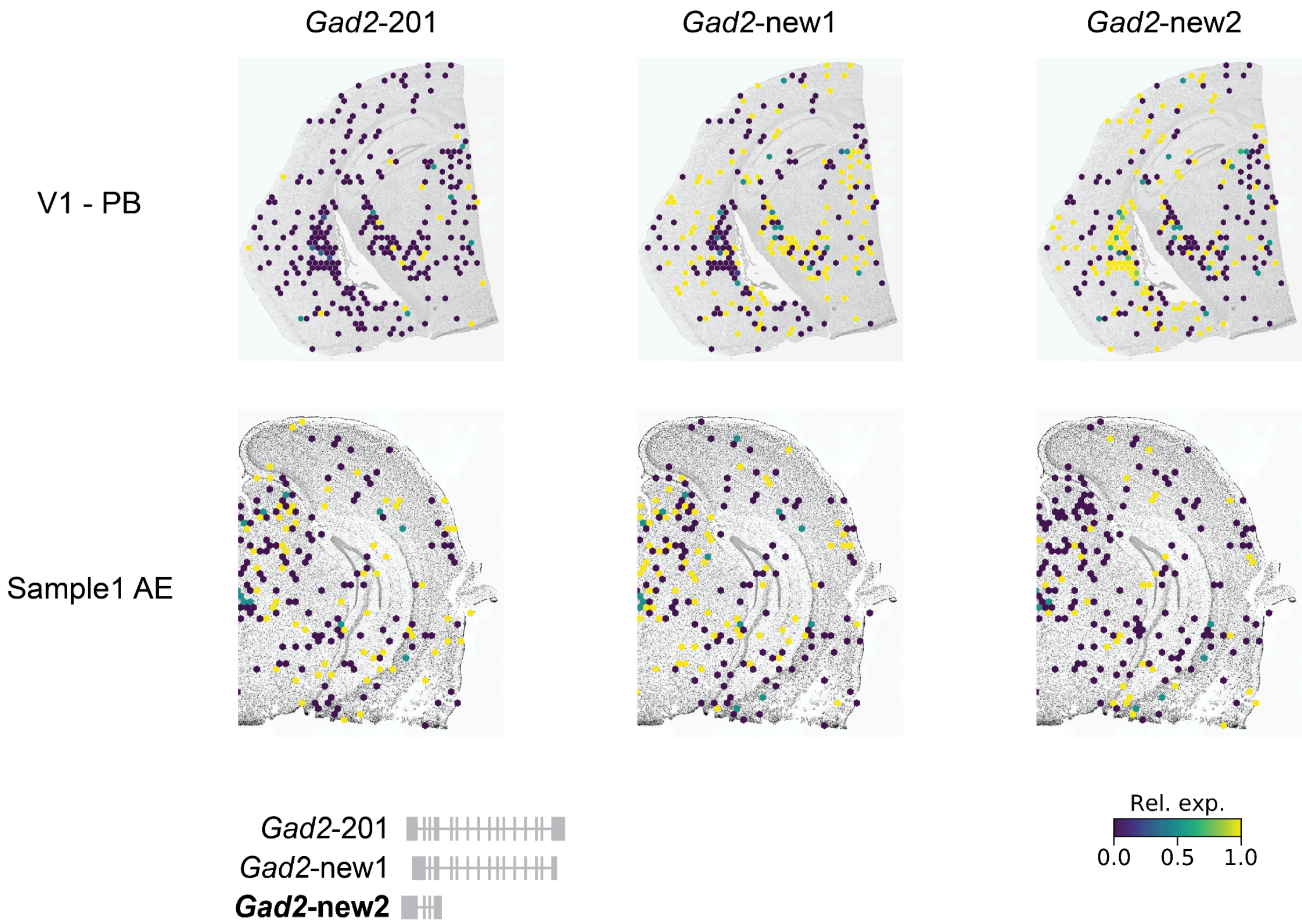

Figure S38. a) Spatial distribution of *Gad2* isoforms in V1-PB and Sample1 (AE). Every hexagon shows the mean relative expression of the underlying cells (Methods).
