# Supplementary material for: Spatial isoform sequencing at single-cell resolution reveals cell-type-specific spatial isoform variability in multiple brain cell types
Source: Nat Methods. 2026 Sep 4;23(9):1869–81. doi: 10.1038/s41592-026-03211-w (PMC13545012; doi:10.1038/s41592-026-03211-w)
Supplement: Supplementary file 2 [file 41592_2026_3211_MOESM2_ESM.pdf]

nature portfolio

Corresponding author(s): Hagen U. Tilgner

Last updated by author(s): 2026/06/27

Reporting Summary

Nature Portfolio wishes to improve the reproducibility of the work that we publish. This form provides structure for consistency and transparency in reporting. For further information on Nature Portfolio policies, see our Editorial Policies and the Editorial Policy Checklist.

Statistics

For all statistical analyses, confirm that the following items are present in the figure legend, table legend, main text, or Methods section.

n/a	Confirmed	
☐	☒	The exact sample size (*n*) for each experimental group/condition, given as a discrete number and unit of measurement
☐	☒	A statement on whether measurements were taken from distinct samples or whether the same sample was measured repeatedly
☐	☒	The statistical test(s) used AND whether they are one- or two-sided<br>*Only common tests should be described solely by name; describe more complex techniques in the Methods section.*
☒	☐	A description of all covariates tested
☐	☒	A description of any assumptions or corrections, such as tests of normality and adjustment for multiple comparisons
☒	☐	A full description of the statistical parameters including central tendency (e.g. means) or other basic estimates (e.g. regression coefficient) AND variation (e.g. standard deviation) or associated estimates of uncertainty (e.g. confidence intervals)
☐	☒	For null hypothesis testing, the test statistic (e.g. *F*, *t*, *r*) with confidence intervals, effect sizes, degrees of freedom and *P* value noted<br>*Give P values as exact values whenever suitable.*
☒	☐	For Bayesian analysis, information on the choice of priors and Markov chain Monte Carlo settings
☒	☐	For hierarchical and complex designs, identification of the appropriate level for tests and full reporting of outcomes
☒	☐	Estimates of effect sizes (e.g. Cohen's *d*, Pearson's *r*), indicating how they were calculated

Our web collection on statistics for biologists contains articles on many of the points above.

Software and code

Policy information about availability of computer code

Data collection

n.a.

Data analysis

Splitting PacBio reads: skera (v 1.4.0)
Long-read processing: Spl-IsoQuant-2 (v2.3, https://github.com/algbio/spl-IsoQuant)
Read alignment (part of Spl-IsoQuant-2 software): minimap2 (v2.26)
Simulating Nanopore data: modified version of Trans-NanoSim (https://github.com/andrewprzh/lrgasp-simulation)
Cell segmentation and barcode to cell assignment: SAW (version 7)
Cell-type indentification: RCTD (v2.2.1)
Slide alignment: spateo (version 1.1.0)
Differential isoform and exon testing using predefined regions: scisorseqr (https://github.com/noush-joglekar/scisorseqr, version 0.1.9) + scisorATAC (https://github.com/careenfoord/scisorATAC)
Spatially variable isoform testing: Spl-IsoFind (https://github.com/tilgnerlab/Spl-IsoFind) and ESDA (version 2.4.3)
GO-enrichment analysis: clusterProfiler (v4.18.4)
Barcode calling using SpaceRanger: SpaceRanger (version 4.0.1)

For manuscripts utilizing custom algorithms or software that are central to the research but not yet described in published literature, software must be made available to editors and reviewers. We strongly encourage code deposition in a community repository (e.g. GitHub). See the Nature Portfolio guidelines for submitting code & software for further information.

Data

Policy information about availability of data

All manuscripts must include a data availability statement. This statement should provide the following information, where applicable:

- Accession codes, unique identifiers, or web links for publicly available datasets
- A description of any restrictions on data availability
- For clinical datasets or third party data, please ensure that the statement adheres to our policy

Sequencing data is available on SRA with BioProject ID PRJNA1282707: https://www.ncbi.nlm.nih.gov/bioproject/PRJNA1282707. 10x Visium HD V2 data is publicly available at https://epi2me.nanoporetech.com/visium_hd_2025.06/. 10x Visium HD V3 data is publicly available at https://downloads.pacbcloud.com/public/dataset/Kinnex-single-cell-RNA/DATA-RevioSPRQ-Kinnex-VisiumHD-mouseBrain/1-Sreads/. The human hippocampus data is available at: https://knowledge.brain-map.org/data/ASP3B09DZ8PXDUYSHDH. Simulated data is available at https://doi.org/10.5281/zenodo.19616234. Intermediate analysis files and supplementary files needed for data processing are available at https://doi.org/10.5281/zenodo.19499423. Processed data is available for interactive visualization at spatialisoforms.weill.cornell.edu.

Research involving human participants, their data, or biological material

Policy information about studies with human participants or human data. See also policy information about sex, gender (identity/presentation), and sexual orientation and race, ethnicity and racism.

Reporting on sex and gender	n.a.
Reporting on race, ethnicity, or other socially relevant groupings	n.a.
Population characteristics	n.a.
Recruitment	n.a.
Ethics oversight	n.a.

Note that full information on the approval of the study protocol must also be provided in the manuscript.

Field-specific reporting

Please select the one below that is the best fit for your research. If you are not sure, read the appropriate sections before making your selection.

☒ Life sciences ☐ Behavioural & social sciences ☐ Ecological, evolutionary & environmental sciences

For a reference copy of the document with all sections, see nature.com/documents/nr-reporting-summary-flat.pdf

Life sciences study design

All studies must disclose on these points even when the disclosure is negative.

Sample size	No sample size calculations were performed in this study
Data exclusions	No data was excluded from the study
Replication	1. We compared verified Spl-IsoQuant-2's transcript assignments by comparing reads sequenced using both ONT and PacBio. 99.4% of such read pairs were assigned to the same isoform.<br>2. We verified our detected significant spatially variable isoforms by comparing two consecutive of the same mouse. This showed that most isoforms are detected in both samples or only in the sample with the highest read depth.<br>3. We compared our results obtained after analysing Spl-ISO-Seq2 to VisiumHD data. This showed that the overlap of signicant spatially variable isoforms was greater than expected even though one sample was more anterior compared to the other.
Randomization	Randomization was not applicable during this study
Blinding	Blinding was not applicable to this study, as all analyses were computational. No subjective scoring or outcome assessment was involved that could introduce observer bias.

Reporting for specific materials, systems and methods

We require information from authors about some types of materials, experimental systems and methods used in many studies. Here, indicate whether each material, system or method listed is relevant to your study. If you are not sure if a list item applies to your research, read the appropriate section before selecting a response.

Materials & experimental systems

n/a	Involved in the study
☒	☐ Antibodies
☒	☐ Eukaryotic cell lines
☒	☐ Palaeontology and archaeology
☐	☒ Animals and other organisms
☒	☐ Clinical data
☒	☐ Dual use research of concern
☒	☐ Plants

Methods

n/a	Involved in the study
☒	☐ ChIP-seq
☒	☐ Flow cytometry
☒	☐ MRI-based neuroimaging

Animals and other research organisms

Policy information about studies involving animals; ARRIVE guidelines recommended for reporting animal research, and Sex and Gender in Research

Laboratory animals	1 male C57BL P56 mouse for the Stereo-seq protocol<br>3 male C57BL P56 mice were used for validation
Wild animals	No wild animals were used during this study
Reporting on sex	For the stereo-seq, only one sample was used which was a male sample. As such, we used a male sample for validation as well.
Field-collected samples	No field-collected samples
Ethics oversight	All experiments were conducted in accordance with relevant NIH guidelines and regulations, related to the Care and Use of Laboratory Animals tissue. Animal procedures were performed according to protocols approved by the Research Animal Resource Center at Weill Cornell College of Medicine.

Note that full information on the approval of the study protocol must also be provided in the manuscript.

Plants

Seed stocks	n.a.
Novel plant genotypes	n.a.
Authentication	n.a.
