# Supplementary material for: Spatial isoform sequencing at single-cell resolution reveals cell-type-specific spatial isoform variability in multiple brain cell types
Source: Nat Methods. 2026 Sep 4;23(9):1869–81. doi: 10.1038/s41592-026-03211-w (PMC13545012; doi:10.1038/s41592-026-03211-w)
Supplement: Supplementary file 3 [file 41592_2026_3211_MOESM3_ESM.pdf]

nature portfolio

Peer Review File

Spatial isoform sequencing at single-cell resolution reveals cell-type-specific spatial isoform variability in multiple brain cell types

Corresponding Author: Dr Hagen Tilgner

Parts of this Peer Review File have been redacted as indicated to remove third-party material.

This file contains all editorial decision letters in order by version, followed by all author rebuttals in order by version.

Version 0:

Decision Letter:

10th Dec 2025

Dear Hagen,

Thank you for your patience. Your Article, "Spatial isoform sequencing at sub-micrometer single-cell resolution reveals novel patterns of spatial isoform variability in brain cell types", has now been seen by three reviewers. As you will see from their comments below, although the reviewers find your work of considerable potential interest, they have raised a number of concerns. We continue to be interested in potentially publishing your manuscript in Nature Methods, but would like to evaluate your response to these concerns before we reach a final decision on publication.

We therefore invite you to revise your manuscript to address these concerns.

We are committed to providing a fair and constructive peer-review process. Do not hesitate to contact us if there are specific requests from the reviewers that you believe are technically impossible or unlikely to yield a meaningful outcome.

When revising your paper:

* include a point-by-point response to the reviewers and to any editorial suggestions

* please underline/highlight any additions to the text or areas with other significant changes to facilitate review of the revised manuscript

* address the points listed described below to conform to our open science requirements

* ensure it complies with our general format requirements as set out in our guide to authors at www.nature.com/naturemethods

* resubmit all the necessary files by using the link below to access your home page

Link Redacted

Note: This URL links to your confidential account and associated information about manuscripts you may have submitted, or that you are reviewing for us. If you wish to forward this email to co-authors, please delete this link.

We hope to receive your revised paper within 2-3 months. If you are substantially delayed, please let us know. In this event, we will still be happy to reconsider your paper at a later date so long as nothing similar has been accepted for publication at Nature Methods or published elsewhere.

OPEN SCIENCE REQUIREMENTS

REPORTING SUMMARY
When revising your manuscript, please update your reporting summary.

Reporting summary: https://www.nature.com/documents/nr-reporting-summary.zip

If your paper includes custom software, we also ask you to complete a supplemental reporting summary.

Software supplement: https://www.nature.com/documents/nr-software-policy.pdf

Please submit these with your revised manuscript. They will be available to reviewers to aid in their evaluation if the paper is re-reviewed. If you have any questions about the checklist, please see http://www.nature.com/authors/policies/availability.html or contact me.

Please note that these forms are dynamic 'smart pdfs' and must therefore be downloaded and completed in Adobe Reader. We will then flatten them for ease of use by the reviewers. If you would like to reference the guidance text as you complete the template, please access these flattened versions at http://www.nature.com/authors/policies/availability.html.

For any revision that includes light microscopy data, we ask our authors to please include a completed light microscopy reporting table [https://www.nature.com/documents/Light_microscopy_reporting_table.xlsx] to ensure the methods are described thoroughly. The table will be available to reviewers and ultimately published should the manuscript be accepted at the journal.

Finally, please ensure that you retain unprocessed data and metadata files after publication, ideally archiving data in perpetuity, as these may be requested during the peer review and production process or after publication if any issues arise.

EXTENDED DATA FIGURES
When re-submitting your manuscript, please ensure that any supplementary figures and tables that are crucial to the manuscript's conclusions are converted into Extended Data figures and tables to increase visibility of these data. Extended Data figures and tables are online-only (present in the online PDF and full-text HTML versions of the paper), peer-reviewed display items that provide essential background to the article but are not included in the main article due to space constraints. A maximum of ten Extended Data display items (figures and tables) is permitted.

DATA AVAILABILITY
We strongly encourage you to deposit all new data associated with the paper in a persistent repository where they can be freely and enduringly accessed. We recommend submitting the data to discipline-specific and community-recognized repositories; a list of repositories is provided here: http://www.nature.com/sdata/policies/repositories

All novel DNA and RNA sequencing data, protein sequences, genetic polymorphisms, linked genotype and phenotype data, gene expression data, macromolecular structures, and proteomics data must be deposited in a publicly accessible database, and accession codes and associated hyperlinks must be provided in the "Data Availability" section.

For papers containing bioimaging data, we strongly recommend depositing the data to Bioimage Archive (https://www.ebi.ac.uk/bioimage-archive/). Associated accession codes and hyperlinks should be provided in the "Data Availability" section.

Refer to our data policies here: https://www.nature.com/nature-research/editorial-policies/reporting-standards#availability-of-data

To further increase transparency, we encourage you to provide, in tabular form, the data underlying the graphical representations used in your figures. This is in addition to our data-deposition policy for specific types of experiments and large datasets. For readers, the source data will be made accessible directly from the figure legend. Spreadsheets can be submitted in .xls, .xlsx or .csv formats. Only one (1) file per figure is permitted: thus if there is a multi-paneled figure the source data for each panel should be clearly labeled in the csv/Excel file; alternately the data for a figure can be included in multiple, clearly labeled sheets in an Excel file. File sizes of up to 30 MB are permitted. When submitting source data files with your manuscript please select the Source Data file type and use the Title field in the File Description tab to indicate which figure the source data pertains to.

Please include a "Data availability" subsection in the Online Methods. This section should inform readers about the availability of the data used to support the conclusions of your study, including accession codes to public repositories, references to source data that may be published alongside the paper, unique identifiers such as URLs to data repository entries, or data set DOIs, and any other statement about data availability. At a minimum, you should include the following statement: "The data that support the findings of this study are available from the corresponding author upon request", describing which data is available upon request and mentioning any restrictions on availability. If DOIs are provided, please include these in the Reference list

(authors, title, publisher (repository name), identifier, year). For more guidance on how to write this section please see: http://www.nature.com/authors/policies/data/data-availability-statements-data-citations.pdf

CODE AVAILABILITY
Please include a “Code Availability” subsection in the Online Methods which details how your custom code is made available. Only in rare cases (where code is not central to the main conclusions of the paper) is the statement “available upon request” allowed (and reasons should be specified).

We request that you deposit code in a DOI-minting repository such as Zenodo, Gigantum or Code Ocean and cite the DOI in the Reference list. We also request that you use code versioning and provide a license.

For more information on our code sharing policy and requirements, please see: https://www.nature.com/nature-research/editorial-policies/reporting-standards#availability-of-computer-code

MATERIALS AVAILABILITY
As a condition of publication in Nature Methods, authors are required to make unique materials promptly available to others without undue qualifications.

Authors reporting new chemical compounds must provide chemical structure, synthesis and characterization details. Authors reporting mutant strains and cell lines are strongly encouraged to use established public repositories.

More details about our materials availability policy can be found at https://www.nature.com/nature-portfolio/editorial-policies/reporting-standards#availability-of-materials

SUPPLEMENTARY PROTOCOL
To help facilitate reproducibility and uptake of your method, we ask you to prepare a step-by-step Supplementary Protocol for the method described in this paper. We encourage authors to share their step-by-step experimental protocols on a protocol sharing platform of their choice and report the protocol DOI in the reference list. Nature Portfolio's protocols.io is a free-to-use and open resource for protocols; protocols deposited onto protocols.io are citable and can be linked from the published article. More details can found at protocols.io.

ORCID
Nature Methods is committed to improving transparency in authorship. As part of our efforts in this direction, we are now requesting that all authors identified as ‘corresponding author’ on published papers create and link their Open Researcher and Contributor Identifier (ORCID) with their account on the Manuscript Tracking System (MTS), prior to acceptance. This applies to primary research papers only. ORCID helps the scientific community achieve unambiguous attribution of all scholarly contributions. You can create and link your ORCID from the home page of the MTS by clicking on ‘Modify my Springer Nature account’. For more information please visit please visit www.springernature.com/orcid.

Please do not hesitate to contact me if you have any questions or would like to discuss these revisions further. We look forward to seeing your revised manuscript and thank you for the opportunity to consider your work.

Best regards,
Nina

Nina Vogt, PhD
Senior Editor
Nature Methods

Reviewers' Comments:

Reviewer #1 (Remarks to the Author):

A. Summary of the key results
The paper entitled “Spatial isoform sequencing at sub-micrometer single-cell resolution reveals novel patterns of spatial isoform variability in brain cell types” by Michielsen et al addresses the important question of spatial variability of expression of RNA isoforms. It applies to that end new methods to the latest generation of tools designed to perform sequence-based spatial transcriptomics. The paper is clearly written and emphasizes well the importance to develop such tools to better intercept the diversity of RNA isoform expression at single-cell and spatial resolution. The authors combined into a spatial transcriptomics

framework one experimental and two computational tools: (1) Spl-ISO-Seq2 for the preparation of the libraries and sequencing of the samples, which is described with the use of Slide-SeqV2, although the authors also demonstrate at the end of their work its applicability to Visium HD 3' long-read. Sequencing is done either on Oxford Nanopore or PacBio; (2) Spl-IsoQuant-2 is a tool for the genome-based analysis of spatial long RNA reads obtained with Spl-ISO-Seq2, also compatible with PacBio and Oxford Nanopore sequencings. Spl-IsoQuant performs barcode detection, read-to-isoform assignment, quantification and PCR deduplication. (3) Spl-IsoFind uses Moran's I score, a global spatial autocorrelation score, to detect spatially variable isoforms in long-read spatial transcriptomics data.

B. Originality and significance: if not novel, please include reference

Tilgner's lab has already significantly contributed to the field of long read single cell transcriptomics, and this present paper details a new approach to identify differentially expressed RNA isoforms, based on Moran's I score, previously used in spatial biology. The article mentions its use to global gene expression (refs 34-36), but then develops its own method adapted for isoform detection, which is novel.

C. Data & methodology: validity of approach, quality of data, quality of presentation

Their approach based on Moran's I score uncovers patterns that were not previously captured by existing predefined-region comparisons. This approach allows them to identify the complex pattern of isoform expression of Tnnc1 in excitatory neurons. This approach leads to the observation that many spatial isoform signals are not only driven by different cell-type compositions. In total, the work is of great quality, and paves the way for future biological investigations. Some conclusions remain at this time limited because the statistical power of the analysis is low (n=2). See for instance the huge error bars in Figures 4C, 5G, S5. Maybe a comparison with the existing literature could clarify somehow this point (compare for instance Figure 3A with Figure 4B from ref 19, in which different contrasts were reported between brain areas, which did not report a particular difference in midbrain). The authors should clarify these different points.

Moran's test can be affected by the coding of the neighborhood structure, which can greatly influence the observations. At which point the selected neighborhood method was adequate, and what can be the impact of such choices on the observed results?

A current limitation of Spl-IsoFind is its low detection rate, which is not unique to the tool, and which can clearly improve by using a higher read depth, as evidenced by the comparison between Sample 1, sequenced with two ONT flow cells, and Sample 2, sequenced with only one. The authors could perhaps discuss what could be possible alternatives, either experimental or computational.

The authors applied their approach to adult male mouse brain, thus revealing cell-type specific isoform changes between predefined regions and also in previously unrecognized spatial patterns.

The use of more resolutive representations like Slide-SeqV2 requires the capacity to read with a high precision many spatial barcodes. One strength of their approach is its capacity to quantify with a ghigh precision these sequences. One interest of their approach is thus to detect well all the different barcodes, even when less precise sequencer, i.e. Oxford Nanopore. This is an important aspect because this technology generates much more sequences than PacBio.

D. Appropriate use of statistics and treatment of uncertainties

A careful evaluation of the validity of the use of Moran's I test in Spl-IsoFind by a statistician is worth being made.

E. Conclusions: robustness, validity, reliability

The proposed approach is of interest and the paper is well written. Its conclusions are appropriate.

F. Suggested improvements: experiments, data for possible revision

Their analysis of an adult mouse brain recapitulates well already known differential isoform abundance between known brain regions, and find some new events. Introduction of Spl-IsoFind allows to identify spatial isoform patterns outside predefined regions. The 2 independent approaches coincide in the identification of known switches, such as Rps24 in oligodendrocytes and Snap25. In that latter case, the authors were able to establish a possible origin of its variability in excitatory neurons. As expression of the two isoforms of SNAP-25 resulting from alternative splicing are possibly regulated by sex (see for instance Irfan, Sci Rep 9, 6403, 2019), the authors may document the differences between males (used in the present study) and females. This can greatly enlarge the list of differentially expressed isoforms.

Minor

- In Figure 3k, indicate scale + define more clearly the relative expression (for instance by using 2 distinct colors for the 2 Gria2 isoforms).
- It is explained that the Visium HD long-read data was generated without long-molecule and exome selection, resulting in a shorter overall read length and a lower percentage of spliced reads compared to Spl-ISO-Seq2. However, 7.8M have an isoform assigned vs 8.0M in Sample 1 (AE)) even though four ONT flow cells were used compared to two flow cells for Sample 1 (AE). This seems to be a very small difference. Please explain.
- 500 ng of the cDNA collected from the previous step was used as input for the exome enrichment, which represents a standard amount for exome capture, but a huge amount in term of cDNA at the end of a single-cell experiment. Do the authors use a particular procedure to perform larger amplification? Can it introduce biases in their measurements?

G. References: appropriate credit to previous work?

Appropriate

H. Clarity and context: lucidity of abstract/summary, appropriateness of abstract, introduction and conclusions

Appropriate

Reviewer #2 (Remarks to the Author):

Summary of Paper

In this study, the authors adapt their previously developed Spl-ISO-Seq long-read spatial transcriptomics method to the higher-resolution Stereo-Seq platform. They combine the platform's sub-micrometer barcoding chemistry with their established exome

enrichment and long-molecule selection workflow to generate full-length, spatially barcoded cDNAs from mouse brain sections. The resulting cDNAs are sequenced using both Oxford Nanopore and PacBio technologies. To process these data, they introduce Spl-IsoQuant-2, a tweaked version of their exsiting software to adapt barcode-parsing and isoform-assignment algorithm to Stereo-Seq's more complex read structure, and Spl-IsoFind, a software tool that identifies spatially variable isoforms using Moran's I statistics, a metric commonly used to find varying patterns of gene expression in spatial transcriptomics . Applying this pipeline, the authors report isoform-level spatial expression maps at near-single-cell resolution, identify examples of spatially variable isoforms such as Snap25 and Rps24, and show that some of these patterns cannot be explained by cell-type composition alone. Finally, they apply their software to publicly available Visium HD long-read data to demonstrate cross-platform compatibility and reproducibility.

Methodological Innovation and Biological Impact

Overall, I find the novel the methods presented here to represent very modest changes to either their (quite good) existing algorithms (Spl-IsoQuant-2 versus the Spl-ISO-Seq pipeline) or adaptation of widely used metrics in spatial transcriptomics for alternative splicing (Moran-I statistic). The biological findings are largely anecdotal and not well-benchmarked. Thus, both the methodological innovation and biological impact are low.

The experimental and computational workflows build directly on the authors' prior Spl-ISO-Seq publications (Nat Commun 2021; NAR 2023; Nat Biotech 2023; and especially Foord, Nat Commun 2025), which combined spatial barcoding with long-read sequencing for isoform-level analysis. The present study largely extends that approach to the higher-resolution Stereo-Seq platform, accompanied by minor software updates (Spl-IsoQuant-2 and Spl-IsoFind). While the adaptation is technically competent and the integration of long-read spatial data at near-single-cell resolution is valuable, the advance is primarily in adapting their existing code rather than a conceptually new algorithmic or experimental approach. The improvement in spatial resolution enables better cellular resolution, but this comes from leveraging an existing commercial platform. Overall, this work represents an update to the author's already published method rather than a fundamentally new paradigm, and thus the innovation here is at best moderate.

One of the two main technical innovations in this paper is Spl-IsoQuant-2, an extension of the authors' IsoQuant pipeline. This component is technically well executed, but the innovation is primarily using standard "old-school" methods (i.e. k-mer matching followed by local alignment) to handle the noiser barcode reads inherent to Oxford Nanopore. In fact, the barcode parsing advances touted as new are already described in their previous work (On p. 5-6 Foord et al uses k-mer indexing followed by Smith Waterman!). Thus, the conceptual novelty for this method over their own previous method is essentially zero. The second innovation is Spl-IsoFind, which identifies spatially variable isoforms using Moran's I, a standard measure of spatial autocorrelation. While this is a sensible extension of existing spatial-omics algorithms to isoform data, it represents an application of a well-established statistic rather than a new analytical framework.

The biological insights derived from the study are also limited. Most examples (for instance Snap25 and Rps24) validate known patterns of isoform variation rather than revealing new mechanisms or cell-state relationships. The authors do not convincingly show that the information gained could not be obtained more directly from single-cell or single-nucleus long-read sequencing combined with anatomical annotation. The biological findings here seem mostly anecdotal. While Nature Methods is focused on methods development, I feel the best way to highlight the utility of a new method is to describe some new biology that would inspire other users to adapt the method. This aspect of the paper – exciting the biologist with the potential of the new types of analyses now possible was lacking.

Overall, it is important that the community have good workflows to ingrate long read sequencing with spatial transcriptomics. But this workflow and protocol presented are not substantially different than what has already been published by this group nor are there any new biological insights provided beyond purely descriptive anecdotes.

Minor Comments and Suggested Revisions

1. Per-cell QC metrics (UMIs/reads/genes per cell) are missing. One of the main advantages of this workflow over previous work is the opportunity to achieve single cell resolution. But basic single-cell–level QC metrics are omitted. For example, median and mean UMIs per segmented cell, genes detected per cell, isoforms per cell, and the fraction of reads assigned per cell. The supplement reports "mean reads per gene/isoform" and various global tallies, but not per-cell coverage, which makes it hard to judge sensitivity and to compare platforms.
2. Cost, throughput, and depth required for power. It would be helpful if the authors provided a clear cost table for one mouse coronal section: array prep, enrichment, ONT/PacBio runs, and expected depth to reach power for detecting spatially variable isoforms (SVIs) at given effect sizes. Include practical guidance on how many flow cells are needed for typical tissues, since Sample 1 used more ONT depth than Sample 2 and yielded substantially more events
3. Cell assignment and segmentation details. The Methods should spell out exactly how capture barcodes are mapped to segmented nuclei, thresholds for singlet calls, and doublet exclusion criteria. Right now the text notes ssDNA nuclear staining and that only RCTD singlets are used, but it lacks quantitative thresholds and error rates. Please add: number of spots assigned per cell, distance rules, and an empirical doublet rate estimate.
4. The paper would be improved with benchmarking (or even discussion) for expected numbers of differentially utilized isoforms in gold standard datasets. They identify 1-4% of DE genes had differential isoform use. Is that similar to what is known in their prior work? Single cell or bulk approaches? Is this because their sensitivity is low?
5. The algorithm implements several conservative decisions to minimize the chance of doublets or mixed cell spots. The tradeoff is likely that they have very low depth of UMI per cell, but these are not reported so it is difficult to get a feel for this.

Reviewer #3 (Remarks to the Author):

This manuscript presents Spl-ISO-Seq2, an innovative spatial transcriptomics method that achieves 500nm resolution with 220nm dot sizes, enabling true single-cell long-read isoform analysis. The integrated computational tools, Spl-IsoQuant-2 and Spl-IsoFind, form a novel framework for robust isoform quantification and spatial pattern detection, effectively revealing significant variations in genes like Nptn and Phactr1. Through its innovative analytical workflow, the study convincingly reveals significant isoform expression variations with specific spatial patterns within well-characterized cell types.

However, I have some concerns regarding certain aspects of the manuscript.
1. The manuscript would benefit from a deeper biological analysis of a small number of representative SVIs, going beyond the current descriptive presentation. A more in-depth analysis of their data For example, the authors already highlight the SNAP25 isoform variability mainly occurred in excitatory neurons. This echos the recent findings that excitatory neurons are evolutionarily more diverse than inbibitory neurons in Macaque V1 and multiple other species and brain regions. There are many subtypes of excitatory neurons with various layer distribution and project targets. It would be very interesting to see whether there are correlations between the isoform, synampic release modes, plasticity and isoform distribution.

2. Following the same line, it is recommended to include pathway enrichment analysis (such as GO and KEGG) to associate spatially variable isoforms (SVIs) with known neural pathways and further reveal the biological meaning of these SVIs.

3. Quantify the frequency of concatenated cDNA molecules in raw data to underscore the necessity of the deconcatenation algorithm.

4. The barcode identification in Spl-IsoQuant-2 demonstrates extremely high accuracy (~99.9%), but the recall rate is approximately 76.62%. This means about 24% of valid reads are discarded. Please discuss how this false-negative rate might affect downstream quantification analyses, and indicate whether any correction methods were applied.

5. Using Moran’s I in Spl-IsoFind to identify spatially variable isoforms (SVIs) is innovative. Please briefly compare its performance with existing spatial gene analysis methods adapted for this purpose to highlight its advantages.

6. Fig. S3 shows that the correlation between long and short read gene expression in Sample 1 (4K) (Pearson r=0.67) is noticeably lower than in other samples. Please explain the reasons for this discrepancy and whether this sequencing approach is less effective for genes associated with brain diseases.

7. When comparing datasets, as illustrated in Fig. 4a more detailed explanation of the potential biases inherent in targeted sequencing is essential.

8. Fig. 4i depicts four isoforms of Arpp19 with highly complex spatial patterns. How can this biological complexity be interpreted? Are there known mechanisms or functional implications associated with such diverse spatial isoform distributions?

9. For newly identified high-confidence isoforms (e.g., Arpp19), it would be beneficial to conduct preliminary bioinformatics functional predictions to gain insights into their potential biological roles.

10. The study emphasizes Spl-ISO-Seq2's 500 nm resolution but lacks direct comparisons with other methods. Providing contrasts in resolution, throughput, cost, and other key metrics would better highlight its competitive advantages.

11. It is recommended to perform spatial multi-omics integration, such as examining the relationship between the spatial expression patterns of specific isoforms and chromatin accessibility, to explore potential regulatory mechanisms.

In summary, this manuscript describes a technically solid and conceptually important advance in spatial isoform profiling, with clear potential impact and broad relevance. With some additional clarifications, slightly more explicit positioning relative to existing methods, and further interrogation to reveal the biological relevance of the isofrm variation it would be well-suited to the goals of Nature Methods as a practically useful and influential methodological article.

Reviewer #3 (Remarks on code availability):

Access to the CodeOcean environment may be restricted for some users. To maximize transparency and reproducibility, I recommend that the authors also provide their code on a universally accessible platform such as GitHub.

Version 1:

Decision Letter:

31st Mar 2026

Dear Hagen,

Your Article, "Spatial isoform sequencing at sub-micrometer single-cell resolution reveals novel patterns of spatial isoform

variability in brain cell types", has now been seen again by the reviewers. As you will see from their comments below, although the reviewers continue to find your work of considerable potential interest, they have raised a number of concerns. We continue to be interested in potentially publishing your manuscript in Nature Methods, but would like to evaluate your response to these concerns before we reach a final decision on publication. We therefore invite you to revise your manuscript to address these concerns.

We are committed to providing a fair and constructive peer-review process. Do not hesitate to contact us if there are specific requests from the reviewers that you believe are technically impossible or unlikely to yield a meaningful outcome.

When revising your paper:

* include a point-by-point response to the reviewers and to any editorial suggestions

* please underline/highlight any additions to the text or areas with other significant changes to facilitate review of the revised manuscript

* address the points listed described below to conform to our open science requirements

* ensure it complies with our general format requirements as set out in our guide to authors at www.nature.com/naturemethods

* resubmit all the necessary files by using the link below to access your home page

Link Redacted

Note: This URL links to your confidential account and associated information about manuscripts you may have submitted, or that you are reviewing for us. If you wish to forward this email to co-authors, please delete this link.

We hope to receive your revised paper within 3-4 weeks. If you are substantially delayed, please let us know. In this event, we will still be happy to reconsider your paper at a later date so long as nothing similar has been accepted for publication at Nature Methods or published elsewhere.

OPEN SCIENCE REQUIREMENTS

REPORTING SUMMARY
When revising your manuscript, please update your reporting summary.

Reporting summary: https://www.nature.com/documents/nr-reporting-summary.zip

If your paper includes custom software, we also ask you to complete a supplemental reporting summary.

Software supplement: https://www.nature.com/documents/nr-software-policy.pdf

Please submit these with your revised manuscript. They will be available to reviewers to aid in their evaluation if the paper is re-reviewed. If you have any questions about the checklist, please see http://www.nature.com/authors/policies/availability.html or contact me.

Please note that these forms are dynamic 'smart pdfs' and must therefore be downloaded and completed in Adobe Reader. We will then flatten them for ease of use by the reviewers. If you would like to reference the guidance text as you complete the template, please access these flattened versions at http://www.nature.com/authors/policies/availability.html.

For any revision that includes light microscopy data, we ask our authors to please include a completed light microscopy reporting table [https://www.nature.com/documents/Light_microscopy_reporting_table.xlsx] to ensure the methods are described thoroughly. The table will be available to reviewers and ultimately published should the manuscript be accepted at the journal.

Finally, please ensure that you retain unprocessed data and metadata files after publication, ideally archiving data in perpetuity, as these may be requested during the peer review and production process or after publication if any issues arise.

EXTENDED DATA FIGURES
When re-submitting your manuscript, please ensure that any supplementary figures and tables that are crucial to the manuscript's conclusions are converted into Extended Data figures and tables to increase visibility of these data. Extended Data figures and tables are online-only (present in the online PDF and full-text HTML versions of the paper), peer-reviewed display items that provide essential background to the article but are not included in the main article due to space constraints. A maximum of ten Extended Data display items (figures and tables) is permitted.

DATA AVAILABILITY

We strongly encourage you to deposit all new data associated with the paper in a persistent repository where they can be freely and enduringly accessed. We recommend submitting the data to discipline-specific and community-recognized repositories; a list of repositories is provided here: http://www.nature.com/sdata/policies/repositories

All novel DNA and RNA sequencing data, protein sequences, genetic polymorphisms, linked genotype and phenotype data, gene expression data, macromolecular structures, and proteomics data must be deposited in a publicly accessible database, and accession codes and associated hyperlinks must be provided in the “Data Availability” section.

For papers containing bioimaging data, we strongly recommend depositing the data to Bioimage Archive (https://www.ebi.ac.uk/bioimage-archive/). Associated accession codes and hyperlinks should be provided in the "Data Availability" section.

Refer to our data policies here: https://www.nature.com/nature-research/editorial-policies/reporting-standards#availability-of-data

To further increase transparency, we encourage you to provide, in tabular form, the data underlying the graphical representations used in your figures. This is in addition to our data-deposition policy for specific types of experiments and large datasets. For readers, the source data will be made accessible directly from the figure legend. Spreadsheets can be submitted in .xls, .xlsx or .csv formats. Only one (1) file per figure is permitted: thus if there is a multi-paneled figure the source data for each panel should be clearly labeled in the csv/Excel file; alternately the data for a figure can be included in multiple, clearly labeled sheets in an Excel file. File sizes of up to 30 MB are permitted. When submitting source data files with your manuscript please select the Source Data file type and use the Title field in the File Description tab to indicate which figure the source data pertains to.

Please include a “Data availability” subsection in the Online Methods. This section should inform readers about the availability of the data used to support the conclusions of your study, including accession codes to public repositories, references to source data that may be published alongside the paper, unique identifiers such as URLs to data repository entries, or data set DOIs, and any other statement about data availability. At a minimum, you should include the following statement: “The data that support the findings of this study are available from the corresponding author upon request”, describing which data is available upon request and mentioning any restrictions on availability. If DOIs are provided, please include these in the Reference list (authors, title, publisher (repository name), identifier, year). For more guidance on how to write this section please see: http://www.nature.com/authors/policies/data/data-availability-statements-data-citations.pdf

CODE AVAILABILITY

Please include a “Code Availability” subsection in the Online Methods which details how your custom code is made available. Only in rare cases (where code is not central to the main conclusions of the paper) is the statement “available upon request” allowed (and reasons should be specified).

We request that you deposit code in a DOI-minting repository such as Zenodo, Gigantum or Code Ocean and cite the DOI in the Reference list. We also request that you use code versioning and provide a license.

For more information on our code sharing policy and requirements, please see: https://www.nature.com/nature-research/editorial-policies/reporting-standards#availability-of-computer-code

MATERIALS AVAILABILITY

As a condition of publication in Nature Methods, authors are required to make unique materials promptly available to others without undue qualifications.

Authors reporting new chemical compounds must provide chemical structure, synthesis and characterization details. Authors reporting mutant strains and cell lines are strongly encouraged to use established public repositories.

More details about our materials availability policy can be found at https://www.nature.com/nature-portfolio/editorial-policies/reporting-standards#availability-of-materials

ORCID

Nature Methods is committed to improving transparency in authorship. As part of our efforts in this direction, we are now requesting that all authors identified as ‘corresponding author’ on published papers create and link their Open Researcher and Contributor Identifier (ORCID) with their account on the Manuscript Tracking System (MTS), prior to acceptance. This applies to primary research papers only. ORCID helps the scientific community achieve unambiguous attribution of all scholarly contributions. You can create and link your ORCID from the home page of the MTS by clicking on ‘Modify my Springer Nature account’. For more information please visit please visit www.springernature.com/orcid.

Please do not hesitate to contact me if you have any questions or would like to discuss these revisions further. We look forward to seeing your revised manuscript and thank you for the opportunity to consider your work.

Best regards,
Nina

Nina Vogt, PhD
Senior Editor
Nature Methods

Reviewers' Comments:

Reviewer #1 (Remarks to the Author):

The authors have provided most answers to the questions raised during the initial review. Some points have now been thoroughly clarified, but a few questions remain unresolved. The main merit of this work is that it extends methodologies already developed by these authors to various sequencing-based spatial transcriptomics technologies. A clear presentation of the different approaches tested and a detailed description of the corresponding data, accompanied by links to access them, would facilitate future comparisons.
The presentation of the data is often unclear or insufficient. For example, all supplementary tables S10 through S14, then S16 through S20, and then S28 through S32 display genes and transcripts as ENSEMBL entries, without gene symbols, which makes any verification very tedious. A column with a genetic symbol should be added. The presentation of these tables should be improved so that they correspond more than the results generated at the end of a computational pipeline, but instead stand as autonomous sources of information. A more compact presentation of these many tables should also be considered.
Lines 372–373: The authors state that they identified 229 exons exhibiting sex-specific inclusion differences, the majority of which (154 exons) were detected in excitatory neurons (Fig. S21a). However, there is no further description of the results, and the figure is insufficient to evaluate them. Indeed, several transcripts also vary by region/cell, and there is a high risk that this finding is actually masked by other factors.

Minor comments:
Figure 2g is mentioned in the caption but does not appear in the figure.
Figure S21. The authors state that Pppp3ca has a sex-specific exon. They should include graphs comparing expression in males and females. The current Figure S21-e shows only the relative expression between Pppp3ca-201 and Pppp3ca-202.
Based on Figure S27a, a k value of 50 appeared more optimal. However, the authors retained a more conservative k value of 10, as they believed that optimization on the same data might lead to overfitting. In my opinion, it would also have been possible to use the settings from one experiment and apply them to another.
Figure S38 is poorly described and likely unnecessary.

Reviewer #2 (Remarks to the Author):

The authors have addressed all of my technical concerns.

Reviewer #3 (Remarks to the Author):

The authors have provided a clear and comprehensive response to the initial reviews, and my previous concerns have been largely addressed. The revised manuscript presents a robust and valuable technical framework.
Regarding my earlier points, I acknowledge the technical constraints involved in achieving high-resolution sub-clustering of excitatory neurons with the current modality and accept the authors' rationale. Furthermore, the identification of novel Gad2 isoforms is a particularly compelling addition. Such findings elegantly underscore the biological potential of this technology, as it captures subtle transcriptomic variations—often overlooked by traditional short-read sequencing—that may drive fundamental functional or evolutionary differences.
However, while these specific examples are excellent, the narrative remains predominantly case-driven. To maximize the utility of this work for the broader community, it would be highly beneficial to provide a more comprehensive, global perspective. I recommend adding a global summary or high level functional analysis to delineate the overall landscape of isoform differences across distinct brain regions and major cell types. For instance, a concise overview may highlight the functional categories, biological pathways, or alternative splicing events that are predominantly enriched in different spatial domains. It would also be valuable to clarify whether specific brain regions or cell types display greater isoform diversity, as this would provide important insights into the evolutionary forces and functional constraints that shape these regions or cell types.
Recognizing that this is fundamentally a methods paper, I do not expect an exhaustive downstream biological investigation or perfectly resolved mechanistic stories. The goal is simply to provide readers with a realistic "big picture" of the global biological variation captured by this technology. Providing this overarching context will serve as a valuable roadmap for future users. Pending this minor, computation-only addition, I am supportive of publication.

Reviewer #3 (Remarks on code availability):

The authors have provided a high-quality code repository. Notably, the documentation is comprehensive and provides a clear, step-by-step guide that ensures a smooth installation process. The tutorial is functional and error-free; the successful execution of all provided examples demonstrates the system's stability and ease of use for the broader community.

Version 2:

Decision Letter:

Our ref: NMETH-A62719B

1st Jun 2026

Dear Hagen,

Thank you for submitting your revised manuscript "Spatial isoform sequencing at sub-micrometer single-cell resolution reveals novel patterns of spatial isoform variability in brain cell types" (NMETH-A62719B). It has now been seen by the original referees and their comments are below. The reviewers find that the paper has improved in revision, and therefore we'll be happy in principle to publish it in Nature Methods, pending minor revisions to satisfy the referees' final requests and to comply with our editorial and formatting guidelines.

We are now performing detailed checks on your paper and will send you a checklist detailing our editorial and formatting requirements within two weeks or so. Please do not upload the final materials and make any revisions until you receive this additional information from us.

TRANSPARENT PEER REVIEW
Nature Methods offers a transparent peer review option for new original research manuscripts. We encourage increased transparency in peer review by publishing the reviewer comments, author rebuttal letters and editorial decision letters if the authors agree. Such peer review material is made available as a supplementary peer review file. **Please state in the cover letter ‘I wish to participate in transparent peer review’ if you want to opt in, or ‘I do not wish to participate in transparent peer review’ if you don’t.** Failure to state your preference will result in delays in accepting your manuscript for publication.

Please note: we allow redactions to authors’ rebuttal and reviewer comments in the interest of confidentiality. If you are concerned about the release of confidential data, please let us know specifically what information you would like to have removed. Please note that we cannot incorporate redactions for any other reasons. Reviewer names will be published in the peer review files if the reviewer signed the comments to authors, or if reviewers explicitly agree to release their name. For more information, please refer to our FAQ page.

ORCID
IMPORTANT: Non-corresponding authors do not have to link their ORCIDs but are encouraged to do so. Please note that it will not be possible to add/modify ORCIDs at proof. Thus, please let your co-authors know that if they wish to have their ORCID added to the paper they must follow the procedure described in the following link prior to acceptance:
https://www.springernature.com/gp/researchers/orcid/orcid-for-nature-research

Author names using non-Roman characters
Nature Portfolio journals can support presentation of author names using non-Roman characters in the HTML version of the article. If you wish to, please include author names in parentheses after the Roman-character spelling; see example online here. Currently supported scripts are: Arabic, Chinese, Cyrillic, Devanagari, Greek, Hebrew, Hangul, Japanese and Persian. You will be asked to verify the rendering is correct at proof stage.

Thank you again for your interest in Nature Methods. Please do not hesitate to contact me if you have any questions. We will be in touch again soon.

Best regards,
Nina

Nina Vogt, PhD
Senior Editor
Nature Methods

Reviewer #1 (Remarks to the Author):

The authors have now properly answered to my technical concerns.

Reviewer #3 (Remarks to the Author):

The authors have adequately responded to all my comments, and the revised manuscript shows clear improvements. I have no additional remarks.

Reviewer #3 (Remarks on code availability):

The code repository is well documented with clear installation steps. All tutorials and examples run reliably, showing good stability and usability.

Version 3:

Decision Letter:

22nd Jul 2026

Dear Hagen,

I am pleased to inform you that your Article, "Spatial isoform sequencing at single-cell resolution reveals cell-type-specific spatial isoform variability in multiple brain cell types", has now been accepted for publication in Nature Methods. The received and accepted dates will be September 11th, 2025 and July 22nd, 2026. This note is intended to let you know what to expect from us over the next month or so, and to let you know where to address any further questions.

Over the next few weeks, your paper will be copyedited to ensure that it conforms to Nature Methods style. Once your paper is typeset, you will receive an email with a link to choose the appropriate publishing options for your paper and our Author Services team will be in touch regarding any additional information that may be required. It is extremely important that you let us know now whether you will be difficult to contact over the next month. If this is the case, we ask that you send us the contact information (email, phone and fax) of someone who will be able to check the proofs and deal with any last-minute problems.

After the grant of rights is completed, you will receive a link to your electronic proof via email with a request to make any corrections within 48 hours. If, when you receive your proof, you cannot meet this deadline, please inform us at rjsproduction@springernature.com immediately.

Authors may need to take specific actions to achieve compliance with funder and institutional open access mandates. If your research is supported by a funder that requires immediate open access (e.g. according to Plan S principles or the NIH public access policy) then you should select the gold OA route, and we will direct you to the compliant route where possible. Because authors warrant under our subscription licensing terms that they haven’t committed to licensing any version of their article under a licence inconsistent with the terms of our agreement – including the applicable embargo period – publication under the subscription model isn’t suitable for authors whose funders require no embargo.

If you have any questions about our publishing options, costs, Open Access requirements, or our legal forms, please contact ASJournals@springernature.com

If you have posted a preprint on any preprint server, please ensure that the preprint details are updated with a publication reference, including the DOI and a URL to the published version of the article on the journal website.

You may wish to make your media relations office aware of your accepted publication, in case they consider it appropriate to organize some internal or external publicity. Once your paper has been scheduled you will receive an email confirming the publication details. This is normally 3-4 working days in advance of publication. If you need additional notice of the date and time of publication, please let the production team know when you receive the proof of your article to ensure there is sufficient time to coordinate. Further information on our embargo policies can be found here: https://www.nature.com/authors/policies/embargo.html

To assist our authors in disseminating their research to the broader community, our SharedIt initiative provides you with a unique shareable link that will allow anyone (with or without a subscription) to read the published article. Recipients of the link with a subscription will also be able to download and print the PDF.

As soon as your article is published, you will receive an automated email with your shareable link.

If you are active on Twitter/X or Bluesky, please e-mail me your and your coauthors’ handles so that we may tag you when the paper is published.

You can now use a single sign-on for all your accounts, view the status of all your manuscript submissions and reviews, access usage statistics for your published articles and download a record of your refereeing activity for the Nature journals.

Please note that you and any of your coauthors will be able to order reprints and single copies of the issue containing your article through Nature Portfolio's reprint website, which is located at http://www.nature.com/reprints/author-reprints.html. If there are any questions about reprints please send an email to author-reprints@nature.com and someone will assist you.

Please feel free to contact me if you have questions about any of these points.

Best regards,
Nina

Nina Vogt, PhD
Senior Editor
Nature Methods

** Visit the Springer Nature Editorial and Publishing website at www.springernature.com/editorial-and-publishing-jobs for more information about our career opportunities. If you have any questions please click here.**

Open Access This Peer Review File is licensed under a Creative Commons Attribution 4.0 International License, which permits use, sharing, adaptation, distribution and reproduction in any medium or format, as long as you give appropriate credit to the original author(s) and the source, provide a link to the Creative Commons license, and indicate if changes were made. In cases where reviewers are anonymous, credit should be given to 'Anonymous Referee' and the source.
The images or other third party material in this Peer Review File are included in the article's Creative Commons license, unless indicated otherwise in a credit line to the material. If material is not included in the article's Creative Commons license and your intended use is not permitted by statutory regulation or exceeds the permitted use, you will need to obtain permission directly from the copyright holder.
To view a copy of this license, visit https://creativecommons.org/licenses/by/4.0/

Reviewer #1 (Remarks to the Author):

A. Summary of the key results

The paper entitled “Spatial isoform sequencing at sub-micrometer single-cell resolution reveals novel patterns of spatial isoform variability in brain cell types” by Michielsen et al addresses the important question of spatial variability of expression of RNA isoforms. It applies to that end new methods to the latest generation of tools designed to perform sequence-based spatial transcriptomics. The paper is clearly written and emphasizes well the importance to develop such tools to better intercept the diversity of RNA isoform expression at single-cell and spatial resolution. The authors combined into a spatial transcriptomics framework one experimental and two computational tools: (1) Spl-ISO-Seq2 for the preparation of the libraries and sequencing of the samples, which is described with the use of Slide-SeqV2, although the authors also demonstrate at the end of their work its applicability to Visium HD 3’ long-read. Sequencing is done either on Oxford Nanopore or PacBio; (2) Spl-IsoQuant-2 is a tool for the genome-based analysis of spatial long RNA reads obtained with Spl-ISO-Seq2, also compatible with PacBio and Oxford Nanopore sequencings. Spl-IsoQuant performs barcode detection, read-to-isoform assignment, quantification and PCR deduplication. (3) Spl-IsoFind uses Moran’s I score, a global spatial autocorrelation score, to detect spatially variable isoforms in long-read spatial transcriptomics data.

We sincerely thank the reviewer for these kind words and the time and effort put into reviewing our manuscript. This constructive feedback helped us to improve the paper.

In summary, we made the following major changes to the manuscript:

- We compared our results with the spatial data from the previously published work (Lebrigand et al., 2023). Overall, this comparison shows that our data has very similar properties in terms of sequencing sensitivity and statistical power for the downstream analysis. We also applied our computational methods to data from Lebrigand et al. and demonstrated that the results are consistent with the ones obtained using our Spl-ISO-Seq2 data. However, Spl-ISO-Seq2 features a truly single-cell resolution thus allowing for investigating cell-type-specific effects.
- In addition to Stereo-seq and Visium HD, the new version of the developed Spl-IsoQuant-2 software now supports common single-cell and spatial protocols (e.g., 10x Visium 3’, 10x 3’ single-cell). We also developed a universal molecule description format that allows a user to process long-read data obtained with any single-cell/spatial protocol, thus making Spl-IsoQuant-2 a universal solution for long-read transcriptomic data.
- We generated and analyzed additional PacBio and Nanopore 10x Visium HD mouse brain data (with long-molecule selection and exome enrichment), which allow for a more direct comparison between Visium HD-derived and Stereo-seq-derived data. We also showed that the cost per informative read is substantially lower when long-molecule selection and exome enrichment are applied.
- We applied our computational methods to 10x single-cell data from Joglekar et al., 2024, and showed that sex differences in alternative splicing are detectable in single-cell long-read data.

Please also note that:

- "Sample 1 (4K)" is renamed to "Sample 1 (3.3K)" for a more accurate presentation of the results.
- All the analysis was redone with the updated software versions. Thus, the exact numbers may slightly differ from the previous version of the manuscript.

Below, we provide a more detailed point-by-point response to all comments.

B. Originality and significance: if not novel, please include reference
Tilgner's lab has already significantly contributed to the field of long read single cell transcriptomics, and this present paper details a new approach to identify differentially expressed RNA isoforms, based on Moran's I score, previously used in spatial biology. The article mentions its use to global gene expression (refs 34-36), but then develops its own method adapted for isoform detection, which is novel.

We thank the reviewer for acknowledging the novelty of our manuscript.

C. Data & methodology: validity of approach, quality of data, quality of presentation
Their approach based on Moran's I score uncovers patterns that were not previously captured by existing predefined-region comparisons. This approach allows them to identify the complex pattern of isoform expression of Tnnc1 in excitatory neurons. This approach leads to the observation that many spatial isoform signals are not only driven by different cell-type compositions.

In total, the work is of great quality, and paves the way for future biological investigations. Some conclusions remain at this time limited because the statistical power of the analysis is low (n=2). See for instance the huge error bars in Figures 4C, 5G, S5. Maybe a comparison with the existing literature could clarify somehow this point (compare for instance Figure 3A with Figure 4B from ref 19, in which different contrasts were reported between brain areas, which did not report a particular difference in midbrain). The authors should clarify these different points.

We appreciate the suggestion to compare our results to the existing literature, which is of course fundamental for any new approach. Specifically, the Lebrigand et al. publication is of high quality and therefore makes for a good comparison, despite some differences in procedure. **In short, we find very similar patterns, including distinct differences between midbrain and the hippocampus and cortex, in the Lebrigand et al. and our data.**

To enhance readability, we split our answer into three parts:

1. A direct comparison between Figure 3A in our manuscript and Figure 4B from Lebrigand et al.
2. Reanalysing the predefined regions for Lebrigand et al. using our pipeline and comparing these results to our results.
3. Applying Spl-IsoFind to Lebrigand et al. and comparing these results to our results.

1. A direct comparison between the reported results

In brief, our statistical approach and that of Lebrigand et al. have important differences. Therefore, directly comparing our Figure 3A to Figure 4B of Lebrigand et al. is not straightforward due to two main reasons:

1. We compared the brain regions at a higher anatomical level (e.g., Cortex vs. Hippocampus), while Lebrigand et al. compare subregions directly (e.g., Isocortex-1 vs. Dentate Gyrus).
2. The statistical test used to detect isoform-switching genes differs. Lebrigand et al. identify isoform-switching genes by testing for differentially expressed isoforms between regions. A gene is defined as isoform-switching if at least two isoforms are differentially expressed in opposite directions. While this is a very robust approach, it relies on the counts directly and might therefore miss some scenarios. For example, imagine a gene with two isoforms: isoform one is equally expressed in both regions, while isoform two is only expressed in one region. Since isoform two is only detected in one region, the gene clearly changes splicing mechanisms. However, the gene would not be classified as isoform-switching by Lebrigand et al., since the first isoform is not differentially expressed. Since we use a chi-square test, which models relative isoform usage, we would detect this change.

Nevertheless, despite the above differences, there are ample consistencies when comparing our Figure 3A to Lebrigand et al.'s Figure 4B. Indeed, focusing on the midbrain:

- Lebrigand et al.'s analysis shows the highest number of differentially expressed isoforms to the four subregions in the hippocampus (DG, Hippocampus area, CA1/2, CA3) and three cortical regions (Isocortex-1, Isocortex-2, Olfactory area).
- Consistent with the analysis of Lebrigand et al, we find that the midbrain has the highest number of differentially-spliced genes when compared to the hippocampus and when compared to the cortex.

These observations indicate that in Lebrigand et al's data, the midbrain is distinct from the hippocampus and cortex, just like in our data.

2. Comparing results after remapping and reanalysing Lebrigand et al.'s data (using pre-defined brain regions)

For a more equal comparison, we applied the same mapping and statistical approach to both datasets. We downloaded the FASTQ files from Lebrigand et al. from the SRA and applied Spl-IsoQuant-2. To harmonize the region annotation between our data and the Lebrigand et al. dataset, we mapped the subregions used by Lebrigand et al. to the broader regions used in our analysis (**Table S15**). Unfortunately, this mapping is imperfect. For example, we assigned the fiber tract spots to the white matter, but in Lebrigand et al.'s original dataset, this also includes some spots at the boundary of the midbrain and hippocampus. As such, we excluded white matter/fiber tracts from the comparison in this first part of the response.

Table S15. Mapping between the original and new labels for the Lebrigand et al. dataset.

**Original label**	**New label**
CA1/CA2, CA3, DG, Hippocampus area	Hippocampus
Iscortex-1, Isocortex-2, Olfactory area, Retrosplenial area,	Cortex
Fiber tracts *(only considered in later part of response)*	White Matter
Thalamus	Thalamus
Midbrain, Hypothalamus	Midbrain

Next, we applied our *n x 2* isoform tests to their relabeled spots. **The resulting heatmaps closely resemble our heatmap in Figure 3A (Response Figure R1)**. The exact numbers of significant genes vary, also between Lebrigand et al's two replicates, which have a similar read depth, but the three heatmaps show the same pattern: **Midbrain vs Cortex and Midbrain vs Hippocampus result in the most significant genes, while other comparisons yield fewer differences - in our approach and in the data of Lebrigand et al**.

Despite our approach having similarly high numbers of differentially spliced genes, the percentage of significant genes is lower in our combined Sample 1+2 (AE) data. Thus, our approach tests more genes but does not give more significant genes. Despite spending significant time investigating this discrepancy, we remain unsure regarding the causes, as the read depth is very similar in both samples.

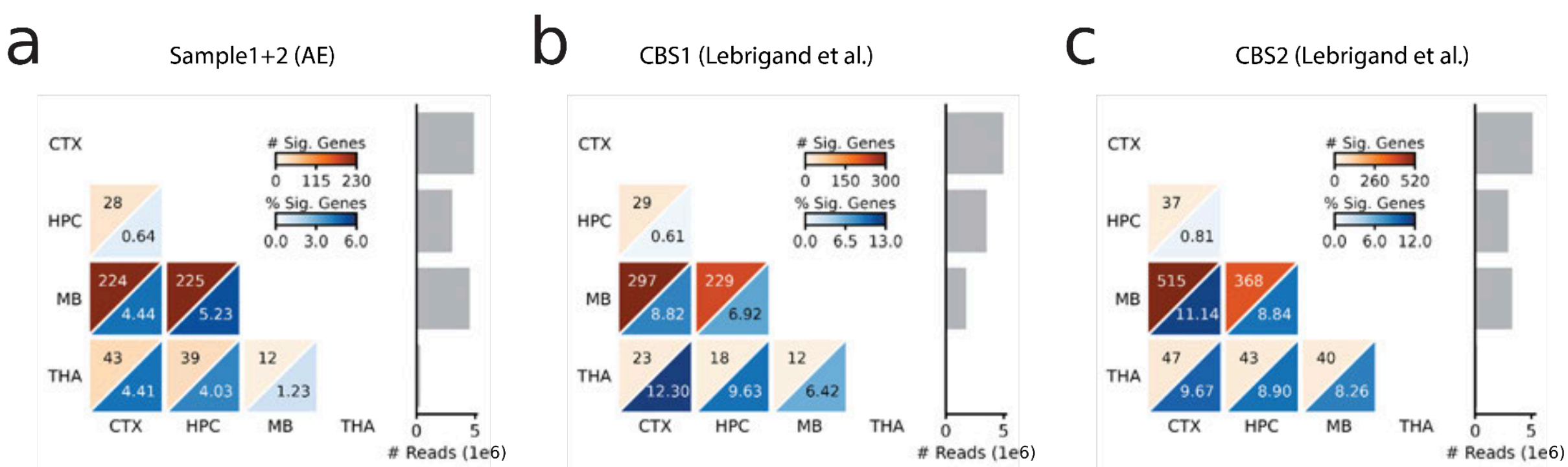

Response Figure R1. Number and percentage of genes changing isoform expression across predefined brain regions using all cells in **a)** combined Sample 1+2 (AE), **b)** Coronal Brain Section 1 (CBS1) from Lebrigand et al., and **c)** Coronal Brain Section 2 (CBS2) from Lebrigand et al.. The barplot on the right shows the number of long reads used during the analysis per brain region.

We observe stronger discrepancies when adding fiber tracts and white matter to the comparison. In CBS1 and CBS2, samples from Lebrigand et al., many differentially spliced genes are found in comparisons involving the fiber tracts. In our data, we observe fewer events in comparisons involving the white matter (**Response Figure R2**). We defined our regions based on anatomical locations, while they clustered their spots and assigned labels

to the spots. When redefining our white matter by clustering the data to include fiber tracts as well, we see a lot of significant genes as well. **The apparent discrepancy between white matter and fiber tracts is thus fundamentally a difference in how these regions were defined.**

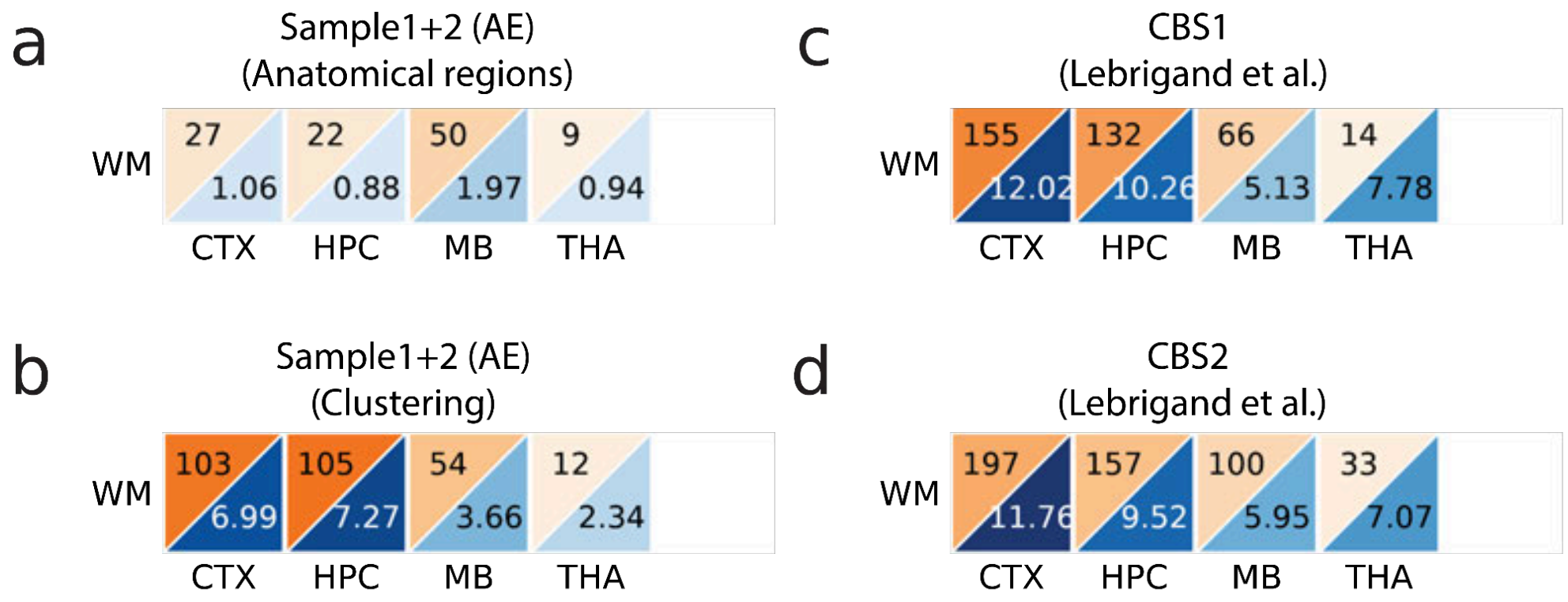

Response Figure R2. Number and percentage of genes changing isoform expression across predefined brain regions when comparing white matter to other brain regions in **a-b)** combined Sample 1+2 (AE) using regions defined based on **a)** anatomical locations and **b)** clustering the cells, **c)** Coronal Brain Section 1 (CBS1) from Lebrigand et al., and **d)** Coronal Brain Section 2 (CBS2) from Lebrigand et al..

3. Comparing Spl-IsoFind results between Lebrigand et al. and our data

The previous predefined region comparisons are an important tool to understand differences between protocols, but are not the main aim of our manuscript, which is our Spl-IsoFind analysis. For this, despite the limited sample size, we would like to emphasize that many of our key findings are consistently replicated across independent platforms (Stereo-seq and Visium HD) (**Fig 5h-i**). This cross-platform concordance supports the robustness of the detected spatial isoform patterns and suggests that the observed signals reflect genuine biological structure rather than sampling noise. **In our revised manuscript, we also ran Spl-IsoFind on Lebrigand et al's data and showed that it gave consistent results with our results.** We discovered 165 and 279 spatially variable isoforms (SVIs) in CBS1 and CBS2, respectively, from Lebrigand et al. In total, 3303 isoforms could be tested in both our combined S1+S2 (AE) data and the Lebrigand CBS1 and CBS2 samples (**Supplementary Figure S17**). The SVIs greatly overlap between the datasets, as is shown in the Venn diagram below.

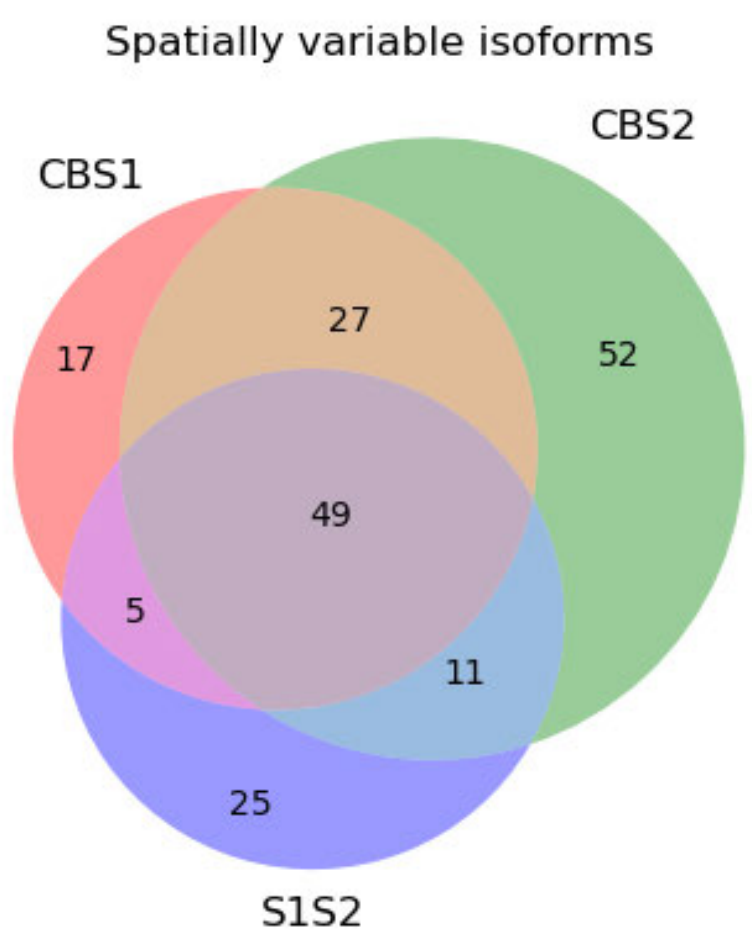

Supplementary Figure S17. Overlap between the detected SVIs in our combined Sample 1+2 (AE) data, CBS1, and CBS2 from Lebrigand et al.

Overall, while we acknowledge that larger sample sizes will be required to reduce uncertainty, these results demonstrate that both our predefined region analysis (**Fig 3**) and our Spl-IsoFind analysis (**Fig 4-5**) reveal broadly reproducible biological patterns.

Changes to the manuscript

- We added Supplementary Figure S12 and S17 and Supplementary Table S15.

- We added the following section to the Results:
  *A similar pattern appears in two previously published coronal brain sections[19] (see Methods) (**Fig S12**). However, in these data, we also detected many differences involving the white matter, which reflects imperfections in harmonizing regional annotations between studies.*

  *Besides strong agreement between the Spl-ISO-Seq2 samples, SVIs detected in the combined Sample1+2 (AE) data also greatly overlap with SVIs we discovered in the two previously published coronal brain sections[19] (**Fig S17**).*

- We added the following section to the Methods:
  Comparison to Visium coronal brain slides
  *We downloaded FASTQ files from the coronal brain slides published by Lebrigand et al.[19] from the Sequence Read Archive under accession numbers SRX8673336 and SRX8673337. We processed these reads using Spl-IsoQuant-2 and used ScisorSeqR as described earlier to detect genes that exhibit differential isoform usage. Since these datasets were annotated at a finer anatomical level, we mapped their original region annotation to the region annotations we used (**Table S15**). Finally, we detected spatially variable isoforms using Spl-IsoFind as described below.*

Moran's test can be affected by the coding of the neighborhood structure, which can greatly influence the observations. At which point the selected neighborhood method was adequate, and what can be the impact of such choices on the observed results?

We thank the reviewer for raising this important point. We agree that Moran's I depends on the definition of the neighborhood structure and that different choices of *k* can influence the detected spatial patterns. **Thus, *k* can be optimized, but we chose to use our original choice of *k* because optimization on the same data could cause overfitting. However, moving forward, readers can choose their *k* according to our supplemental analysis on their independent data.**

In more detail, we initially choose *k*=10 because this setting emphasizes the direct neighborhood of a cell and, as such, would be more sensitive to subtle or elongated spatial patterns. We acknowledge, however, that this choice was somewhat arbitrary. Therefore, we tested the impact of this parameter now. We ran Spl-IsoFind on the combined Sample1+2 (AE) data using a range of neighborhood sizes (**Fig S25a**). For each value of *k*, we set the minimum number of cells equal to 5**k*. Which implies that not all *k* can be tested for all isoforms.

We observed that the optimal number of *k* differs per cell type. For all cells combined and excitatory neurons, most spatially variable isoforms were detected using *k*=50, while for inhibitory neurons and astrocytes, *k*=100 seems most optimal. Most importantly, for most cell types, any number between *k*=10 and *k*=200 yielded broadly consistent results, with substantial overlap between the significant isoforms (**Fig S25b**). Specifically, 78 of 157 SVIs (49.7%) detected using *k*=10 were detected using all *k* between 10 and 200. Among the remaining 79 SVIs, 65 could not be tested using all *k* because of insufficient read depth.

Although these results suggest that *k*=10 might not be the optimal choice for Spl-IsoFind, we chose not to retrospectively adjust this parameter to avoid tuning it based on the observed results. Such post-hoc optimization would risk overfitting or double use of the data. Instead, we include this analysis in the main text so that readers can decide on the optimal *k* for their own datasets. We also added to the discussion that alternative neighborhood definitions, or strategies that combine results across multiple values of *k*, may further improve detection power.

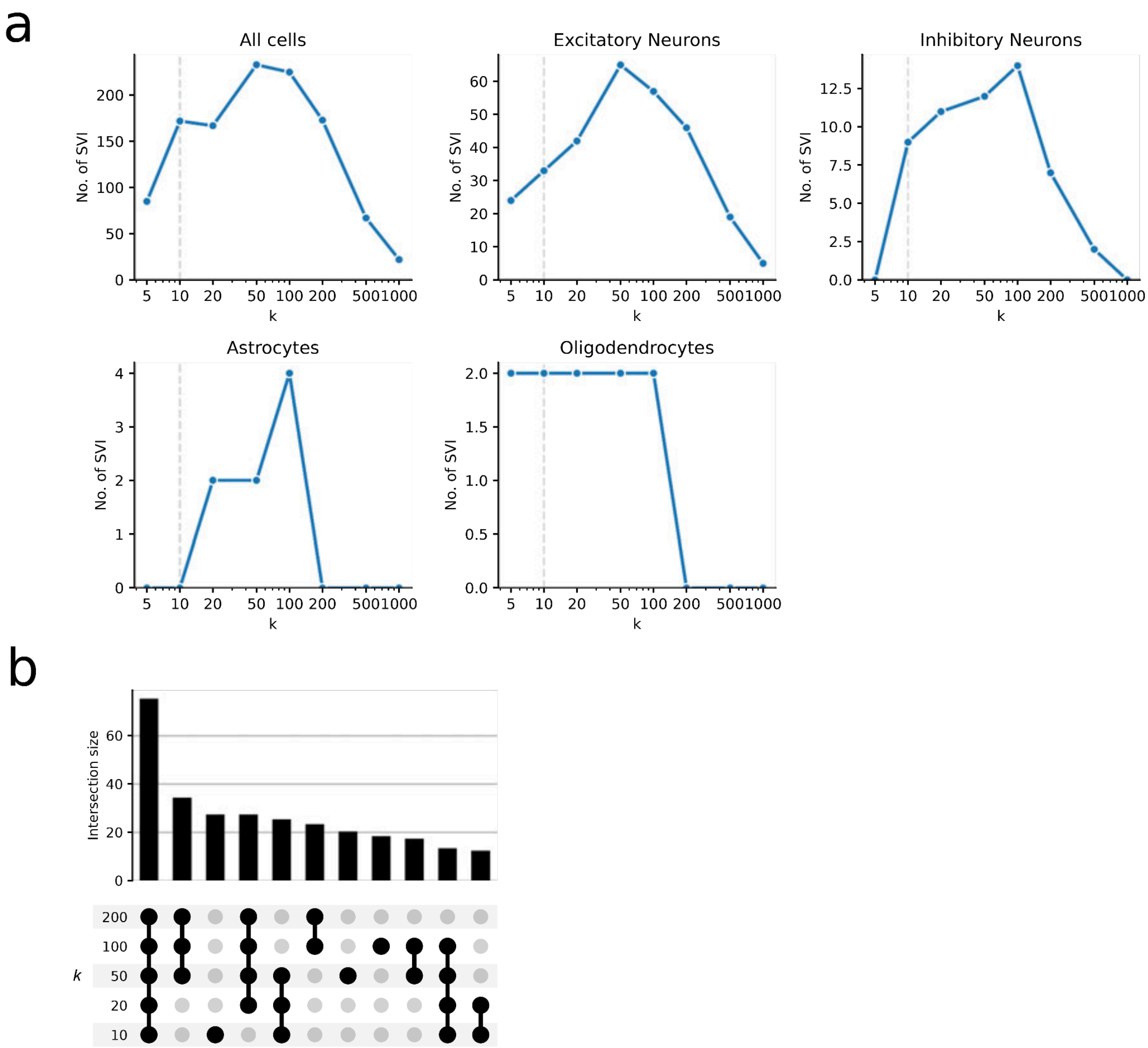

Figure S25 a) Number of significant SVIs detected by Spl-IsoFind using different numbers of nearest neighbors (*k*) when using all cells and for each cell type. **b)** The overlap in SVIs when using a different number of neighbors when using all cells.

Changes to the manuscript

- We added Supplementary Figure S25

- We added the following section to the Results:
  *During all experiments, we initially ran Spl-IsoFind using 10 nearest neighbors (k=10) as this emphasizes the direct neighborhood of a cell. We evaluated the effect of this parameter by using a range of neighborhood sizes on the combined Sample1+2 (AE) data. The optimal number of k differs per cell type: for all cells combined and excitatory neurons, most SVIs were detected using k=50, while for inhibitory neurons and oligodendrocytes, k=100 seems most optimal (**Fig S25a**). Most importantly, for most cell types, any number between k=10 and k=200 yielded broadly consistent results, with substantial overlap between the significant hits (**Fig S25b**). Specifically, 78 of 157 SVIs (49.7%) detected using k=10 were detected using all k between 10 and 200. Among the remaining 79 SVIs, 65 could not be tested using all k because of insufficient read depth. Although the selected neighborhood size (k=10) may be sub-optimal, it was maintained without retrospective adjustment to avoid overfitting.*

- We added the following section to the Discussion:
  In addition, optimizing the neighborhood size, or combining results across multiple neighborhood sizes to avoid relying on a single definition, may further increase detection power.

A current limitation of Spl-IsoFind is its low detection rate, which is not unique to the tool, and which can clearly improve by using a higher read depth, as evidenced by the comparison between Sample 1, sequenced with two ONT flow cells, and Sample 2, sequenced with only one. The authors could perhaps discuss what could be possible alternatives, either experimental or computational.

We agree with the reviewer that discussing how to improve the detection rate of Spl-IsoFind is informative for readers. A low read depth is not specific to Spl-ISO-Seq2, but reflects a more general challenge in spatial transcriptomics. Even though we applied exome enrichment and long-molecule selection to increase the number of informative reads for downstream analysis, the read depth remains limited.

1. Quantifying the effect of read depth

First, before discussing alternatives, we would like to discuss the effect of the read depth in more detail. To better quantify this limitation, we performed a downsampling analysis (**Supplementary Figure S16**). We evaluated the number of deduplicated reads, informative reads (spliced reads with an assigned isoform that overlap a segmented cell in the correct hemisphere), and detected spatially variable isoforms (SVIs). While deduplicated and informative reads do not seem fully saturated, the number of SVIs detected in all cells starts to saturate after ~100M sequenced reads for S1 (AE). In contrast, cell-type-specific SVIs do not yet appear to be saturated, suggesting that increasing the read depth would increase power. Combining consecutive slides, as done for the combined S1+S2 (AE) data, increases the number of detected SVIs even further, even for the cell-type-agnostic SVIs. However, this is a more expensive strategy.

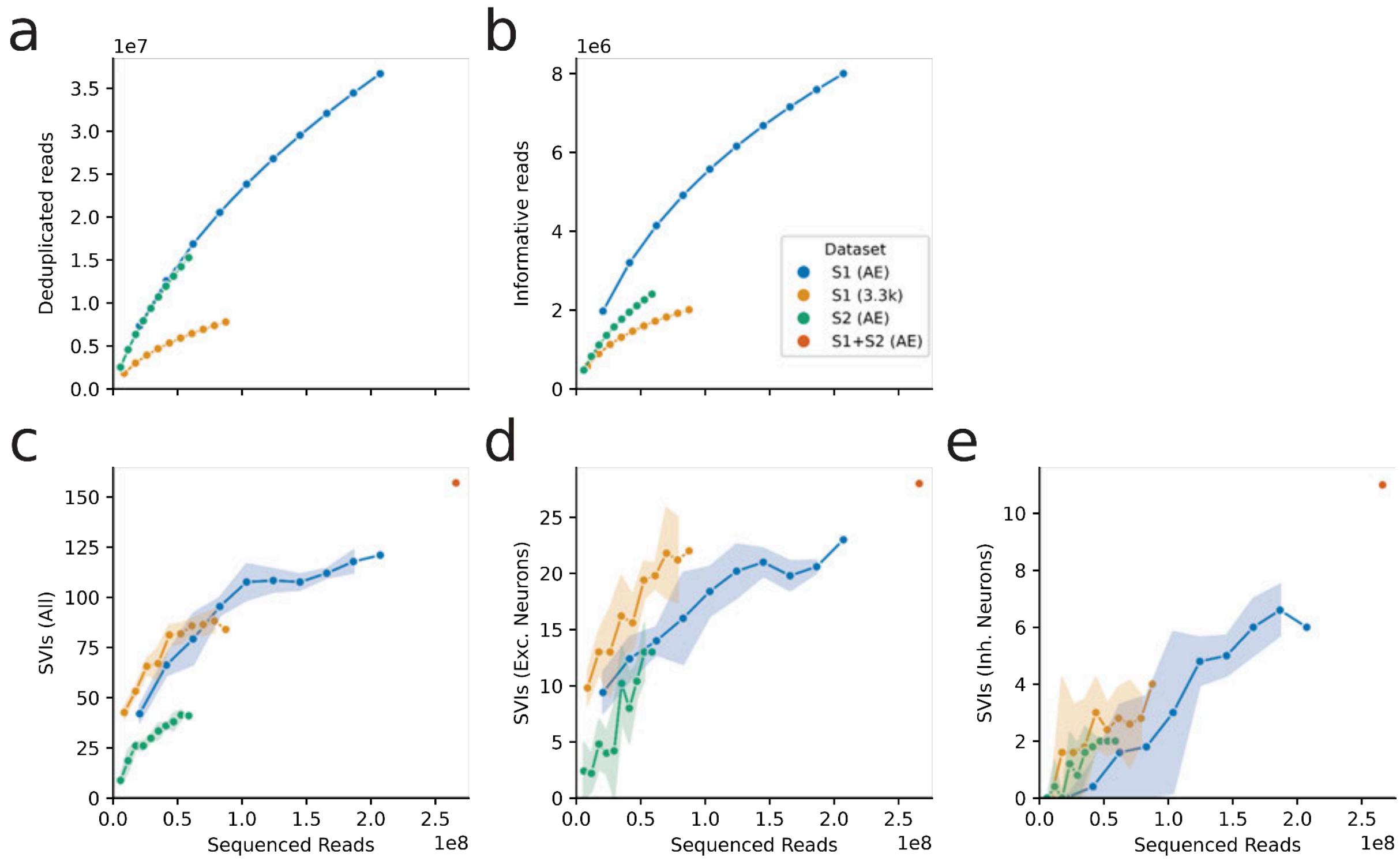

Figure S16. Saturation analysis for the Spl-ISO-Seq2 data. The plots show the number of downsampled sequenced reads vs. **a)** deduplicated reads, **b)** informative reads (spliced reads with an assigned isoform that overlap a segmented cell in the correct hemisphere), **c-e)** spatially variable isoforms (SVIs) detected in **c)** all cells, **d)** excitatory neurons, and **e)** inhibitory neurons. Lines show the mean across 10 downsampling experiments; shaded regions indicate ±1 standard deviation.

2. Imputing long-read sequencing data

From a computational perspective, one solution would be to impute long-read or isoform-level expression using a matched single-cell dataset as a reference. Several methods have been developed to impute short-read gene expression in spatial transcriptomics datasets using single-cell data as a reference (Lopez et al., 2019; Abdelaal et al., 2020; Biancalani et al., 2021). Similar strategies could be applied to long-read data. However, this would require careful validation since all current methods have been developed for short-read data. Furthermore, imputed values might introduce false positives during downstream analysis.

3. Optimizing the number of neighbors for Spl-IsoFind

Another computational strategy could be to optimize the number of neighbors used in Spl-IsoFind or to aggregate results across multiple neighborhoods. As shown in the previous answer, different neighborhoods result in a set of consistently detected SVIs, but some SVIs are unique for a certain *k*. Combining results across neighborhoods may further improve detection power.

We extended the paragraph in the discussion to address these experimental and computational avenues for improving the detection rate.

Changes to the manuscript:

- We added Figure S16

- We added the following text to the Results:
  *To better assess the effect of read depth, we performed a downsampling analysis (Methods) (**Fig S16**). While deduplicated and informative reads do not seem fully saturated, the number of SVIs detected in all cells starts to saturate at ~100M sequenced reads for Sample1 (AE). In contrast, cell-type-specific SVIs do not show clear saturation, suggesting that additional sequencing depth would increase detection power. Combining consecutive slides, as in the combined Sample1+2 (AE) dataset, increases the number of detected SVIs further, even for the cell-type-agnostic SVIs.*

- We added the following text to the Discussion:
  *A current limitation of Spl-IsoFind is its low detection rate, which is partially explained by read depth. For example, we discovered more than three times as many SVIs in Sample 1, where we used two ONT flow cells, compared to Sample 2, which was sequenced using only one. Besides sequencing depth, tissue placement affected the number of informative reads: in Sample 2, many reads mapped to the opposite hemisphere and were excluded from downstream analysis. We therefore recommend only sequencing the region of interest. In general, our saturation analysis showed that cell-type-agnostic SVIs begin to plateau at approximately one PromethION flow cell per coronal section, whereas cell-type-specific SVIs do not show clear saturation. We therefore recommend using one to two PromethION flow cells or PB SMRT cells, depending on whether the primary goal is global SVI discovery or cell-type-resolved analyses. Finally, for PB sequencing, library configuration strongly influences the number of sequenced and informative reads. Samples 1 and 2 were prepared using 16-fold MAS-Seq concatenation, whereas V1 used 12-fold concatenation (Kinnex). Because long-molecule selection produced relatively long molecules (**Fig. 5b**), 16-fold concatenation likely generated molecules exceeding optimal sequencing length and reduced efficiency. Matching the fold concatenation to read length, as in V1, substantially improved throughput.*
  Alternatively, the statistical power could be increased computationally by imputing isoform-level expression using matched single-cell data. Similar approaches have been developed for short-read spatial transcriptomics[68–70], although applying such methods would require careful validation to avoid false positives. In addition, optimizing the neighborhood size or combining results across multiple neighborhood sizes to avoid relying on a single definition may further increase detection power.

- We added the following section to the Methods:
  Saturation analysis
  For S1 (AE), S2 (AE), and S1 (3.3K), we randomly subsampled the sequenced reads to 10%, 20%,…, 90% of the total read count. We repeated each subsampling step ten times to account for stochastic variation.

The authors applied their approach to adult male mouse brain, thus revealing cell-type specific isoform changes between predefined regions and also in previously unrecognized spatial patterns.

The use of more resolutive representations like Slide-SeqV2 requires the capacity to read with a high precision many spatial barcodes. One strength of their approach is its capacity to quantify with a ghigh precision these sequences. One interest of their approach is thus to detect well all the different barcodes, even when less precise sequencer, i.e. Oxford Nanopore. This is an important aspect because this technology generates much more sequences than PacBio.

We thank the reviewer for appreciating our approach.

D. Appropriate use of statistics and treatment of uncertainties
A careful evaluation of the validity of the use of Moran's I test in Spl-IsoFind by a statistician is worth being made.

We discussed the validity of Spl-IsoFind with Dr. Wodan Ling, Assistant Professor in the Biostatistics Division in the Population Health Sciences Department at Weill Cornell Medicine. The only methodological consideration raised concerned the permutation strategy. In our implementation, the spatial graph is fixed and permutations are only performed over observed values (i.e., excluding all cells in which a gene is not expressed). To create an exact null distribution, all values, including missing entries, would have to be permuted. However, Dr. Ling pointed out that our current strategy is more conservative and more biologically meaningful, because the expression/non-expression of a gene is informative, which is taken into account by our approach.

E. Conclusions: robustness, validity, reliability
The proposed approach is of interest and the paper is well written. Its conclusions are appropriate.

We thank the reviewer for the positive evaluation.

F. Suggested improvements: experiments, data for possible revision
Their analysis of an adult mouse brain recapitulates well already known differential isoform abundance between known brain regions, and find some new events. Introduction of Spl-IsoFind allows to identify spatial isoform patterns outside predefined regions. The 2 independent approaches coincide in the identification of known switches, such as Rps24 in oligodendrocytes and Snap25. In that latter case, the authors were able to establish a possible origin of its variability in excitatory neurons. As expression of the two isoforms of SNAP-25 resulting from alternative splicing are possibly regulated by sex (see for instance Irfan, Sci Rep 9, 6403, 2019), the authors may document the differences between males (used in the present study) and females. This can greatly enlarge the list of differentially expressed isoforms.

We agree with the reviewer that investigating sex-specific regulation of isoform usage would be of great interest. However, performing a direct male–female comparison in the spatial long-read setting requires generating additional spatial samples (including at least two extra male and three female replicates), which was not possible given the time constraint and current financial strain on the science system in our country. However, we did perform a sex-specific analysis on single-cell long-read data (see below).

Nevertheless, we value the reviewer's suggestion on a sex comparison. To deliver such an analysis, we used the now enhanced capabilities of Spl-IsoQuant-2, which we engineered in response to another reviewer to deal with all barcoding strategies (10x 3' single-cell, spatial Slide-seqV2, Visium, Visium HD and Stereo-seq have all been tested). Thus, we investigated sex-specific splicing differences using an independent single-nucleus long-read dataset consisting of human hippocampal samples from three male and three female individuals (Joglekar et al., 2024). Although the sample size is limited and, especially for human data, inter-individual variability is expected, this dataset allows an initial exploration of sex-associated splicing patterns. We processed these data using the improved Spl-IsoQuant-2 and tested for sex-specific exon inclusion differences by aggregating reads per sex and performing exon-level comparisons within each cell type.

Exons were considered significant if they had an adjusted p-value < 0.05 (after Benjamini-Yekutieli correction) and an absolute $\Delta PSI > 0.1$ between males and females. To reduce the influence of outlier individuals, we additionally required that at least two individuals per sex had sufficient read coverage (≥ 10 reads) and that all individuals with sufficient coverage agreed on the direction of the effect. Using these criteria, we identified 229 exons with sex-specific inclusion differences, the majority of which were detected in excitatory neurons (154 exons), consistent with their higher read depth and known transcriptional diversity (**Supplementary Fig S21a**). **Thus, sex differences in alternative splicing are detectable in single-cell long-read data, and as the reviewer suggested, this will be an important topic for the future**.

Notably, one gene that we also identified as harboring spatially variable isoforms in our mouse brain spatial analysis, *Ppp3ca*, showed a sex-specific splicing difference as well (**Supplementary Fig S21b-c**). The same exon, which is conserved between mouse and human, distinguishing spatially variable isoforms, was differentially included between males and females. While our spatial data showed region-specific isoform patterns in male mouse cortex and hippocampus, with differences between the dentate gyrus (DG) and cornu ammonis (CA) regions, the single-nucleus data indicate that exon inclusion levels are higher in females in both DG and CA excitatory neurons (**Supplementary Fig S21d-e**). *PPP3CA* regulates synaptic and transcriptional signaling through presynaptic and postsynaptic dephosphorylation events and NFAT-mediated transcription, and plays a role in brain circuits involved in reward, memory, and aging (Yakel, 1997; Gerber et al., 2003). The sex-specific exon, which has higher inclusion in females, does not alter known protein domains but has been reported to modulate protein activity in differentiated myotubes (Brinegar et al., 2017).

For *SNAP25*, we did not find any sex-specific splicing differences in the human hippocampal data, in brief, because the human samples (28-40yrs old) were past development. *SNAP25*-202 is the main isoform during early development, and *SNAP25*-201 during adulthood. In the mouse hippocampus, this switch occurs later in females compared to males (Irfan et al., 2019). All human donors were 28-40 years old, and as such, the developmental switch has happened in both males and females already.

In summary, these results support the idea that spatial isoform regulation and sex-specific splicing can intersect, and they highlight the importance of incorporating sex as a biological variable in future spatial long-read studies. We have added this analysis and a discussion about the importance of studying sex-specific alternative splicing to the manuscript.

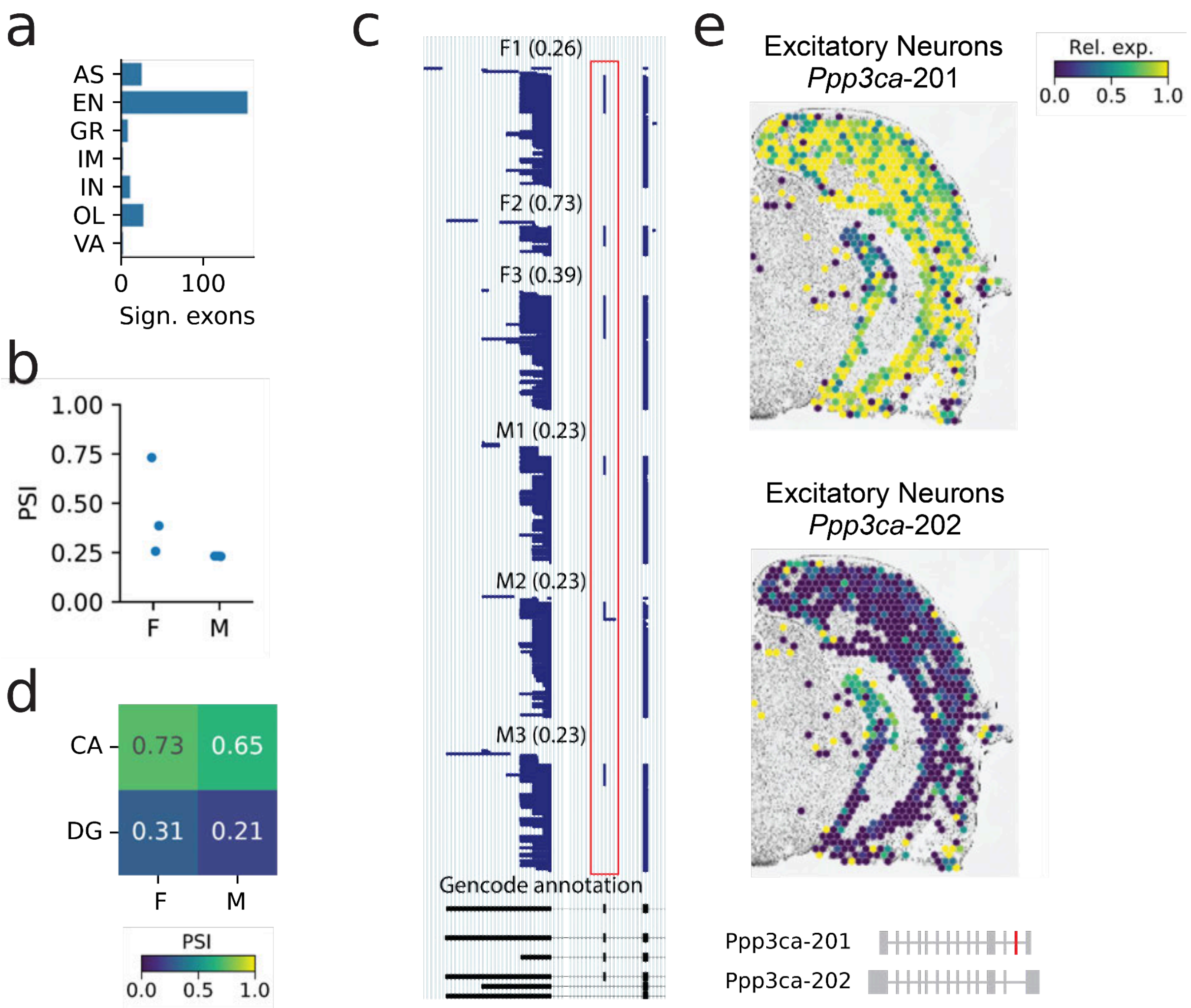

Figure S21 a) Number of significant sex-specific exons per cell type. **b)** PSI value for exon chr4_101029166_101029195_- in *PPP3CA* for each individual in excitatory neurons. **c)** Single-cell long-reads for *PPP3CA* measured in excitatory neurons per individual. Each line in the top six tracks represents a single cDNA molecule. The exon, chr4_101029166_101029195_-, in the red box is sex-specific. The bottom black track shows the Gencode annotation. The PSI value for each individual is indicated between brackets. **d)** PSI value for exon chr4_101029166_101029195_- in *PPP3CA* in excitatory neurons in the Dentate Gyrus (DG) and Cornu Ammonis (CA) for females (F) and males (M). **e)** Relative expression of *Ppp3ca*-201 and *Ppp3ca*-202 in excitatory neurons in Sample 1 (AE). Every hexagon shows the mean relative expression of the underlying cells. The ssDNA staining is shown in the background. The schematic diagram in the corner shows the gene structure of *Ppp3ca*-201 and *Ppp3ca*-202, the two most common isoforms in the data. The exon highlighted in red is the main difference between the two isoforms. This is the same exon that shows sex-specific inclusion differences, noting that PPP3CA is encoded on the positive strand in human and on the negative strand in mouse. AS = Astrocytes, EN = Excitatory Neurons, GR = Granule cells, IM = Immune cells, IN = Inhibitory Neurons, OL = Oligodendrocytes, VA = Vascular cells.

Changes to the manuscript:

- We added Figure S21

- We added the following text to the Results:

*Besides region- and cell-type-specific splicing, Snap25 exhibits sex- and development-specific splicing differences[56]. While Snap25-202 is the main isoform during early development, Snap25-201 is during adulthood. In the mouse hippocampus, this switch occurs later in females compared to males. As sex-specific splicing occurs in more genes[57,58], our discovered SVIs in the male mouse brain might thus not translate to females. Using human single-nuclei isoform RNA sequencing (SnISOr-Seq) data consisting of three female and three male hippocampal samples[9], we identified 229 exons with sex-specific inclusion differences, the majority of which were detected in excitatory neurons (154 exons) (**Fig S21a**). One gene, PPP3CA, showed both sex-specific splicing differences in human and has two SVIs in our mouse data (**Fig S21b-c**). The same exon, which is conserved between human and mouse, distinguished the SVIs and was differentially included between males and females. PPP3CA regulates synaptic and transcriptional signaling through presynaptic and postsynaptic dephosphorylation events and NFAT-mediated transcription, and plays a role in brain circuits involved in reward, memory, and aging[59,60]. The sex-specific exon, which has higher inclusion in females, does not alter known protein domains but has been reported to modulate protein activity in differentiated myotubes[61]. This exon shows spatial variation within the cortex and the hippocampus, with the biggest observed difference between the dentate gyrus (DG) and CA regions (**Fig S21e**). In the single-nucleus data, exon inclusion is higher in excitatory neurons in both brain regions in females (**Fig S21d**). SNAP25 did not show any sex-specific exon inclusion. All human donors were 28-40 years old, and as such, the developmental switch has happened in both females and males already.*

- We added the following Methods section:
  Sex-specific exon-inclusion analysis
  We investigated sex-specific splicing differences using a single-nucleus long-read dataset consisting of hippocampal samples from three males and three females[9]. We downloaded FASTQ files here: https://knowledge.brain-map.org/data/ASP3B09DZ8PXDUYSHDH. We processed these using Spl-IsoQuant-2 and tested for sex-specific exon inclusion differences by aggregating reads per sex and performing exon-level comparisons within each cell type (see differential exon testing using predefined comparisons). We used the cell-type labels defined in the original publication. Exons were considered significant if they passed a BY-adjusted p-value threshold of 0.05 and showed an absolute ΔPSI > 0.1 between males and females. To reduce the influence of outlier individuals, we additionally required that at least two out of three individuals per sex had sufficient read coverage (≥10 reads) and that all individuals with sufficient coverage agreed on the direction of the effect.

- We added the following to the Discussion:
  In addition to region- and cell-type-specific differences, several genes, including SNAP25, exhibit sex-dependent splicing[56–58], raising the possibility that SVIs identified in the male mouse brain may not fully translate to females. Using human single-nucleus long-read RNA sequencing data from three female and three male hippocampal samples, we identified 229 exons with sex-specific inclusion differences, predominantly in excitatory neurons. Although this analysis is limited by

small sample size and substantial inter-individual variability, it suggests that spatial and sex-dependent splicing regulation can intersect. These findings highlight sex as an important biological variable and underscore the need to study this in more detail.

Minor

• In Figure 3k, indicate scale + define more clearly the relative expression (for instance by using 2 distinct colors for the 2 Gria2 isoforms).

We apologize for lapsus. We believe the reviewer meant that panels in Figure 4 (i.e., panel i, j, and l) had a missing scale (while panel 3k had it). This is a clear oversight of ours and we have corrected it.

Regarding the representation of relative expression, *Gria2*-201 and *Gria2*-202 are the two most highly expressed isoforms in the combined Sample 1+2 (AE) data. Hence, we added these two isoforms to the schematic in panel 3k. However, a previously unannotated isoform (*Gria2*-new) is also expressed in these samples. A yellow spot means that *Gria2*-201 is expressed, while a dark blue spot means that *Gria2*-201 is not expressed at that location, but either *Gria2*-202 or *Gria2*-new is expressed. We have adjusted the caption of Figure 3 to clarify this. Also, we added a supplementary figure showing the expression of *Gria2*-202 and *Gria2*-new separately (**Supplementary Fig S9-10**).

Changes to the manuscript

- We added Supplementary Figure S9

- We added color bars to panels i,j, and l in Figure 4

- We added the following sentence to the caption of Figure 3
  As such, a yellow spot means that Gria2-201 is expressed, while a dark blue spot means that Gria2-201 is not expressed at that location, but another Gria2 isoform is expressed.

• It is explained that the Visium HD long-read data was generated without long-molecule and exome selection, resulting in a shorter overall read length and a lower percentage of spliced reads compared to Spl-ISO-Seq2. However, 7.8M have an isoform assigned vs 8.0M in Sample 1 (AE)) even though four ONT flow cells were used compared to two flow cells for Sample 1 (AE). This seems to be a very small difference. Please explain.

We apologize to the reviewer that this was not clear. We intended not to emphasize the absolute difference in informative reads (spliced reads with an isoform assigned) but to **highlight the difference in long-read sequencing efficiency and cost required to generate a similar number of informative reads**.

Even though the total number of informative reads is comparable between the Visium HD long-read dataset (now called V2 in the manuscript) and Sample 1 (AE), the sequencing effort required to obtain these reads differs considerably. While four flow cells were needed for V2, only two flow cells were used for Sample 1 (AE), which results in a lower cost per informative reads (**Supplementary Table S27**).

In the current manuscript, we added new Visium HD datasets (with long-molecule selection and exome enrichment), which allow for a more direct comparison. V1, which was generated with long-molecule selection and exome enrichment, was sequenced on a single flow cell and yielded 8.4M informative reads. In contrast, V2, which was naively sequenced, was sequenced using four flow cells and yielded 7.2M informative reads.

In summary, **although the absolute number of isoform-assigned reads appears similar, the number of flow cells required to obtain these reads differs considerably**. Consequently, the cost per informative read is substantially lower when long-molecule selection and exome enrichment are applied.

Table S27 Estimated total cost per experiment and cost per sequencing output. Costs are reported per 1M total reads and per 100K informative reads. Informative reads are spliced reads with an assigned isoform overlapping a segmented cell in the correct hemisphere and thus are suitable for downstream analysis. Estimates are calculated for the complete workflow and for the long-read sequencing part alone.

	Including array prep			Long-read sequencing only			
	Est. total cost	Cost per 1M seq. reads	Cost per 100K informative reads	Est. total cost	Cost per 1M seq. reads	Cost per 100K informative reads	
S1 (AE) - ONT	$7,390	$36	$92	$2,390	$12	$30	
S1 (AE) - PB	$7,365	$368	$323	$2,365	$118	$104	S1 (AE) - PB was sequenced using PacBio Mas-Seq with 16-fold concatenation resulting in fewer reads compared to V1 - PB, which was sequencing with the Kinnex kit with 12-fold concatenation
S1 (3.3K) - ONT	$6,700	$76	$325	$1,700	$19	$82	
S2 (AE) - ONT	$6,350	$108	$264	$1,350	$23	$56	For S2 (AE) - ONT, many reads were mapped to cells in the other hemisphere that we discarded. This largely accounts for the difference compared to S1 (AE) - ONT
S2 (AE) - PB	$7,365	$409	$550	$2,365	$131	$177	S2 (AE) - PB was sequenced using PacBio Mas-Seq with 16-fold concatenation resulting in fewer reads compared to V1 - PB, which was sequencing with the Kinnex kit with 12-fold concatenation
V1 - ONT	$5,850	$59	$70	$1,350	$14	$16	

V1 - PB	$6,865	$54	$98	$2,365	$19	$34	
V2 - ONT	$8,780	$15	$122	$4,280	$7	$60	In comparison to V1-ONT, this did not use exome enrichment and long-molecule selection. As a result, it needed more flow cells, raising the cost
V3 - PB	$11,775	$28	$93	$7,275	$17	$58	In comparison to V1-PB, this did not use exome enrichment and long-molecule selection. As a result, it needed more flow cells, raising the cost

Changes to the manuscript

- We added Table S26-27

- We added the following sentence to the Results section:
  *For both ONT and PacBio, applying long-molecule selection and exome enrichment thus greatly reduced the sequencing cost per informative read (**Table S26-27**).*

• 500 ng of the cDNA collected from the previous step was used as input for the exome enrichment, which represents a standard amount for exome capture, but a huge amount in term of cDNA at the end of a single-cell experiment. Do the authors use a particular procedure to perform larger amplification? Can it introduce biases in their measurements?

We appreciate the question from the reviewer; this question provides an opportunity to remedy the missing explanations we previously had in our manuscript. We performed linear/asymmetric amplification (see Hardwick et al, Nat Biotechnol, 2022) on the cDNA aliquoted for long-read experiments with the purpose of appending additional base pairs to the 3' end of reads in order to avoid any cDNAs with secondary structure or double-TSO structure. More details can be found in the method section "Linear PCR of cDNA", which was first described as part of our previously published method SnISOr-Seq (Hardwick et al, Nature Biotech, 2022). SnISOr-Seq, which consists of linear/asymmetric PCR (LAP) and exome enrichment (CAP), was designed to improve the single-nuclei long-read isoform sequencing efficiency by enriching for barcode/UMI-containing cDNA and excluding pure intronic molecules. In the SnISOr-Seq paper, we proved that the gene expression is highly correlated between two conditions: naive cDNA (pre-LAPCAP) and cDNA derived from LAP-CAP (post-LAPCAP). The figure, of which panel D is of particular interest, has been attached below and can also be found at https://www.nature.com/articles/s41587-022-01231-3/figures/2. In summary, it suggests that the similar procedures conducted in this study would not introduce any obvious bias at the transcriptional level.

[redacted]

G. References: appropriate credit to previous work?
Appropriate

H. Clarity and context: lucidity of abstract/summary, appropriateness of abstract, introduction and conclusions
Appropriate

We thank the reviewer for the thorough and helpful feedback.

Reviewer #2 (Remarks to the Author):

Summary of Paper

In this study, the authors adapt their previously developed Spl-ISO-Seq long-read spatial transcriptomics method to the higher-resolution Stereo-Seq platform. They combine the platform's sub-micrometer barcoding chemistry with their established exome enrichment and long-molecule selection workflow to generate full-length, spatially barcoded cDNAs from mouse brain sections. The resulting cDNAs are sequenced using both Oxford Nanopore and PacBio technologies. To process these data, they introduce Spl-IsoQuant-2, a tweaked version of their exsiting software to adapt barcode-parsing and isoform-assignment algorithm to Stereo-Seq's more complex read structure, and Spl-IsoFind, a software tool that identifies spatially variable isoforms using Moran's I statistics, a metric commonly used to find varying patterns of gene expression in spatial transcriptomics . Applying this pipeline, the authors report isoform-level spatial expression maps at near-single-cell resolution, identify examples of spatially variable isoforms such as Snap25 and Rps24, and show that some of these patterns cannot be explained by cell-type composition alone. Finally, they apply their software to publicly available Visium HD long-read data to demonstrate cross-platform compatibility and reproducibility.

Methodological Innovation and Biological Impact

Overall, I find the novel the methods presented here to represent very modest changes to either their (quite good) existing algorithms (Spl-IsoQuant-2 versus the Spl-ISO-Seq pipeline) or adaptation of widely used metrics in spatial transcriptomics for alternative splicing (Moran-I statistic). The biological findings are largely anecdotal and not well-benchmarked. Thus, both the methodological innovation and biological impact are low.

The experimental and computational workflows build directly on the authors' prior Spl-ISO-Seq publications (Nat Commun 2021; NAR 2023; Nat Biotech 2023; and especially Foord, Nat Commun 2025), which combined spatial barcoding with long-read sequencing for isoform-level analysis. The present study largely extends that approach to the higher-resolution Stereo-Seq platform, accompanied by minor software updates (Spl-IsoQuant-2 and Spl-IsoFind). While the adaptation is technically competent and the integration of long-read spatial data at near-single-cell resolution is valuable, the advance is primarily in adapting their existing code rather than a conceptually new algorithmic or experimental approach. The improvement in spatial resolution enables better cellular resolution, but this comes from leveraging an existing commercial platform. Overall, this work represents an update to the author's already published method rather than a fundamentally new paradigm, and thus the innovation here is at best moderate.

One of the two main technical innovations in this paper is Spl-IsoQuant-2, an extension of the authors' IsoQuant pipeline. This component is technically well executed, but the innovation is primarily using standard “old-school” methods (i.e. k-mer matching followed by local alignment) to handle the noiser barcode reads inherent to Oxford Nanopore. In fact, the barcode parsing advances touted as new are already described in their previous work (On p. 5-6 Foord et al uses k-mer indexing followed by Smith Waterman!). Thus, the conceptual novelty for this method over their own previous method is essentially zero. The second

innovation is Spl-IsoFind, which identifies spatially variable isoforms using Moran's I, a standard measure of spatial autocorrelation. While this is a sensible extension of existing spatial-omics algorithms to isoform data, it represents an application of a well-established statistic rather than a new analytical framework.

The biological insights derived from the study are also limited. Most examples (for instance Snap25 and Rps24) validate known patterns of isoform variation rather than revealing new mechanisms or cell-state relationships. The authors do not convincingly show that the information gained could not be obtained more directly from single-cell or single-nucleus long-read sequencing combined with anatomical annotation. The biological findings here seem mostly anecdotal. While Nature Methods is focused on methods development, I feel the best way to highlight the utility of a new method is to describe some new biology that would inspire other users to adapt the method. This aspect of the paper – exciting the biologist with the potential of the new types of analyses now possible was lacking.

Overall, it is important that the community have good workflows to ingrate long read sequencing with spatial transcriptomics. But this workflow and protocol presented are not substantially different than what has already been published by this group nor are there any new biological insights provided beyond purely descriptive anecdotes.

We thank the reviewer for highlighting our unclear presentation of innovation in our manuscript and for the time and effort put into the revision. While the algorithmic base of Spl-IsoQuant-2 is indeed inherited from previous work, there are several important innovations that we implemented in the software.

Firstly, we adapted the algorithm for **extremely large barcode whitelists containing up to 500 million barcodes**. Our current implementation allows us to index half a billion Stereo-seq barcodes in less than 10 minutes using 16 threads (instead of 20 hours with the previous algorithm) using 120GB of RAM (instead of 500GB+ of RAM for the previous implementation). Barcode detection allows processing roughly 60 million reads per hour without deconcatenation, or 10 million reads per hour with deconcatenation, in 16 threads, making it applicable even to very large spatial datasets. Unfortunately, as there are no other tools for Stereo-seq barcode detection, the only software we could compare to is Flexiplex, which detected barcodes in only 2% of 1 million reads after running for 5 days. **Thus, Spl-IsoQuant2 enables a utility that Spl-IsoQuant and other tools could not.**

Furthermore, in response to the reviewer's comment, we have reengineered Spl-IsoQuant-2 so that it can take into account user-specified molecule structures - meaning it will be applicable to most future protocols. We implemented a molecule description format that allows a user to describe their own molecule structure and perform barcode calling for any custom protocol. While Flexiplex allows specifying custom adapter sequences, Spl-IsoQuant-2 allows fully describing a custom molecule structure, including sophisticated cases with multiple barcodes (such as 10x Visium HD) or barcodes separated by a linker sequence (such as for Curio Biosciences' Slide-seqV2 spatial protocol). Additionally, we have dedicated, specialized versions for Stereo-seq, 10x 3' single-cell, 10x Visium, 10x Visium HD, and Curio Biosciences' Slide-seqV2.

Last but not least, **we would like to point out that, especially for Stereo-seq, in contrast to other spatial transcriptomic technologies (which utilize different cDNA structures that are less likely to concatenate), the deconcatenation strategy was of high importance and not previously implemented**. This not only increases the number of detected barcoded cDNAs by ~10%, but also prevents false positive barcode-isoform pairs in which the barcode originating from cDNA1 would be matched to the isoform of cDNA2.

Thus, Spl-IsoQuant-2 presents not a simply adapted version of the previously published software, but a **universal and efficient solution for existing common single-cell and spatial protocols, as well as presents novel functionality that allows researchers to perform data processing for custom protocols without implementing new software**.

Additionally, we'd like to point out that our experimental approach was not simply combining Stereo-seq-derived cDNA with long-read sequencing. **We employed an experimental optimization that limited the formation of the above-mentioned concatenated cDNA** molecules with a linear PCR, followed by exome enrichment and long-molecule selection. Although exome enrichment and long-molecule selection were previously introduced in Foord, Prjibelski, Hu, et al. 2025, this was further optimized by performing a linear-PCR in order to add a sequence to the barcoded end of the cDNA, such that the two ends of the molecules differed. This helped to prevent (although not entirely eradicate) molecule concatenation in further experimental steps.

Furthermore, regarding Spl-IsoFind, we would like to highlight that we not only adjusted Moran's I for isoform data, but also **implemented a permutation strategy using cell-type-constrained permutations**. This allows us to distinguish between the three models that can explain alternative splicing across brain regions: 1) multiple cell types show distinct spatial splicing patterns, 2) one cell type shows a spatial splicing pattern, 3) no cell type directly shows a spatial splicing pattern, but the cell type composition differs between regions. **To our knowledge, a similar permutation approach has not been implemented for gene expression data.**

In summary, our current manuscript presents the following algorithmic and experimental procedures:

- Spl-ISO-Seq2 is optimized to limit cDNA concatenations;
- Spl-IsoQuant-2 efficiently indexes extremely long barcode whitelists;
- Spl-IsoQuant-2 can be applied to existing common single-cell and spatial protocols;
- Spl-IsoQuant-2 can be applied to custom protocols without implementing new software;
- Spl-IsoFind implements a novel permutation strategy, distinguishing between distinct causes underlying spatial variation patterns.

Besides the improvements mentioned above, during this revision we also made the following major changes to the manuscript:

- We compared our results with the spatial data from the previously published work (Lebrigand et al., 2023). Overall, this comparison shows that our data has very similar properties in terms of sequencing sensitivity and statistical power for the downstream analysis. We also applied our computational methods to data from Lebrigand et al. and demonstrated that the results are consistent with the ones

obtained using our Spl-ISO-Seq2 data. However, Spl-ISO-Seq2 features a truly single-cell resolution thus allowing for investigating cell-type-specific effects.
- We generated and analyzed additional PacBio and Nanopore 10x Visium HD mouse brain data (with long-molecule selection and exome enrichment), which allow for a more direct comparison between Visium HD-derived and Stereo-seq-derived data. We also showed that the cost per informative read is substantially lower when long-molecule selection and exome enrichment are applied.

Among the minor changes, please also note that:
- "Sample 1 (4K)" is renamed to "Sample 1 (3.3K)" for a more accurate presentation of the results.
- All the analysis was redone with the updated software versions. Thus, the exact numbers may slightly differ from the previous version of the manuscript.

Below, we provide a more detailed point-by-point response to all comments.

Minor Comments and Suggested Revisions
1. Per-cell QC metrics (UMIs/reads/genes per cell) are missing. One of the main advantages of this workflow over previous work is the opportunity to achieve single cell resolution. But basic single-cell–level QC metrics are omitted. For example, median and mean UMIs per segmented cell, genes detected per cell, isoforms per cell, and the fraction of reads assigned per cell. The supplement reports "mean reads per gene/isoform" and various global tallies, but not per-cell coverage, which makes it hard to judge sensitivity and to compare platforms.

We apologize that this information was missing from the manuscript. **In brief, we added these statistics to the supplement and compared our sensitivity to other long-read spatial transcriptomics datasets, which show comparable results (while we added single-cell resolution).**

1.1 We now added these statistics to the supplementary material. We added **Table S1** now to include per-cell QC metrics for the short-read Stereo-seq data, which include the number of segmented cells, mean and median number of barcodes per cell, mean and median number of UMIs per cell, and mean and median number of genes per cell. For the long-read datasets, we extended **Supplementary Tables S7, S8, S25** to include the mean and median number of spliced reads per cell, percentage of spliced reads assigned to a gene per cell, mean and median number of genes per cell, percentage of spliced reads assigned to an isoform per cell, and mean and median number of isoforms detected per cell.

1.2 We compared our sequencing statistics to another long-read spatial transcriptomics dataset in the mouse brain (which, in contrast to ours, does not have single-cell resolution). To our knowledge, our current study presents the first long-read spatial transcriptomics dataset at a single-cell resolution. As such, we cannot directly compare these per-cell QC metrics to other long-read spatial studies. As an alternative, we compared overall sequencing statistics to closely related work, which also sequenced two coronal mouse brain sections using Nanopore, but relied on the Visium technology with 55μm (implying no single-cell resolution) to generate the cDNA (Lebrigand et al., 2023). They sequenced two coronal sections (CBS1 and CBS2) using three and eight flow cells,

resulting in 174M and 286M reads, respectively. **This is thus very comparable to the number of sequenced reads for our Sample 1 (AE) (207M).**

To ensure a fair comparison, we reprocessed this data using our tool Spl-IsoQuant-2. However, for the Lebrigand et al. data, a subset, containing 44M and 99M reads, respectively, was uploaded to SRA. The uploaded data likely represent reads already enriched for informative molecules, as the number of reads after UMI deduplication is consistent with those reported in their Supplementary Information. When comparing the informative reads (UMI-deduplicated, spliced reads with an assigned isoform and overlapping a segmented cell or spot in the correct hemisphere), we observe a comparable number of informative reads (~8M) between Sample 1 (AE) and CBS1 and CBS2 (**Supplementary Table S9**). **This similar number of informative reads indicates that the overall sensitivity is comparable, while Spl-ISO-Seq2 additionally enables a single-cell resolution.**

1.3 We equally provided UMI and barcode statistics for a newly added 10x Visium HD sample and compared its sensitivity to the Stereo-seq-derived observations. Of note, our algorithms (see above) work for all these platforms. In this revised version of the manuscript, we added new Visium HD datasets, which we sequenced with long-molecule selection and exome enrichment. This sample, V1, was sequenced on a single PromenthION flow cell and a single PacBio SMRT cell, resulting in 8.4M and 7.0M informative reads, respectively. The number of informative reads is again comparable to that of Sample 1 (AE) and the Lebrigand et al. Visium data. However, for the Visium HD data, fewer sequenced reads were needed (99-127M), indicating a slightly higher sensitivity (**Supplementary Table S9**). However, the resolution of Visium HD is slightly lower compared to Stereo-seq (2µm compared to 500nm). The higher sensitivity could be explained by the absence of gaps between the barcoded spots on the Visium HD slide, which is the case for the Stereo-seq and Visium platforms.

Together, the newly added per-cell QC metrics and this contextual comparison clarify both the sensitivity and resolution of our approach and facilitate comparison across platforms.

Table S9. A comparison of read statistics between samples generated using the Stereo-seq, Visium HD, and Visium platforms. Informative reads are defined as UMI-deduplicated, spliced reads with an assigned isoform and overlapping a segmented cell or spot in the correct hemisphere. However, for the Lebrigand et al paper, a subset, containing 44M and 99M reads, respectively, was uploaded to SRA. The uploaded data likely represent reads already enriched for informative molecules, as the number of reads after UMI deduplication is consistent with those reported in their Supplementary Materials.

	Stereo-seq Sample 1 (AE - ONT)	Visium HD V1 (ONT)	Visium HD V1 (PacBio)	Visium CBS1	Visium CBS2
Sequenced reads	207,282,773	98,713,075	126,023,947	173,996,000	286,458,673
Analysed reads	207,282,773	98,713,075	126,023,947	43,899,878	98,892,086
Barcode detected	130,360,866	49,089,229	84,196,089	36,625,448	80,219,056
Uniquely assigned	100,571,599	38,561,039	66,968,834	31,032,886	67,505,940
Uniquely assigned and spliced	79,030,635	25,987,614	53,617,739	19,972,628	48,768,593
Reads after deduplication	36,728,127	22,313,578	18,616,621	17,581,867	18,489,755
Spliced reads after deduplication	25,916,277	14,740,254	13,620,397	10,929,678	12,189,522
Informative reads	8,006,816	8,354,635	6,982,558	8,204,667	8,749,685

Changes to the manuscript

- We added Tables S1 and S9 and extended Tables S7, S8, and S25.

- We added the following to the Results section:
  *We compared the number of sequenced reads required to obtain a given number of informative reads (spliced reads with an assigned isoform that overlap a segmented cell in the correct hemisphere) to a similar long-read spatial transcriptomics dataset but generated on the Visium platform[19] (Methods). Both platforms show a comparable sensitivity as a similar number of sequenced reads (~200M) yields a comparable number of informative reads (~8M) (**Table S9**). Importantly, Spl-ISO-Seq2 additionally provides single-cell resolution.*

2. Cost, throughput, and depth required for power. It would be helpful if the authors provided a clear cost table for one mouse coronal section: array prep, enrichment, ONT/PacBio runs, and expected depth to reach power for detecting spatially variable isoforms (SVIs) at given effect sizes. Include practical guidance on how many flow cells are needed for typical tissues, since Sample 1 used more ONT depth than Sample 2 and yielded substantially more events

We thank the reviewer for guiding us towards making this work more accessible and useful for readers. **In summary, we recommend (a) performing exome enrichment with long-molecule selection and (b) using one-to-two complete PromethION flow cells or one-to-two PacBio SMRT cells per sample. Furthermore, we advise only putting the**

region of interest on the slide to avoid losing reads to regions not of interest. We added a section to the Discussion to advise readers and discuss it in more detail below.

2.1 We performed a saturation analysis supporting the "1-to-2 flowcells per sample" recommendation (<u>after exome enrichment</u>).

To know what power is needed for detecting spatially variable isoforms, we performed a saturation analysis (**Supplementary Figure S18**). We downsampled the number of sequenced reads and evaluated the number of deduplicated reads, informative reads (spliced reads with an assigned isoform and overlapping a segmented cell in the correct hemisphere), and detected spatially variable isoforms (SVIs). In this saturation analysis, the number of deduplicated and informative reads slowly starts to saturate. Interestingly, for downstream processes (i.e., detecting spatially variable isoforms with Spl-IsoFind), the results are mixed. When we combined all cells to detect cell-type-agnostic SVIs, the number of SVIs seems to saturate for Sample 1 (AE) at 50% of the sequenced reads (which would relate to one flow cell). **For this cell-type-agnostic level of analysis, a single flowcell is therefore enough.** For cell-type-specific analysis (excitatory and inhibitory neurons), the results don't seem saturated yet. Here, we greatly benefit from adding extra read depth. **For this cell-type-specific analysis, a second flowcell is very informative**.

Also interestingly, for the same read depth, more neuron-specific SVIs are detected in Sample 1 (3.3K), which was expected, as many neuronal genes are among the targeted genes.

Finally, although Sample 1 (AE) and Sample 2 (AE) yield a similar number of deduplicated reads for comparable sequencing depth, the number of informative reads is lower in Sample 2 (AE). This is mainly explained by the large fraction of reads mapping to the other, incomplete hemisphere, which was excluded from downstream analysis. **To maximize sequencing efficiency, we therefore recommend placing only the region of interest on the slide to avoid wasting sequenced reads.**

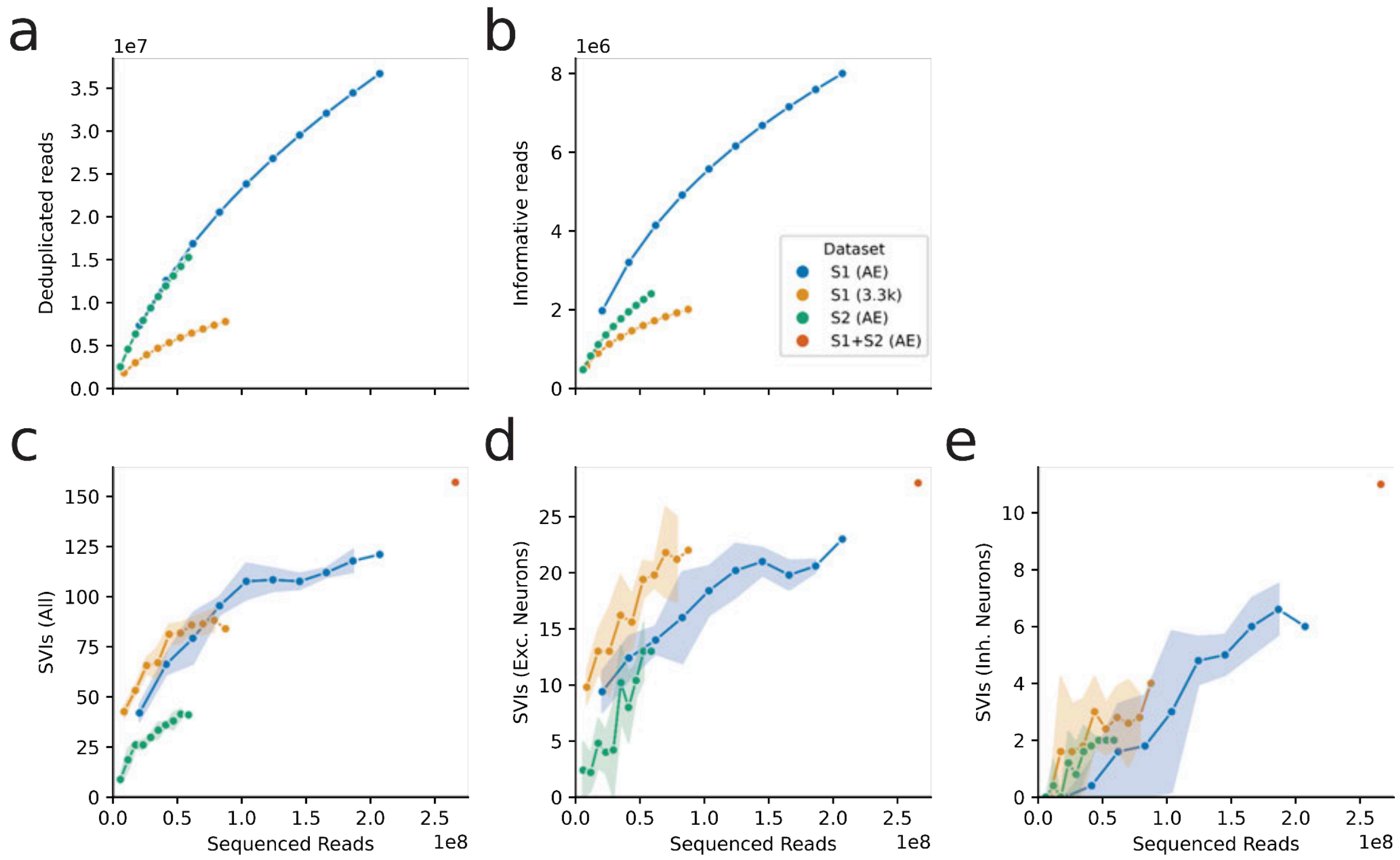

Figure S18. Saturation analysis for the Spl-ISO-Seq2 data. The plots show the number of downsampled sequenced reads vs. **a)** deduplicated reads, **b)** informative reads (spliced reads with an isoform assigned overlapping a segmented cell in the correct hemisphere), **c-e)** spatially variable isoforms (SVIs) detected in **c)** all cells, **d)** excitatory neurons, and **e)** inhibitory neurons. Lines show the mean across 10 downsampling experiments; shaded regions indicate ±1 standard deviation.

2.2 We have added a cost table including the total cost per experiment and cost per informative read (Supplementary Tables S26-27).

We added a cost table including all Stereo-seq-derived and Visium HD-derived datasets analyzed in the manuscript. We report the cost per 1M sequenced reads and per 100K informative reads (i.e., reads usable for downstream analysis), with the latter being the more relevant metric. Based on this table, we want to highlight some interesting observations:

- V1-ONT, generated using long-molecule selection and exome enrichment, was sequenced on a single flow cell and yielded 8.4M informative reads. In contrast, V2-ONT, which was sequenced without enrichment, required four flow cells and yielded only 7.2M informative reads, resulting in a substantially higher cost per informative read.
- For PacBio sequencing, V1-PB produced considerably more total reads than Sample 1 (AE) - PB and Sample 2 (AE) - PB, thereby considerably reducing both the cost per sequenced read and the cost per informative read. Samples 1 and 2 were sequenced using MAS-Seq with 16-fold concatenation, whereas V1 was sequenced using the Kinnex kit with 12-fold concatenation. Given the relatively long read length after long-molecule selection (**Fig. 5b**), 16-fold concatenation likely generated molecules that exceeded optimal sequencing length, thereby reducing yield. The 12-fold concatenation used for V1 appears better suited for our read length distribution.

- The cost per informative read for Sample 2 (AE) is considerably higher because a large fraction of reads mapped to cells in the opposite hemisphere, which were excluded from downstream analysis.

Overall, this highlights that exome enrichment and long-molecule selection, optimal concatenation for PacBio, and only sequencing the region of interest reduce the cost per informative read.

Table S26. Estimated costs for the individual steps during each experiment. Per type of experiment we also indicated which steps were included. The total cost per experiment is shown in Table S27. Shared steps (e.g., the Stereo-seq slide) are included for multiple experiments, even when the same slide was used in practice to get a fair estimate of the cost per read (e.g., as in Sample 1 (AE) and Sample 1 (3.3K)). The reported costs are an estimate and may vary across institutes depending on available infrastructure and vendor discounts.

	Est. cost	S1/S2 (AE) ONT	S1/S2 (AE) PB	S1 (3.3K) ONT	V1 ONT	V1 PB	V2 ONT	V3 PB
Stereo-seq slide (including SR seq.)	$5000	1x	1x	1x				
Visium HD slide (excluding SR seq.)	$4500				1x	1x	1x	1x
Long-mol. selection	$35	1x	1x	1x	1x	1x		
Exome enrichment	$155	1x	1x	1x	1x	1x		
3378 gene enrichment	$350			1x				
ONT library prep	$120	1x		1x	1x		1x	
PromethION flow Cell	$1040	1-2x		1x	1x		4x	
PacBio library prep	$475		1x			1x		1x
PacBio SMRT Cell	$1700		1x			1x		4x

Table S27. Estimated total cost per experiment and cost per sequencing output. Costs are reported per 1M total reads and per 100K informative reads. Informative reads are spliced reads with an assigned isoform overlapping a segmented cell in the correct hemisphere and thus suitable for downstream analysis. Estimates are calculated for the complete workflow and for the long-read sequencing part alone.

	Including array prep			Long-read sequencing only			
	Est. total cost	Cost per 1M seq. reads	Cost per 100K informative reads	Est. total cost	Cost per 1M seq. reads	Cost per 100K informative reads	
S1 (AE) - ONT	$7,390	$36	$92	$2,390	$12	$30	
S1 (AE) - PB	$7,365	$368	$323	$2,365	$118	$104	S1 (AE) - PB was sequenced using PacBio Mas-Seq with 16-fold concatenation resulting in fewer reads compared to V1 - PB, which was sequencing with the Kinnex kit with 12-fold concatenation
S1 (3.3K) - ONT	$6,700	$76	$325	$1,700	$19	$82	
S2 (AE) - ONT	$6,350	$108	$264	$1,350	$23	$56	For S2 (AE) - ONT, many reads were mapped to cells in the other hemisphere that we discarded. This largely accounts for the difference compared to S1 (AE) - ONT
S2 (AE) - PB	$7,365	$409	$550	$2,365	$131	$177	S2 (AE) - PB was sequenced using PacBio Mas-Seq with 16-fold concatenation resulting in fewer reads compared to V1 - PB, which was sequencing with the Kinnex kit with 12-fold concatenation
V1 - ONT	$5,850	$59	$70	$1,350	$14	$16	
V1 - PB	$6,865	$54	$98	$2,365	$19	$34	
V2 - ONT	$8,780	$15	$122	$4,280	$7	$60	In comparison to V1-ONT, this did not use exome enrichment and long-molecule selection. As a result, it needed more flow cells, raising the cost
V3 - PB	$11,775	$28	$93	$7,275	$17	$58	In comparison to V1-PB, this did not use exome enrichment and long-molecule selection. As a result, it needed more flow cells, raising the cost

Changes to the manuscript:

- We added Table S26-27 and Figure S18

- We added the following text to the Results:
  *To better assess the effect of read depth, we performed a downsampling analysis (Methods) (**Fig S18**). While deduplicated and informative reads do not seem fully saturated, the number of SVIs detected in all cells starts to saturate at ~100M sequenced reads for Sample1 (AE). In contrast, cell-type-specific SVIs do not show clear saturation, suggesting that additional sequencing depth would increase detection power. Combining consecutive slides, as in the combined Sample1+2 (AE) dataset, increases the number of detected SVIs further, even for the cell-type-agnostic SVIs.*

  *For both ONT and PacBio, applying long-molecule selection and exome enrichment thus greatly reduced the sequencing cost per informative read (**Table S26-27**).*

- We added the following section to the Methods:
  Saturation analysis
  For S1 (AE), S2 (AE), and S1 (3.3K), we randomly subsampled the sequenced reads to 10%, 20%,…, 90% of the total read count. We repeated each subsampling step ten times to account for stochastic variation.

- We added the following to the Discussion:
  *A current limitation of Spl-IsoFind is its low detection rate, which is partially explained by read depth. For example, we discovered more than three times as many SVIs in Sample 1, where we used two ONT flow cells, compared to Sample 2, which was sequenced using only one. Besides sequencing depth, tissue placement affected the number of informative reads: in Sample 2, many reads mapped to the opposite hemisphere and were excluded from downstream analysis. We therefore recommend only sequencing the region of interest. In general, our saturation analysis showed that cell-type-agnostic SVIs begin to plateau at approximately one PromethION flow cell per coronal section, whereas cell-type-specific SVIs do not show clear saturation. We therefore recommend using one to two PromethION flow cells or PB SMRT cells, depending on whether the primary goal is global SVI discovery or cell-type-resolved analyses. Finally, for PB sequencing, library configuration strongly influences the number of sequenced and informative reads. Samples 1 and 2 were prepared using 16-fold MAS-Seq concatenation, whereas V1 used 12-fold concatenation (Kinnex). Because long-molecule selection produced relatively long molecules (**Fig. 5b**), 16-fold concatenation likely generated molecules exceeding optimal sequencing length and reduced efficiency. Matching the fold concatenation to read length, as in V1, substantially improved throughput.*

3. Cell assignment and segmentation details. The Methods should spell out exactly how capture barcodes are mapped to segmented nuclei, thresholds for singlet calls, and doublet exclusion criteria. Right now the text notes ssDNA nuclear staining and that only RCTD singlets are used, but it lacks quantitative thresholds and error rates. Please add: number of spots assigned per cell, distance rules, and an empirical doublet rate estimate.

We apologize as these important details were incomplete. We will elaborate on each of these points below and have added these details to the Methods section. Also, we have added a Supplementary Table to include the mean and median number of barcodes per cell for the Stereo-seq-derived data (**Supplementary Table S1**) and the new Visium HD-derived data (**Supplementary Table S21**)

3.1 Cell segmentation and barcode-to-cell assignment
We used the SAW pipeline (version 7) for cell segmentation. For nucleus segmentation, SAW version 7 relies on a built-in deep-learning model trained on nucleus-stained images to segment nuclei from the ssDNA staining. After segmentation, cell boundaries are estimated by expanding each nucleus by up to 10 barcodes (corresponding to 5 µm) or until the expanded boundaries of neighboring cells collide.

This pipeline results in a masking file indicating the cell boundaries, a barcode-to-position mapping file, and a Gene Expression File (GEF) containing the CellID for each (x, y) coordinate. However, this standard SAW-generated GEF only includes coordinates with at least one short-read assigned. If we were to use this file directly, we would lose coordinates that contain long reads but no short reads.

To ensure that all coordinates inside a segmented cell were assigned a CellID, we generated an artificial GEM (gene expression matrix) with one gene expressed for each (x, y) coordinate. Using the `saw convert bin2cell` function and the previously obtained masking file, we converted this artificial GEM file into a new cell-binned GEF. The resulting GEF contains CellID assignments for all coordinates inside a cell.

3.2 Cell-type assignments
We used RCTD (v2.2.1) to assign cell types to the segmented cells and identify singlets and doublets. We decided to use RCTD for this task as it is the top-performing method in multiple benchmarking studies (Li et al., 2022; Chen et al., 2022; Li et al., 2023; Sang-aram et al., 2024). We ran RCTD in ‘doublet’ mode, as recommended by the authors for datasets with fine spatial resolution, allowing each segmented cell to be modeled as consisting of either one or two cell types. The details of the RCTD algorithm in ‘doublet’ mode and thresholds used are described in more detail in the ‘Cell type identification by model selection’ section of the original RCTD publication (Cable et al, 2022).

We added a Supplementary Figure showing the percentage and spatial location of singlets and doublets (**Supplementary Figure S2, S31-33**). In the Stereo-seq-derived data, 54.3% of the cells in Sample 1 and 62.3% in Sample 2 were classified as singlets. These fractions are lower than estimates reported based on Xenium data (~80%, Tiesmeyer et al., 2025). However, we used segmented cells during our analysis, while Tiesmeyer et al. focused on the segmented nucleus. Regardless, we decided to keep the threshold as is, since we prefer a conservative filtering strategy to minimize the number of false positives, which we will elaborate on in our response to point 5. In the Visium HD samples, 39.4%, 27.5% and 50.4% of segmented cells or binned spots were annotated as a singlet respectively for V1, V2, and V3. This lower fraction is primarily due to the use of long-read data for deconvolution, which resulted in insufficient read depth for RCTD in a large proportion of the cells/bins (27.1%, 55.5%, 29.3%).

Changes to the manuscript

- We added Tables S1 and S21 and Figures S2 and S31-33

- We rewrote the 'cell segmentation and barcode to cell assignment' Methods section for clarity:
  Cells were segmented based on the ssDNA staining and short-read gene expression using the SAW[76] pipeline (version 7). For nucleus segmentation, SAW version 7 relies on a built-in deep-learning model trained on nucleus-stained images to segment nuclei from the ssDNA staining. After segmentation, cell boundaries are estimated by expanding each nucleus by up to 10 barcodes (corresponding to 5 μm) or until the expanded boundaries of neighboring cells collide. This generates several outputs including a masking image indicating the cell boundaries, a barcode-to-position mapping file, and a GEF (gene expression file) that contains the CellID for each x and y coordinate combination. However, this GEF only includes coordinates that have at least one short-read assigned. If we used this file directly to map coordinates (and thus barcodes) to the cells, we would lose the coordinates that only have long-reads but no short-reads assigned. To ensure that all coordinates inside a segmented cell were assigned a CellID, we generated an artificial GEM (gene expression matrix) with one short-read for each x and y coordinate. Using the `saw convert bin2cell` function and the previously obtained masking file, we converted this artificial GEM into a new cell-binned GEF. This new GEF includes the CellID for all coordinates inside a cell.

- We rewrote the 'Cell-type identification' Methods section for clarity:
  We used Robust Cell Type Decomposition (RCTD) (V2.2.1)[37] to assign cell types to segmented cells and identify singlets and doublets. For the Spl-ISO-Seq2 datasets, we input gene expression matrices obtained from the short-read sequencing data. For the Visium HD datasets, short-read data were unavailable, so we constructed gene expression matrices using the long reads. We used a reference[9] file including six P56 mouse single-cell samples from multiple brain regions including hippocampus, visual cortex, striatum, and thalamus. Previously annotated "doublets" from the single-cell data were removed prior to analysis. We ran RCTD in "doublet" mode allowing each segmented cell to be modeled as consisting of either one or two cell types. We ran RCTD with default parameters following published tutorials (https://github.com/dmcable/spacexr/blob/master/vignettes/spatial-transcriptomics.Rmd). Spots designated as "singlets" were assigned as cell types per sample.

4. The paper would be improved with benchmarking (or even discussion) for expected numbers of differentially utilized isoforms in gold standard datasets. They identify 1-4% of DE genes had differential isoform use. Is that similar to what is known in their prior work? Single cell or bulk approaches? Is this because their sensitivity is low?

We appreciate the suggestion and added a comparison to existing literature for the predefined region analysis and the Spl-IsoFind analysis. Specifically, the previously mentioned datasets from Lebrigand et al. make an interesting comparison. They sequenced two coronal mouse brain sections using Nanopore on cDNA derived from the Visium technology with 55μm (implying no single-cell resolution) (Lebrigand et al., 2023). **This**

comparison demonstrates that both our predefined region analysis and our Spl-IsoFind analysis reveal broadly reproducible biological patterns.

4.1. Comparing the predefined region analysis

We analysed the Lebrigand et al. data using our predefined region analysis using our *n* x 2 isoform tests and compared the results to our data. We had to harmonize the region annotation between our data and the Lebrigand et al. dataset. We mapped the subregions used by Lebrigand et al. to the broader regions used in our analysis (**Supplementary Table S15**). Unfortunately, this mapping is imperfect. For example, we assigned the fiber tract spots to the white matter, but in Lebrigand et al.'s data this also includes some spots at the boundary of the midbrain and hippocampus. As such, we excluded white matter/fiber tracts from the comparison in this first part of the response.

Supplementary Table S15. Mapping between the original and new labels for the Lebrigand et al. dataset.

**Original label**	**New label**
CA1/CA2, CA3, DG, Hippocampus area	Hippocampus
Iscortex-1, Isocortex-2, Olfactory area, Retrosplenial area,	Cortex
Fiber tracts *(only considered in the later part of the response)*	White Matter
Thalamus	Thalamus
Midbrain, Hypothalamus	Midbrain

Next, we applied our *n x 2* isoform tests to their relabeled spots. **The resulting heatmaps closely resemble our heatmap in Figure 3A (Response Figure R1)**. The exact numbers of significant genes vary, also between Lebrigand et al's two replicates, which have a similar read depth, but the three heatmaps show the same pattern: **Midbrain vs Cortex and Midbrain vs Hippocampus result in the most significant genes, while other comparisons yield fewer differences - in our approach and in the data of Lebrigand et al**.

Despite our approach having similarly high numbers of differentially spliced genes, the percentage of significant genes is lower in our combined Sample 1+2 (AE) data. Thus, our approach tests more genes but does not give more significant genes. Despite spending significant time investigating this discrepancy, we remain unsure regarding the causes, as the read depth is very similar in both samples.

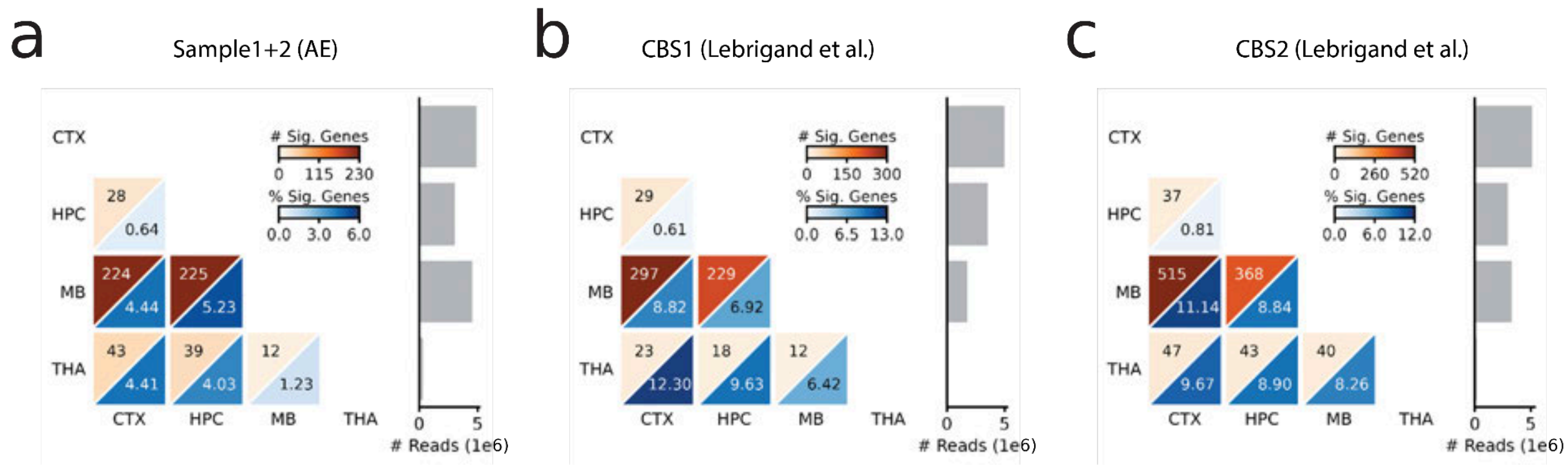

Response Figure R1. Number and percentage of genes changing isoform expression across predefined brain regions using all cells in **a)** combined Sample 1+2 (AE), **b)** Coronal Brain Section 1 (CBS1) from Lebrigand et al., and **c)** Coronal Brain Section 2 (CBS2) from Lebrigand et al.. The barplot on the right shows the number of long reads used during the analysis per brain region.

We observe stronger discrepancies when adding fiber tracts and white matter to the comparison. In CBS1 and CBS2, samples from Lebrigand et al., many differentially spliced genes are found in comparisons involving the fiber tracts. In our data, we observe fewer events in comparisons involving the white matter (**Response Figure R2**). We defined our regions based on anatomical locations, while they clustered their spots and assigned labels to the spots. When redefining our white matter by clustering the data to include fiber tracts as well, we see a lot of significant genes as well. **The apparent discrepancy between white matter and fiber tracts is thus fundamentally a difference in how these regions were defined.**

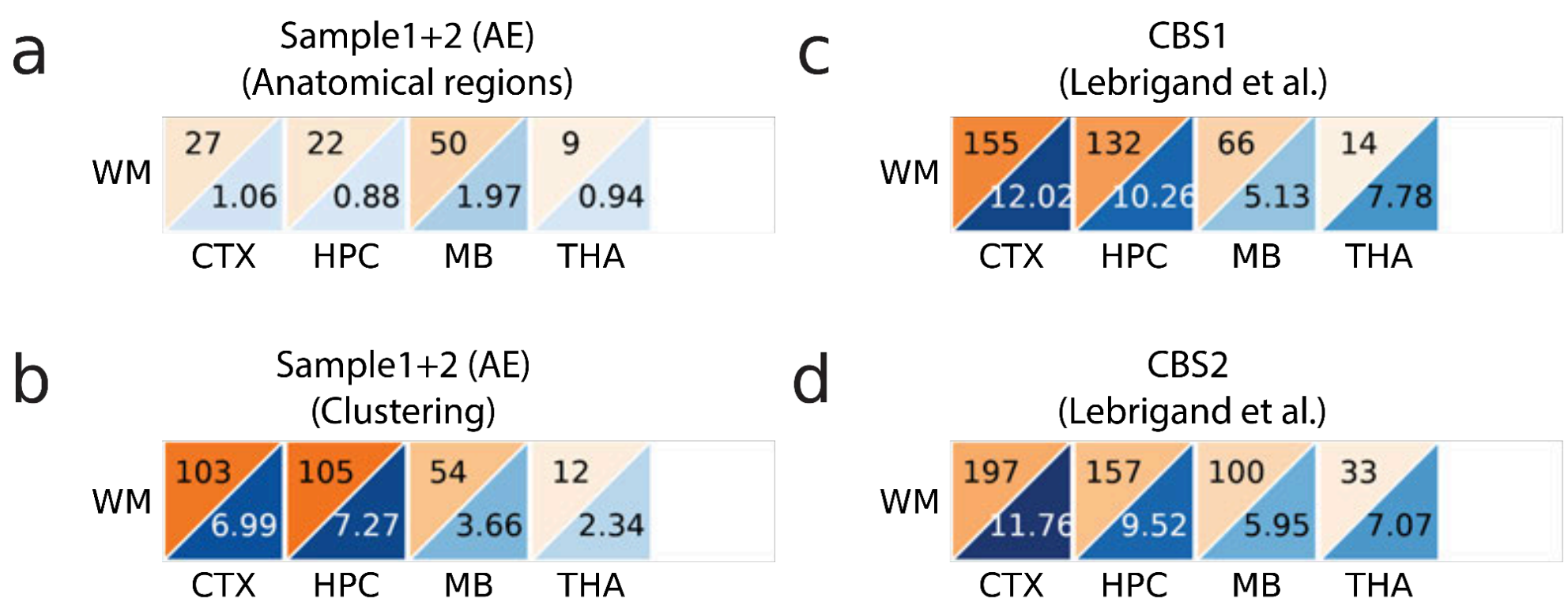

Response Figure R2. Number and percentage of genes changing isoform expression across predefined brain regions when comparing white matter to other brain regions in **a-b)** combined Sample 1+2 (AE) using regions defined based on **a)** anatomical locations and **b)** clustering the cells, **c)** Coronal Brain Section 1 (CBS1) from Lebrigand et al., and **d)** Coronal Brain Section 2 (CBS2) from Lebrigand et al..

4.2. Comparing Spl-IsoFind results between Lebrigand et al. and our data

Many of our Spl-IsoFind key findings are consistently replicated across independent platforms (Stereo-seq and Visium HD) (**Fig 5h-i**). This cross-platform concordance supports the robustness of the detected spatial isoform patterns and suggests that the observed signals reflect genuine biological structure rather than sampling noise. **In our revised manuscript, we also ran Spl-IsoFind on Lebrigand et al's data and showed that it gave consistent results with our results.** We discovered 165 and 279 spatially variable isoforms

(SVIs) in CBS1 and CBS2, respectively, from Lebrigand et al. In total, 3303 isoforms could be tested in both our combined S1+S2 (AE) data and the Lebrigand CBS1 and CBS2 samples (**Supplementary Figure S17**). The SVIs greatly overlap between the datasets, as is shown in the Venn diagram below.

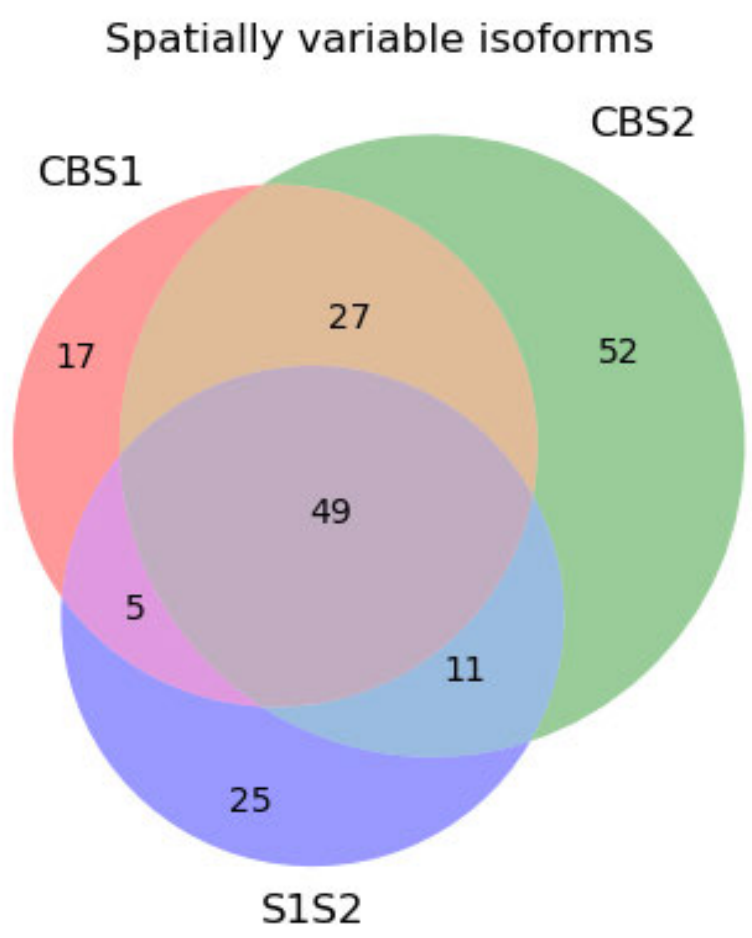

Supplementary Figure S17. Overlap between the detected SVIs in our combined Sample 1+2 (AE) data, CBS1 and CBS2 from Lebrigand et al.

Changes to the manuscript

- We added Supplementary Figure S12 and S17 and Supplementary Table S15.

- We added the following section to the Results:
  *A similar pattern appears in two previously published coronal brain sections[19] (see Methods) (**Fig S12**). However, in these data, we also detected many differences involving the white matter, which reflects imperfections in harmonizing regional annotations between studies.*

  *Besides strong agreement between the Spl-ISO-Seq2 samples, SVIs detected in the combined Sample1+2 (AE) data also greatly overlap with SVIs we discovered in the two previously published coronal brain sections[19] (**Fig S17**).*

- We added the following section to the Methods:
  Comparison to Visium coronal brain slides
  *We downloaded FASTQ files from the coronal brain slides published by Lebrigand et al.[19] from the Sequence Read Archive under accession numbers SRX8673336 and SRX8673337. We processed these reads using Spl-IsoQuant-2 and used ScisorSeqR as described earlier to detect genes that exhibit differential isoform usage. Since these datasets were annotated at a finer anatomical level, we mapped their original region annotation to the region annotations we used (**Table S15**). Finally, we detected spatially variable isoforms using Spl-IsoFind as described below.*

5. The algorithm implements several conservative decisions to minimize the chance of doublets or mixed cell spots. The tradeoff is likely that they have very low depth of UMI per cell, but these are not reported so it is difficult to get a feel for this.

We agree with the reviewer that several steps in our pipeline are conservative. In our analyses, **we prioritize specificity over sensitivity** to minimize the number of false positives.

This tradeoff is reflected in several steps of the workflow. For example, Spl-IsoQuant-2 is optimized for high precision with a slightly lower recall (**Fig 2f**). Barcodes are assigned with high confidence, but as a consequence, some reads are discarded for downstream analysis (e.g., 22% during the simulation). In our opinion, using fewer reads, but reliably assigned reads, reduces the risk of detecting false signals during downstream analysis.

A similar strategy is applied during cell-type annotation. From previous analysis on image-based spatial transcriptomics, we know that cells can overlap in a slide and doublets can thus still occur after cell segmentation (Tiesmeyer et al., 2025). Our current approach might indeed be too conservative with more doublets than expected, but this ensures that the cells used during our cell-type-specific analysis are well defined.

These decisions are important for readers to know. Therefore, also in response to previous comments (see point 1 and point 3), we have included the read depth per cell and more information about our cell segmentation and cell-type annotation methods in the manuscript. We also added a paragraph to the discussion to inform readers explicitly about these more conservative decisions, so that readers can adjust these parameters if deemed necessary for their analysis.

In summary, we do agree that our choices might be conservative, but at least this makes our conclusions more robust.

Changes to the manuscript

- We rewrote the following paragraph in the Discussion:
  The number of cell-type-specific SVIs could further be improved by decreasing the number of doublets in the data, thereby increasing the number of cells per cell type. However, due to 10µm slices, it is not always possible to completely exclude the presence of long reads from two distinct cells at the same cartesian (x,y) coordinate but varying in the "z" direction. Glial cells especially often form doublets[23], which may explain our low number of singlets for oligodendrocytes and astrocytes and the correspondingly low number of detected SVIs. Current thresholds for detecting doublets might be overly strict, but we prioritize minimizing false positives, as is also reflected in our Spl-IsoQuant-2 parameters.

Reviewer #3 (Remarks to the Author):

This manuscript presents Spl-ISO-Seq2, an innovative spatial transcriptomics method that achieves 500nm resolution with 220nm dot sizes, enabling true single-cell long-read isoform analysis. The integrated computational tools, Spl-IsoQuant-2 and Spl-IsoFind, form a novel framework for robust isoform quantification and spatial pattern detection, effectively revealing significant variations in genes like Nptn and Phactr1. Through its innovative analytical workflow, the study convincingly reveals significant isoform expression variations with specific spatial patterns within well-characterized cell types.

We sincerely thank the reviewer for these kind words and the time and effort put into reviewing our manuscript. This constructive feedback helped us to improve the paper.

In summary, we made the following major changes to the manuscript:

- We compared our results with the spatial data from the previously published work (Lebrigand et al., 2023). Overall, this comparison shows that our data has very similar properties in terms of sequencing sensitivity and statistical power for the downstream analysis. We also applied our computational methods to data from Lebrigand et al. and demonstrated that the results are consistent with the ones obtained using our Spl-ISO-Seq2 data. However, Spl-ISO-Seq2 features a truly single-cell resolution thus allowing for investigating cell-type-specific effects.
- In addition to Stereo-seq and Visium HD, the new version of the developed Spl-IsoQuant-2 software now supports common single-cell and spatial protocols (e.g., 10x Visium 3’, 10x 3’ single-cell). We also developed a universal molecule description format that allows a user to process long-read data obtained with any single-cell/spatial protocol, thus making Spl-IsoQuant-2 a universal solution for long-read transcriptomic data.
- We generated and analyzed additional PacBio and Nanopore 10x Visium HD mouse brain data (with long-molecule selection and exome enrichment), which allow for a more direct comparison between Visium HD-derived and Stereo-seq-derived data. We also showed that the cost per informative read is substantially lower when long-molecule selection and exome enrichment are applied.
- We added more biological insights by for instance predicting the amino acid sequence of the novel isoforms that are spatially variable.

Please also note that:

- “Sample 1 (4K)” is renamed to “Sample 1 (3.3K)” for a more accurate presentation of the results.
- All the analysis was redone with the updated software versions. Thus, the exact numbers may slightly differ from the previous version of the manuscript.

Below, we provide a more detailed point-by-point response to all comments.

However, I have some concerns regarding certain aspects of the manuscript.
1. The manuscript would benefit from a deeper biological analysis of a small number of representative SVIs, going beyond the current descriptive presentation. A more in-depth analysis of their data For example, the authors already highlight the SNAP25 isoform variability mainly occurred in excitatory neurons. This echos the recent findings that

excitatory neurons are evolutionarily more diverse than inbibitory neurons in Macaque V1 and multiple other species and brain regions. There are many subtypes of excitatory neurons with various layer distribution and project targets. It would be very interesting to see whether there are correlations between the isoform, synampic release modes, plasticity and isoform distribution.

We agree with the reviewer that a deeper biological analysis of some SVIs, such as *Snap25*, is of interest.

Regarding *Snap25:* *Snap25* is a core component of the SNARE complex and is involved in neurotransmitter release. Although the two isoforms *Snap25*-201 and *Snap25*-202 differ by only 9 amino acids, previous work suggests functional differences: *Snap25*-201 is linked to a higher release efficiency and increased precision compared to *Snap25*-202 (Delgado-Martinez et al, 2007; Daraio et al., 2018). This indeed suggests that isoform usage could be related to specific neuronal subtypes with different precision and synaptic strength, as the isoform usage might directly influence this. Similarly, for *Gria2*, the flip and flop variants differ in desensitization and deactivation kinetics, with the flop isoform gating more rapidly and the flip isoform exhibiting slower desensitization. Thus, isoform usage at both the presynaptic (*Snap25*) and postsynaptic (*Gria2*) level has the potential to modulate subtype-specific synaptic dynamics.

Indeed, we observe distinct isoform usage of *Snap25* in excitatory neurons of different layers. The 202 isoform is mainly used in Layer 5 (**Fig 4g**). Unfortunately, we cannot yet pinpoint whether two or more subtypes of excitatory neurons account for this observation because our short-read data is not sufficient to distinguish excitatory neuron subtypes within the cortex. As an alternative, we also explored this differently: we tested for differentially expressed genes between excitatory neurons expressing *Snap25*-201 versus *Snap25*-202 with the goal to find marker genes of specific neuronal subtypes. Unfortunately, we did not observe any differentially expressed genes between the two groups of cells (**Response Fig R3**). **In summary, *Snap25* in excitatory neurons shows differentially used isoforms between the layers. However, the sparsity inherent to spatial transcriptomics limits statistical power for cell-subtype classification within the layer.**

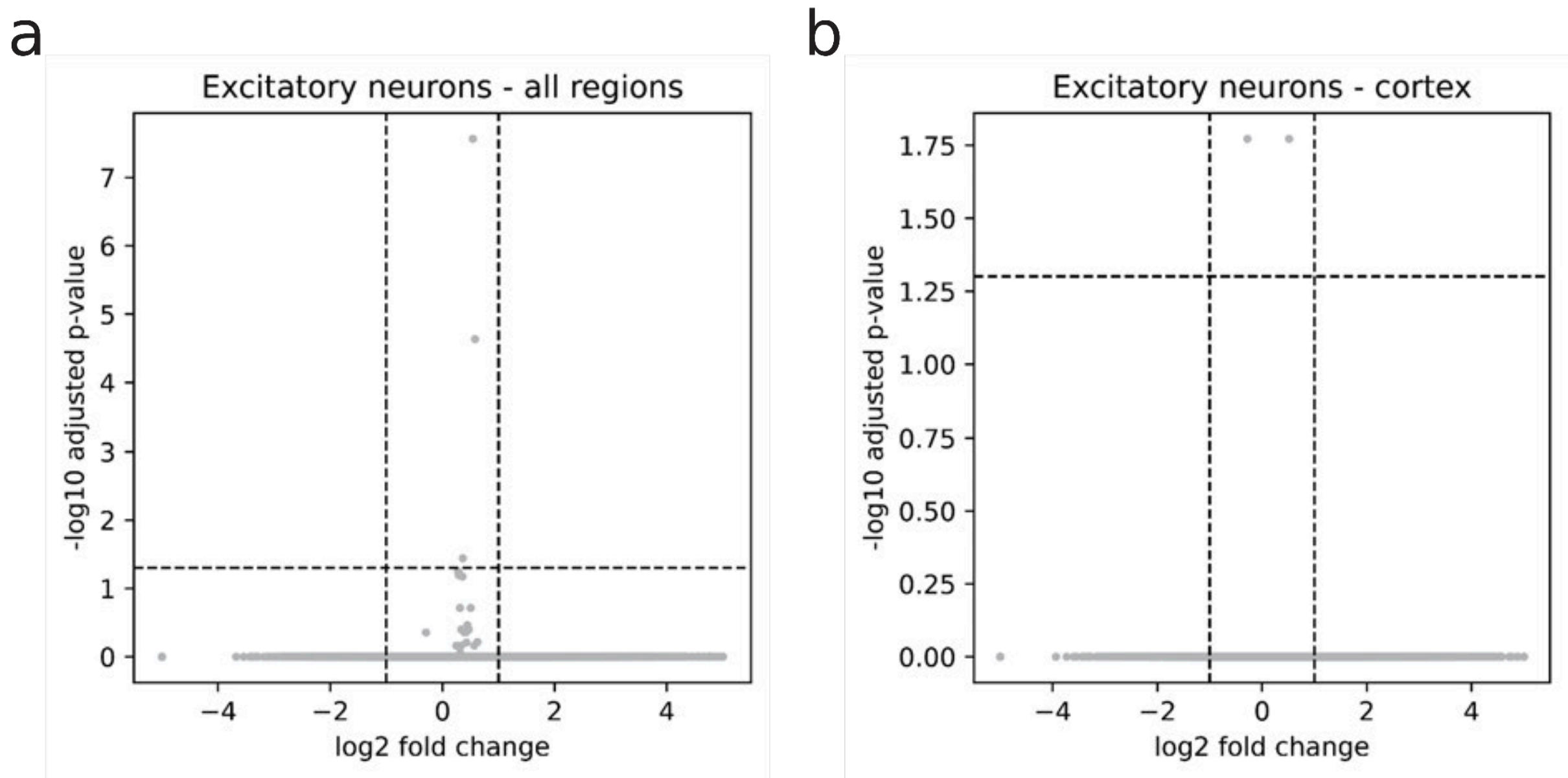

Response Figure R3. Vulcano plot showing the -log10(adjusted p-value) and log2(fold change) for each gene between excitatory neurons expressing *Snap25*-201 and *Snap25*-202, either using cells in **a)** all brain regions, or **b)** focusing on the cortex only. In both comparisons, no genes are detected with an adjusted p-value < 0.05 and |log(fold change)| > 1.

In general, excitatory neuron subtypes in the cortex are strongly layer-enriched. **Laminar isoform pattern may thus reflect subtype-specific isoform regulation**. However, these subtypes are enriched rather than strictly confined to individual layers, and individual layers might be enriched for multiple subtypes as well. Regarding layer-specific isoform usage, we do find several other interesting examples in excitatory neurons, besides *Snap25* and *Gria2* discussed earlier, that show this (**Fig 3j, Supplementary Figure S8**):

- *Snca*
- *Tpm1*
- *Caly*
- *Ppp3ca*
- *Arpp21*
- *Nnat*
- *Ccl27a*
- *Dtnbp1*

Despite these limitations, our data show that *Snap25* isoform variability is spatially structured. This may suggest that isoform regulation can provide an additional layer of neuronal specialization that is not necessarily accompanied by large gene expression changes. Future studies with increased depth or complementary functional assays will be needed to link isoform usage to synaptic release modes or plasticity.

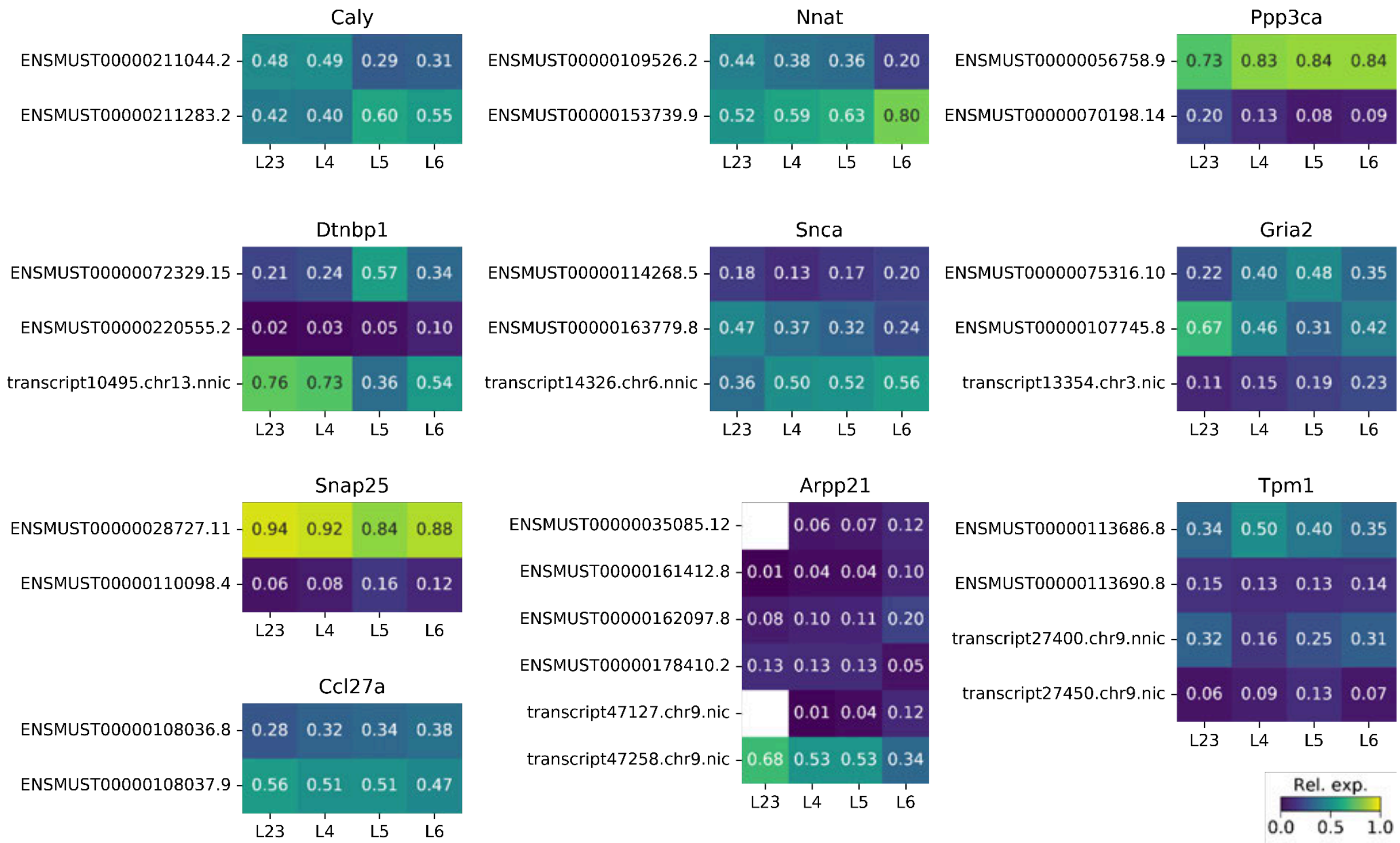

Figure S8. For all genes showing layer-specific isoform abundance in excitatory neurons, a heatmap shows the relative expression of each isoform in the different layers. Only isoforms with relative expression ≥ 0.1 in at least one layer are plotted.

Changes to the manuscript:

- We added Figure S8

- We added the following to the Results section:
  *Likewise, in the cortex, layer 2-3 showed important changes in isoform regulation especially when compared to layer 5 and layer 6 (**Fig 3j**). For some genes with layer-specific splicing changes, such as Nnat and Arpp21, the relative isoform abundance shows a gradient across the layers, while for others, such as Dtnbp1 and Tpm1, one isoform seems to be more specific to one layer (**Fig S8**). Since excitatory neuron subtypes in the cortex are strongly layer-enriched, laminar isoform patterns may reflect subtype-specific isoform regulation. However, these subtypes are enriched rather than strictly confined to individual layers, and individual layers might be enriched for multiple subtypes as well.*

2. Following the same line, it is recommended to include pathway enrichment analysis (such as GO and KEGG) to associate spatially variable isoforms (SVIs) with known neural pathways and further reveal the biological meaning of these SVIs.

We thank the reviewer for this suggestion. **In brief, we performed the suggested analyses and linked spatially variable isoform usage to molecular programs involved in synaptic release, vesicle dynamics, and neuronal excitability, thereby providing a biological context for the SVIs identified by Spl-IsoFind.**

In more detail, we did a GO enrichment analysis for SVIs identified in the combined Sample 1+2 (AE) data. Using the enrichGO() function in clusterProfiler v4.18.4 (Yu, 2024) with the

BP ontology and an FDR cutoff of q < 0.05, we identified 71 significant terms. We used the simplify() function to remove redundancy among the terms, which resulted in 29 categories (**Supplementary Figure S14**). Processes related to synaptic vesicle trafficking and regulated secretion, including vesicle-mediated transport in synapses, synaptic vesicle exocytosis, membrane fusion, and neurotransmitter secretion, are strongly enriched in the detected SVIs. Additional enriched terms were associated with regulation of synaptic signaling and membrane excitability, such as regulation of membrane potential and ion transport. A third group of enriched terms involved actin-mediated and contractility-related processes. This most likely reflects shared cytoskeletal mechanisms underlying vesicle transport and fusion rather than muscle-specific biology.

We also performed KEGG pathway enrichment analysis using the same gene set and multiple-testing correction, but no pathways reached statistical significance.

We added this analysis to the results section.

Changes to the manuscript:

- We added Figure S14

- We added the following text to the Results:
  *In the combined Sample1+2 (AE) dataset, we detected the most SVIs (157 SVIs corresponding to 94 genes). Gene ontology (GO) enrichment analysis revealed that these genes are enriched in synaptic vesicle trafficking, synaptic signaling, and membrane excitability (**Fig S14**).*

- We added the following to the Methods:
  GO-enrichment analysis
  We used the enrichGO() function in clusterProfiler v4.18.4[69] with the BP ontology and an FDR cutoff of q < 0.05. We used the simplify() function to remove redundancy among the terms.

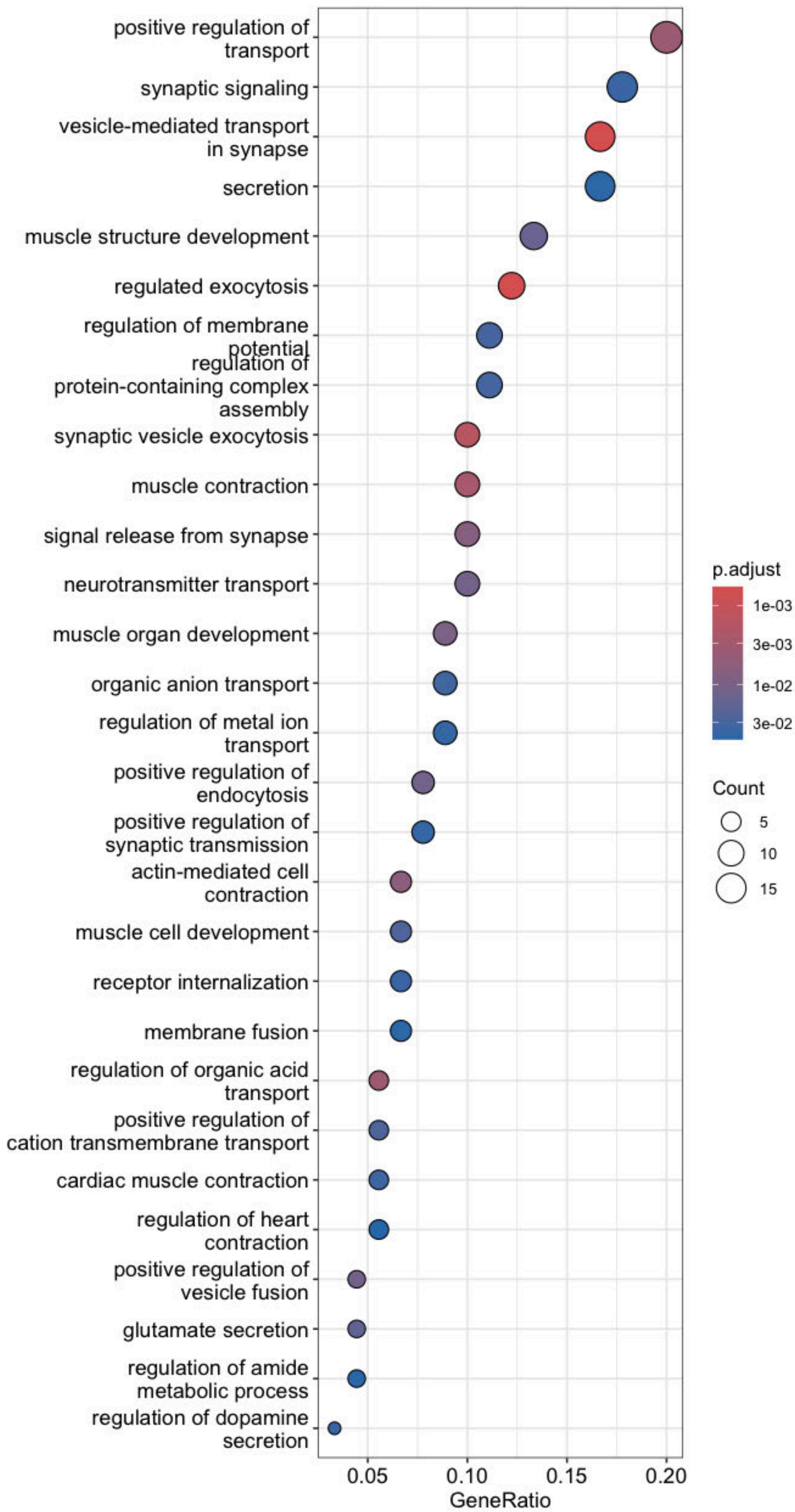

Figure S14. Significantly enriched biological processes (BP) in SVIs in the combined Sample 1+2 (AE) identified using GO enrichment analysis.

3. Quantify the frequency of concatenated cDNA molecules in raw data to underscore the necessity of the deconcatenation algorithm.

We thank the reviewer for the suggestion. **In short, the deconcatenation algorithm increases the number of detected barcoded cDNAs by 9-11%.**

We apologize that this algorithm characteristic was not explicitly demonstrated in the original manuscript. Below, we provide detailed statistics for cDNA extraction results on two Stereo-seq Nanopore datasets.

Dataset	S1 (AE)	S2 (AE)
Total reads	207,282,775	58,799,051
cDNAs extracted	218,287,260	62,775,130
Barcoded cDNAs	129,178,416	40,354,173
Barcoded cDNAs without deconcatenation	118,173,931	36,378,094
Reads with >= 2 cDNAs	10,458,285	3,903,874
Barcoded cDNAs in these reads	21,462,770	7,879,953
Additional barcoded cDNAs recovered	11,004,485	3,976,079
**Additional barcoded cDNAs recovered (%)**	**9.31%**	**10.93%**

While the number of extracted cDNAs seems to be just slightly higher than the number of sequenced reads (5-7%), this evaluation should answer the question “how many additional barcoded cDNAs were rescued by the deconcatenation algorithm compared to the single-read algorithm?”

Thus, the deconcatenation algorithm was able to recover 9-11% percent more barcoded cDNAs. Although this percentage is lower than we anticipated initially, it does provide additional data that might eventually be important for downstream statistical analysis.

We have now included this table in the supplementary material as **Supplementary Table S3**.

Changes to the manuscript:

- We added Table S3

4. The barcode identification in Spl-IsoQuant-2 demonstrates extremely high accuracy (~99.9%), but the recall rate is approximately 76.62%. This means about 24% of valid reads are discarded. Please discuss how this false-negative rate might affect downstream quantification analyses, and indicate whether any correction methods were applied.

We thank the reviewer and we now discuss the implications as suggested. Barcode detection in long reads is influenced by multiple different factors, including sequencing errors, library preparation artefacts, and read truncation. In our simulation experiment, we

only focused on the sequencing errors, as no correction algorithm can detect the barcode if it is absent due to read truncation or library construction artifacts that result in barcode loss.

1. Usage of an error-aware correction algorithm:

Indeed, we implement a barcode-correction algorithm, which is based on k-mer matching and local-alignment. Once a barcode candidate is extracted from a read, the most similar barcode sequence for the whitelist is selected. If this barcode exceeds an alignment score threshold, it is reported as the corrected version of the initial barcode. If two or more equally similar barcodes exist in a whitelist, such a read is discarded to avoid false-positive calls. We have now explicitly stated that our barcode detection algorithm performs correction.

2. Do missed barcodes introduce bias?

To investigate its performance and recall in particular, we used the simulated data without cDNA concatenation to analyze why some reads were not assigned a barcode. **Response Table R1** shows that out of 10 million reads, the barcode was detected for 76.7% reads with 99.95% precision. Out of 2,334,334 no-barcode-detected reads, for 35.2%, the linker sequence was not detected, making it impossible to accurately detect barcode coordinates. For 0.8%, the k-mer matching algorithm was not able to detect any candidate and for the remaining 64.0%, the extracted barcode had an alignment score below the threshold (default = 22). When lowering the alignment score to 21, precision dropped to 99.77%, increasing the false-positive rate more than 4-fold. Using a lower alignment score cutoff of 20 increases the false-positive rate even 20-fold (98.95% precision). A threshold of 15 drops precision to an unacceptable 86.33%. Thus, despite rejecting the barcode due to low similarity with a true barcode for roughly 15% of all reads, such a cutoff allows the algorithm to maintain extremely high precision even when sequencing errors are present. We believe that assigning a read to the wrong spot may have a worse effect compared to missing a barcoded read.

Response Table R1. Investigating barcode assignments in simulated reads.

Reads simulated	10,000,000
**Of them**	
Barcode detected	7,665,666
Correct barcode detected	7,661,747
No barcode detected	2,334,334
**Of no-barcode detected reads**	
No linker	821,786
Barcode candidate is not found	18,013
Barcode candidate score below cutoff	1,494,535

We also checked whether missed barcodes are uniformly distributed with no particular bias. The only barcodes that are missed frequently are the ones that contain the entire linker sequence (TTGTCTTCCTAAGAC), thus complicating barcode recognition. Fortunately, there are only 7,961 such barcodes in the entire whitelist containing 451,979,245 sequences (0.002%). Hence, as the probability of not calling a barcode is equal for almost all of the

barcodes, rejecting a portion of reads from the analysis is equivalent to downsampling and does not introduce any bias for downstream analysis.

In summary, the probability of any bias caused by missed barcodes is extremely small.

Changes to the manuscript:

- We rewrote the following Results section:
  *Detection of multiple concatenated isoforms is performed by locating the template-switch oligo (TSO) as well as linker and barcode sequences far from read extremities (**Fig 2b**). To enable downstream analysis of long-read Stereo-seq data, Spl-IsoQuant-2 corrects read barcodes by finding the closest candidate from the whitelist using a k-mer index and local alignment (**Fig 2c**) (see Methods for details). Our implementation indexes about half a billion Stereo-seq 25bp barcodes in less than 10 minutes using 16 threads. Barcode correction allows processing roughly 60 million ONT reads per hour without deconcatenation and 10 million reads per hour with deconcatenation in 16 threads, making it applicable even for large datasets. Although various software for barcode detection in ONT reads exists at the moment[45–47], none of these tools are capable of "parsing" Stereo-seq's molecule structure. Even when using Flexiplex, arguably the most versatile tool, we were able to process only 1 million ONT reads in 5 days (Methods).*

5. Using Moran's I in Spl-IsoFind to identify spatially variable isoforms (SVIs) is innovative. Please briefly compare its performance with existing spatial gene analysis methods adapted for this purpose to highlight its advantages.

We thank the reviewer for this suggestion. **In brief, an alternative to Moran's I, called Geary's C, is almost perfectly anticorrelated and therefore yields highly similar results.**

To our knowledge, no other existing spatial gene analysis methods have been explicitly adapted to detect spatially variable isoforms. Therefore, as an alternative, we implemented Geary's C to assess Moran's I robustness. Geary's C is another spatial autocorrelation measure and is implemented, for example, in the Scanpy framework for detecting spatially variable genes as well.

We adapted Geary's C analogously to Moran's I such that it can be applied to the relative isoform abundances. In contrast to Moran's I, a Geary's C score smaller than 1 indicates spatial autocorrelation. We evaluated both metrics on the combined Sample 1+2 (AE) data. Moran's I and Geary's C scores are negatively correlated on this data (Pearson r = -0.92), but not identical, resulting in a very similar set of spatially variable isoforms (**Supplementary Fig S15**). This indicates that the spatial isoform patterns identified by Spl-IsoFind are robust to the choice of spatial autocorrelation metric.

Since both metrics perform similarly, we have added Geary's C as an alternative option in the Spl-IsoFind Python package.

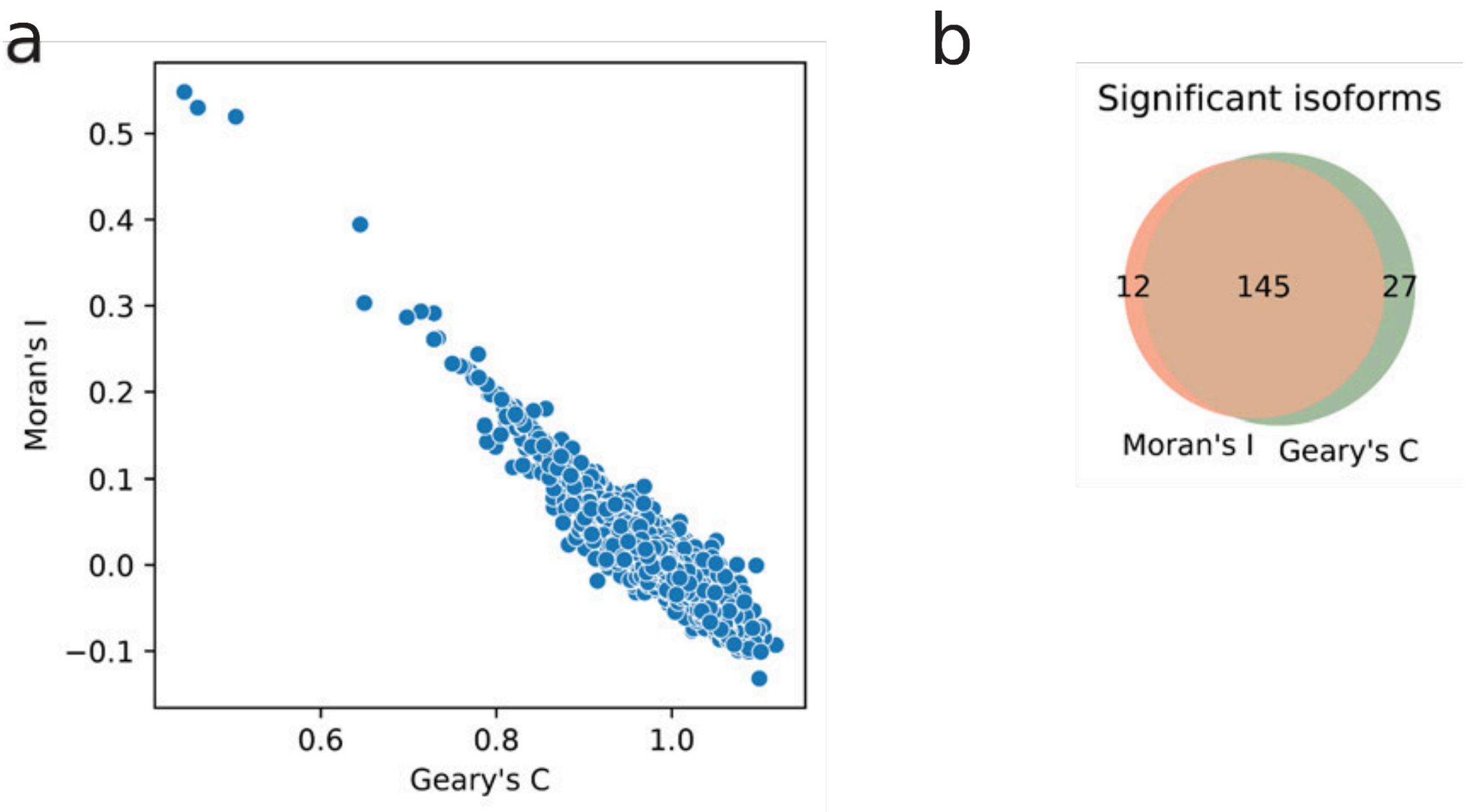

Figure S15. a) Geary's C vs. Moran's I score for the tested isoforms in the combined Sample 1+2 (AE) data. **b)** Venn diagram showing the overlap of significant isoforms when using Moran's I or Geary's C.

Changes to the manuscript:

- We added Figure S15

- We added the following to the Results:
  In general, the Moran's I score is very correlated to Geary's C[55]*, another autocorrelation metric more sensitive to local variations, which discovered almost the exact same set of SVIs for combined Sample1+2 (AE) (**Fig S15**).*

- We added the following to the Methods:
  When comparing to Geary's C, we replaced the `esda.Moran` *function by* `esda.Geary`*. Both options are available in the Spl-IsoFind package.*

6. Fig. S3 shows that the correlation between long and short read gene expression in Sample 1 (4K) (Pearson r=0.67) is noticeably lower than in other samples. Please explain the reasons for this discrepancy and whether this sequencing approach is less effective for genes associated with brain diseases.

This is indeed a fundamental question that we had not sufficiently considered. **In brief, this is due to the gene-set selection, which strongly reduces zero inflation as we selected 'interesting known' genes.**

The lower correlation observed in Sample 1 (3.3K) is mainly explained by the distribution of the genes included in the analysis rather than by differences in sequencing performance. In the AE samples, we calculate the correlation across all genes, which include many genes with zero expression in both the short-read (SR) and long-read (LR) data (**Response Figure R4**). These shared zero values inflate the Pearson correlation. In contrast, the 3.3K dataset contains few genes with zero or low expression values, which results in a lower correlation coefficient.

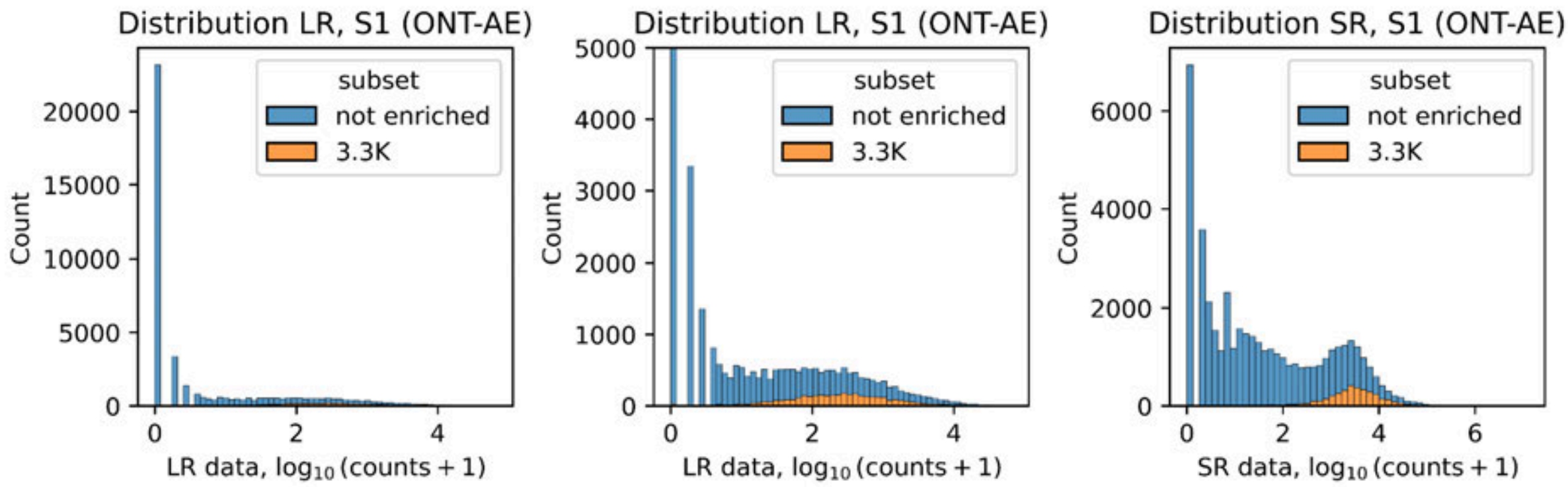

Response Figure R4. Gene expression distribution in the long-read and short-read Sample 1 (ONT-AE) data. Panel B is the zoomed-in version of the y-axis of Panel A to better show the distribution. Genes included in the 3.3K enriched are shown in orange, non-enriched genes are shown in blue.

When we subset the AE datasets for the 3.3K enriched genes, the correlation between the SR and LR data is very similar to the correlation on the 3.3K enriched dataset (**Response Figure R5**), which indicates that the lower correlation is indeed driven by the gene set. In conclusion, the lower correlation does not reflect differences in sequencing efficiency, but the statistical properties of the gene sets being compared.

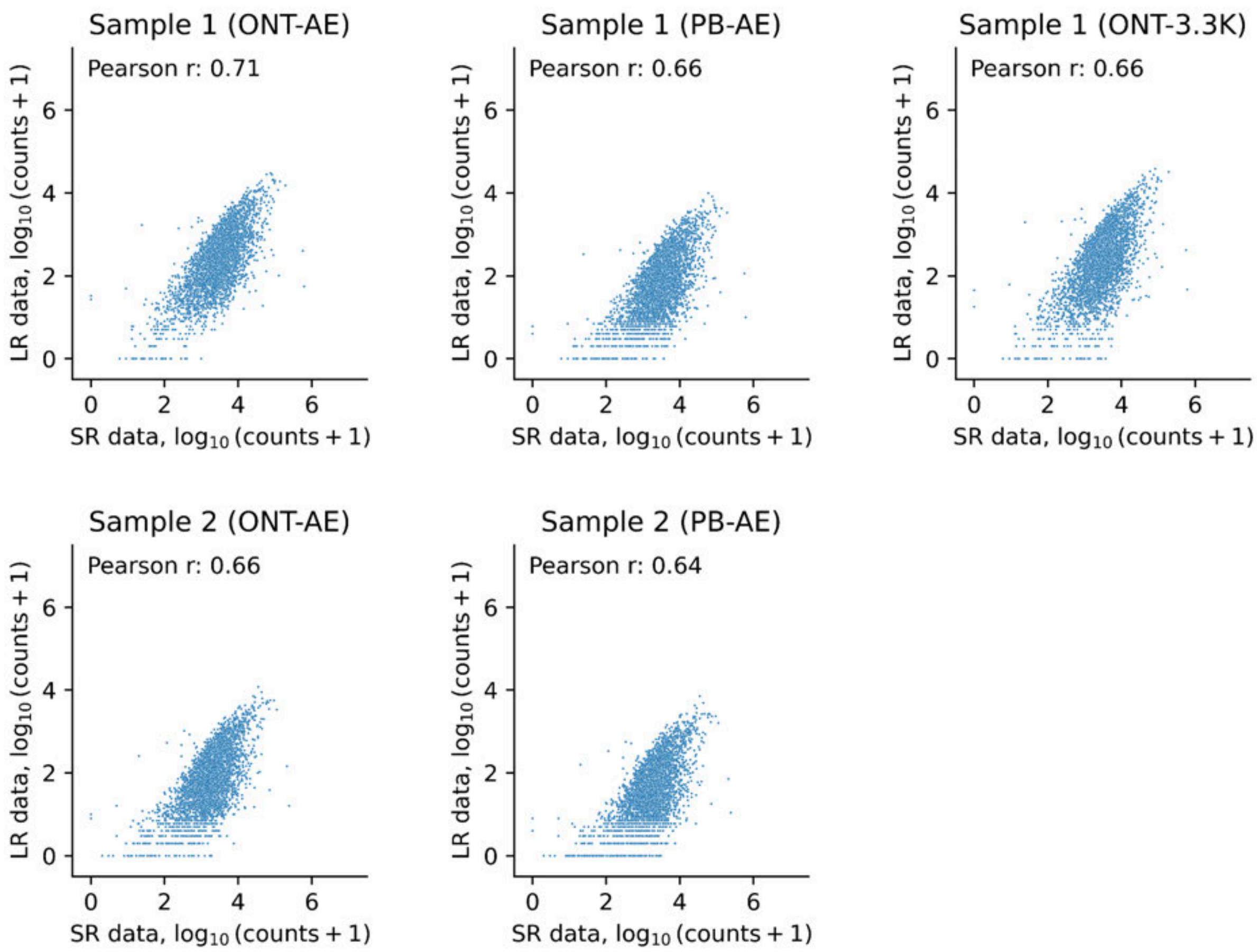

Response Figure R5. Correlation between gene expression as measured using the long-read and short-read sequencing subsetted for the 3.3K genes.

7. When comparing datasets, as illustrated in Fig. 4a more detailed explanation of the potential biases inherent in targeted sequencing is essential.

We assume that the reviewer refers to Fig. 4b-d instead of Fig. 4a. In Fig. 4b-d, we show the performance of Spl-IsoFind on multiple datasets including the targeted Sample 1 (3.3K), while Fig. 4a shows a schematic illustration to explain the concept of spatial autocorrelation. **Importantly, as shown in Fig. 4a, all statistical tests are performed within a single dataset and within each gene (for predefined regions) or isoform (for spatial autocorrelation).** We do not combine targeted and non-targeted datasets in a joint analysis.

In principle, targeted sequencing introduces a bias in measured gene expression for the targeted genes. However, **Spl-IsoFind detects spatial variation by using relative isoform expression within each gene**, rather than by comparing expression levels across genes or datasets. **Enrichment primarily affects read depth per gene/isoform and thus statistical power, but does not systematically bias isoform ratios for a given gene**.

The differences between the AE and 3.3K datasets in Fig. 4b-d reflect this difference in statistical power. For example, the higher percentage of significant isoforms for S1 3.3K in Fig. 4c arises from evaluating performance within the enriched gene subset. Fig. 4d shows a substantial overlap in spatially variable isoforms detected across datasets. However, some isoforms are only identified in the combined S1+S2 (AE) data and S1 (AE) or only identified in S1 (3.3K), which is largely explained by higher read depth and increased statistical power (see **Fig. S10**). When comparing Sample 1 (AE) and Sample 1 (3.3K) in more detail, we noticed that, of the isoforms tested in both datasets, only 15 isoforms are specific to Sample 1 (AE) (**Response Figure R6a**). Eleven out of 15 isoforms were not targeted during enrichment, so even though we had even reads to test them, we did not expect to detect these in our S1 3.3K dataset. Furthermore, we would like to emphasize that the Moran's I scores in both datasets are very correlated and that certain dataset-specific SVIs are thus a cut-off question (i.e., adjusted p-value slightly below or above 0.05) (**Response Figure R6b**).

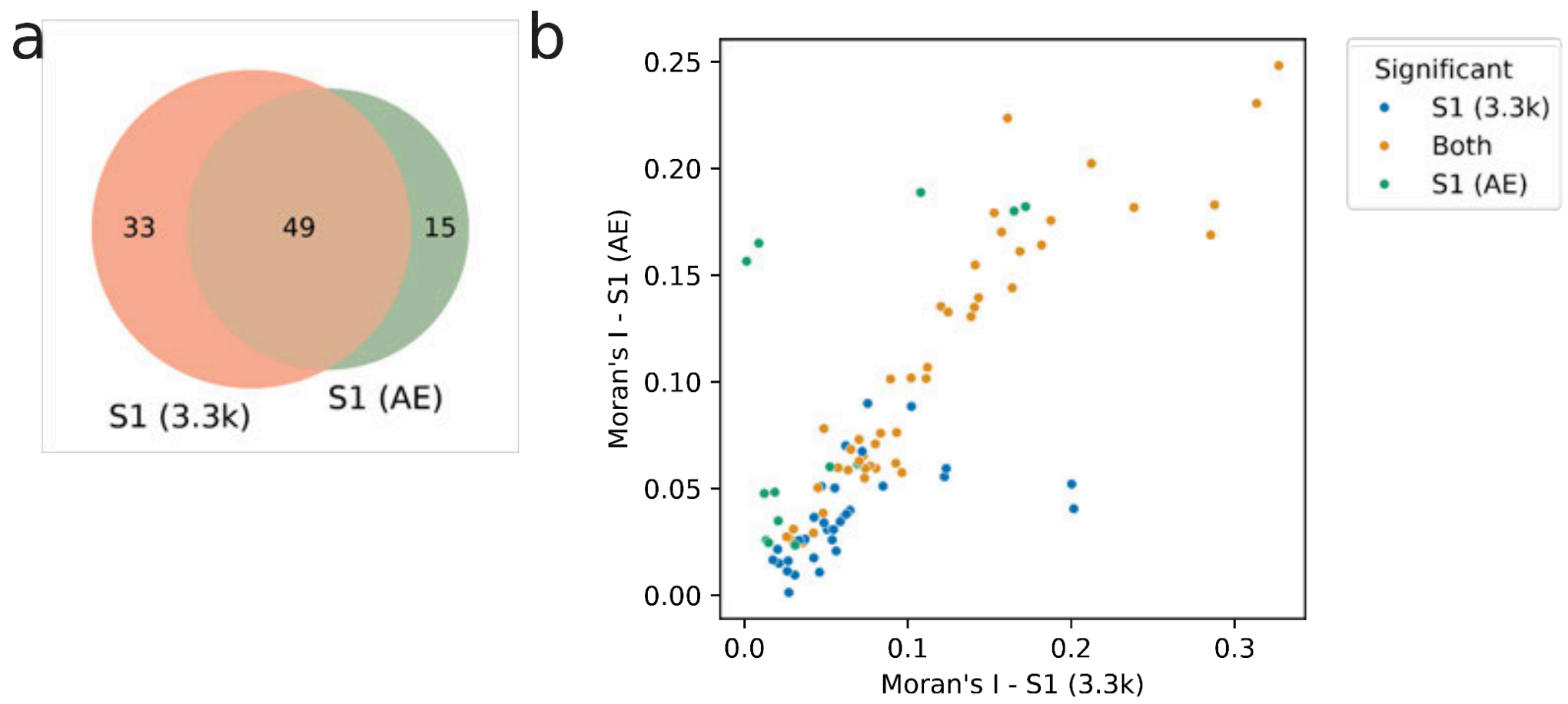

Response Figure R6. a) Venn diagram showing the overlap in significant genes between Sample 1 (3.3K) and Sample 1 (AE). Only isoforms tested in both datasets are considered. **b)** Scatterplot showing the Moran's I score for S1 (3.3K) vs. S1 (AE).

8. Fig. 4i depicts four isoforms of Arpp19 with highly complex spatial patterns. How can this biological complexity be interpreted? Are there known mechanisms or functional implications associated with such diverse spatial isoform distributions?

We thank the reviewer for highlighting this example - we were very ignorant about it. **In brief, our findings below suggest that the complex spatial patterns of Arpp19 isoforms reflect both functional diversification at the protein level (ARPP16 vs ARPP19) and regulatory diversification at the 3′ UTR level, consistent with known mechanisms of brain-specific isoform regulation.**

The four detected *Arpp19* transcripts encode two related proteins: ARPP16 (encoded by transcript 203 and the newly identified transcript) and ARPP19 (encoded by transcripts 205 and 209). These proteins both regulate protein phosphatase 2A (PP2A). ARPP19, which is broadly expressed, plays a well-established role in cell cycle regulation and mitotic control, whereas ARPP16 has been implicated in neuronal signaling and regulation of PP2A activity in a neural context. ARPP16 is thought to have emerged later during evolution and has been associated with more complex brain functions (Girault et al., 1990).

In our data, oligodendrocytes and astrocytes predominantly express ARPP19-encoding transcripts, while both excitatory and inhibitory neurons express transcripts encoding both ARPP16 and ARPP19, with the highest levels of ARPP16 observed in the cortex (**Supplementary Figure S23**). This suggests region- and cell-type-specific regulation of ARPP16 versus ARPP19 isoform usage.

In addition, the two ARPP19 transcripts differ in their 3′ UTR length. Alternative 3′ UTR usage, which is common in the brain, can influence mRNA stability, localization, and translational efficiency, and has been linked to regulatory factors such as PQBP1 (Miura et al., 2013; Liu et al., 2024). The spatially distinct distribution of these 3′ UTR isoforms thus likely reflects an additional layer of post-transcriptional regulation, beyond protein-coding differences.

We added these interpretations to the manuscript.

Changes to the manuscript:

- We added Figure S23

- We added the following text to the Results:
  Arpp19-205 and Arpp19-209 both encode ARPP19, whereas Arpp19-203 and Arpp19-new encode ARPP16. ARPP16 and ARPP19 differentially regulate protein phosphatase 2A (PP2A) activity. ARPP19 has established roles in cell-cycle regulation and phosphatase inhibition, whereas ARPP16 appears more enriched in neural tissues and has been implicated in brain-specific PP2A modulation[62]. In addition, the two ARPP19 isoforms (Arpp19-205 and Arpp19-209) differ in their 3′ UTR length, suggesting potential differences in post-transcriptional regulation, such as mRNA stability or translational control, which is a common regulatory mechanism in mammalian brains[63].

9. For newly identified high-confidence isoforms (e.g., Arpp19), it would be beneficial to conduct preliminary bioinformatics functional predictions to gain insights into their potential biological roles.

This is a really interesting suggestion. To gain insight into the potential biological roles of newly discovered isoforms, we translated the predicted open reading frame using the ExPASy translation tool (Gasteiger et al., 2003) and compared the resulting amino acid sequence to annotated UniProt entries. We performed such predictions for the three genes with newly discovered isoforms highlighted in the manuscript: *Arpp19, Tncc1,* and *Gad2.*

Arpp19

Arpp19 encodes two related proteins, ARPP19 and ARPP16. ARPP16 is a shorter isoform lacking part of the N-terminus present in ARPP19. The newly identified transcript is predicted to encode ARPP16, indicating that it does not introduce a novel protein product but rather alters the spatial distribution of a known protein isoform. As discussed in the previous answer, ARPP16 and ARPP19 have distinct regulatory roles in PP2A modulation.

Tnnc1

Tnnc1-202, the canonical transcript, encodes a 161 amino acid protein containing four EF-hand motifs, three of which are functional Ca^{2+}-binding domains (Li and Hwang, 2015). The newly identified isoform is predicted to encode a truncated 117 amino acid protein lacking the N-terminal region, which includes the first EF-hand motif. The remaining EF-hand domains are preserved, suggesting that the protein may retain partial Ca^{2+}-binding capacity. While the precise role of *Tnnc1* in the brain remains unclear, this truncation may alter calcium sensitivity or regulatory interactions.

Gad2

Two novel *Gad2* isoforms were identified. *Gad2*-new1 is predicted to encode a 471 amino acid protein that lacks part of the N-terminal region compared to the canonical isoform *Gad2*-201. *Gad2*-new2 is predicted to encode a shorter 189 amino acid product. Highly similar protein sequences are annotated in human, with strong sequence conservation (97.9% for the 471 aa isoform and 88.2% for the 189 aa isoform). The 189 aa isoform lacks an in-frame stop codon and is annotated in human as a likely target of nonsense-mediated decay (NMD), suggesting that it may represent a regulatory rather than protein-coding transcript. Prior work in the human cortex has reported developmental and disease-associated shifts in full-length canonical versus NMD-associated *GAD2* transcripts (Davis et al., 2016).

Together, these analyses support that the newly identified isoforms are likely biologically meaningful. We have added a brief functional description for each isoform to the revised manuscript.

Changes to the manuscript:

- We added the following to the Results:
  Arpp19-205 and Arpp19-209 both encode ARPP19, whereas Arpp19-203 and Arpp19-new encode ARPP16. ARPP16 and ARPP19 differentially regulate protein phosphatase 2A (PP2A) activity. ARPP19 has established roles in cell-cycle regulation and phosphatase inhibition, whereas ARPP16 appears more enriched in

neural tissues and has been implicated in brain-specific PP2A modulation[62]. In addition, the two ARPP19 isoforms (Arpp19-205 and Arpp19-209) differ in their 3' UTR length, suggesting potential differences in post-transcriptional regulation, such as mRNA stability or translational control, which is a common regulatory mechanism in mammalian brains[63].

Tnnc1-202, the canonical transcript, encodes a 161 amino acid (aa) protein containing four EF-hand motifs, three of which are functional Ca^{2+}-binding domains[64]. The newly identified isoform is predicted to encode a truncated 117 aa protein lacking the N-terminal region which includes the first EF-hand motif. The remaining EF-hand domains are preserved, suggesting that the protein may retain partial Ca^{2+}-binding capacity. While the precise role of Tnnc1 in the brain remains unclear, this truncation may alter calcium sensitivity or regulatory interactions.

The two Gad2 SVIs are both newly discovered isoforms using IsoQuant. Gad2-new1 is predicted to encode a 471 aa protein that lacks part of the N-terminal region compared to the canonical isoform Gad2-201, which is not spatially variable in our data. Gad2-new2 is predicted to encode a shorter 189 aa product. Highly similar protein sequences are annotated in human, with strong sequence conservation (97.9% for the 471 aa isoform and 88.2% for the 189 aa isoform). The 189 aa isoform lacks an in-frame stop codon and is annotated in human as a likely target of nonsense-mediated decay (NMD), suggesting that it may represent a regulatory rather than protein-coding transcript. Prior work in the human cortex has reported developmental and disease-associated shifts in full-length canonical versus NMD-associated GAD2 transcripts[65].

10. The study emphasizes Spl-ISO-Seq2's 500 nm resolution but lacks direct comparisons with other methods. Providing contrasts in resolution, throughput, cost, and other key metrics would better highlight its competitive advantages.

This is a good suggestion by the reviewer. We now added a comparison regarding resolution, throughput, cost, and detected spatially variable isoforms to the manuscript. We compared our Stereo-seq-derived data and newly added Visium HD-derived data (with exome enrichment and long-molecule selection) to long-read spatial transcriptomics data from Lebrigand et al. (Lebrigand et al., 2023). **In summary, the throughput, expected cost, and detected number of spatially variable isoforms are comparable, while we offer single-cell resolution.**

<u>10.1 Sequencing throughput and sensitivity</u>

We compared our sequencing throughput to another long-read spatial transcriptomics dataset (which, in contrast to ours, does not have single-cell resolution) in the mouse brain. We compared overall sequencing statistics to closely related work, which also sequenced two coronal mouse brain sections using Nanopore, but relied on the Visium technology with 55µm (implying no single-cell resolution) to generate the cDNA (Lebrigand et al., 2023). They sequenced two coronal sections (CBS1 and CBS2) using three and eight flow cells, resulting in 174M and 286M reads, respectively. **This is very comparable to the number of sequenced reads for our Sample 1 (AE) (207M).**

To ensure a fair comparison, we reprocessed this data using our tool Spl-IsoQuant-2. However, for the Lebrigand et al. data, a subset, containing 44M and 99M reads, respectively, was uploaded to SRA. The uploaded data likely represent reads already enriched for informative molecules, as the number of reads after UMI deduplication is consistent with those reported in their Supplementary Information. When comparing the informative reads (UMI-deduplicated, spliced reads with an assigned isoform and overlapping a segmented cell or spot in the correct hemisphere), we observe a comparable number of informative reads (~8M) between Sample 1 (AE) and CBS1 and CBS2 (**Supplementary Table S9**). **This similar number of informative reads indicates that the overall sensitivity is comparable, while Spl-ISO-Seq2 additionally enables a single-cell resolution.**

In this revised version of the manuscript, we added new Visium HD datasets, which we sequenced with long-molecule selection and exome enrichment. This sample, V1, was sequenced on a single PromenthION flow cell and a single PacBio SMRT cell, resulting in 8.4M and 7.0M informative reads, respectively. The number of informative reads is again comparable to that of Sample 1 (AE) and the Lebrigand Visium data. **However, for the Visium HD, fewer sequenced reads were needed (99-127M), indicating a slightly higher sensitivity** (**Supplementary Table S9**). The higher sensitivity could be explained by the absence of gaps between the barcoded spots on the Visium HD slide, which is the case for the Stereo-seq and Visium platforms. The resolution of Visium HD-derived data is slightly lower compared to Stereo-seq-derived data (2µm compared to 500nm).

Table S9. A comparison of read statistics between samples generated using the Stereo-seq, Visium HD, and Visium platforms. Informative reads are defined as UMI-deduplicated, spliced reads with an assigned isoform and overlapping a segmented cell or spot in the correct hemisphere. However, for the Lebrigand et al paper, a subset, containing 44M and 99M reads, respectively, was uploaded to SRA. The uploaded data likely represent reads already enriched for informative molecules, as the number of reads after UMI deduplication is consistent with those reported in their Supplementary Materials.

	Stereo-seq Sample 1 (AE - ONT)	Visium HD V1 (ONT)	Visium HD V1 (PacBio)	Visium CBS1	Visium CBS2
Sequenced reads	207,282,773	98,713,075	126,023,947	173,996,000	286,458,673
Analysed reads	207,282,773	98,713,075	126,023,947	43,899,878	98,892,086
Barcode detected	130,360,866	49,089,229	84,196,089	36,625,448	80,219,056
Uniquely assigned	100,571,599	38,561,039	66,968,834	31,032,886	67,505,940
Uniquely assigned and spliced	79,030,635	25,987,614	53,617,739	19,972,628	48,768,593
Reads after deduplication	36,728,127	22,313,578	18,616,621	17,581,867	18,489,755
Spliced reads after deduplication	25,916,277	14,740,254	13,620,397	10,929,678	12,189,522
Informative reads	8,006,816	8,354,635	6,982,558	8,204,667	8,749,685

10.2 A cost comparison

We have added a cost table including the total cost per experiment and cost per informative read for our Stereo-seq-derived and Visium HD-derived data (Table S26-27). We report the cost per 1M sequenced reads and per 100K informative reads (i.e., reads usable for downstream analysis), with the latter being the more relevant metric. Based on this table, we want to highlight some interesting observations:

- V1-ONT, generated using long-molecule selection and exome enrichment, was sequenced on a single flow cell and yielded 8.4M informative reads. In contrast, V2-ONT, which was sequenced without enrichment, required four flow cells and yielded only 7.2M informative reads, resulting in a substantially higher cost per informative read.
- For PacBio sequencing, V1-PB produced considerably more total reads than Sample 1 (AE) - PB and Sample 2 (AE) - PB, thereby considerably reducing both the cost per sequenced read and the cost per informative read. Samples 1 and 2 were sequenced using MAS-Seq with 16-fold concatenation, whereas V1 was sequenced using the Kinnex kit with 12-fold concatenation. Given the relatively long read length after long-molecule selection (**Fig. 5b**), 16-fold concatenation likely generated molecules that exceeded optimal sequencing length, thereby reducing yield. The 12-fold concatenation used for V1 appears better suited for our read length distribution.
- The cost per informative read for Sample 2 (AE) is considerably higher because a large fraction of reads mapped to cells in the opposite hemisphere, which were excluded from downstream analysis.

We did not include the Lebrigand et al. data in this table, as we did not generate these datasets ourselves. However, given that a similar number of sequenced reads yields a comparable number of informative reads as observed for Sample 1 (AE), the expected cost per informative read will also be similar.

Table S26. Estimated costs for the individual steps during each experiment. Per type of experiment we also indicated which steps were included. The total cost per experiment is shows in Table S27. Shared steps (e.g., the Stereo-seq slide) are included for multiple experiments, even when the same slide was used in practice to get a fair estimate of the cost per read (e.g., as in Sample 1 (AE) and Sample 1 (3.3K)). The reported costs are an estimate and may vary across institutes depending on available infrastructure and vendor discounts.

	Est. cost	S1/S2 (AE) ONT	S1/S2 (AE) PB	S1 (3.3K) ONT	V1 ONT	V1 PB	V2 ONT	V3 PB
Stereo-seq slide (including SR seq.)	$5000	1x	1x	1x				
Visium HD slide (excluding SR seq.)	$4500				1x	1x	1x	1x
Long-mol. selection	$35	1x	1x	1x	1x	1x		
Exome enrichment	$155	1x	1x	1x	1x	1x		
3378 gene enrichment	$350			1x				
ONT library prep	$120	1x		1x	1x		1x	
PromethION flow Cell	$1040	1-2x		1x	1x		4x	
PacBio library prep	$475		1x			1x		1x
PacBio SMRT Cell	$1700		1x			1x		4x

Table S27. Estimated total cost per experiment and cost per sequencing output. Costs are reported per 1M total reads and per 100K informative reads. Informative reads are spliced reads with an assigned isoform overlapping a segmented cell in the correct hemisphere and thus suitable for downstream analysis. Estimates are calculated for the complete workflow and for the long-read sequencing part alone.

	Including array prep			Long-read sequencing only			
	Est. total cost	Cost per 1M seq. reads	Cost per 100K informative reads	Est. total cost	Cost per 1M seq. reads	Cost per 100K informative reads	
S1 (AE) - ONT	$7,390	$36	$92	$2,390	$12	$30	
S1 (AE) - PB	$7,365	$368	$323	$2,365	$118	$104	S1 (AE) - PB was sequenced using PacBio Mas-Seq with 16-fold concatenation resulting in fewer reads compared to V1 - PB, which was sequencing with the Kinnex kit with 12-fold concatenation
S1 (3.3K) - ONT	$6,700	$76	$325	$1,700	$19	$82	
S2 (AE) - ONT	$6,350	$108	$264	$1,350	$23	$56	For S2 (AE) - ONT, many reads were mapped to cells in the other hemisphere that we discarded. This largely accounts for the difference compared to S1 (AE) - ONT
S2 (AE) - PB	$7,365	$409	$550	$2,365	$131	$177	S2 (AE) - PB was sequenced using PacBio Mas-Seq with 16-fold concatenation resulting in fewer reads compared to V1 - PB, which was sequencing with the Kinnex kit with 12-fold concatenation
V1 - ONT	$5,850	$59	$70	$1,350	$14	$16	
V1 - PB	$6,865	$54	$98	$2,365	$19	$34	
V2 - ONT	$8,780	$15	$122	$4,280	$7	$60	In comparison to V1-ONT, this did not use exome enrichment and long-molecule selection. As a result, it needed more flow cells, raising the cost
V3 - PB	$11,775	$28	$93	$7,275	$17	$58	In comparison to V1-PB, this did not use exome enrichment and long-molecule selection. As a result, it needed more flow cells, raising the cost

10.3 Comparing the predefined region analysis

Besides comparing sequencing statistics, we also compared whether similar methods give similar downstream results. As such, **we analysed the Lebrigand et al. data using our predefined region analysis using our *n* x 2 isoform tests (this part) and Spl-IsoFind (see 10.4) and compared the results to our data.**

For the predefined region analysis, we had to harmonize the region annotation between our data and the Lebrigand et al. dataset. We mapped the subregions used by Lebrigand et al. to the broader regions used in our analysis (**Supplementary Table S15**). Unfortunately, this mapping is imperfect. For example, we assigned the fiber tract spots to the white matter, but in Lebrigand et al.'s data this also includes some spots at the boundary of the midbrain and hippocampus. As such, we excluded white matter/fiber tracts from the comparison in this first part of the response.

Supplementary Table S15. Mapping between the original and new labels for the Lebrigand et al. dataset.

**Original label**	**New label**
CA1/CA2, CA3, DG, Hippocampus area	Hippocampus
Iscortex-1, Isocortex-2, Olfactory area, Retrosplenial area,	Cortex
Fiber tracts *(only considered in the later part of the response)*	White Matter
Thalamus	Thalamus
Midbrain, Hypothalamus	Midbrain

Next, we applied our *n x 2* isoform tests to their relabeled spots. **The resulting heatmaps closely resemble our heatmap in Figure 3A (Response Figure R1)**. The exact numbers of significant genes vary, also between Lebrigand et al's two replicates, which have a similar read depth, but the three heatmaps show the same pattern: **Midbrain vs Cortex and Midbrain vs Hippocampus result in the most significant genes, while other comparisons yield fewer differences - in our approach and in the data of Lebrigand et al**.

Despite our approach having similarly high numbers of differentially spliced genes, the percentage of significant genes is lower in our combined Sample 1+2 (AE) data. Thus, our approach tests more genes but does not give more significant genes. Despite spending significant time investigating this discrepancy, we remain unsure regarding the causes, as the read depth is very similar in both samples.

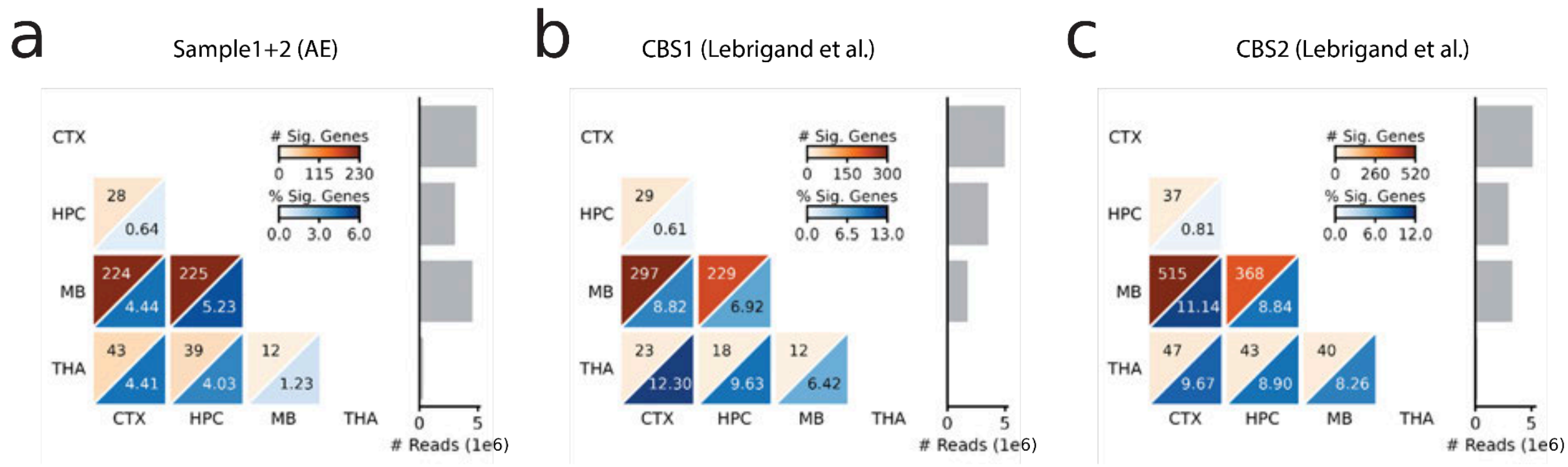

Response Figure R1. Number and percentage of genes changing isoform expression across predefined brain regions using all cells in **a)** combined Sample 1+2 (AE), **b)** Coronal Brain Section 1 (CBS1) from Lebrigand et al., and **c)** Coronal Brain Section 2 (CBS2) from Lebrigand et al.. The barplot on the right shows the number of long reads used during the analysis per brain region.

We observe stronger discrepancies when adding fiber tracts and white matter to the comparison. In CBS1 and CBS2, samples from Lebrigand et al., many differentially spliced genes are found in comparisons involving the fiber tracts. In our data, we observe fewer events in comparisons involving the white matter (**Response Figure R2**). We defined our regions based on anatomical locations, while they clustered their spots and assigned labels to the spots. When redefining our white matter by clustering the data to include fiber tracts as well, we see a lot of significant genes as well. **The apparent discrepancy between white matter and fiber tracts is thus fundamentally a difference in how these regions were defined.**

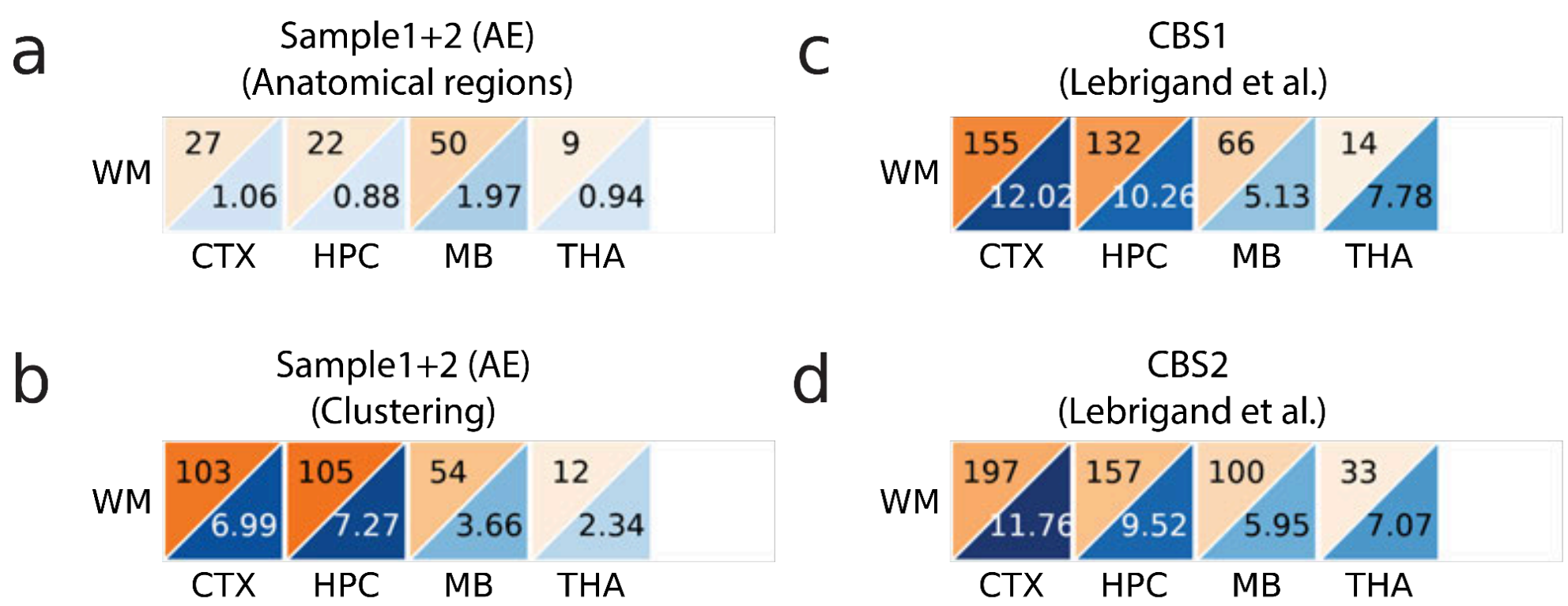

Response Figure R2. Number and percentage of genes changing isoform expression across predefined brain regions when comparing white matter to other brain regions in **a-b)** combined Sample 1+2 (AE) using regions defined based on **a)** anatomical locations and **b)** clustering the cells, **c)** Coronal Brain Section 1 (CBS1) from Lebrigand et al., and **d)** Coronal Brain Section 2 (CBS2) from Lebrigand et al..

10.4 Comparing Spl-IsoFind results between Lebrigand et al. and our data

Many of our Spl-IsoFind key findings are consistently replicated across independent platforms (Stereo-seq and Visium HD) (**Fig 5h-i**). This cross-platform concordance supports the robustness of the detected spatial isoform patterns and suggests that the observed signals reflect genuine biological structure rather than sampling noise. **In our revised manuscript, we also ran Spl-IsoFind on Lebrigand et al's data and showed that it gave**

consistent results with our results. We discovered 165 and 279 spatially variable isoforms (SVIs) in CBS1 and CBS2, respectively, from Lebrigand et al. In total, 3303 isoforms could be tested in both our combined S1+S2 (AE) data and the Lebrigand CBS1 and CBS2 samples (**Supplementary Figure S17**). The SVIs greatly overlap between the datasets, as is shown in the Venn diagram below.

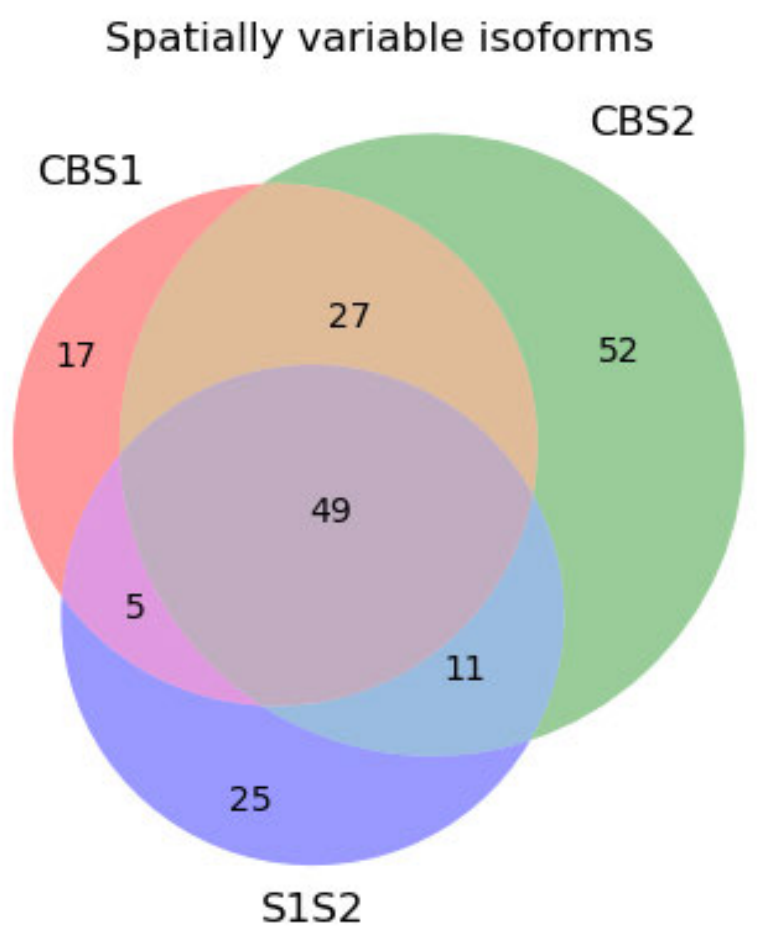

Supplementary Figure S17. Overlap between the detected SVIs in our combined Sample 1+2 (AE) data, CBS1 and CBS2 from Lebrigand et al.

In summary, the cost, throughput, and number of SVIs is very similar between our data and the Lebrigand et al. data, but we provide the extra single-cell resolution.

Changes to the manuscript

- We added Supplementary Figure S12 and S17 and Supplementary Table S15 and S26-27.

- We added the following section to the Results:
  *We compared the number of sequenced reads required to obtain a given number of informative reads (spliced reads with an assigned isoform that overlap a segmented cell in the correct hemisphere) to a similar long-read spatial transcriptomics dataset but generated on the Visium platform[19] (Methods). Both platforms show a comparable sensitivity as a similar number of sequenced reads (~200M) yields a comparable number of informative reads (~8M) (**Table S9**). Importantly, Spl-ISO-Seq2 additionally provides single-cell resolution.*

- *A similar pattern appears in two previously published coronal brain sections[19] (see Methods) (**Fig S12**). However, in these data, we also detected many differences involving the white matter, which reflects imperfections in harmonizing regional annotations between studies.*

  *Besides strong agreement between the Spl-ISO-Seq2 samples, SVIs detected in the combined Sample1+2 (AE) data also greatly overlap with SVIs we discovered in the two previously published coronal brain sections[19] (**Fig S17**).*

*For both ONT and PacBio, applying long-molecule selection and exome enrichment thus greatly reduced the sequencing cost per informative read (**Table S26-27**).*

- We added the following section to the Methods:
  Comparison to Visium coronal brain slides
  *We downloaded FASTQ files from the coronal brain slides published by Lebrigand et al.[19] from the Sequence Read Archive under accession numbers SRX8673336 and SRX8673337. We processed these reads using Spl-IsoQuant-2 and used ScisorSeqR as described earlier to detect genes that exhibit differential isoform usage. Since these datasets were annotated at a finer anatomical level, we mapped their original region annotation to the region annotations we used (**Table S15**). Finally, we detected spatially variable isoforms using Spl-IsoFind as described below.*

11. It is recommended to perform spatial multi-omics integration, such as examining the relationship between the spatial expression patterns of specific isoforms and chromatin accessibility, to explore potential regulatory mechanisms.

We thank the reviewer for this suggestion and agree that this would be interesting to explore, especially as there are known regulatory mechanisms between chromatin accessibility and splicing.

Integrating spatial isoform expression with chromatin accessibility would indeed be a very interesting way to study potential regulatory mechanisms. In the single-cell field, such multi-omic measurements have recently become feasible: ScISOr-ATAC enables joint profiling of gene expression, chromatin accessibility, and splicing (Hu et al., 2025), and SPLONGGET profiles the genome, chromatin accessibility, and the full-length transcriptome (Pančíková et al., 2025). However, both methods have so far only been applied to single-cell or single-nucleus assays and not in the spatial context. Although this is a very interesting avenue, adapting such techniques for spatial assays would require substantial optimization and is therefore beyond the scope of this study.

As an alternative, we explored whether existing publicly available spatial chromatin accessibility data could be integrated with our data. While several spatial chromatin accessibility protocols have been developed (e.g., spatial-ATAC-seq by Deng et al. 2022; Spatial ATAC by Llorens-Bobadilla et al. 2023), to our knowledge, none of these methods has been applied to a similar coronal section of the adult mouse brain. As such, multi-omics integration is currently unfeasible.

We acknowledge that multi-omic integration or multi-omic measurements would be an interesting avenue to explore and added this to the discussion section.

Changes to the manuscript:

- We added the following to the Discussion:
  Interesting extensions to Spl-ISO-Seq2 would include simultaneous measurements of proteins and/or open chromatin as has been done before in single-cells[10,71]. This could reveal regulatory relationships such as the interplay between promoters and alternative transcription-start-site usage as well as the interplay between RNA and protein isoforms.

In summary, this manuscript describes a technically solid and conceptually important advance in spatial isoform profiling, with clear potential impact and broad relevance. With some additional clarifications, slightly more explicit positioning relative to existing methods, and further interrogation to reveal the biological relevance of the isofrm variation it would be well-suited to the goals of Nature Methods as a practically useful and influential methodological article.

Reviewer #3 (Remarks on code availability):

Access to the CodeOcean environment may be restricted for some users. To maximize transparency and reproducibility, I recommend that the authors also provide their code on a universally accessible platform such as GitHub.

We agree with the reviewer that it is important to share code on platforms such as GitHub. Therefore, both Spl-IsoQuant-2 and Spl-IsoFind are publicly available there:

- https://github.com/tilgnerlab/Spl-IsoFind
- https://github.com/algbio/spl-IsoQuant

Reviewers' Comments:

Reviewer #1 (Remarks to the Author):

The authors have provided most answers to the questions raised during the initial review. Some points have now been thoroughly clarified, but a few questions remain unresolved. The main merit of this work is that it extends methodologies already developed by these authors to various sequencing-based spatial transcriptomics technologies.

We thank the reviewer for carefully reviewing our manuscript again.

A clear presentation of the different approaches tested and a detailed description of the corresponding data, accompanied by links to access them, would facilitate future comparisons.

We agree with the reviewer that a detailed description of the data and accessibility is essential for future comparisons. Therefore, we have uploaded all raw sequencing data to the SRA (BioProject ID PRJNA1282707, reviewer link: dataview.ncbi.nlm.nih.gov/object/PRJNA1282707?reviewer=9pbcb9ru2jcd3n2m8tdlf41006) and made all the simulated data, the intermediate analysis files, and additional files needed for processing the data available at Zenodo (simulated data can be found here: doi.org/10.5281/zenodo.19616234, other data: doi.org/10.5281/zenodo.19499423).

Furthermore, we created a reproducibility GitHub that contains the code and all the command lines for reproducing the long-read analysis and the figures in the main text (https://github.com/tilgnerlab/Spl-IsoFind_reproducibility). We thank the reviewer for this valuable suggestion that will promote reproducibility and open science.

The presentation of the data is often unclear or insufficient. For example, all supplementary tables S10 through S14, then S16 through S20, and then S28 through S32 display genes and transcripts as ENSEMBL entries, without gene symbols, which makes any verification very tedious. A column with a genetic symbol should be added. The presentation of these tables should be improved so that they correspond more than the results generated at the end of a computational pipeline, but instead stand as autonomous sources of information. A more compact presentation of these many tables should also be considered.

We thank the reviewer for this suggestion and have now added the gene symbol to these tables. We would like to note that these results are visualized in figures in the main text and supplement as well. E.g., Tables S10–S14 are summarized in Figure 3, Tables S16–S20 in Figure 4, and Tables S28–S32 in Figure 5. The detailed supplementary tables are mainly included as a resource for other researchers who want to utilize these results in their own downstream analysis or look up a specific gene of interest.

Lines 372–373: The authors state that they identified 229 exons exhibiting sex-specific inclusion differences, the majority of which (154 exons) were detected in excitatory neurons (Fig. S21a). However, there is no further description of the results, and the figure is insufficient to evaluate them. Indeed, several transcripts also vary by region/cell, and there is a high risk that this finding is actually masked by other factors.

We agree with the reviewer’s concern that sex-specific differences could be confounded by regional or cell-type composition difference between male and female samples. For our analysis, we chose to group the cells by cell type. We could stratify the cells further by subtype or region, but we would lose statistical power given the limited read depth of long-read sequencing. Nevertheless, for *PPP3CA*, the sex-specific difference remains significant when restricting the analysis to DG excitatory neurons alone (two-sided Fisher's exact test, p = 0.00032), suggesting the effect is not an artifact of regional composition.

We fully acknowledge that with only three individuals per sex the analysis is underpowered and that inter-individual variability in human data is substantial. We therefore applied conservative filtering criteria, requiring concordant effect direction across all individuals with sufficient read coverage. We have been careful to frame this analysis as a preliminary exploration throughout the manuscript, and have added an extra statement to the main text noting the limited sample size. In the discussion, we already mentioned that while the data suggest an intersection between spatial and sex-dependent splicing regulation, this question warrants more dedicated follow-up with larger cohorts.

Changes to the manuscript:

- We added the following sentence to the Results section:
  Although the sample size is limited and, especially for human data, inter-individual variability is expected, this dataset allows an initial exploration of sex-associated splicing patterns.

Minor comments:
Figure 2g is mentioned in the caption but does not appear in the figure.

We thank the reviewer for this accurate observation. We removed the reference to this panel from the main text and figure caption.

Figure S21. The authors state that Pppp3ca has a sex-specific exon. They should include graphs comparing expression in males and females. The current Figure S21-e shows only the relative expression between Pppp3ca-201 and Pppp3ca-202.

We added the absolute expression to Figure S22 (previously Figure S21) for the human hippocampus data stratified by sex and region (**Fig S22e**) and for the mouse spatial data from Sample 1 (AE) (**Fig S22g**). Figure S22e shows that the total read counts are comparable between males and females in the hippocampus. Figure S22g shows that the 202 isoform (exon excluded) is lowly expressed in most parts of the cortex and the CA region of the hippocampus, but shows increased expression in the DG.

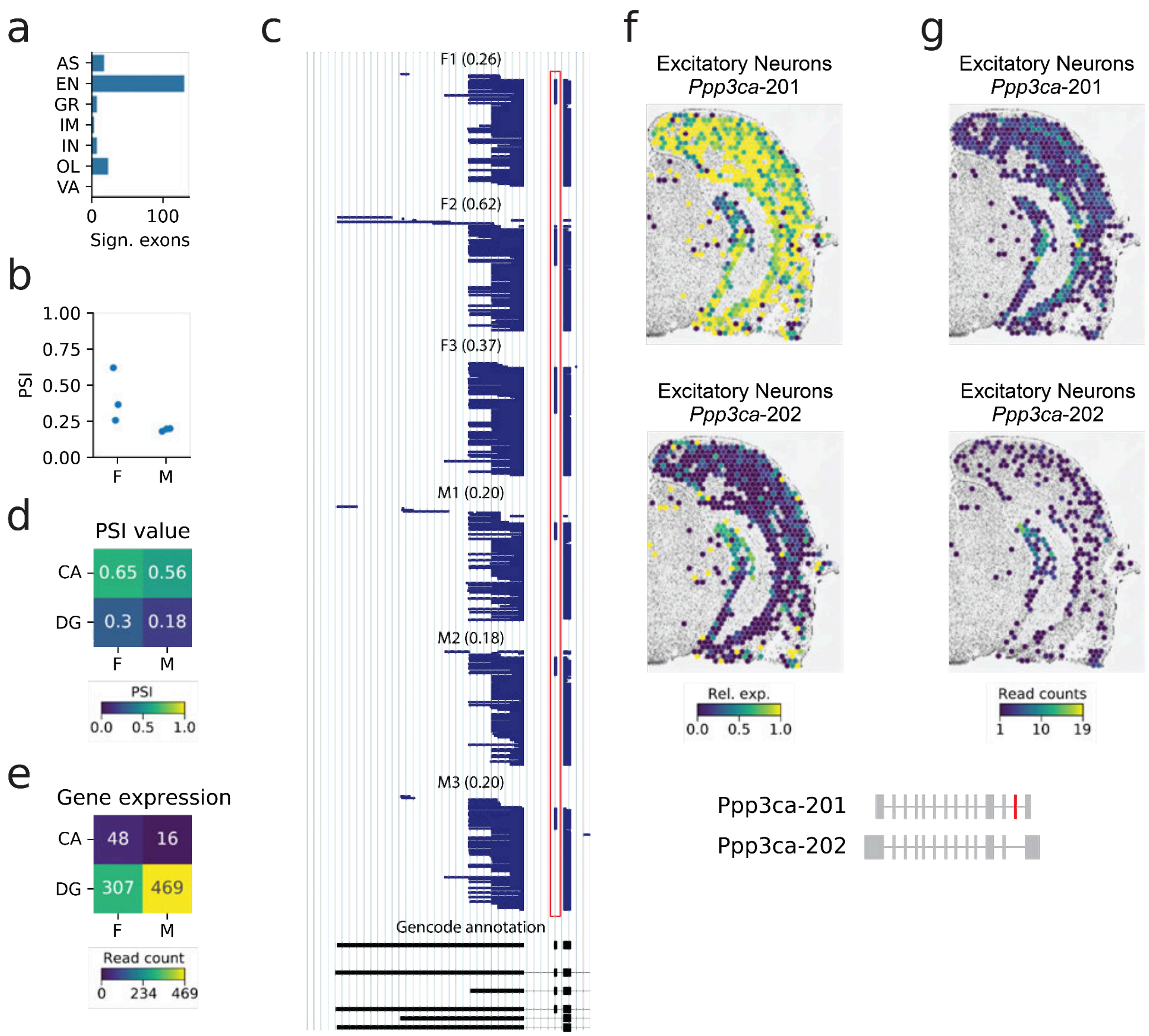

Figure S22. a) Number of significant sex-specific exons per cell type. **b)** PSI value for exon chr4_101029166_101029195_- in *PPP3CA* for each individual in excitatory neurons. **c)** Single-cell long-reads for *PPP3CA* measured in excitatory neurons per individual. Each line in the top six tracks represents a single cDNA molecule. The exon, chr4_101029166_101029195_-, in the red box is sex-specific. The bottom black track shows the Gencode annotation. The PSI value for each individual is indicated between brackets. **d)** PSI value and **e)** gene expression (number of spanning long reads) for exon chr4_101029166_101029195_- in *PPP3CA* in excitatory neurons in the Dentate Gyrus (DG) and Cornu Ammonis (CA) for females (F) and males (M). **f)** Relative and **g)** absolute expression of *Ppp3ca*-201 and *Ppp3ca*-202 in excitatory neurons in Sample 1 (AE). In **f)** every hexagon shows the mean relative expression of the underlying cells, while in **g)** every hexagon shows the sum of the absolute read count of the underlying cells. The ssDNA staining is shown in the background. The schematic diagram at the bottom shows the gene structure of *Ppp3ca*-201 and *Ppp3ca*-202, the two most common isoforms in the data. The exon highlighted in red is the main difference between the two isoforms. This is the same exon that shows sex-specific inclusion differences, noting that PPP3CA is encoded on the positive strand in human and on the negative strand in mouse. AS = Astrocytes, EN = Excitatory Neurons, GR = Granule cells, IM = Immune cells, IN = Inhibitory Neurons, OL = Oligodendrocytes, VA = Vascular cells.

Based on Figure S27a, a k value of 50 appeared more optimal. However, the authors retained a more conservative k value of 10, as they believed that optimization on the same data might lead to overfitting. In my opinion, it would also have been possible to use the settings from one experiment and apply them to another.

We thank the reviewer for this suggestion. Upon reflection, we agree that k=50 is the more appropriate choice, and we have updated all analyses in the revised manuscript accordingly. Switching to k=50 not only increased the number of detected spatially variable isoforms but also improved precision and recall in our simulations (**Fig S13**) and increased the number of SVIs uniquely detected by Spl-IsoFind compared to the predefined region approach (**Fig 4k**, **S26**). One previously described example, *Tnnc1*, is no longer significant in the revised analysis as the isoform does not meet the minimum read count threshold required for testing with k=50 (<250 counts). We have therefore replaced this example with *Ighm*.

Figure S38 is poorly described and likely unnecessary.

We agree with the reviewer and removed this figure from the supplement.

Reviewer #2 (Remarks to the Author):

The authors have addressed all of my technical concerns.

Reviewer #3 (Remarks to the Author):

The authors have provided a clear and comprehensive response to the initial reviews, and my previous concerns have been largely addressed. The revised manuscript presents a robust and valuable technical framework.
Regarding my earlier points, I acknowledge the technical constraints involved in achieving high-resolution sub-clustering of excitatory neurons with the current modality and accept the authors' rationale. Furthermore, the identification of novel Gad2 isoforms is a particularly compelling addition. Such findings elegantly underscore the biological potential of this technology, as it captures subtle transcriptomic variations—often overlooked by traditional short-read sequencing—that may drive fundamental functional or evolutionary differences.
However, while these specific examples are excellent, the narrative remains predominantly case-driven. To maximize the utility of this work for the broader community, it would be highly beneficial to provide a more comprehensive, global perspective. I recommend adding a global summary or high level functional analysis to delineate the overall landscape of isoform differences across distinct brain regions and major cell types. For instance, a concise overview may highlight the functional categories, biological pathways, or alternative splicing events that are predominantly enriched in different spatial domains. It would also be valuable to clarify whether specific brain regions or cell types display greater isoform diversity, as this would provide important insights into the evolutionary forces and functional constraints that shape these regions or cell types.
Recognizing that this is fundamentally a methods paper, I do not expect an exhaustive downstream biological investigation or perfectly resolved mechanistic stories. The goal is simply to provide readers with a realistic "big picture" of the global biological variation

captured by this technology. Providing this overarching context will serve as a valuable roadmap for future users. Pending this minor, computation-only addition, I am supportive of publication.

We thank the reviewer for these kind words and the constructive suggestion. To add a bigger picture of global biological variation, we characterized the structural differences between spatially variable isoforms (SVIs) and non-SVIs across all expressed genes.

For each gene, we compared the annotated transcript structures of all expressed isoforms (≥20 reads) and classified each pair into nine splicing event categories:

- alternative TSS;
- alternative first exon;
- alternative polyadenylation site;
- alternative last exon;
- exon skipping;
- mutually exclusive exons;
- alternative donor site;
- alternative acceptor site;
- intron retention.

Pairs can have multiple co-occurring splicing events. For every pair, we also annotated whether none, one, or both of the isoforms are spatially variable, yielding three comparison types: SVI vs SVI, SVI vs non-SVI, and non-SVI vs non-SVI. To avoid genes with many isoforms dominating the analysis, we averaged all event frequencies per gene and comparison type during downstream analysis.

This analysis revealed three main findings. First, when comparing the number of splicing events between isoforms, pairs of SVIs showed fewer co-occurring splicing events than SVIs vs non-SVIs or non-SVIs compared to each other (**Fig S15a**). This suggests that SVIs within the same gene tend to be structurally more similar to each other than to non-SVIs. Second, exon skipping is significantly enriched in SVI vs non-SVI comparisons relative to background non-SVI comparisons (two-sided Mann-Whitney U, FDR-adjusted $p = 0.038$, **Fig S15b-c**). Third, when exon skipping distinguishes an SVI from a non-SVI, the SVI more often includes the exon (**Fig S15d**), suggesting that region-specific splicing factor activity promotes exon inclusion more often.

We have added this analysis to the manuscript.

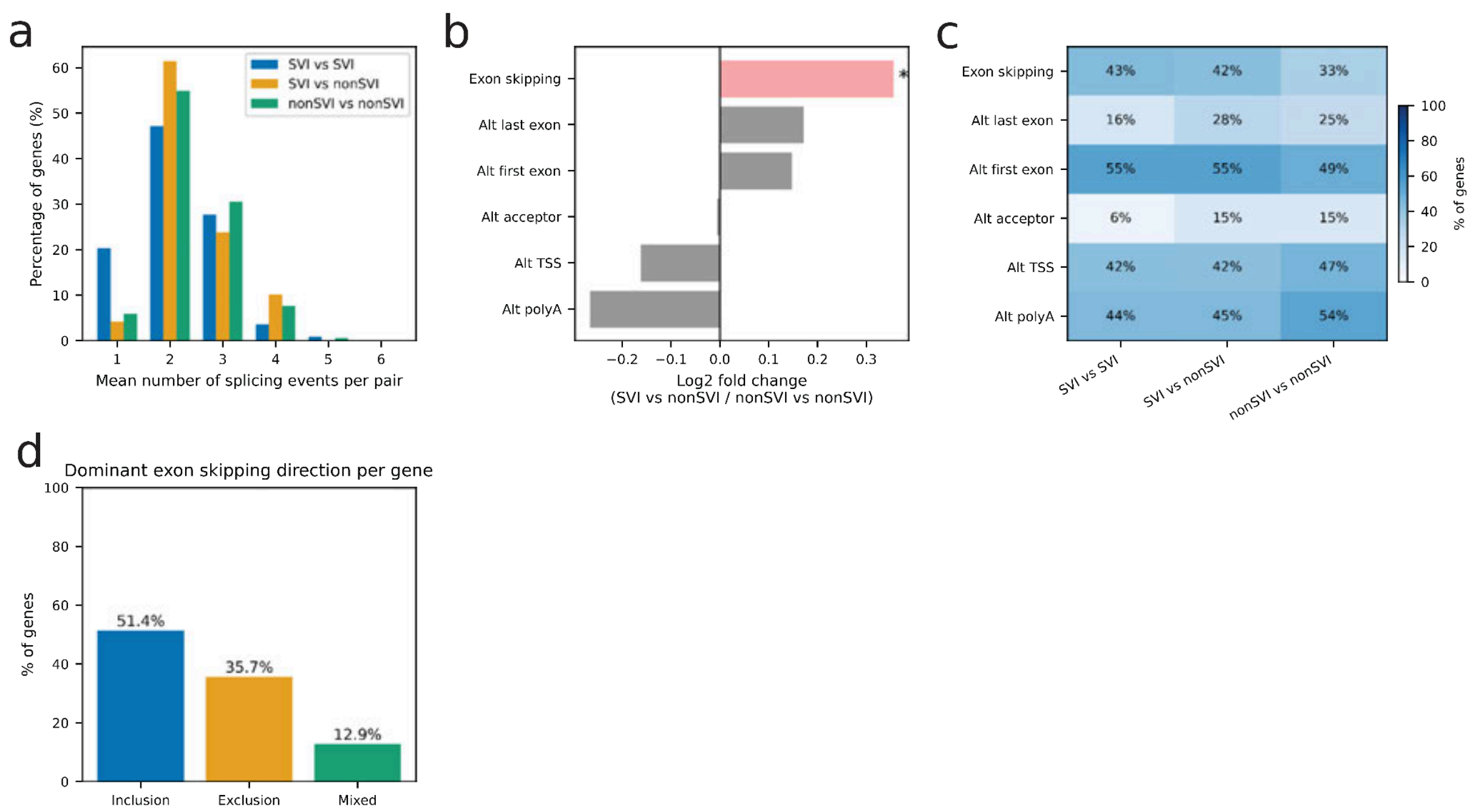

Figure S15. a) Distribution of the mean number of co-occurring splicing events per isoform pair, grouped by comparison type (SVI vs SVI, SVI vs nonSVI, nonSVI vs nonSVI). Values are averaged per gene to avoid bias from genes with large isoform repertoires. **b)** Log2 fold change of splicing event frequencies in SVI vs nonSVI pairs relative to nonSVI vs nonSVI pairs. The highlighted bar (exon skipping) indicates a statistically significant enrichment (Mann-Whitney U test, FDR-adjusted p = 0.038). Event categories with a mean frequency below 10% across all comparison types were excluded from statistical testing. **c)** Heatmap showing the percentage of genes in which each splicing event type was detected, stratified by comparison type. Values represent gene-level means averaged across all pairwise comparisons within each comparison type. **d)** Exon skipping direction per gene (for genes with at least one SVI vs nonSVI pair showing an exon skipping event). For each gene, the direction was defined as the direction (inclusion or exclusion in the SVI) observed in the majority of pairs. Genes with equal numbers of inclusion and exclusion pairs were classified as mixed.

Changes to the manuscript:

- We added Figure S15

- We added the following text to the Results:

  To characterize the splicing mechanisms underlying these SVIs, we systematically compared the annotated transcript structures of all expressed isoforms (≥20 reads in Sample1+2 (AE)) per gene, classifying each pairwise comparison into nine splicing events (Methods). SVIs are structurally more similar to each other than to non-SVIs, showing fewer co-occurring splicing events per pair (Fig S15a). When comparing SVIs to non-SVIs, exon skipping was the only significantly enriched event type relative to background (two-sided Mann-Whitney U, FDR-adjusted p = 0.038) (Fig S15b-c). In the majority of cases the SVI represented the exon-included rather than the exon-excluded isoform (51% vs 36% of genes) (Fig S15d). This

suggests that spatial isoform variation is predominantly driven by discrete, region-specific exon inclusion events rather than broad transcriptomic remodeling.

- We added the following section to the Methods:

  Splicing event classification of expressed isoforms
  To understand what structurally distinguishes spatially variable isoforms from other expressed isoforms, we compared the transcript structures of all expressed isoforms (≥20 reads in the Sample1+2 (AE) data) for each gene. For every pair of isoforms within a gene, we classified the structural differences into nine categories: alternative TSS, alternative first exon, alternative polyadenylation site, alternative last exon, exon skipping, alternative donor site, alternative acceptor site, intron retention, and mutually exclusive exons. Pairs can have multiple co-occurring splicing events.
  For the first and last exon categories, we distinguished between a shift in the transcription start or polyadenylation site versus the use of a completely different exon. Specifically, if two isoforms shared the 3' boundary of their first exon but differed at the 5' end, the event was classified as an alternative TSS. However, if the 3' boundary itself differed, it was classified as an alternative first exon. The same logic was applied in reverse for the last exon. Intron retention required that a single exon in one isoform exactly spanned two or more exons and their intervening sequence in the other. Mutually exclusive exons were defined as exon pairs, one in each isoform, occupying the same position between identical flanking exons. Alternative donor and acceptor sites were identified as internal exon pairs sharing one splice site but differing at the other. Exon skipping was assigned to any remaining internal exon present in one isoform but absent in the other, after excluding exons already explained by the above categories. Exon coordinates were extracted from the extended annotation GTF generated by IsoQuant. All comparisons were strand-aware.
  Each pair was labeled by whether zero, one, or both isoforms were spatially variable, giving three comparison types: SVI vs SVI, SVI vs non-SVI, and non-SVI vs non-SVI. Since genes with many isoforms contribute more pairs, we averaged event frequencies across all pairs within each gene and comparison type before any statistical comparisons. Differences between comparison types were tested using two-sided Mann-Whitney U tests with Benjamini-Hochberg FDR correction. Event types with a mean frequency below 10% across all three comparison types were excluded from testing due to low power. For exon skipping events in SVI vs non-SVI pairs, the direction was determined by exon count: the isoform with more internal exons was considered the exon-included isoform. Per gene, the dominant direction was the one observed in the majority of pairs; genes with equal counts in both directions were labeled mixed.

Reviewer #3 (Remarks on code availability):

The authors have provided a high-quality code repository. Notably, the documentation is comprehensive and provides a clear, step-by-step guide that ensures a smooth installation process. The tutorial is functional and error-free; the successful execution of all provided examples demonstrates the system's stability and ease of use for the broader community.

We thank the reviewer for these kind words. We also added a Reproducibility GitHub now that contains the code and the command lines to reproduce the long-read analysis and the figures in the main text: https://github.com/tilgnerlab/Spl-IsoFind_reproducibility.
