# Supplementary material for: Spatial isoform sequencing at single-cell resolution reveals cell-type-specific spatial isoform variability in multiple brain cell types
Source: Nat Methods. 2026 Sep 4;23(9):1869–81. doi: 10.1038/s41592-026-03211-w (PMC13545012; doi:10.1038/s41592-026-03211-w)
Supplement: Supplementary file 4 [file 41592_2026_3211_MOESM5_ESM.pdf]

Source data for Figure 3f

Filename: 2025-04-01 - 22.42.24.D1000

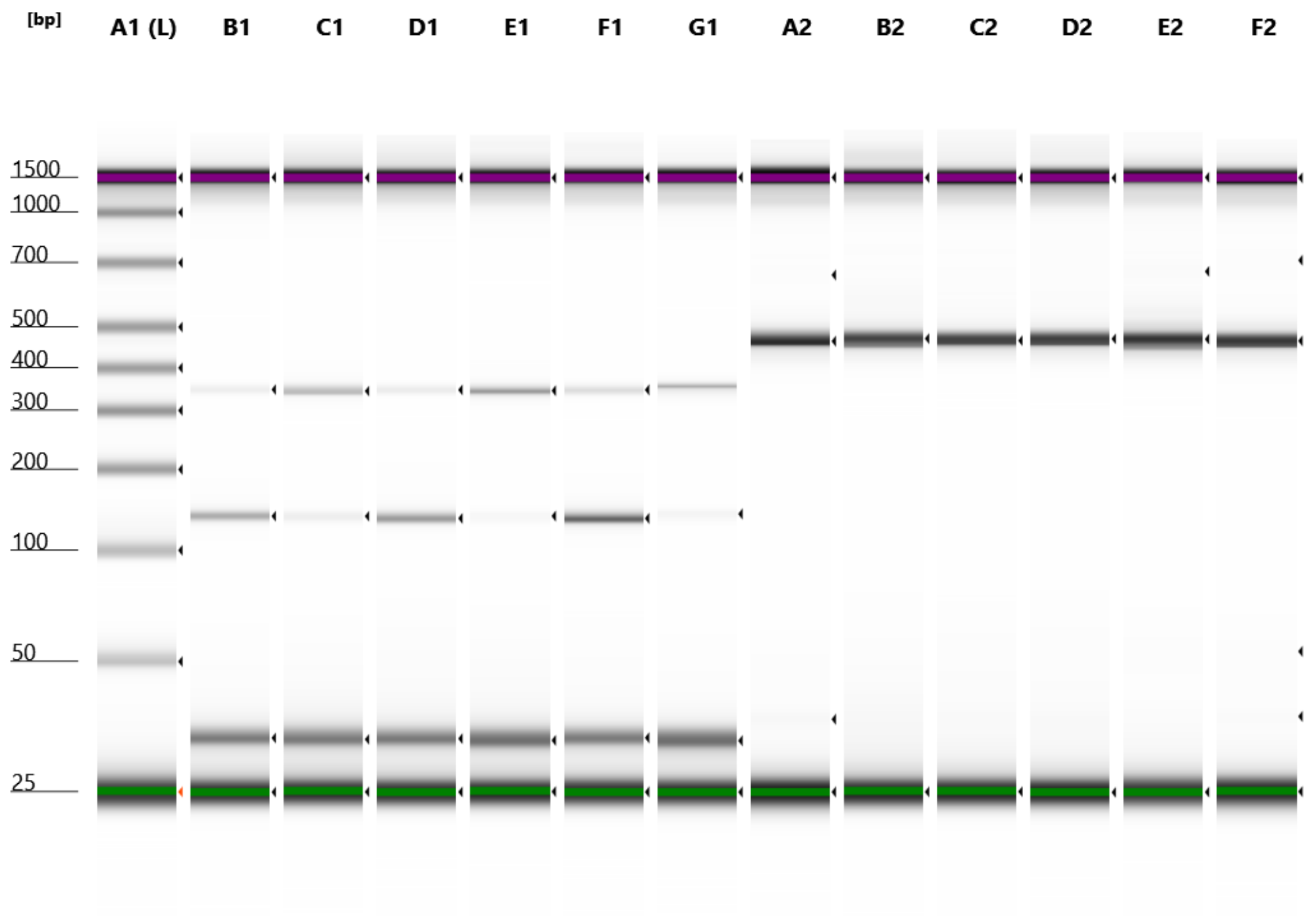

Default image (Contrast 100%)

Sample Info

Well	Conc. [ng/µl]	Sample Description	Alert	Observations
A1	7.67	Ladder		Ladder
B1	2.69	phactr1 m3 0		
C1	2.75	phactr1 m3 mb		
D1	2.94	phactr1 m7 o		
E1	3.10	phactr1 m7 mb		
F1	3.53	phactr1 m8 o		
G1	2.66	phactr1 mb mb		
A2	2.63	GAPDH m3 o		
B2	2.76	gapdh m3 b		
C2	2.54	gapdh m7 o		
D2	2.64	gapdh m7 mb		
E2	3.59	gapdh m8 o		
F2	2.97	gapdh m8 mb		

© Agilent Technologies, Inc. 2021

Source data for Figure 3f

A1: Ladder

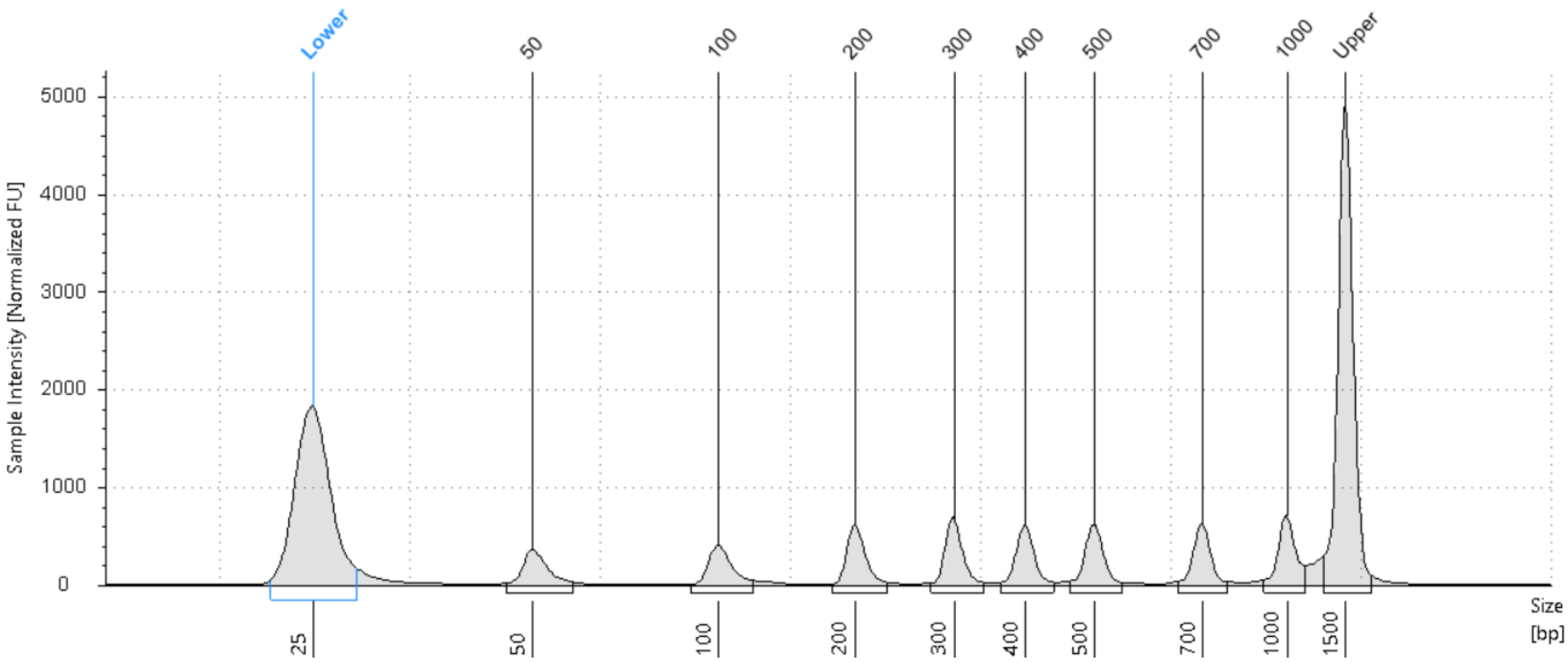

Sample Table

Well	Conc. [ng/µl]	Sample Description	Alert	Observations
A1	7.67	Ladder		Ladder

Peak Table

Size [bp]	Calibrated Conc. [ng/µl]	Assigned Conc. [ng/µl]	Peak Molarity [nmol/l]	% Integrated Area	Peak Comment	Observations
25	5.66	-	349	-		Lower Marker
50	0.777	-	23.9	10.13		
100	0.822	-	12.6	10.72		
200	1.01	-	7.80	13.23		
300	1.06	-	5.42	13.78		
400	0.975	-	3.75	12.72		
500	1.02	-	3.13	13.27		
700	0.954	-	2.10	12.44		
1000	1.05	-	1.62	13.71		
1500	6.50	6.50	6.67	-		Upper Marker

© Agilent Technologies, Inc. 2021

Source data for Figure 3f

B1: phactr1 m3 0

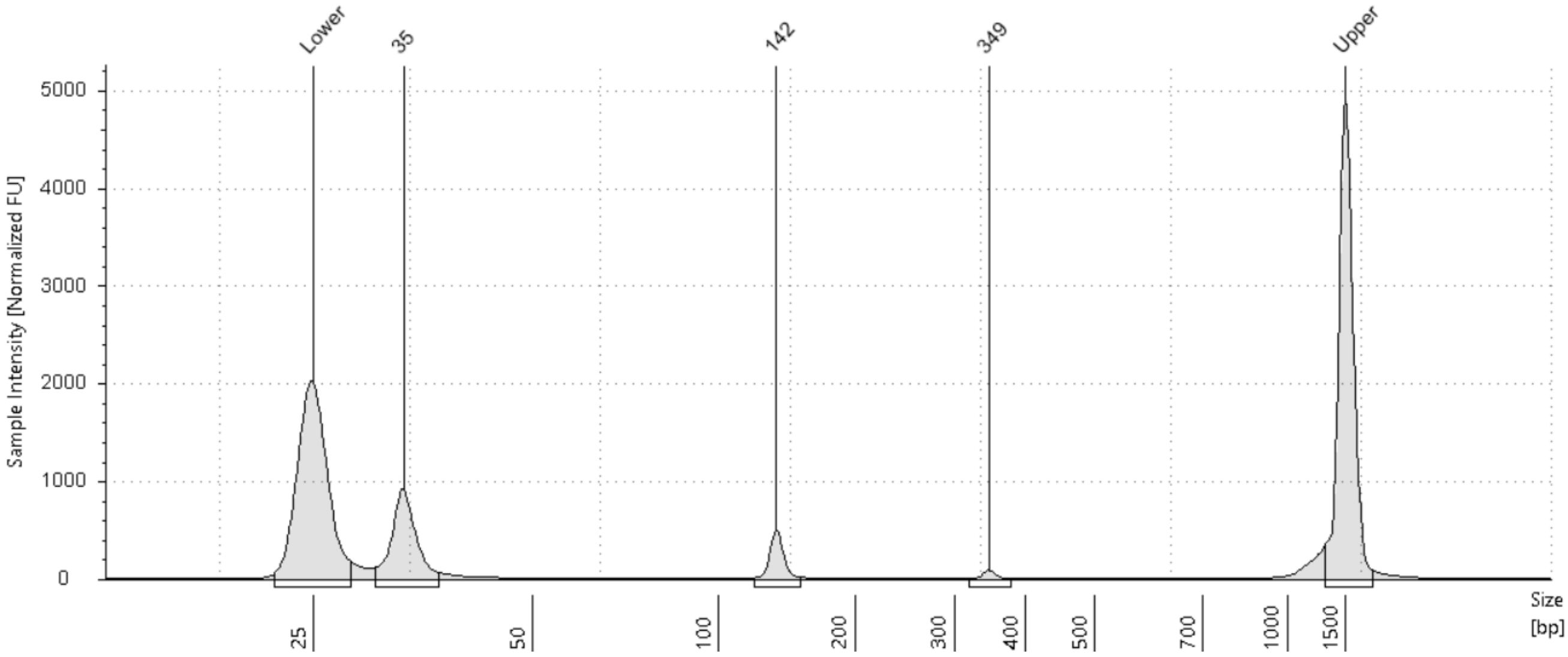

Sample Table

Well	Conc. [ng/µl]	Sample Description	Alert	Observations
B1	2.69	phactr1 m3 0		

Peak Table

Size [bp]	Calibrated Conc. [ng/µl]	Assigned Conc. [ng/µl]	Peak Molarity [nmol/l]	% Integrated Area	Peak Comment	Observations
25	5.50	-	338	-		Lower Marker
35	1.96	-	85.0	72.63		
142	0.627	-	6.78	23.28		
349	0.110	-	0.485	4.09		
1500	6.50	6.50	6.67	-		Upper Marker

© Agilent Technologies, Inc. 2021

Source data for Figure 3f

C1: phactr1 m3 mb

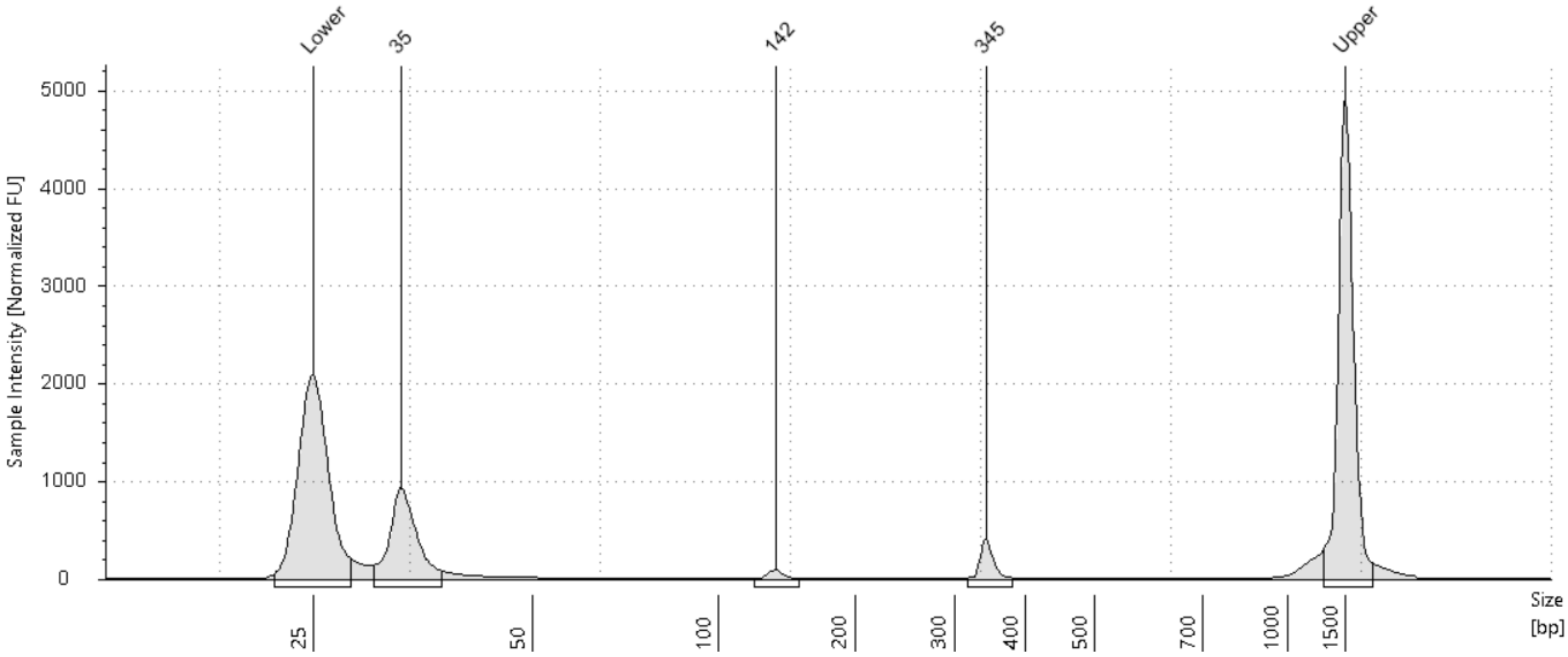

Sample Table

Well	Conc. [ng/µl]	Sample Description	Alert	Observations
C1	2.75	phactr1 m3 mb		

Peak Table

Size [bp]	Calibrated Conc. [ng/µl]	Assigned Conc. [ng/µl]	Peak Molarity [nmol/l]	% Integrated Area	Peak Comment	Observations
25	5.47	-	337	-		Lower Marker
35	2.14	-	94.0	77.94		
142	0.127	-	1.38	4.62		
345	0.479	-	2.13	17.44		
1500	6.50	6.50	6.67	-		Upper Marker

© Agilent Technologies, Inc. 2021

Source data for Figure 3f

D1: phactr1 m7 o

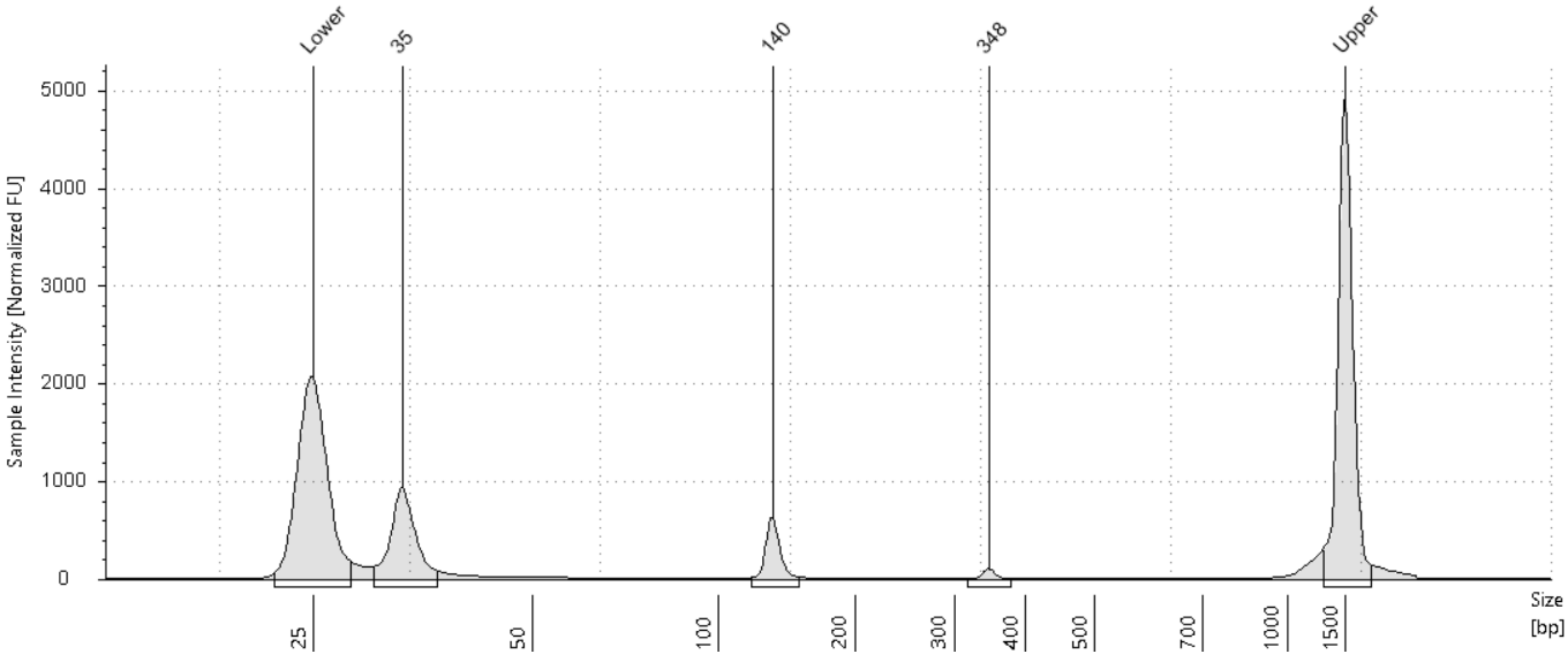

Sample Table

Well	Conc. [ng/µl]	Sample Description	Alert	Observations
D1	2.94	phactr1 m7 o		

Peak Table

Size [bp]	Calibrated Conc. [ng/µl]	Assigned Conc. [ng/µl]	Peak Molarity [nmol/l]	% Integrated Area	Peak Comment	Observations
25	5.51	-	339	-		Lower Marker
35	2.02	-	88.2	68.74		
140	0.796	-	8.78	27.11		
348	0.122	-	0.538	4.15		
1500	6.50	6.50	6.67	-		Upper Marker

© Agilent Technologies, Inc. 2021

Source data for Figure 3f

E1: phactr1 m7 mb

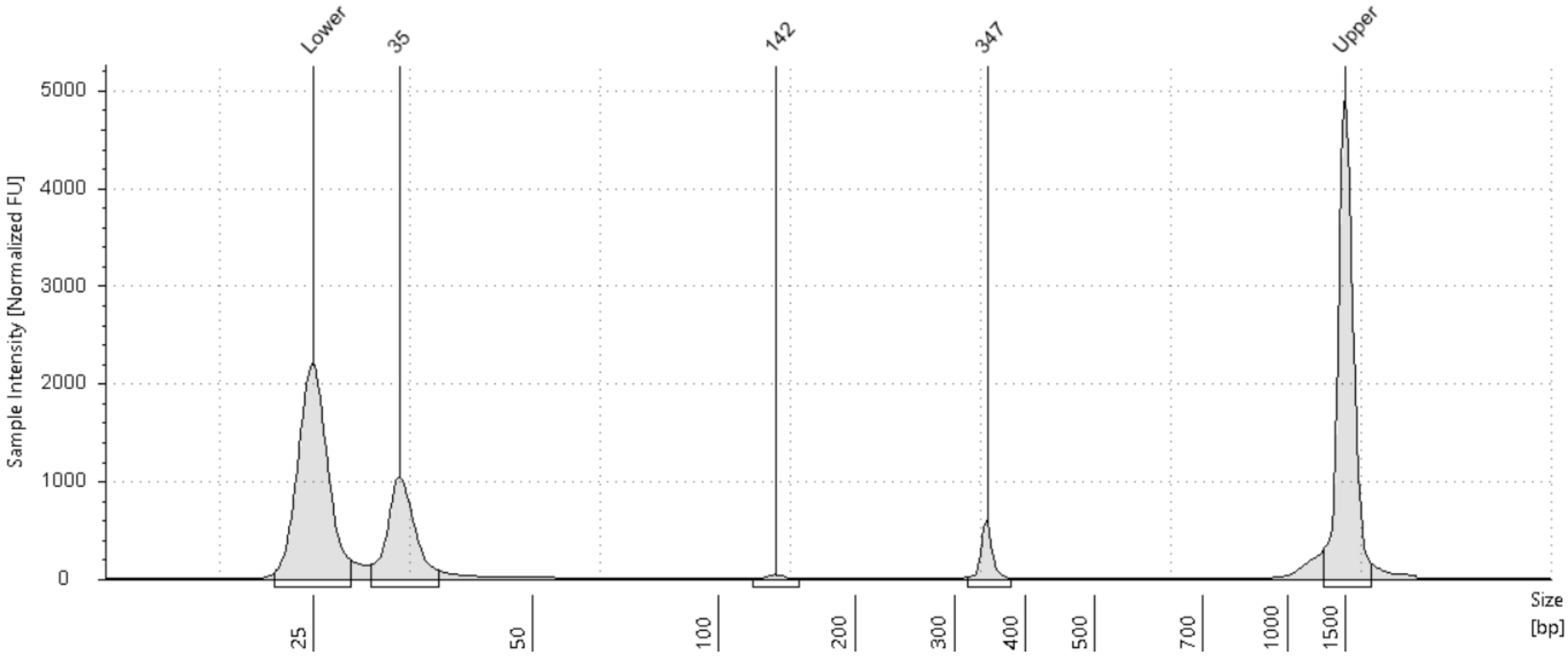

Sample Table

Well	Conc. [ng/µl]	Sample Description	Alert	Observations
E1	3.10	phactr1 m7 mb		

Peak Table

Size [bp]	Calibrated Conc. [ng/µl]	Assigned Conc. [ng/µl]	Peak Molarity [nmol/l]	% Integrated Area	Peak Comment	Observations
25	5.68	-	349	-		Lower Marker
35	2.43	-	108	78.63		
142	0.0666	-	0.720	2.15		
347	0.595	-	2.64	19.22		
1500	6.50	6.50	6.67	-		Upper Marker

© Agilent Technologies, Inc. 2021

Source data for Figure 3f

F1: phactr1 m8 o

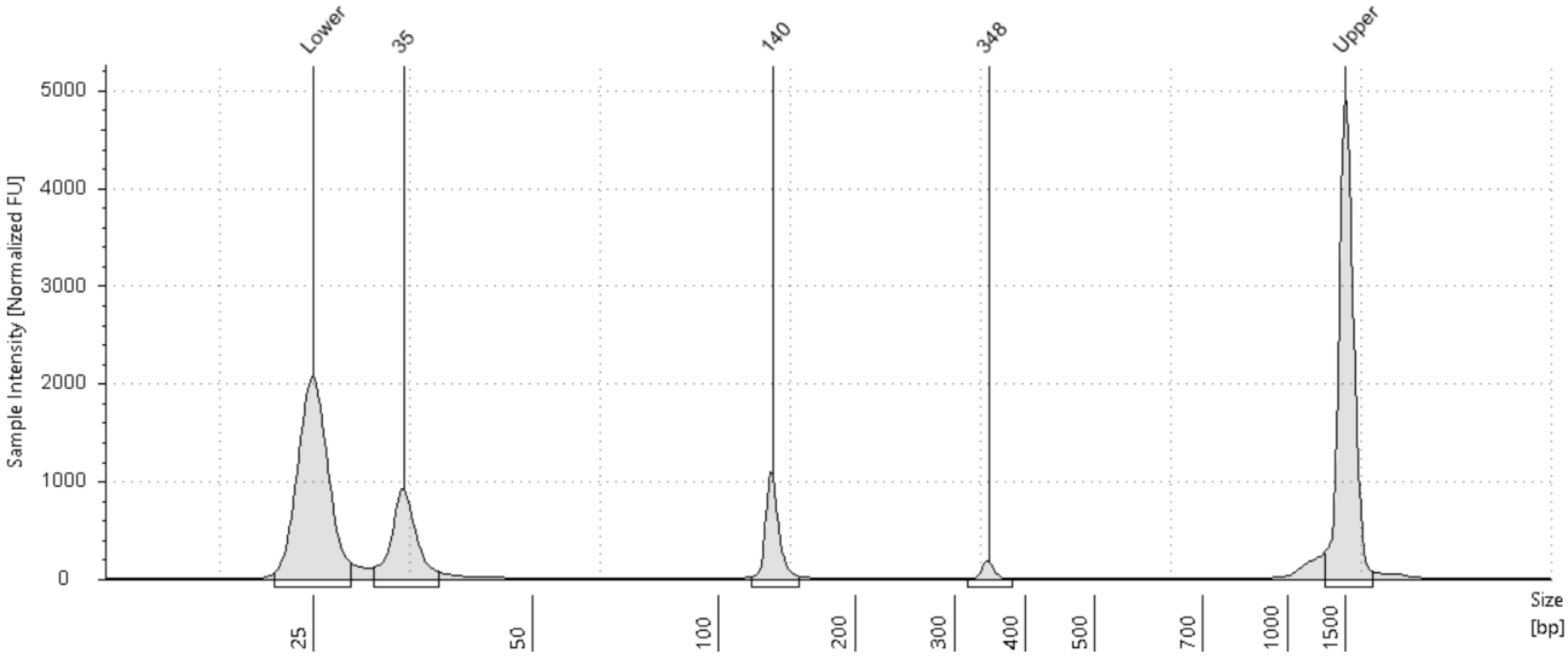

Sample Table

Well	Conc. [ng/µl]	Sample Description	Alert	Observations
F1	3.53	phactr1 m8 o		

Peak Table

Size [bp]	Calibrated Conc. [ng/µl]	Assigned Conc. [ng/µl]	Peak Molarity [nmol/l]	% Integrated Area	Peak Comment	Observations
25	5.66	-	348	-		Lower Marker
35	2.02	-	87.8	57.22		
140	1.29	-	14.2	36.60		
348	0.218	-	0.963	6.18		
1500	6.50	6.50	6.67	-		Upper Marker

© Agilent Technologies, Inc. 2021

Source data for Figure 3f

G1: phactr1 mb mb

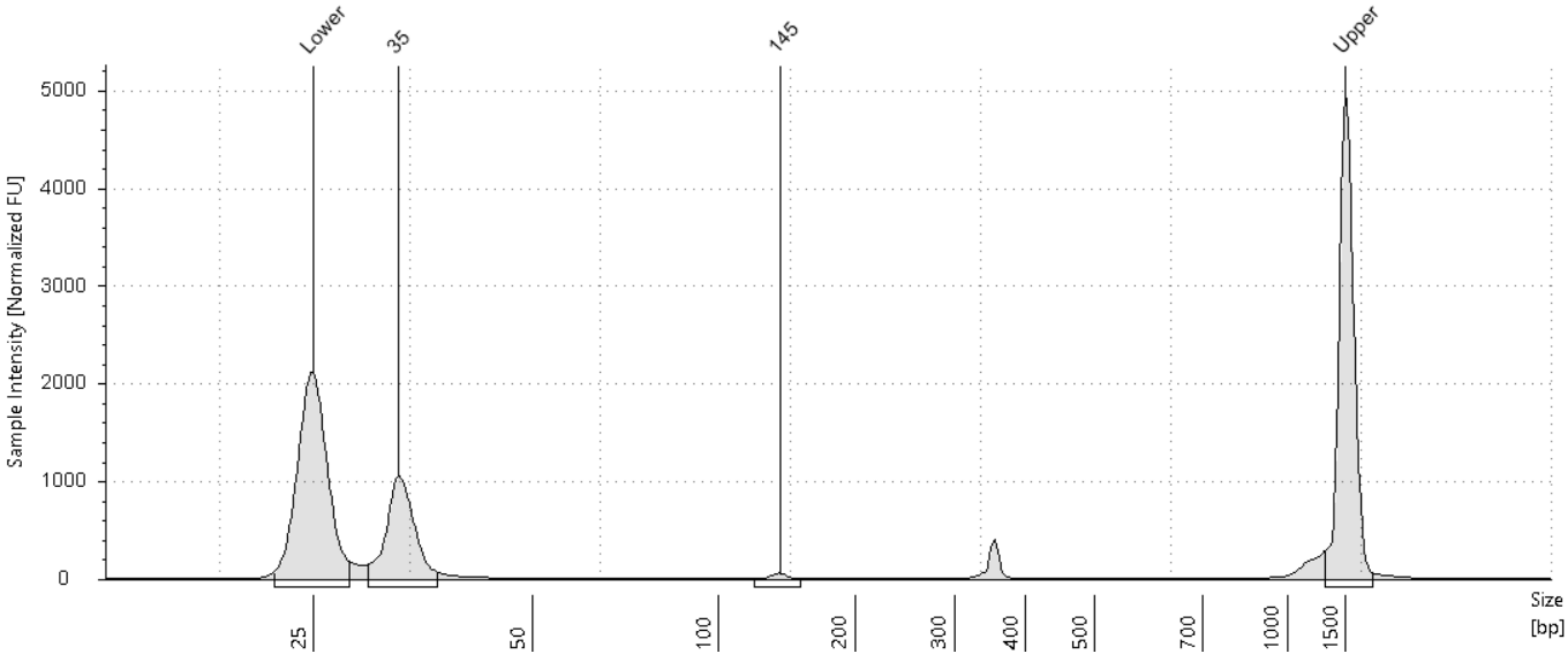

Sample Table

Well	Conc. [ng/µl]	Sample Description	Alert	Observations
Gl	2.66	phactr1 mb mb		

Peak Table

Size [bp]	Calibrated Conc. [ng/µl]	Assigned Conc. [ng/µl]	Peak Molarity [nmol/l]	% Integrated Area	Peak Comment	Observations
25	5.58	-	343	-		Lower Marker
35	2.57	-	114	96.81		
145	0.0848	-	0.900	3.19		
1500	6.50	6.50	6.67	-		Upper Marker

© Agilent Technologies, Inc. 2021

Source data for Figure 3f

A2: GAPDH m3 o

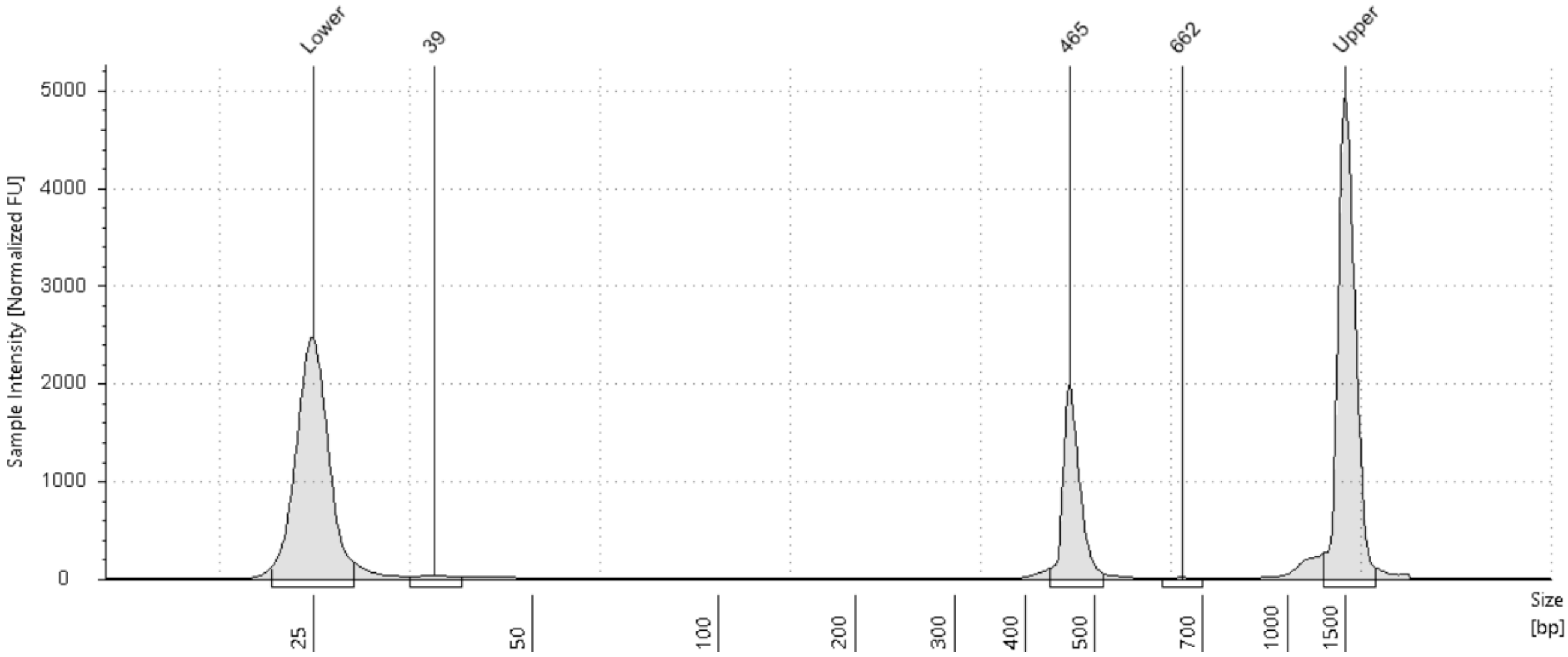

Sample Table

Well	Conc. [ng/µl]	Sample Description	Alert	Observations
A2	2.63	GAPDH m3 o		

Peak Table

Size [bp]	Calibrated Conc. [ng/µl]	Assigned Conc. [ng/µl]	Peak Molarity [nmol/l]	% Integrated Area	Peak Comment	Observations
25	6.02	-	371	-		Lower Marker
39	0.102	-	4.02	3.87		
465	2.50	-	8.25	94.83		
662	0.0343	-	0.0796	1.30		
1500	6.50	6.50	6.67	-		Upper Marker

© Agilent Technologies, Inc. 2021

Source data for Figure 3f

B2: gapdh m3 b

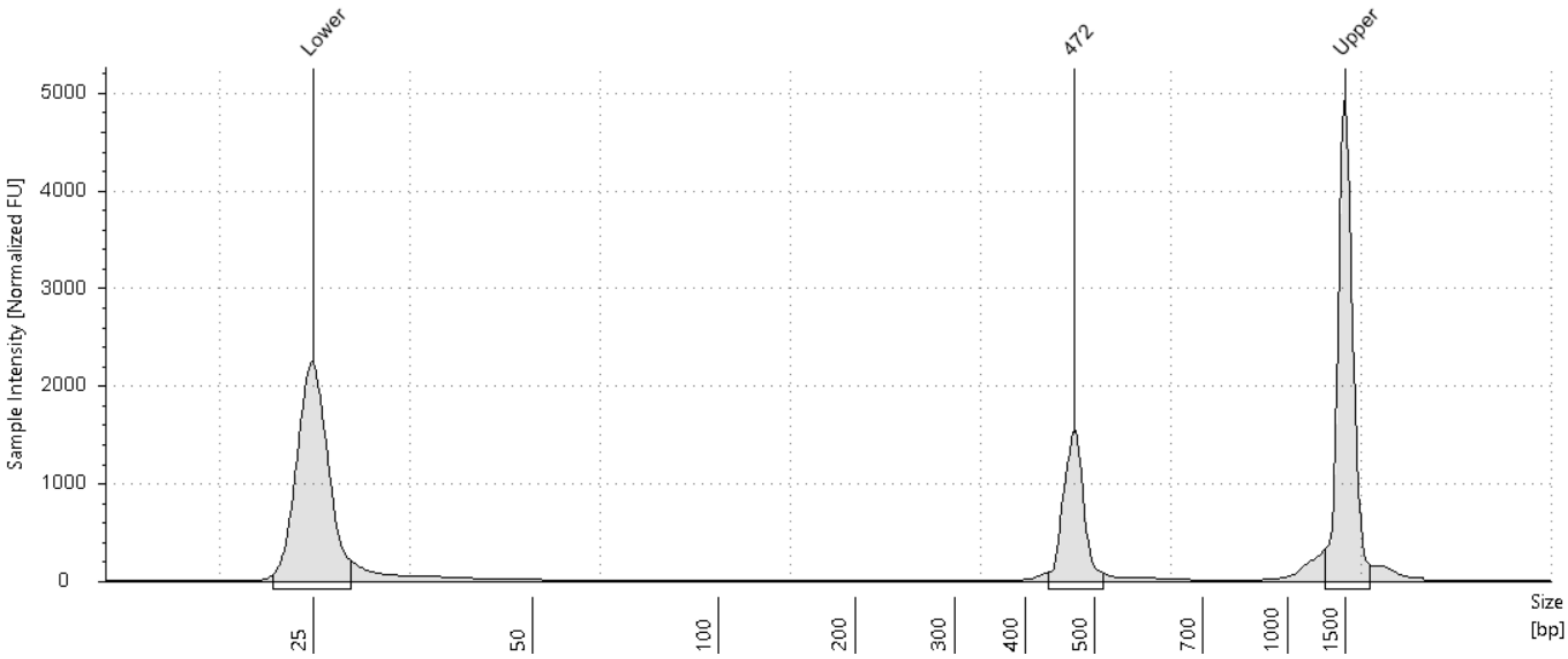

Sample Table

Well	Conc. [ng/µl]	Sample Description	Alert	Observations
B2	2.76	gapdh m3 b		

Peak Table

Size [bp]	Calibrated Conc. [ng/µl]	Assigned Conc. [ng/µl]	Peak Molarity [nmol/l]	% Integrated Area	Peak Comment	Observations
25	6.21	-	382	-		Lower Marker
472	2.76	-	9.00	100.00		
1500	6.50	6.50	6.67	-		Upper Marker

© Agilent Technologies, Inc. 2021

Source data for Figure 3f

C2: gapdh m7 o

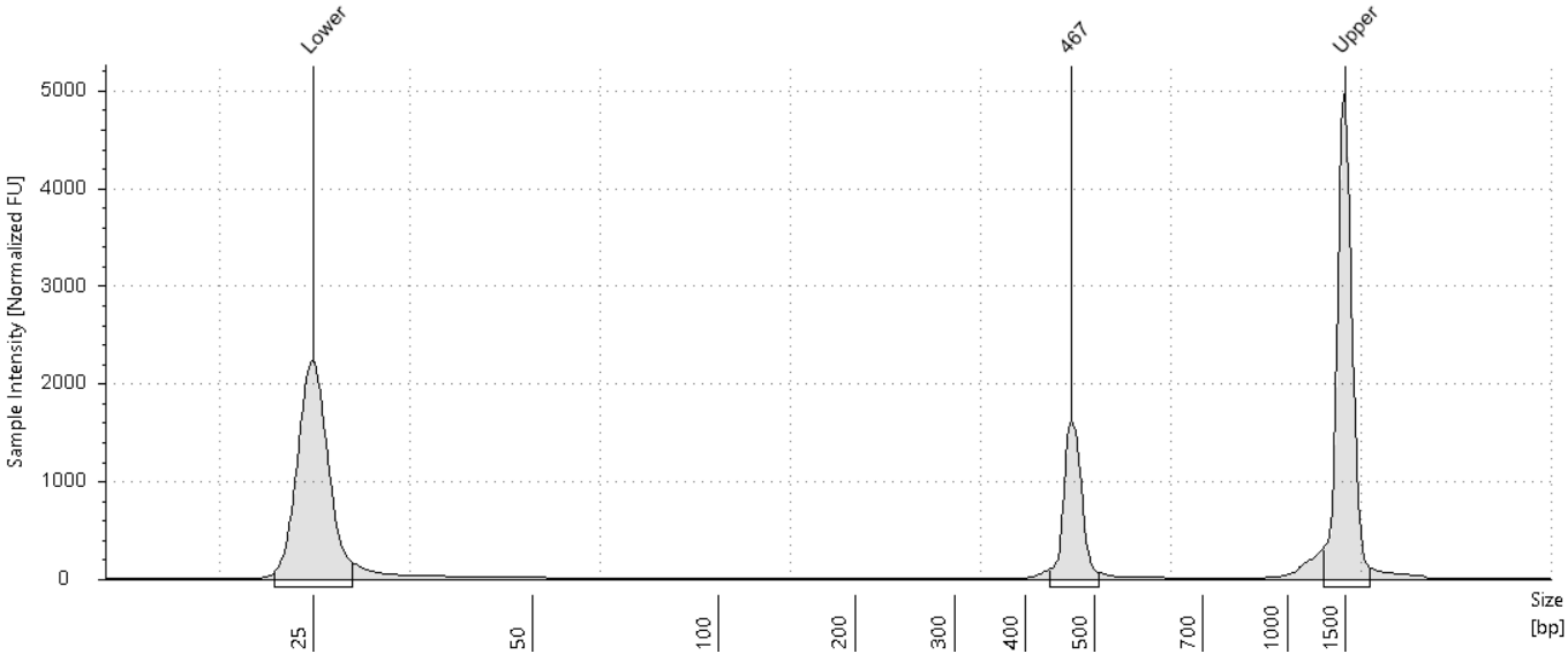

Sample Table

Well	Conc. [ng/µl]	Sample Description	Alert	Observations
C2	2.54	gapdh m7 o		

Peak Table

Size [bp]	Calibrated Conc. [ng/µl]	Assigned Conc. [ng/µl]	Peak Molarity [nmol/l]	% Integrated Area	Peak Comment	Observations
25	5.98	-	368	-		Lower Marker
467	2.54	-	8.37	100.00		
1500	6.50	6.50	6.67	-		Upper Marker

 © Agilent Technologies, Inc. 2021

Source data for Figure 3f

D2: gapdh m7 mb

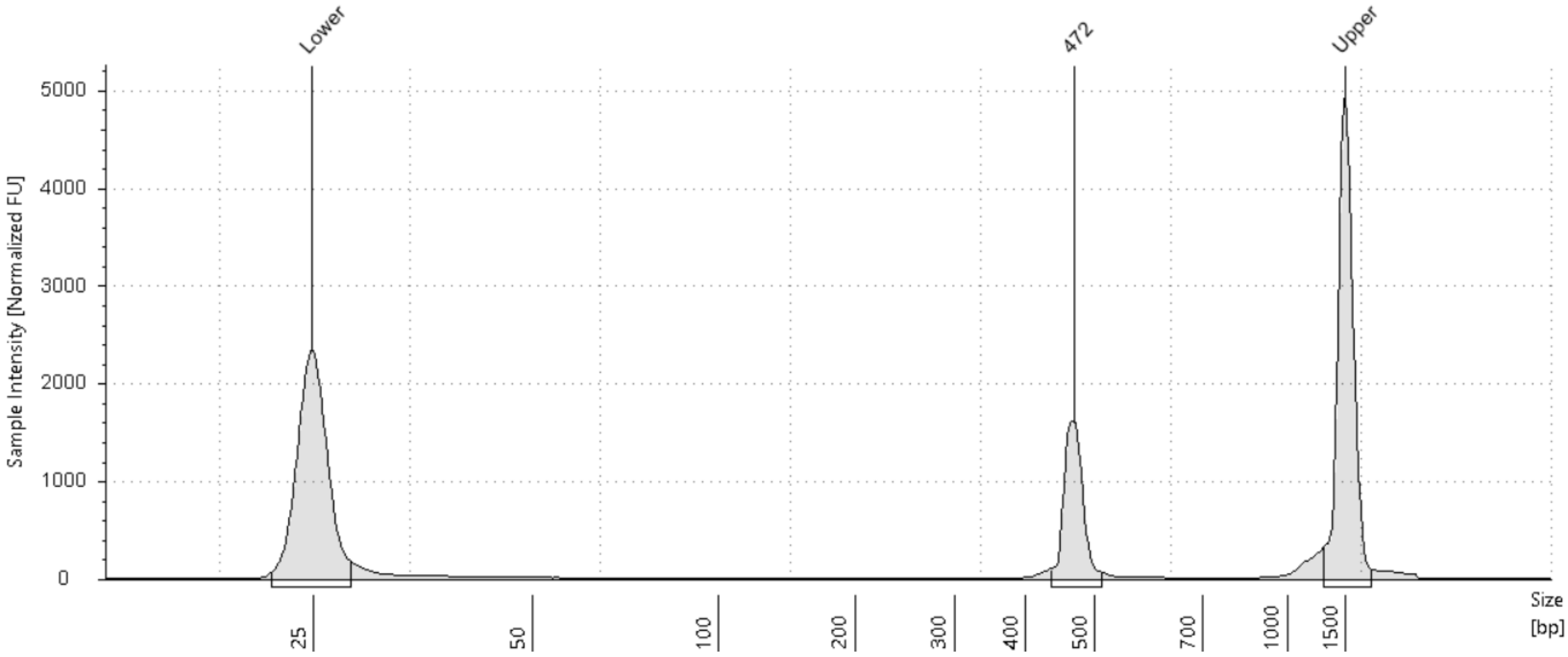

Sample Table

Well	Conc. [ng/µl]	Sample Description	Alert	Observations
D2	2.64	gapdh m7 mb		

Peak Table

Size [bp]	Calibrated Conc. [ng/µl]	Assigned Conc. [ng/µl]	Peak Molarity [nmol/l]	% Integrated Area	Peak Comment	Observations
25	6.07	-	374	-		Lower Marker
472	2.64	-	8.61	100.00		
1500	6.50	6.50	6.67	-		Upper Marker

© Agilent Technologies, Inc. 2021

Source data for Figure 3f

E2: gapdh m8 o

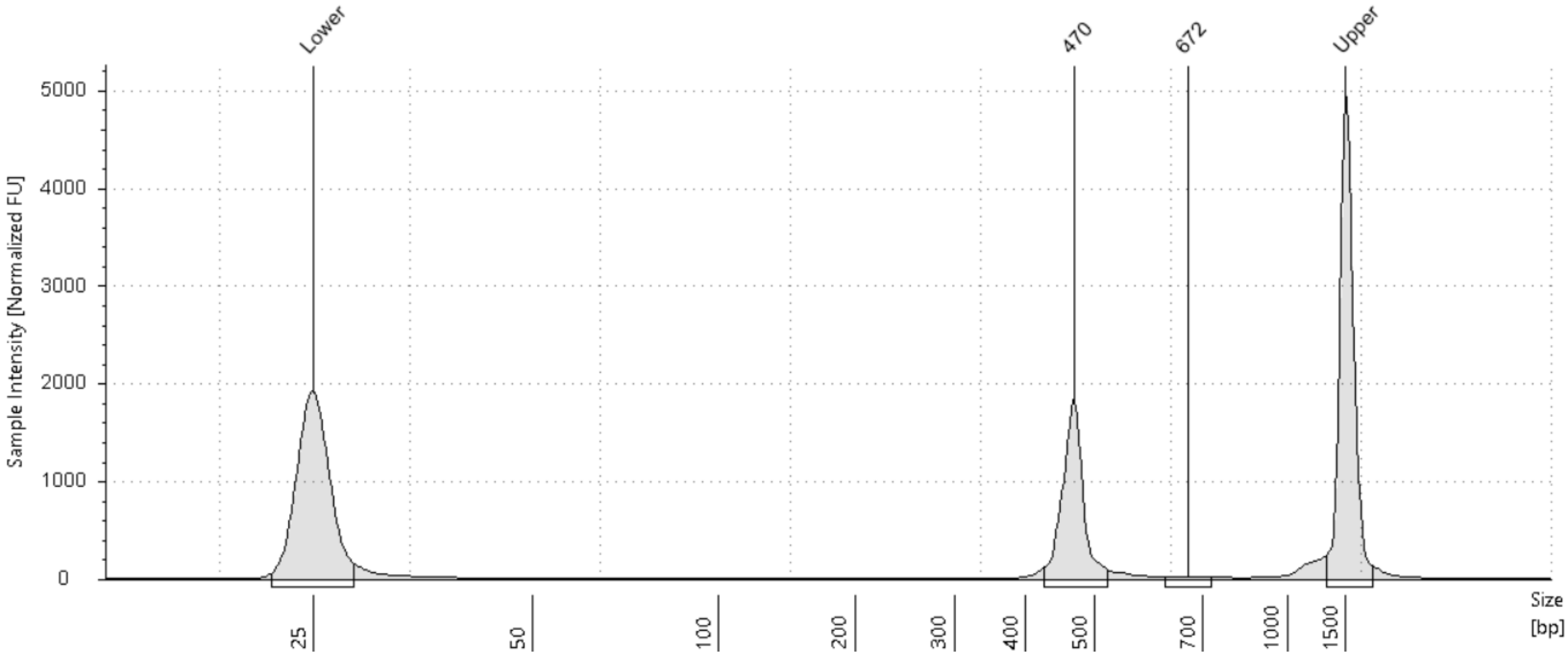

Sample Table

Well	Conc. [ng/µl]	Sample Description	Alert	Observations
E2	3.59	gapdh m8 o		

Peak Table

Size [bp]	Calibrated Conc. [ng/µl]	Assigned Conc. [ng/µl]	Peak Molarity [nmol/l]	% Integrated Area	Peak Comment	Observations
25	6.00	-	369	-		Lower Marker
470	3.51	-	11.5	97.69		
672	0.0830	-	0.190	2.31		
1500	6.50	6.50	6.67	-		Upper Marker

© Agilent Technologies, Inc. 2021

Source data for Figure 3f

F2: gapdh m8 mb

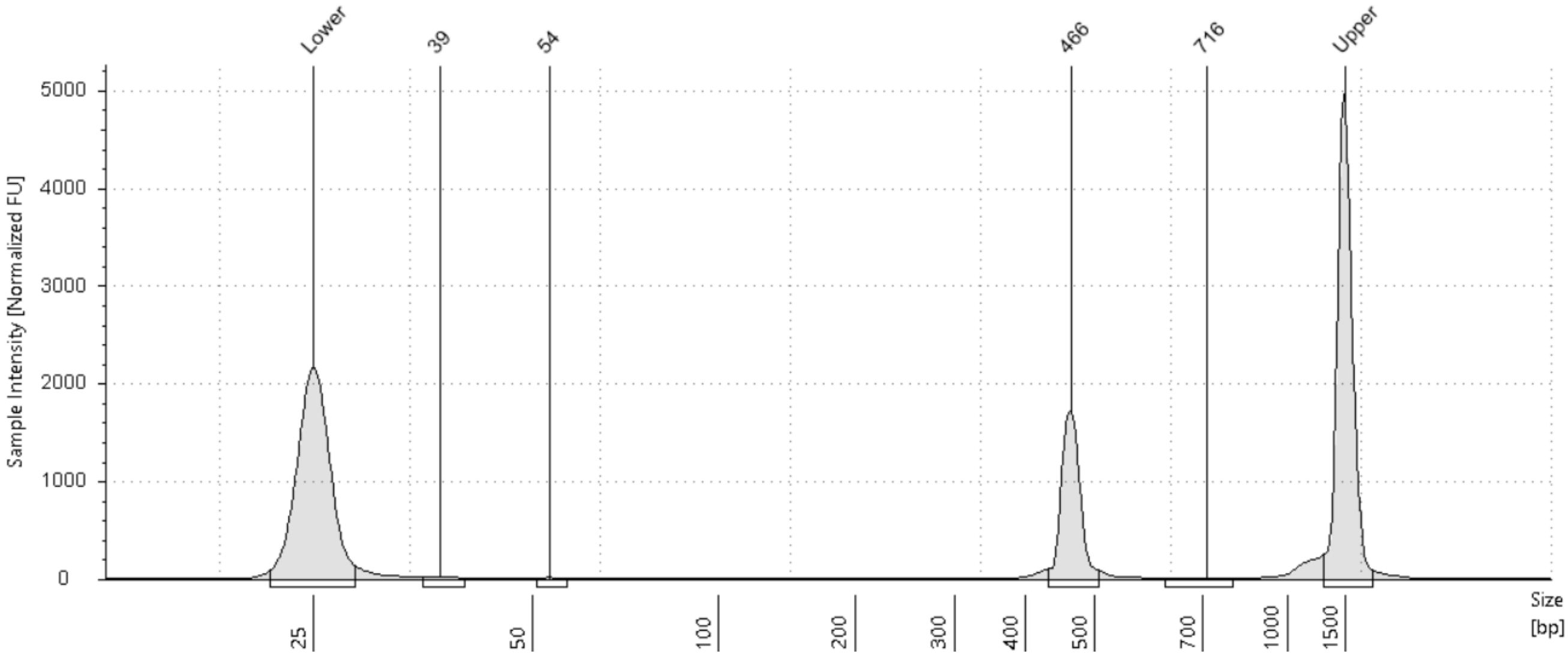

Sample Table

Well	Conc. [ng/µl]	Sample Description	Alert	Observations
F2	2.97	gapdh m8 mb		

Peak Table

Size [bp]	Calibrated Conc. [ng/µl]	Assigned Conc. [ng/µl]	Peak Molarity [nmol/l]	% Integrated Area	Peak Comment	Observations
25	6.23	-	383	-		Lower Marker
39	0.0632	-	2.47	2.13		
54	0.0313	-	0.884	1.05		
466	2.81	-	9.28	94.76		
716	0.0612	-	0.132	2.06		
1500	6.50	6.50	6.67	-		Upper Marker

© Agilent Technologies, Inc. 2021

Source data for Figure 3c

Filename: 2025-04-01 - 23.02.09.D1000

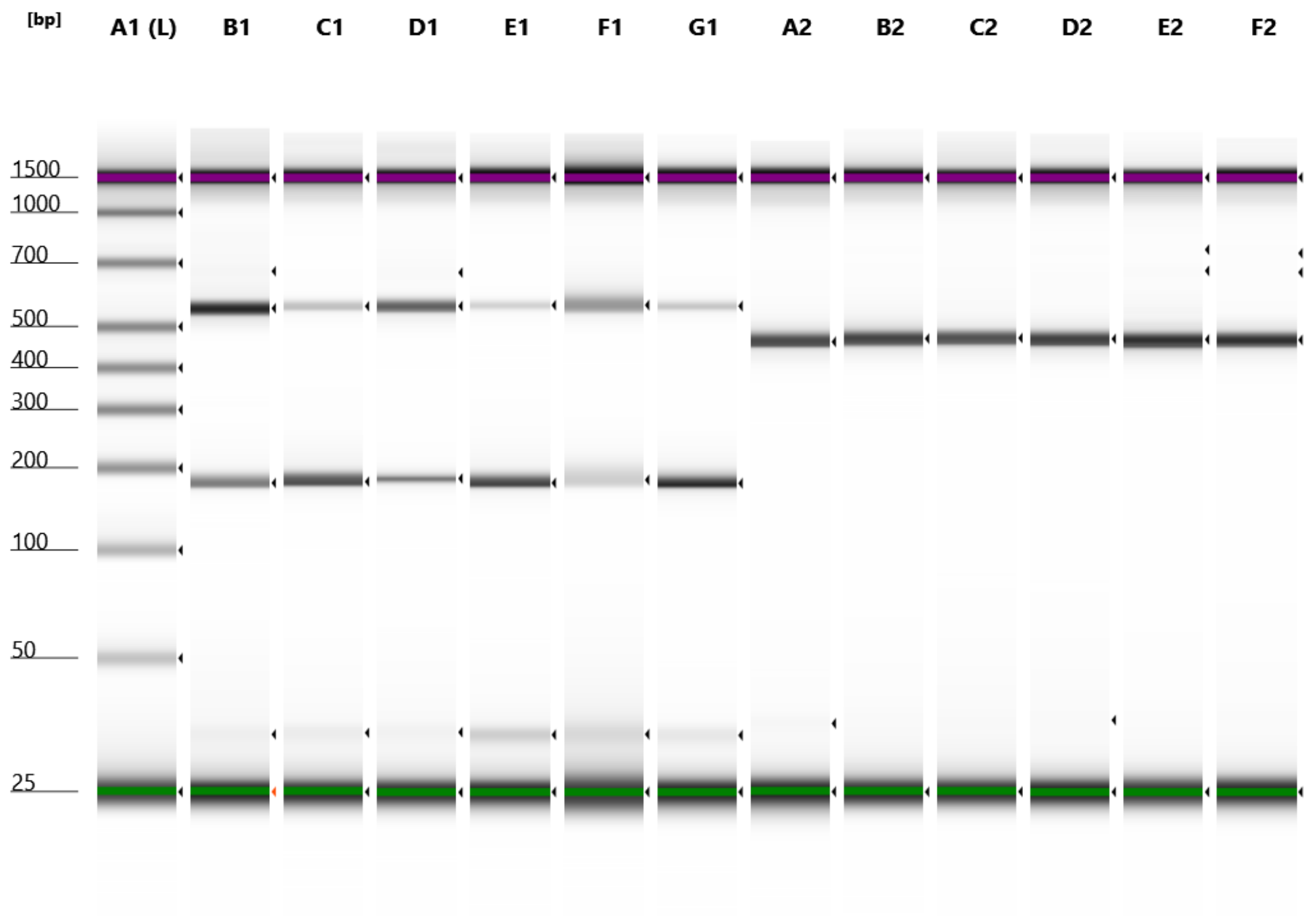

Default image (Contrast 100%)

Sample Info

Well	Conc. [ng/µl]	Sample Description	Alert	Observations
A1	9.27	Ladder		Ladder
B1	4.64	NPTN M3 O		
C1	3.00	NPTN M3 MB		
D1	2.99	NPTN M7 O		
E1	3.01	ntpn m7 mb		
F1	2.44	nptn m8 o		
G1	3.07	nptn m8 mb		
A2	2.48	gapdh m3 o		
B2	2.71	gapdh m3 mb		
C2	2.42	gapdh m7 o		
D2	2.70	gapdh m7 mb		
E2	3.52	gapdh m8 o		
F2	2.97	gapdh m8 mb		

 © Agilent Technologies, Inc. 2021

Source data for Figure 3c

A1: Ladder

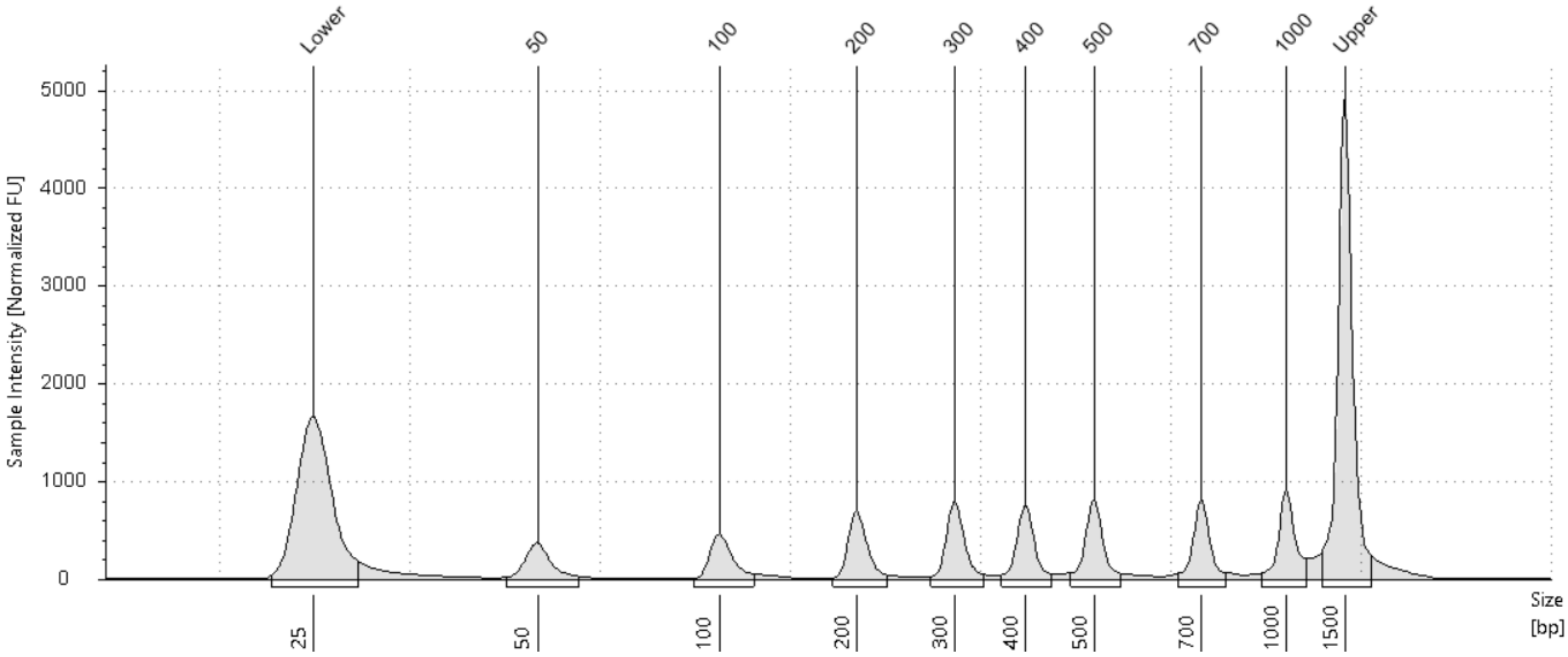

Sample Table

Well	Conc. [ng/µl]	Sample Description	Alert	Observations
A1	9.27	Ladder		Ladder

Peak Table

Size [bp]	Calibrated Conc. [ng/µl]	Assigned Conc. [ng/µl]	Peak Molarity [nmol/l]	% Integrated Area	Peak Comment	Observations
25	5.22	-	321	-		Lower Marker
50	0.849	-	26.1	9.16		
100	0.929	-	14.3	10.03		
200	1.19	-	9.14	12.83		
300	1.29	-	6.61	13.90		
400	1.19	-	4.60	12.90		
500	1.28	-	3.93	13.80		
700	1.20	-	2.63	12.93		
1000	1.34	-	2.06	14.45		
1500	6.50	6.50	6.67	-		Upper Marker

© Agilent Technologies, Inc. 2021

Source data for Figure 3c

B1: NPTN M3 O

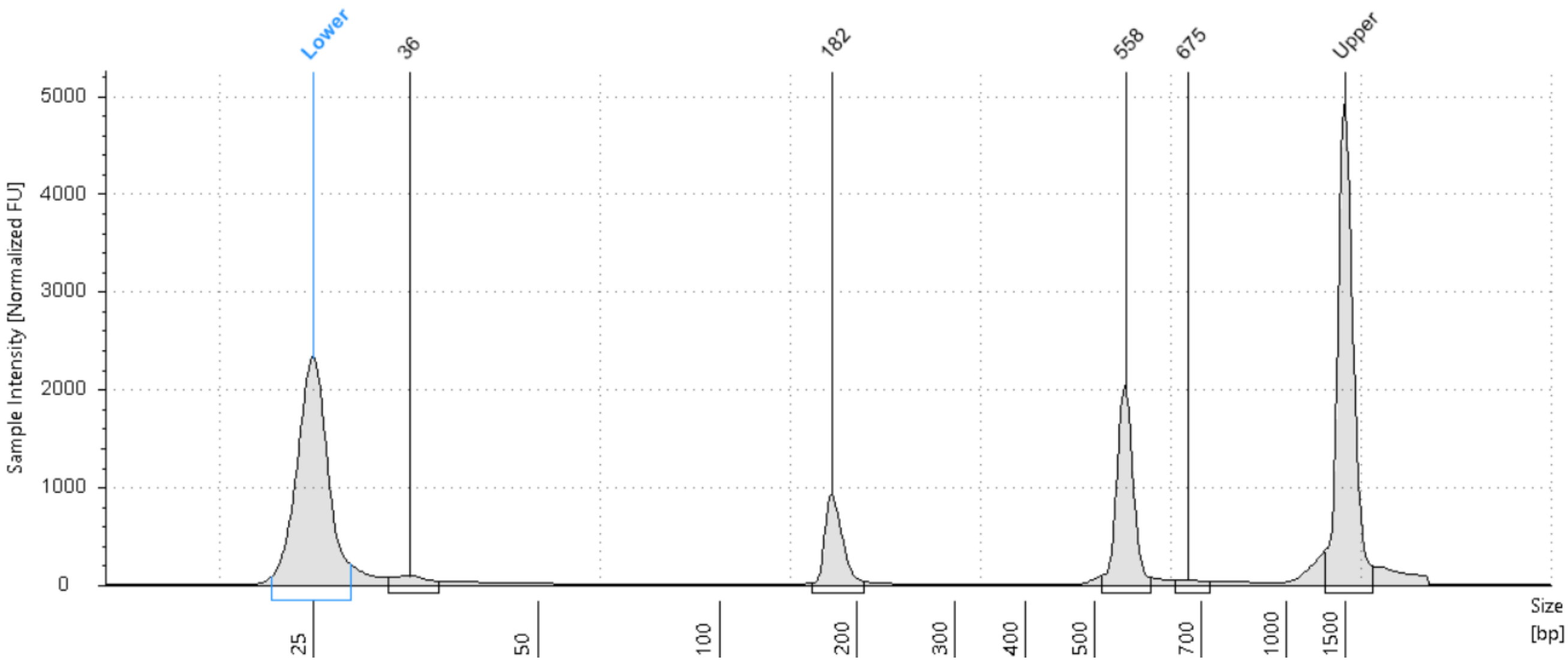

Sample Table

Well	Conc. [ng/µl]	Sample Description	Alert	Observations
B1	4.64	NPTN M3 O		

Peak Table

Size [bp]	Calibrated Conc. [ng/µl]	Assigned Conc. [ng/µl]	Peak Molarity [nmol/l]	% Integrated Area	Peak Comment	Observations
25	6.03	-	371	-		Lower Marker
36	0.276	-	11.9	5.95		
182	1.38	-	11.7	29.79		
558	2.85	-	7.87	61.53		
675	0.127	-	0.289	2.74		
1500	6.50	6.50	6.67	-		Upper Marker

© Agilent Technologies, Inc. 2021

Source data for Figure 3c

C1: NPTN M3 MB

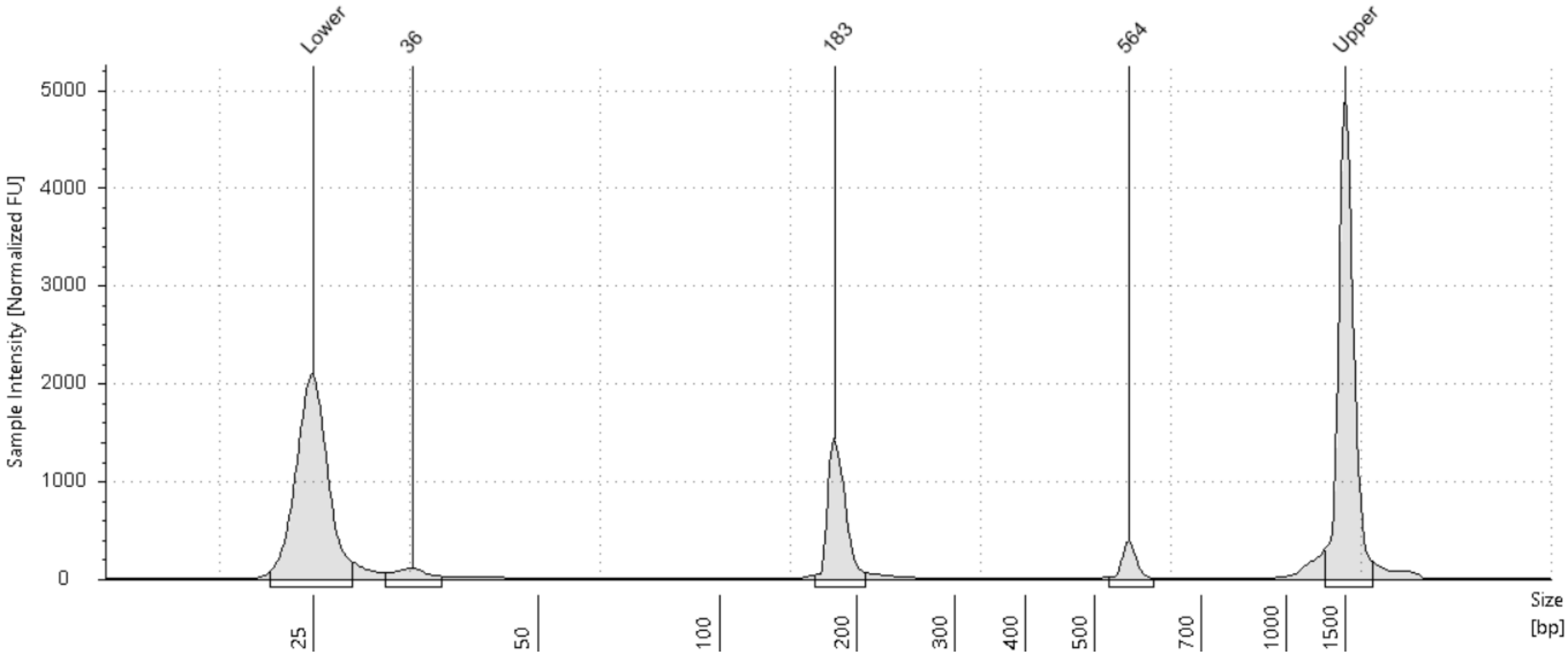

Sample Table

Well	Conc. [ng/µl]	Sample Description	Alert	Observations
C1	3.00	NPTN M3 MB		

Peak Table

Size [bp]	Calibrated Conc. [ng/µl]	Assigned Conc. [ng/µl]	Peak Molarity [nmol/l]	% Integrated Area	Peak Comment	Observations
25	5.74	-	353	-		Lower Marker
36	0.317	-	13.5	10.55		
183	2.20	-	18.4	73.14		
564	0.490	-	1.34	16.32		
1500	6.50	6.50	6.67	-		Upper Marker

© Agilent Technologies, Inc. 2021

Source data for Figure 3c

D1: NPTN M7 O

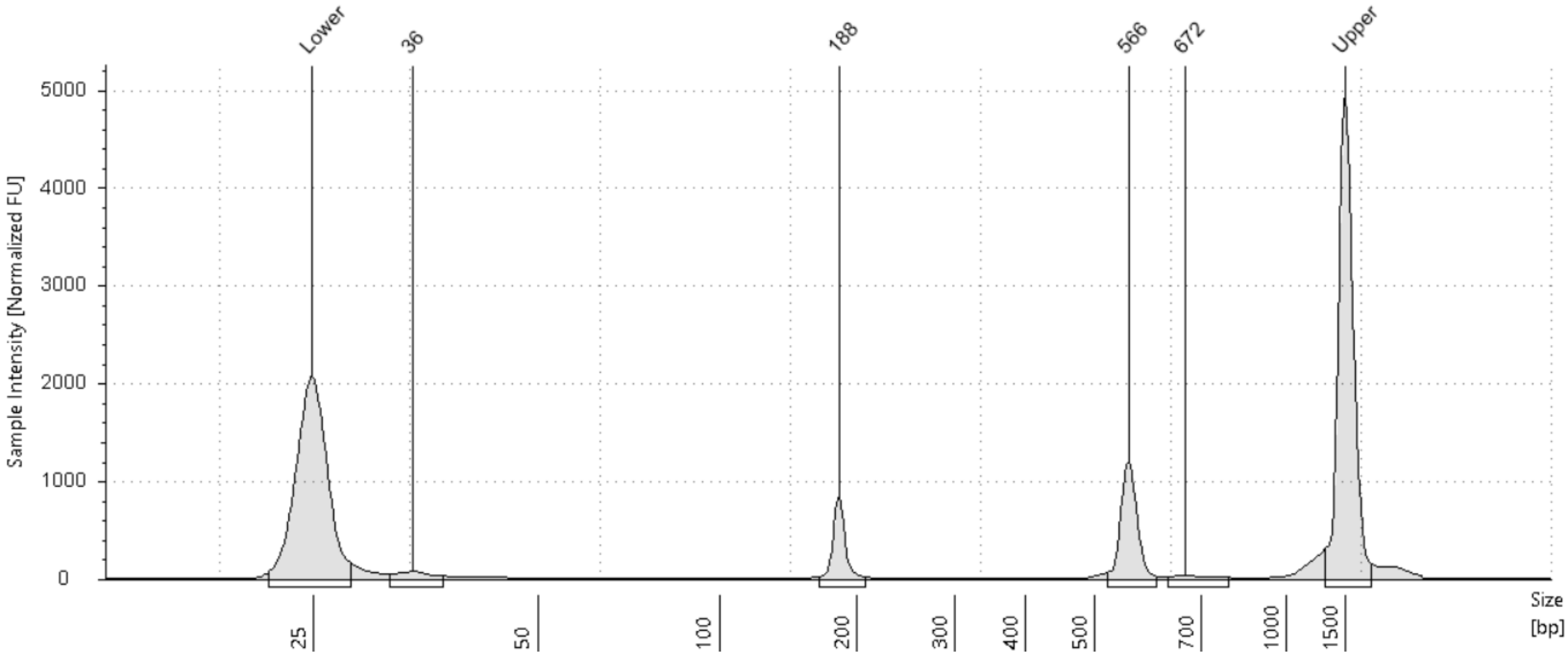

Sample Table

Well	Conc. [ng/µl]	Sample Description	Alert	Observations
D1	2.99	NPTN M7 O		

Peak Table

Size [bp]	Calibrated Conc. [ng/µl]	Assigned Conc. [ng/µl]	Peak Molarity [nmol/l]	% Integrated Area	Peak Comment	Observations
25	5.68	-	349	-		Lower Marker
36	0.233	-	9.89	7.79		
188	0.894	-	7.32	29.89		
566	1.75	-	4.75	58.43		
672	0.116	-	0.266	3.88		
1500	6.50	6.50	6.67	-		Upper Marker

 © Agilent Technologies, Inc. 2021

Source data for Figure 3c

E1: ntpn m7 mb

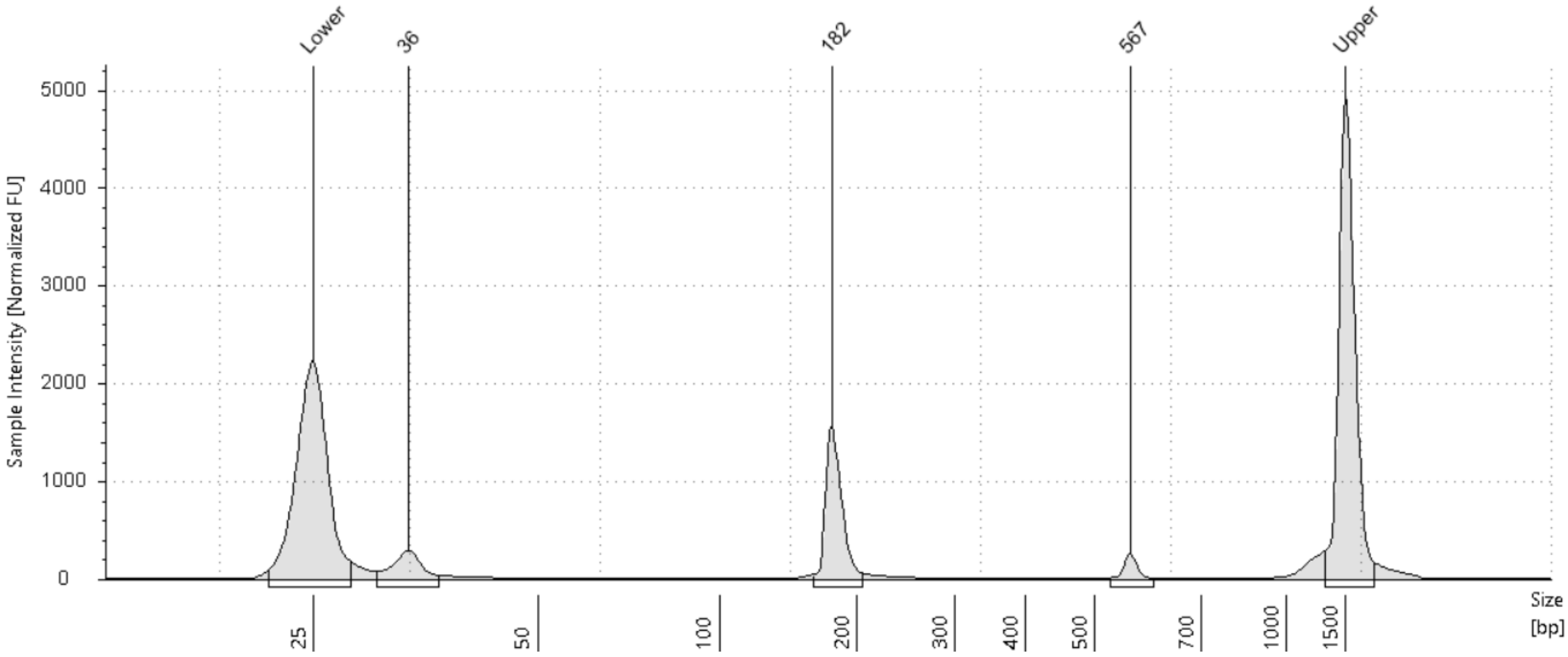

Sample Table

Well	Conc. [ng/µl]	Sample Description	Alert	Observations
E1	3.01	ntpn m7 mb		

Peak Table

Size [bp]	Calibrated Conc. [ng/µl]	Assigned Conc. [ng/µl]	Peak Molarity [nmol/l]	% Integrated Area	Peak Comment	Observations
25	5.61	-	345	-		Lower Marker
36	0.666	-	28.7	22.15		
182	2.05	-	17.3	68.09		
567	0.294	-	0.796	9.76		
1500	6.50	6.50	6.67	-		Upper Marker

© Agilent Technologies, Inc. 2021

Source data for Figure 3c

F1: nptn m8 o

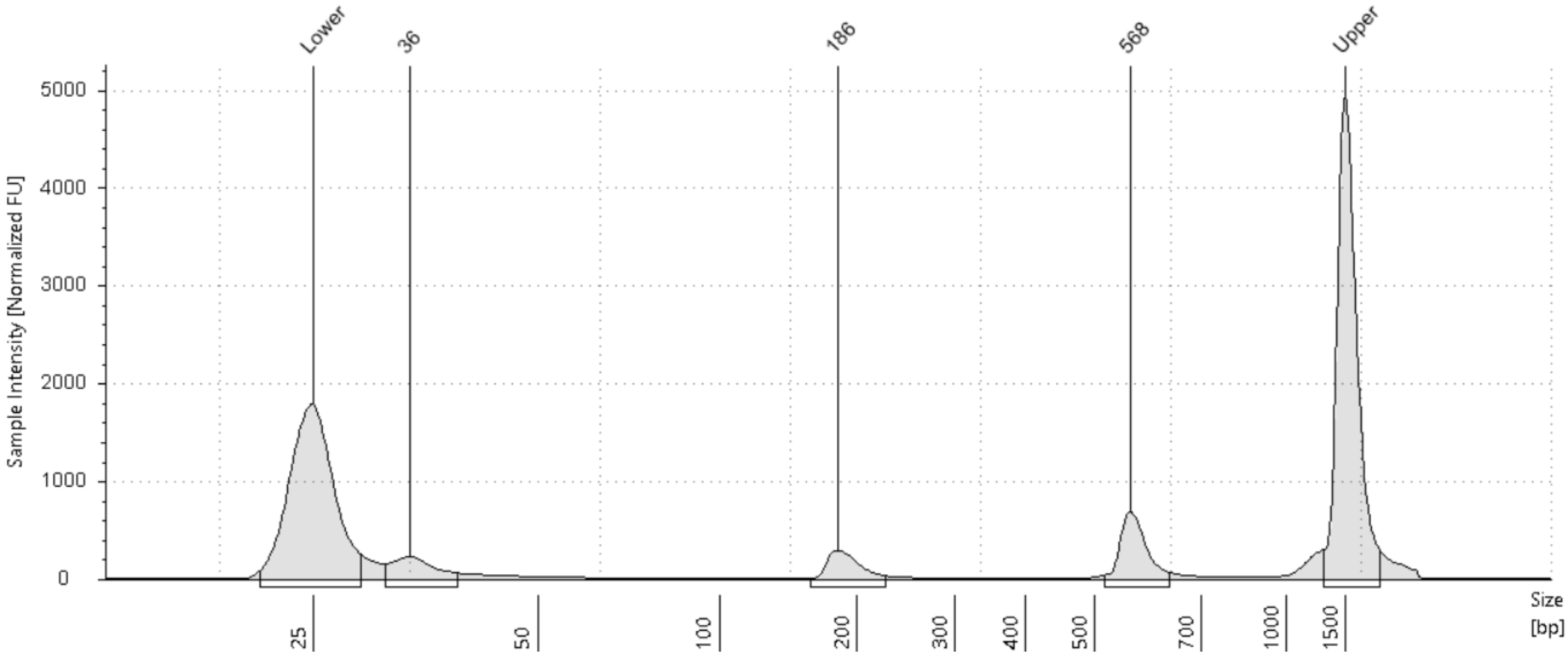

Sample Table

Well	Conc. [ng/µl]	Sample Description	Alert	Observations
F1	2.44	nptn m8 o		

Peak Table

Size [bp]	Calibrated Conc. [ng/µl]	Assigned Conc. [ng/µl]	Peak Molarity [nmol/l]	% Integrated Area	Peak Comment	Observations
25	5.28	-	325	-		Lower Marker
36	0.647	-	27.8	26.58		
186	0.614	-	5.10	25.23		
568	1.17	-	3.18	48.20		
1500	6.50	6.50	6.67	-		Upper Marker

 © Agilent Technologies, Inc. 2021

Source data for Figure 3c

G1: nptn m8 mb

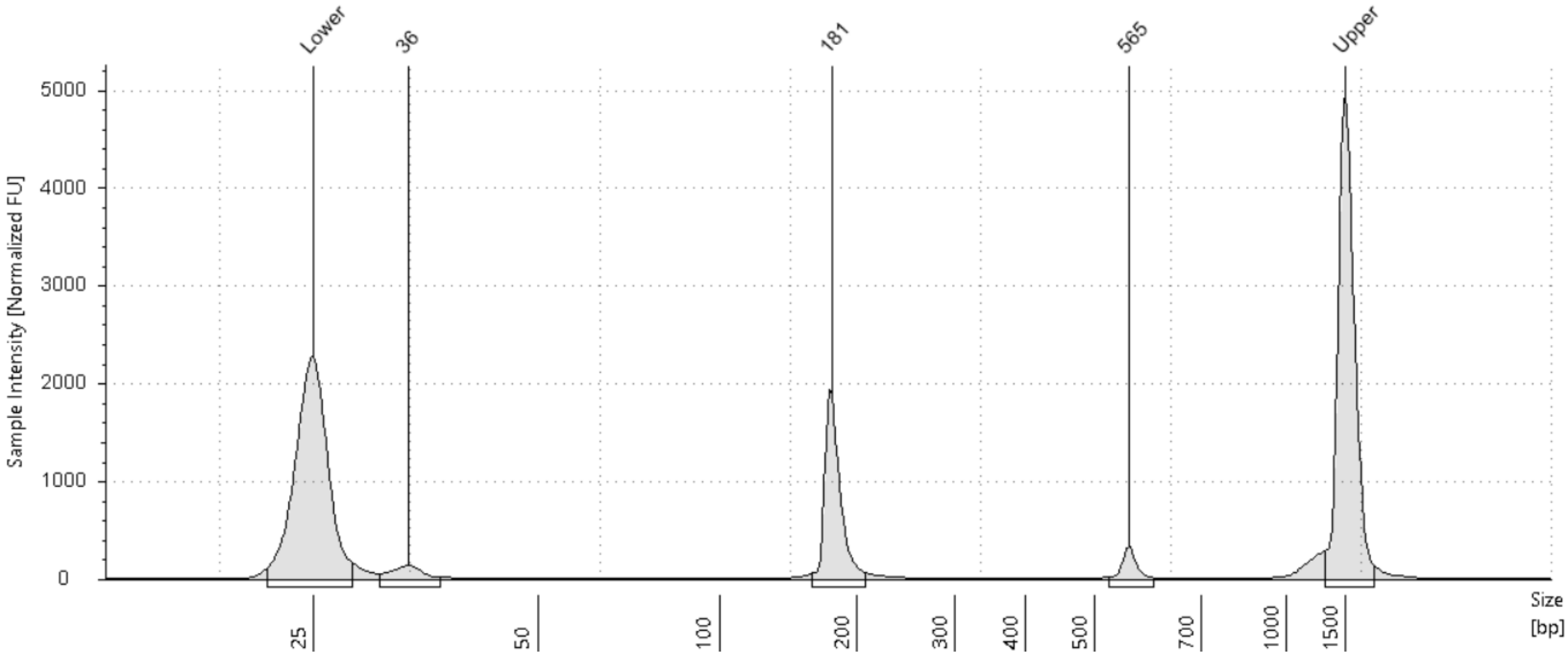

Sample Table

Well	Conc. [ng/µl]	Sample Description	Alert	Observations
Gl	3.07	nptn m8 mb		

Peak Table

Size [bp]	Calibrated Conc. [ng/µl]	Assigned Conc. [ng/µl]	Peak Molarity [nmol/l]	% Integrated Area	Peak Comment	Observations
25	5.88	-	362	-		Lower Marker
36	0.337	-	14.6	10.96		
181	2.36	-	20.0	76.70		
565	0.379	-	1.03	12.34		
1500	6.50	6.50	6.67	-		Upper Marker

© Agilent Technologies, Inc. 2021

Source data for Figure 3c

A2: gapdh m3 o

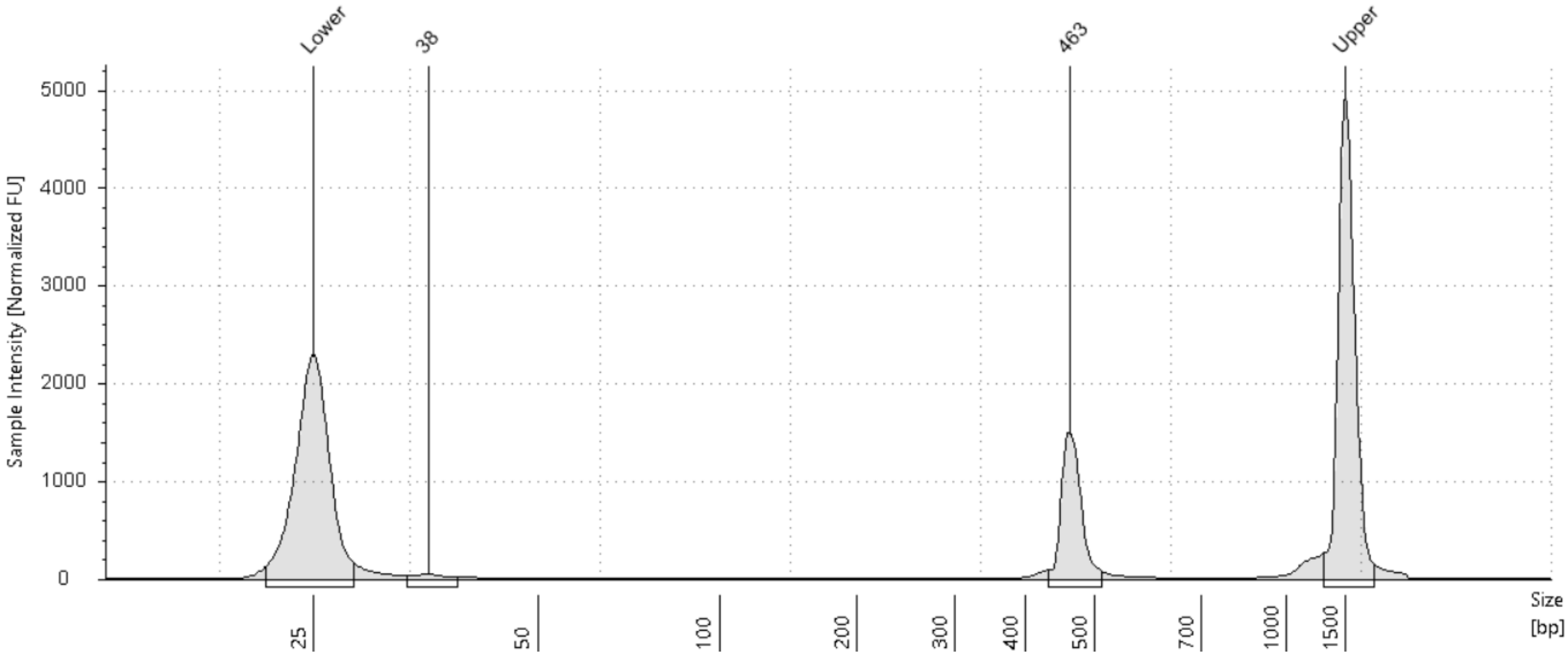

Sample Table

Well	Conc. [ng/µl]	Sample Description	Alert	Observations
A2	2.48	gapdh m3 o		

Peak Table

Size [bp]	Calibrated Conc. [ng/µl]	Assigned Conc. [ng/µl]	Peak Molarity [nmol/l]	% Integrated Area	Peak Comment	Observations
25	6.12	-	377	-		Lower Marker
38	0.127	-	5.18	5.12		
463	2.36	-	7.82	94.88		
1500	6.50	6.50	6.67	-		Upper Marker

© Agilent Technologies, Inc. 2021

Source data for Figure 3c

B2: gapdh m3 mb

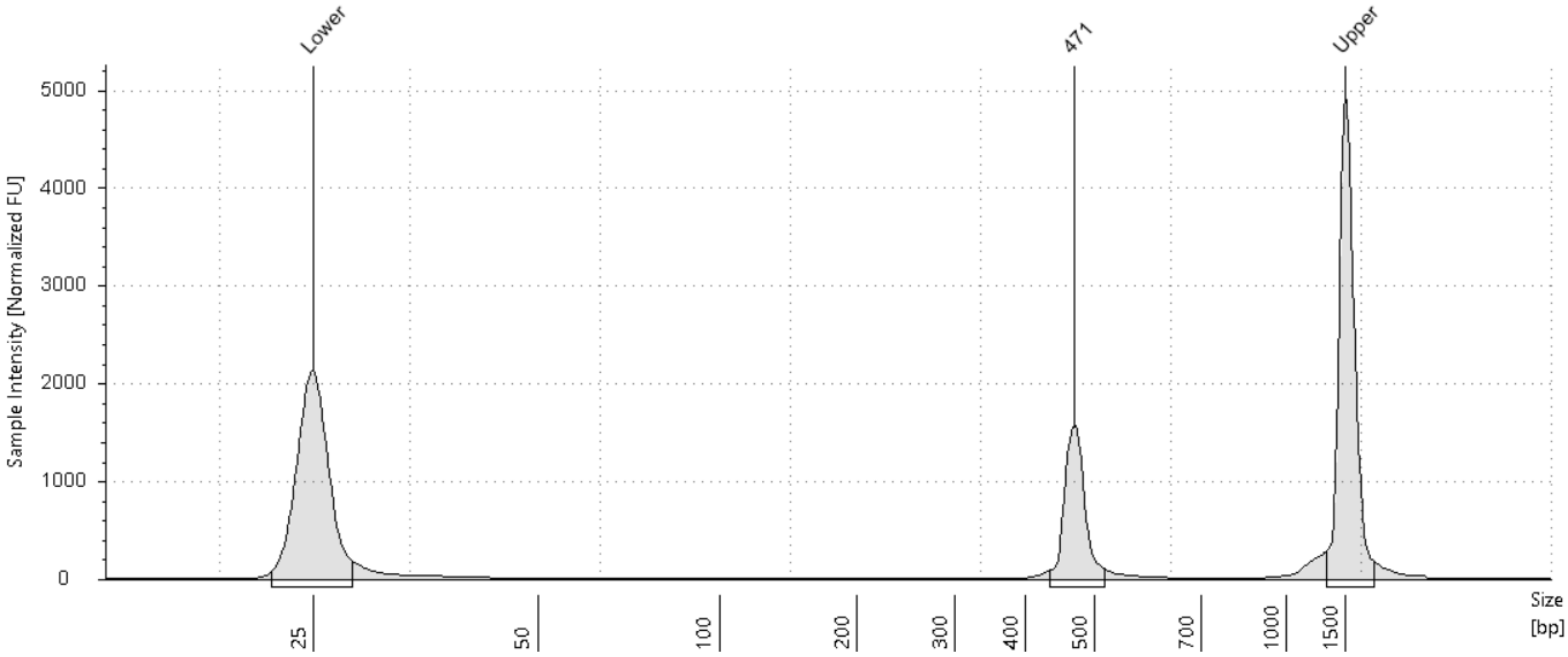

Sample Table

Well	Conc. [ng/µl]	Sample Description	Alert	Observations
B2	2.71	gapdh m3 mb		

Peak Table

Size [bp]	Calibrated Conc. [ng/µl]	Assigned Conc. [ng/µl]	Peak Molarity [nmol/l]	% Integrated Area	Peak Comment	Observations
25	5.79	-	356	-		Lower Marker
471	2.71	-	8.85	100.00		
1500	6.50	6.50	6.67	-		Upper Marker

© Agilent Technologies, Inc. 2021

Source data for Figure 3c

C2: gapdh m7 o

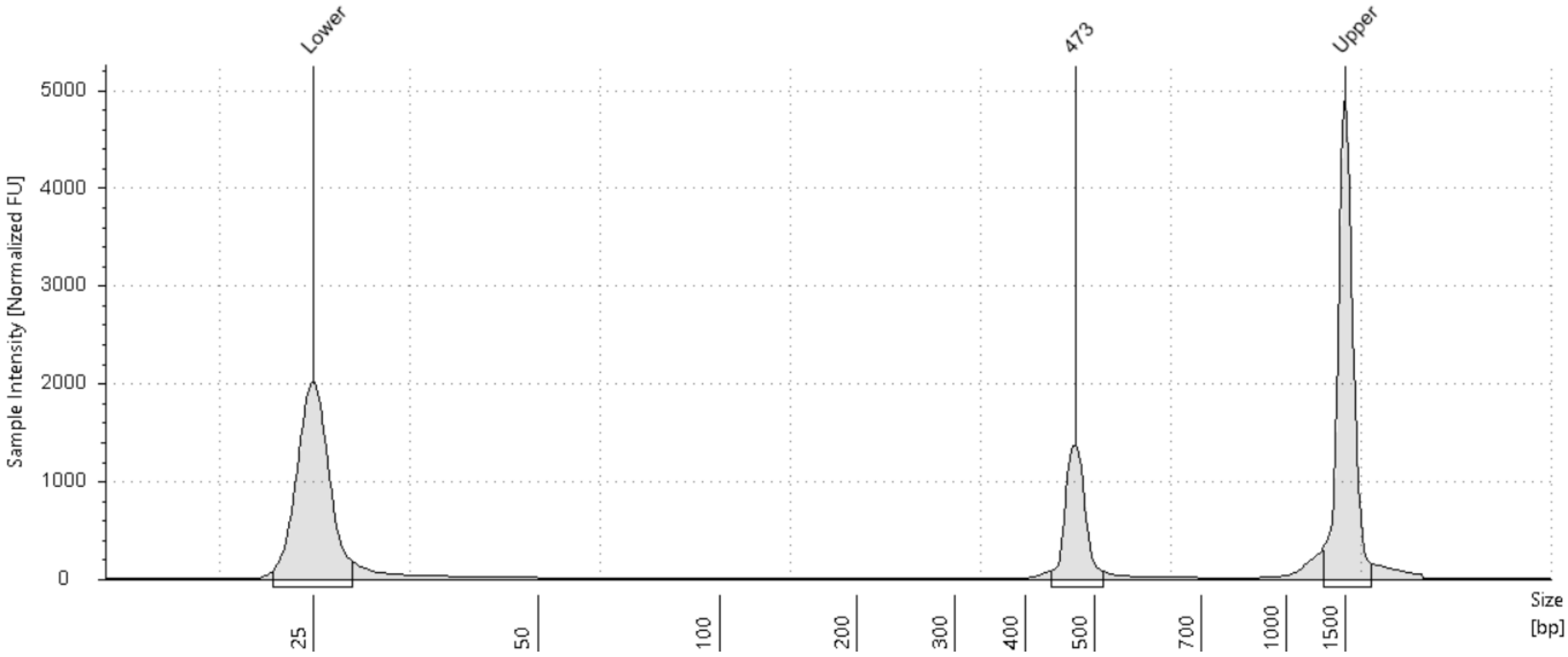

Sample Table

Well	Conc. [ng/µl]	Sample Description	Alert	Observations
C2	2.42	gapdh m7 o		

Peak Table

Size [bp]	Calibrated Conc. [ng/µl]	Assigned Conc. [ng/µl]	Peak Molarity [nmol/l]	% Integrated Area	Peak Comment	Observations
25	5.69	-	350	-		Lower Marker
473	2.42	-	7.89	100.00		
1500	6.50	6.50	6.67	-		Upper Marker

 © Agilent Technologies, Inc. 2021

Source data for Figure 3c

D2: gapdh m7 mb

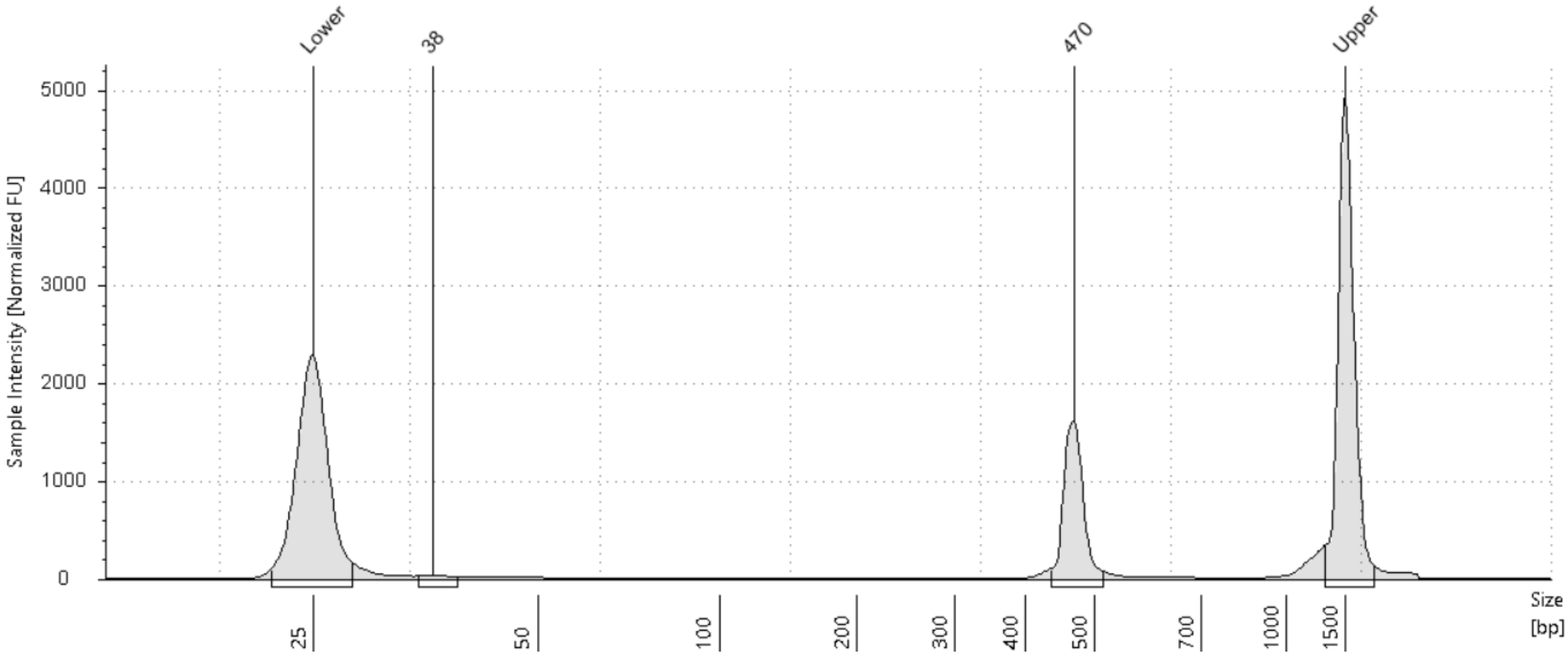

Sample Table

Well	Conc. [ng/µl]	Sample Description	Alert	Observations
D2	2.70	gapdh m7 mb		

Peak Table

Size [bp]	Calibrated Conc. [ng/µl]	Assigned Conc. [ng/µl]	Peak Molarity [nmol/l]	% Integrated Area	Peak Comment	Observations
25	5.89	-	362	-		Lower Marker
38	0.0855	-	3.42	3.17		
470	2.61	-	8.54	96.83		
1500	6.50	6.50	6.67	-		Upper Marker

© Agilent Technologies, Inc. 2021

Source data for Figure 3c

E2: gapdh m8 o

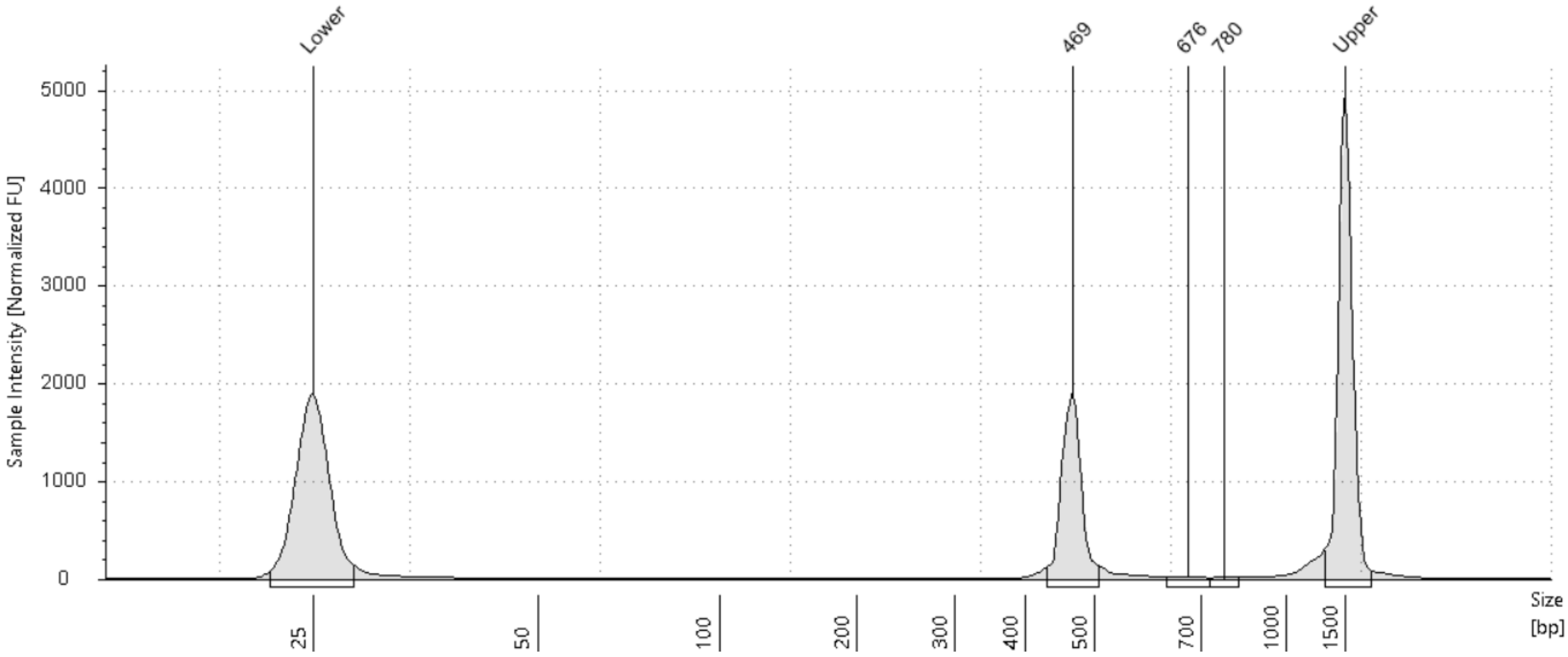

Sample Table

Well	Conc. [ng/µl]	Sample Description	Alert	Observations
E2	3.52	gapdh m8 o		

Peak Table

Size [bp]	Calibrated Conc. [ng/µl]	Assigned Conc. [ng/µl]	Peak Molarity [nmol/l]	% Integrated Area	Peak Comment	Observations
25	5.82	-	358	-		Lower Marker
469	3.41	-	11.2	96.91		
676	0.0708	-	0.161	2.01		
780	0.0379	-	0.0748	1.08		
1500	6.50	6.50	6.67	-		Upper Marker

© Agilent Technologies, Inc. 2021

Source data for Figure 3c

F2: gapdh m8 mb

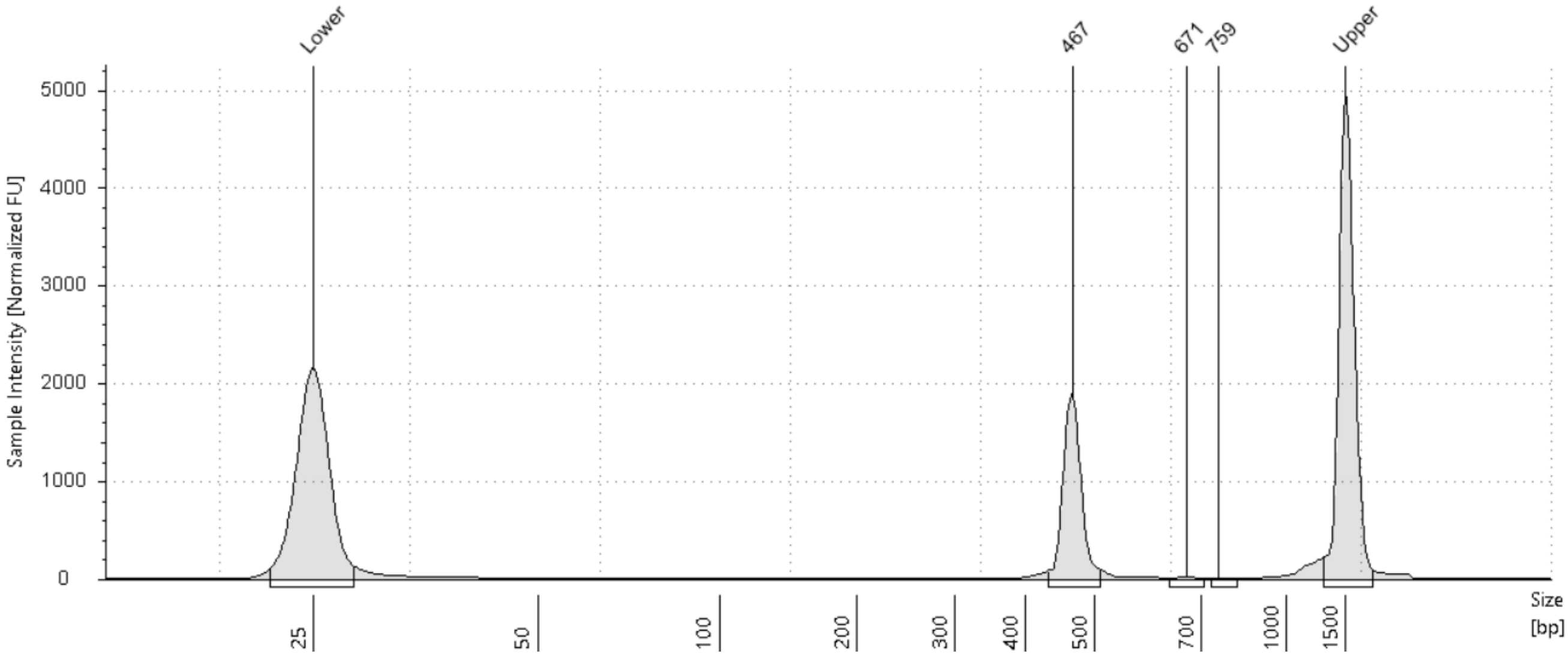

Sample Table

Well	Conc. [ng/µl]	Sample Description	Alert	Observations
F2	2.97	gapdh m8 mb		

Peak Table

Size [bp]	Calibrated Conc. [ng/µl]	Assigned Conc. [ng/µl]	Peak Molarity [nmol/l]	% Integrated Area	Peak Comment	Observations
25	6.04	-	372	-		Lower Marker
467	2.90	-	9.56	97.87		
671	0.0383	-	0.0878	1.29		
759	0.0247	-	0.0502	0.83		
1500	6.50	6.50	6.67	-		Upper Marker

 © Agilent Technologies, Inc. 2021
